# The ERA Registry Annual Report 2023: epidemiology of kidney replacement therapy in Europe, with a focus on age comparisons

**DOI:** 10.1093/ckj/sfag036

**Published:** 2026-02-11

**Authors:** Marin W F Hoekstra, Rianne Boenink, Marjolein Bonthuis, Brittany A Boerstra, Megan E Astley, Iris R Montez de Sousa, Nikola Gjorgjievski, Halima Resic, Nicos Mitsides, Kristine Hommel, Rebecca Guidotti, Inmaculada Marín Sánchez, María F Slon-Roblero, Marta Artamendi, Pazit Beckerman, Lukas Buchwinkler, Ryszard Gellert, Viktorija Kuzema, Adrián Okša, Jordi Comas, Line M T J Heylen, Mathilde Lassalle, Sara Hernandez Ramirez, Shalini Santhakumaran, Nurhan Seyahi, Anders Åsberg, Laurent E Weekers, José M Muñoz-Terol, Sara Trujillo-Alemán, Fatime Memeti Smaili, Mai Ots-Rosenberg, Olafur S Indridason, Kirill S Komissarov, Marc A G J ten Dam, Maria Stendahl, Ana A Galvão, Alberto Ortiz, Vianda S Stel, Anneke Kramer

**Affiliations:** ERA Registry, Amsterdam UMC location University of Amsterdam, Department of Medical Informatics, Amsterdam, The Netherlands; Amsterdam Public Health, Quality of Care, Amsterdam, The Netherlands; ERA Registry, Amsterdam UMC location University of Amsterdam, Department of Medical Informatics, Amsterdam, The Netherlands; Amsterdam Public Health, Quality of Care, Amsterdam, The Netherlands; ERA Registry, Amsterdam UMC location University of Amsterdam, Department of Medical Informatics, Amsterdam, The Netherlands; Amsterdam Public Health, Quality of Care, Amsterdam, The Netherlands; ESPN/ERA Registry, Amsterdam UMC location University of Amsterdam, Department of Medical Informatics, Amsterdam, The Netherlands; ERA Registry, Amsterdam UMC location University of Amsterdam, Department of Medical Informatics, Amsterdam, The Netherlands; Amsterdam Public Health, Quality of Care, Amsterdam, The Netherlands; ERA Registry, Amsterdam UMC location University of Amsterdam, Department of Medical Informatics, Amsterdam, The Netherlands; Amsterdam Public Health, Quality of Care, Amsterdam, The Netherlands; Amsterdam Public Health, Health Behaviours & Chronic Diseases, Amsterdam, The Netherlands; ERA Registry, Amsterdam UMC location University of Amsterdam, Department of Medical Informatics, Amsterdam, The Netherlands; Amsterdam Public Health, Quality of Care, Amsterdam, The Netherlands; ESPN/ERA Registry, Amsterdam UMC location University of Amsterdam, Department of Medical Informatics, Amsterdam, The Netherlands; University Hospital of Nephrology, Skopje, North Macedonia; Faculty of Medicine, Ss. Cyril and Methodius University, Skopje, North Macedonia; Society for Nephrology, Dialysis and transplantation of Bosnia and Herzegovina, Sarajevo, Bosnia and Herzegovina; Cyprus Kidney Care Research and Innovation Unit, Medical School, University of Cyprus, Nicosia, Cyprus; Nephrology Department, Nicosia General Hospital, State Health Services Organization, Nicosia, Cyprus; Cyprus Renal Registry, Health Monitoring Unit, Ministry of Health, Nicosia, Cyprus; Department of nephrology, Holbaek Hospital Denmark, Holbaek, Denmark; Institute of Nephrology, City Hospital Zurich, Zurich, Switzerland; Murcia Renal Registry, Department of Epidemiology, Murcia Regional Health Council, IMIB Arrixaca Murcia, Murcia, Spain; Hospital Universitario de Navarra, Pamplona, Spain; Nephrology Department, San Pedro University Hospital and Registry of patients with Chronic Kidney Disease La Rioja, Spain; Sheba Medical Center and Tel Aviv University School of Medicine, Israel; Austrian Dialysis and Transplantation Registry, Medical University Innsbruck, Innsbruck, Austria; Department of Nephrology and Internal Medicine, Centre of Postgraduate Medical Education, Warsaw, Poland; Paul Stradins Clinical University Hospital, Center of Nephrology, Riga, Latvia; Riga Stradins University, Department of Internal Medicine, Riga, Latvia; Faculty of Medicine, Slovak Medical University, Bratislava, Slovakia; Catalan Renal Registry, Catalan Transplant Organization, Health Department, Generalitat of Catalonia, Barcelona, Spain; Department of Nephrology, Ziekenhuis Oost-Limburg, Synaps Park 1, Genk, Belgium; UHasselt, Faculty of Medicine and Life Sciences, Agoralaan, Diepenbeek, Belgium; Renal Epidemiology and Information Network (REIN) Registry, Agence de la Biomédecine, Saint-Denis, France; Transplant Autonomic Coordination Department, Health Service of Castilla y León, Castilla y León, Spain; UK Renal Registry, Bristol, UK; Department of Nephrology, Istanbul Üniversity-Cerrahpasa, Cerrahpasa Medical Faculty, Istanbul, Turkey; Department of Transplantation Medicine, Oslo University Hospital – Rikshospitalet and Department of Pharmacy, University of Oslo, Oslo, Norway; Department of Nephrology and Transplantation, University of Liège, Liège, Belgium; Department of Nephrology, Hospital Universitario Virgen del Rocío, Seville, Spain; Department of Medicine, University of Seville, Seville, Spain; Health Quality Assessment and Information System Service, Dirección General de Programas Asistenciales, Servicio Canario de la Salud, Canary Islands, Spain; Nephrology Clinic, University Clinical Centre of Kosova, Prishtina, Kosovo; Department of Internal medicine, Institute of Clinical Medicine, Tartu University Hospital, University of Tartu, Tartu, Estonia; Section of Nephrology, Landspitali University Hospital, Reykjavik, Iceland; State Institution “Minsk Scientific and Practical Center for Surgery, Transplantology and Hematology”, Minsk, Belarus; Nefrodata Dutch Renal Registry, Utrecht, The Netherlands; Swedish Renal Registry, Jonkoping Regional Hospital, Jönköping, Sweden; Portuguese Society of Nephrology, Coimbra, Portugal; Department of Nephrology and Hypertension, IIS-Fundacion Jimenez Diaz UAM, Madrid, Spain; RICORS2040, Madrid, Spain; ERA Registry, Amsterdam UMC location University of Amsterdam, Department of Medical Informatics, Amsterdam, The Netherlands; Amsterdam Public Health, Quality of Care, Amsterdam, The Netherlands; ERA Registry, Amsterdam UMC location University of Amsterdam, Department of Medical Informatics, Amsterdam, The Netherlands; Amsterdam Public Health, Quality of Care, Amsterdam, The Netherlands

**Keywords:** dialysis, graft survival, kidney failure, kidney transplantation, patient survival

## Abstract

The European Renal Association (ERA) Registry collects data on patients with kidney failure receiving kidney replacement therapy (KRT). This paper presents a summary of the ERA Registry Annual Report 2023, and focuses specifically on comparisons by age. The complete ERA Registry Annual Report 2023 is available in the Supplementary information. For 2023, data were collected from 34 countries in Europe and countries bordering the Mediterranean Sea. Using these data, incidence and prevalence of KRT, kidney transplantation rates, survival probabilities, and expected remaining lifetimes were calculated. In 2023, the ERA Registry covered 519 million people in the participating countries. The incidence of KRT was 151 per million population (pmp). Among incident patients, 29% were aged ≥75 years, 64% were male, and the most common primary renal disease (PRD) was diabetes mellitus (22%). Most patients (83%) started KRT with haemodialysis (HD), 11% started with peritoneal dialysis (PD), and 6% underwent pre-emptive kidney transplantation. On 31 December 2023, the prevalence of KRT was 1101 pmp. Among prevalent patients, 24% were aged ≥75 years, 62% were male, and the most common PRD was of miscellaneous origin (18%). Moreover, 56% of prevalent patients received HD, 5% received PD, and 39% were living with a functioning graft. In 2023, the kidney transplantation rate was 43 pmp, with 69% of kidneys coming from deceased donors. For patients starting KRT between 2014 and 2018, 5-year survival probability was 51%. The proportions of incident and prevalent patients aged ≥75 varied considerably across European countries. In addition, incident patients aged ≥75 were more often male, and had more often hypertension as PRD compared with younger patients. Only 1% of incident patients aged ≥75 received a pre-emptive kidney transplant, while among prevalent patients of the same age, 22% was living with a functioning graft.

## GRAPHICAL ABSTRACT

**GRAPHICAL ABSTRACT:**
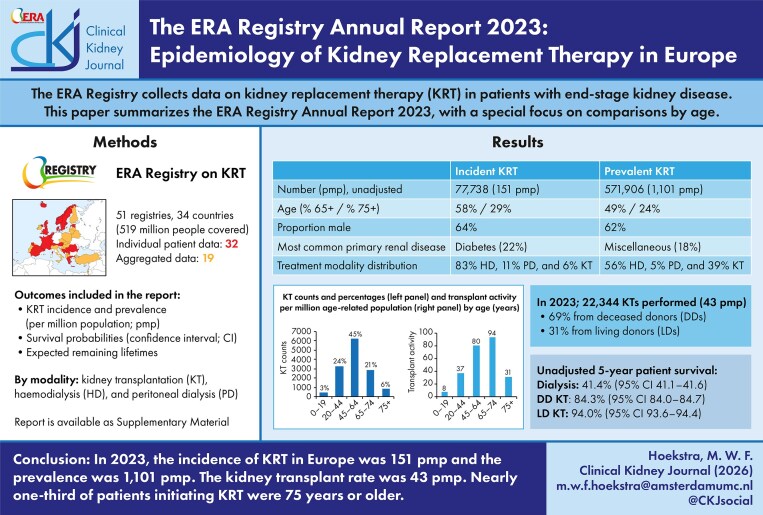

## INTRODUCTION

The European Renal Association (ERA) Registry Annual Report 2023 (Supplementary information) reports the latest data on the epidemiology of kidney replacement therapy (KRT) for patients with kidney failure in Europe and countries bordering the Mediterranean Sea. Data were provided by 51 national or regional renal registries from 34 countries, of which 32 registries from 16 countries contributed individual patient data and 19 registries from 19 countries contributed aggregated data (Appendix 1). The participating registries covered ∼519 million people, corresponding to 65% of the total European population, with registry coverage within participating countries and regions being almost 100%. Compared with the previous annual report, there were some changes in the participating countries [1]. Data from Albania and Ireland were included, whereas Montenegro, Ukraine, and the Sfax region in Tunisia could not participate.

This paper provides a summary of the 2023 ERA Registry Annual Report, offering an overview of the incidence and prevalence of KRT, kidney transplantation rates, patient and graft survival, and life expectancy. In addition, this year’s report focuses specifically on the comparison across age groups. The complete ERA Registry Annual Report 2023, including methodological details, can be found in the Supplementary information.

## RESULTS

### KRT incidence

**Figure 1:**
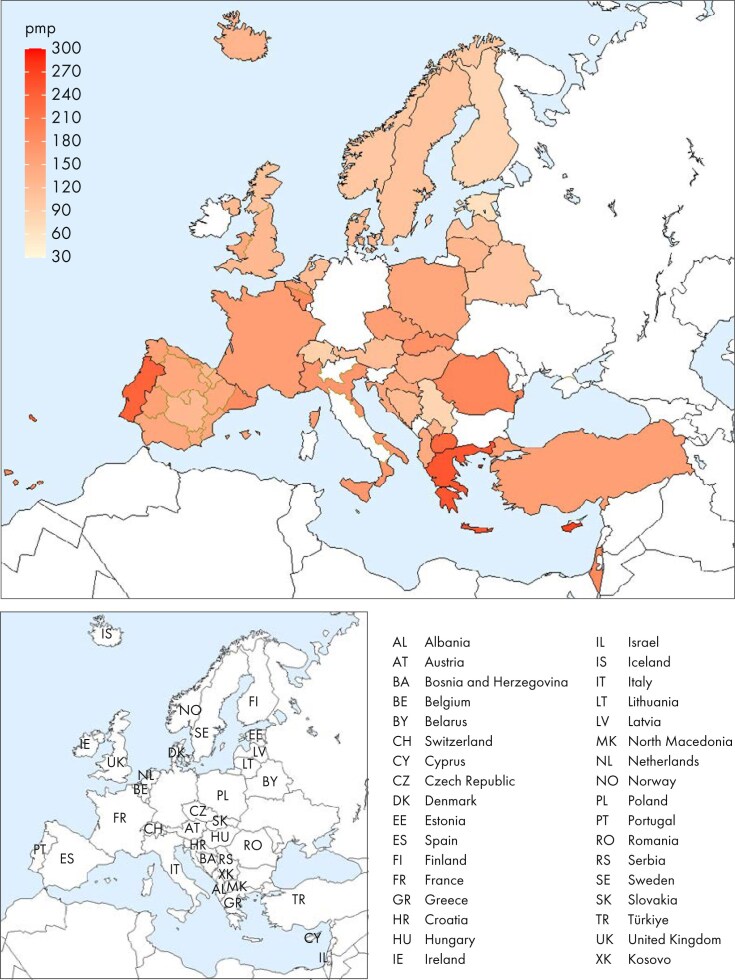
Incidence per million population (pmp) of KRT in 2023 on day 1 by country or region, unadjusted.

**Figure 2:**
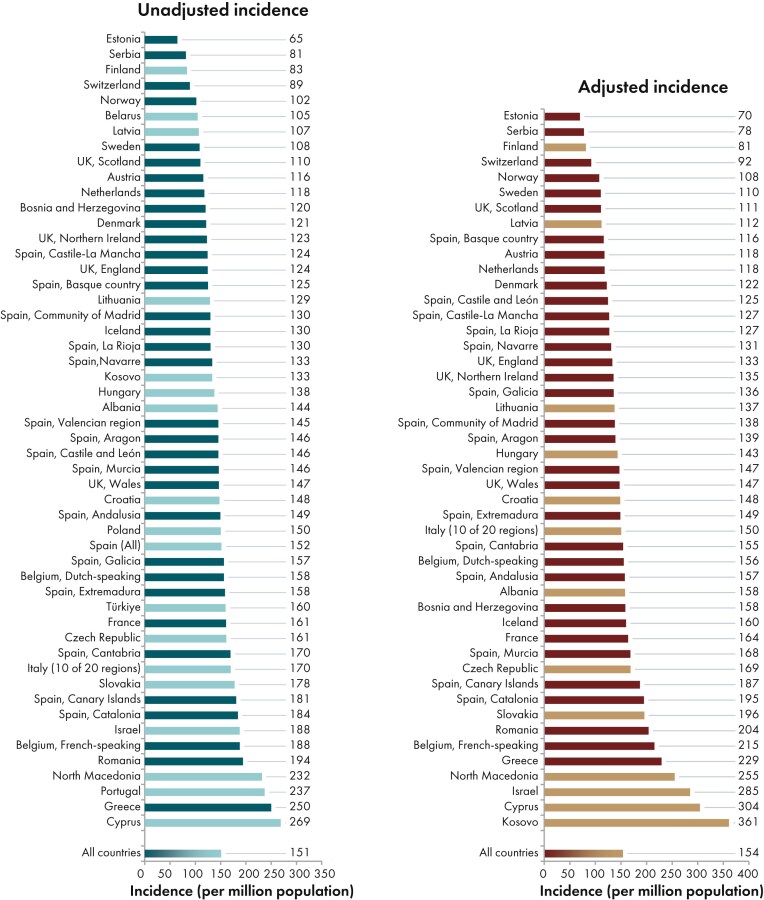
Incidence of KRT per million population in 2023 on day 1 by country or region, unadjusted (left panel) and adjusted (right panel). Registries providing individual patient data are shown as dark bars and registries providing aggregated data as light bars. Adjustment was performed by standardizing the incidence to the age and sex distribution of the EU27 population.

**Figure 3:**
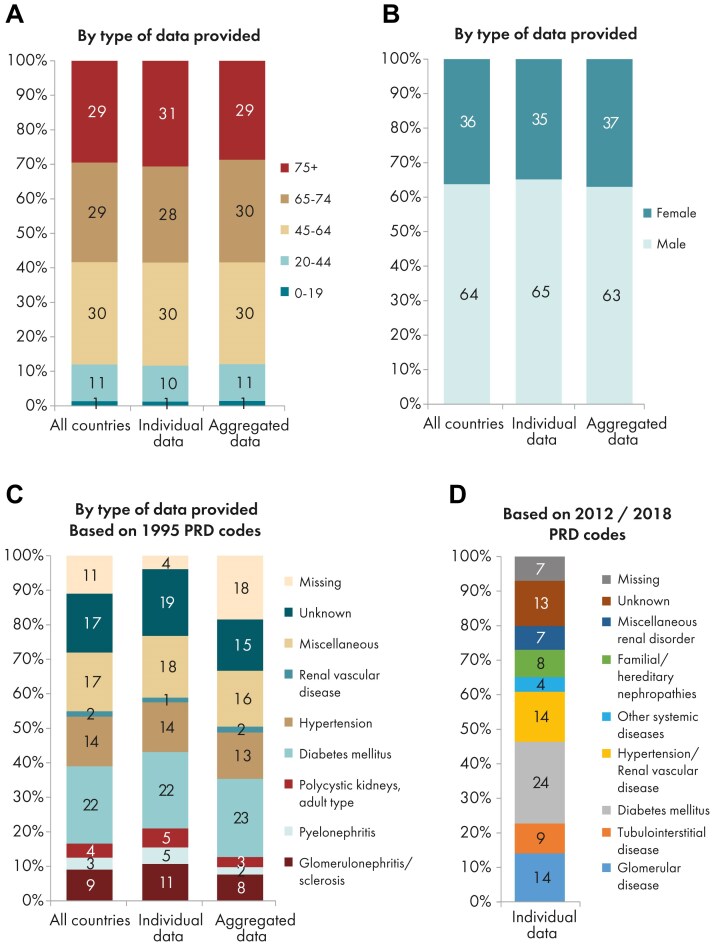
Distribution of (A) age, (B) sex, (C) PRD (1995 ERA codes), and (D) PRD (2012/2018 ERA codes), by type of data provided for incident patients accepted for KRT in 2023 on day 1, unadjusted. See Appendix 1 for a list of countries and regions providing individual patient or aggregated data. Panel (D) is only based on the data from registries providing individual patient data. Bars may not add up to 100% due to rounding.

**Figure 4:**
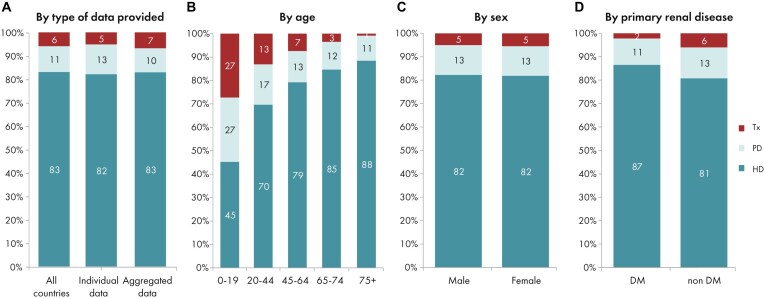
Distribution of treatment modality by (A) type of data provided, (B) age, (C) sex, and (D) PRD (DM and non-DM) for incident patients accepted for KRT in 2023 on day 1, unadjusted. Panels (B)–(D) are only based on the data from registries providing individual patient data. See Appendix 1 for a list of countries and regions providing individual patient or aggregated data. Abbreviation: HD: haemodialysis; PD: peritoneal dialysis; Tx: transplantation.

**Figure 5:**
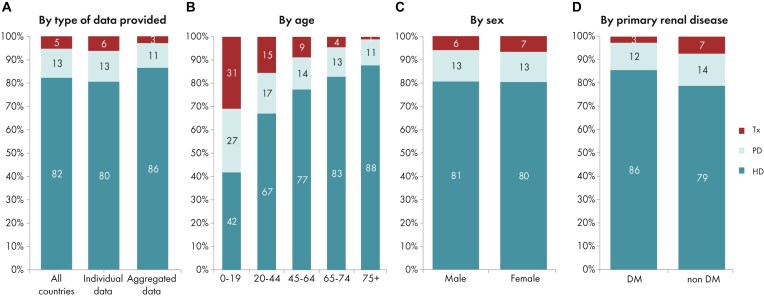
Distribution of treatment modality by (A) type of data provided, (B) age, (C) sex, and (D) PRD (DM and non-DM) for incident patients accepted for KRT in 2023 on day 91, unadjusted. Not all countries providing aggregated data provide information at treatment day 91. Therefore, the percentages in (A) may not be comparable with the percentages from Fig. 4a. Panels (B)–(D) are only based on the data from registries providing individual patient data. See Appendix 1 for a list of countries and regions providing individual patient or aggregated data. Bars may not add up to 100% due to rounding. Abbreviation: HD: haemodialysis; PD: peritoneal dialysis; Tx: transplantation.

In 2023, 77 738 individuals with kidney failure out of a population of 515 million people initiated KRT (Table 1). This corresponds to an unadjusted KRT incidence rate of 151 per million population (pmp) or 1 in 6600 inhabitants (Table 1), which is similar to the 2022 rate of 152 pmp [1]. The unadjusted incidence rate was lowest in Estonia (65 pmp; 1 in 15 400 inhabitants) and Serbia (81 pmp; 1 in 12 300 inhabitants) and highest in Greece (250 pmp; 1 in 4000 inhabitants) and Cyprus (269 pmp; 1 in 3700 inhabitants, Table 1 and Figs 1 and 2). After adjustment for age and sex using the distribution of the European Union’s 27 countries’ (EU27) population (Appendix 1) [2], the differences between countries with the highest and lowest KRT incidence rates remained consistent (Fig. 2). The median age of patients starting KRT was 68.4 year; it was lowest in La Rioja, Spain (62.2 years) and highest in Cantabria, Spain and Dutch-speaking Belgium (73.7 years, Table 1). Among all incident patients, 29% were aged ≥75 years, 64% were male, and the most common primary renal disease (PRD) was diabetes mellitus (22%, Fig. 3). At KRT initiation, 83% of patients received haemodialysis (HD), 11% received peritoneal dialysis (PD), and 6% received a pre-emptive kidney transplant (Fig. 4). For countries providing individual patient data, the initial treatment modality varied among age categories. The proportion of patients receiving HD increased with age, whereas the proportions receiving PD and pre-emptive kidney transplantation decreased with advancing age (Fig. 4). The distribution of initial treatment modalities was comparable between males and females. Patients with diabetes mellitus as their PRD were less likely to receive a pre-emptive kidney transplant than patients with other PRDs (2% vs 6%; Fig. 4). At day 91 after KRT initiation, 82% of all incident patients were receiving HD, 13% were receiving PD, and 5% were living with a functioning graft (Fig. 5).

**Table 1:** Summary data on the unadjusted incidence of KRT in 2023 on day 1 by country or region, the mean and median age at the start of KRT, and the incidence of KRT in patients with diabetes mellitus as PRD.

		Incidence of KRT in 2023 on day 1					
Country/region	General population covered by the registry in thousands	All (*n*)	All (pmp)	Mean age (years)	Median age (years)	DM (*n*)	DM (pmp)
Albania	2734	394	144	61.6	65.0	95	35
Austria^a^	8923	1032	116	64.5	68.2	200	22
Belarus^b^	8444	883	105			198	23
Belgium, Dutch-speaking^c^	6813	1075	158	70.8	73.7	228	33
Belgium, French-speaking^c^	4967	932	188	68.3	70.7	178	36
Bosnia and Herzegovina	3531	424	120	65.1	68.3	112	32
Croatia^d^	3281	484	148	67.2	70.0	119	36
Cyprus	949	255	269	67.3	71.0	91	96
Czech Republic	10 828	1744	161	66.0	69.0	363	34
Denmark	5947	722	121	64.5	67.8	187	31
Estonia	1302	84	65	63.5	64.4	15	12
Finland	5492	458	83	62.0	65.8	158	29
France	68 372	10 974	161	67.3	71.0	2467	36
Greece	10 407	2602	250	70.8	73.3	604	58
Hungary	9600	1321	138	63.8	67.0	491	51
Iceland	386	50	130	64.8	65.8	6	16
Israel	9633	1807	188	66.2	69.5	697	72
Italy (10 of 20 regions)	28 614	4860	170	67.4	71.3	557	19
Kosovo^b^	1688	225	133	65.5	68.0	70	41
Latvia	1732	185	107	61.7	63.0	34	20
Lithuania	2857	368	129	63.6	65.2	60	21
Netherlands	16 626	1958	118	62.8	66.6	421	25
North Macedonia	1826	423	232	65.3	67.0	109	60
Norway	5520	563	102	62.5	66.7	83	15
Poland^d^	37 637	5655	150				
Portugal^e^	10 640	2521	237			768	72
Romania	19 061	3702	194	63.8	66.4	438	23
Serbia	6391	519	81	62.0	65.0	121	19
Slovakia^d^	4472	794	178	62.9	66.0	235	53
Spain (All)	48 085	7287	152	63.5	68.3	1602	33
Spain, Andalusia	8608	1285	149	65.1	68.4	296	34
Spain, Aragon	1346	196	146	65.6	69.5	41	30
Spain, Basque Country	2222	278	125	66.0	68.6	66	30
Spain, Canary Islands	2226	402	181	63.7	65.9	124	56
Spain, Cantabria^c^	590	100	170	70.0	73.7	18	31
Spain, Castile and León^c^	2384	347	146	67.7	69.5	84	35
Spain, Castile-La Mancha^c^	2094	260	124	65.9	67.8	66	32
Spain, Catalonia	7902	1455	184	66.8	70.4	338	43
Spain, Community of Madrid	6872	890	130	62.0	66.4	167	24
Spain, Extremadura	1054	167	158	68.1	70.4	45	43
Spain, Galicia	2703	423	157	67.0	70.0	98	36
Spain, La Rioja	323	42	130	61.5	62.2	10	31
Spain, Murcia	1552	227	146	64.1	67.4	52	34
Spain, Navarre^c^	675	90	133	64.6	66.8	13	19
Spain, Valencian region	5216	758	145	66.4	69.4	152	29
Sweden	10 537	1142	108	64.7	68.4	283	27
Switzerland	8444	752	89	66.4	70.0	133	16
Türkiye^f^	85 372	13 641	160			2883	54
UK, England	53 075	6599	124	60.2	62.7	1766	33
UK, Northern Ireland	1920	236	123	62.5	65.2	52	27
UK, Scotland	5490	603	110	60.4	63.1	180	33
UK, Wales	3164	464	147	62.6	65.3	131	41
							
All countries	514 760	77 738	151	65.0	68.4	16 135	36

DM = diabetes mellitus as PRD.

When cells are left empty, the data are unavailable and could not be used for the calculation of the summary data.

^a^The incidence is underestimated by ∼2% due to one haemodialysis centre not submitting data.

^b^Patients <18 years are not reported.

^c^Patients <20 years are not reported.

^d^Data include dialysis patients only.

^e^Data on PRD are available for dialysis patients only (*N* = 2489, 98.7% of total).

^f^Data on DM are extrapolated from data of 8494 patients (62.3% of total).

### KRT prevalence

**Figure 6:**
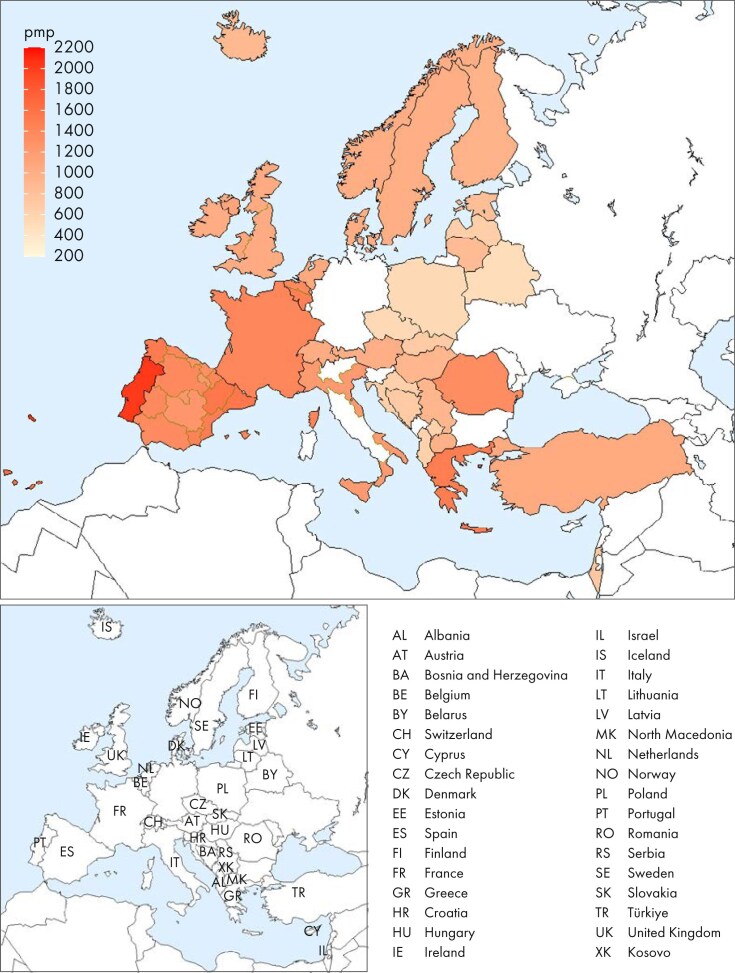
Prevalence per million population (pmp) of KRT on 31 December 2023 by country or region, unadjusted.

**Figure 7:**
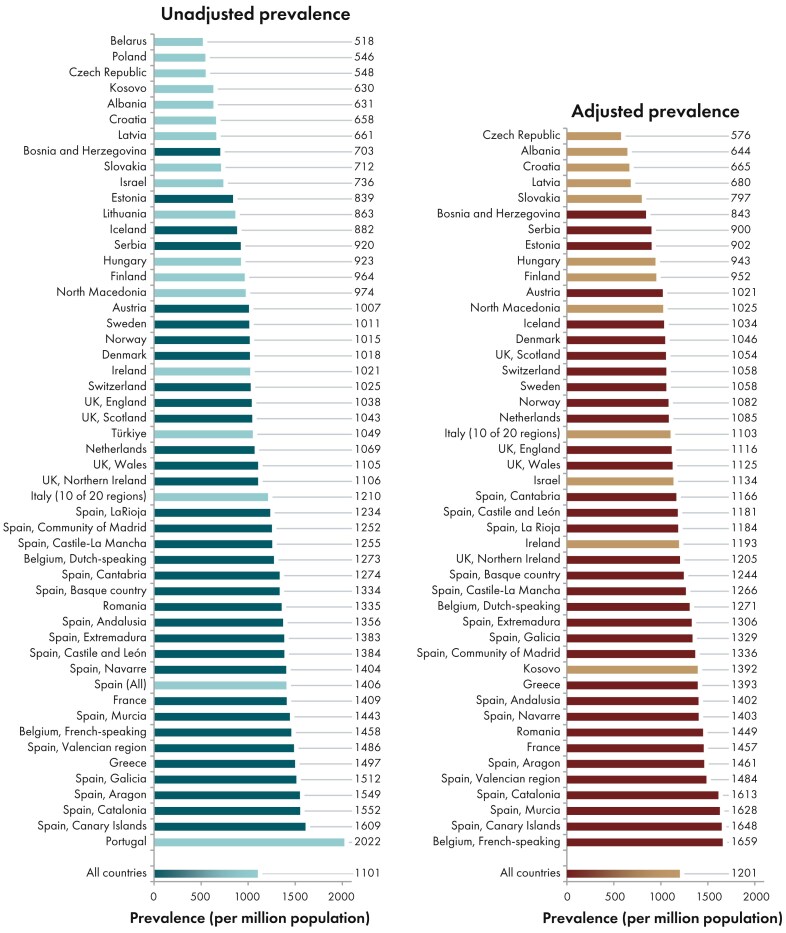
Prevale:nce per million population of KRT on 31 December 2023 by country or region, unadjusted (left panel) and adjusted (right panel). Registries providing individual patient data are shown as dark bars and registries providing aggregated data as light bars. Adjustment was performed by standardizing the prevalence to the age and sex distribution of the EU27 population.

**Figure 8:**
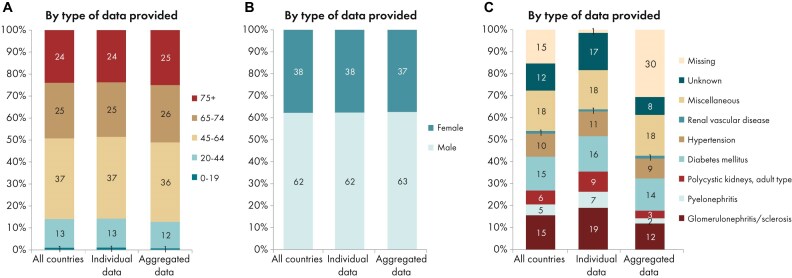
Distribution of (A) age, (B) sex, and (C) PRD (1995 ERA codes) by type of data provided for prevalent patients on KRT on 31 December 2023, unadjusted. See Appendix 1 for a list of countries and regions providing individual patient or aggregated data. Bars may not add up to 100% due to rounding.

**Figure 9:**
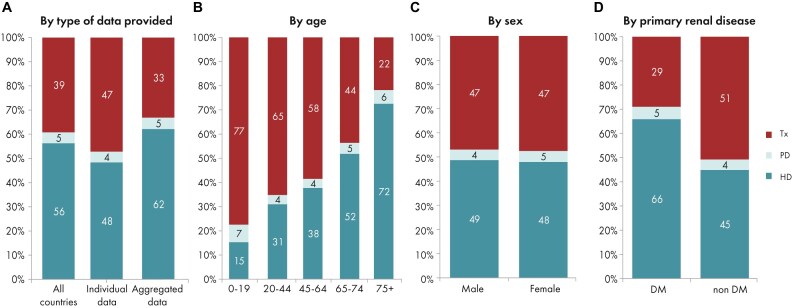
Distribution of treatment modality by (A) type of data provided, (B) age, (C) sex, and (D) PRD (DM and non-DM) for prevalent patients on KRT on 31 December 2023, unadjusted. Panels (B)–(D) are only based on the data from registries providing individual patient data. See Appendix 1 for a list of countries and regions providing individual patient or aggregated data. Bars may not add up to 100% due to rounding. Abbreviation: HD: haemodialysis; PD: peritoneal dialysis; Tx: transplantation.

On 31 December 2023, 571 906 individuals were receiving KRT, representing a prevalence of 1101 pmp, equal to 1 in 910 inhabitants (Table 2). The unadjusted prevalence was lowest in Belarus (518 pmp; 1 in 1900 inhabitants) and Poland (dialysis only, 546 pmp; 1 in 1800 inhabitants), and highest in the Canary Islands, Spain (1609 pmp; 1 in 620 inhabitants) and Portugal (2022 pmp; 1 in 490 inhabitants, Table 2 and Figs 6 and 7). After adjustment for age and sex using the EU27 population distribution [2], country differences in KRT prevalence persisted (Fig. 7). The median age of prevalent patients was 64.5 years, and was lowest in Albania (52.9 years) and highest in Israel (70.9 years, Table 2). Among prevalent patients, 24% were aged ≥75 years, 62% were male, and the most common PRD was of miscellaneous origin (18%, Fig. 8). Of prevalent patients 56% were receiving HD, 39% were living with a kidney transplant, and 5% was receiving PD (Fig. 9). Among countries providing individual patient data, the distribution of treatment modalities varied across age groups. The proportion of patients living with a functioning graft was highest among patients aged 0 to 19 years (77%) and lowest among patients aged ≥75 years (22%, Fig. 9). The distribution of treatment modalities was almost equal between males and females. Patients with diabetes mellitus as PRD were more likely to receive HD (66%) and less likely to have a kidney transplant (29%) than those with other PRDs (HD, 45%; Tx, 51%, Fig. 9).

**Table 2:** Summary data on the unadjusted prevalence of KRT on 31 December 2023 by country or region, the mean and median age on 31 December 2023, and the prevalence of KRT in patients with diabetes mellitus as PRD.

		Prevalent patients on KRT in 2023					
Country/region	General population covered by the registry in thousands	All (*n*)	All (pmp)	Mean age (years)	Median age (years)	DM (*n*)	DM (pmp)
Albania	2734	1724	631	52.8	52.9	455	166
Austria^a^	8923	8985	1007	62.3	64.0	1553	174
Belarus^b^	8444	4374	518			532	63
Belgium, Dutch-speaking^c^	6813	8676	1273	66.8	68.7	1442	212
Belgium, French-speaking^c^	4967	7242	1458	66.6	68.5	1238	249
Bosnia and Herzegovina	3531	2482	703	60.7	62.5	472	134
Croatia^d^	3281	2159	658	66.7	69.0	528	161
Czech Republic^d^	10 828	5937	548	67.0	70.0	1448	134
Denmark	5947	6056	1018	59.9	61.2	998	168
Estonia	1302	1092	839	60.6	61.9	187	144
Finland	5492	5292	964	60.2	62.9	1275	232
France	68 372	96 317	1409	63.6	65.9	15 829	232
Greece	10 407	15 583	1497	66.5	68.5	2746	264
Hungary	9600	8862	923	59.3	61.0	2104	219
Iceland	386	340	882	58.3	59.6	41	106
Ireland	5149	5257	1021	57.9	59.0		
Israel^d^	9633	7087	736	68.3	70.9	3180	330
Italy (10 of 20 regions)	28 614	34 633	1210	62.2	65.7	3396	119
Kosovo^b^	1688	1063	630	61.1	63.0	296	175
Latvia	1732	1145	661	56.8	59.0	142	82
Lithuania^e^	2857	2466	863			196	129
Netherlands	17 162	18 342	1069	61.3	63.3	2432	142
North Macedonia	1826	1779	974	60.2	62.0	334	183
Norway	5520	5605	1015	60.4	62.5	725	131
Poland^d^	37 637	20 536	546			4816	128
Portugal^f^	10 640	21 511	2022	67.7		3709	536
Romania	19 061	25 442	1335	65.0	67.0	2336	123
Serbia	6391	5881	920	61.8	63.8	1020	160
Slovakia^d^	4472	3183	712	64.1	67.0	876	196
Spain (all)	48 085	67 604	1406	61.4	65.0	10 983	228
Spain, Andalusia	8608	11 676	1356	62.1	63.6	1961	228
Spain, Aragon	1346	2086	1549	66.5	68.4	363	270
Spain, Basque Country	2222	2964	1334	62.5	64.8	417	188
Spain, Canary Islands	2226	3581	1609	63.1	64.0	898	403
Spain, Cantabria^c^	590	751	1274	64.9	66.3	115	195
Spain, Castile and León^c^	2384	3300	1384	66.3	67.3	562	236
Spain, Castile-La Mancha^c^	2094	2628	1255	64.9	65.6	444	212
Spain, Catalonia	7902	12 267	1552	63.6	65.3	1895	240
Spain, Community of Madrid	6872	8602	1252	63.0	64.6	1419	206
Spain, Extremadura	1054	1458	1383	64.4	65.5	239	227
Spain, Galicia	2703	4087	1512	64.5	65.9	672	249
Spain, La Rioja	323	399	1234	62.4	62.8	50	155
Spain, Murcia	1552	2239	1443	63.2	64.6	362	233
Spain, Navarre^c^	675	948	1404	63.6	65.4	156	231
Spain, Valencian region	5216	7753	1486	64.3	66.2	1127	216
Sweden	10 537	10 649	1011	60.7	62.6	1795	170
Switzerland	8444	8659	1025	63.5	65.5	1119	133
Türkiye^g^	85 372	89 527	1049			6131	350
UK, England	53 075	55 070	1038	58.4	59.9	10 104	190
UK, Northern Ireland	1920	2123	1106	58.8	60.4	310	161
UK, Scotland	5490	5725	1043	58.0	59.7	961	175
UK, Wales	3164	3498	1105	58.8	60.0	651	206
All countries	519 496	571 906	1101	62.4	64.5	86 360	196

Abbreviations: DM = diabetes mellitus as PRD.

When cells are left empty, the data are unavailable and could not be used for the calculation of the summary data.

^a^The prevalence is underestimated by ∼2% due to one haemodialysis centre not submitting data.

^b^Patients <18 years are not reported.

^c^Patients <20 years are not reported.

^d^Data include dialysis patients only.

^e^Data on DM are extrapolated from data of 1314 patients (53.3% of total).

^f^Data on DM are extrapolated from data of 13 976 patients (65.0% of total).

^g^Data on DM are extrapolated from data of 18 378 patients (20.5% of total).

### Kidney transplantation

**Figure 10:**
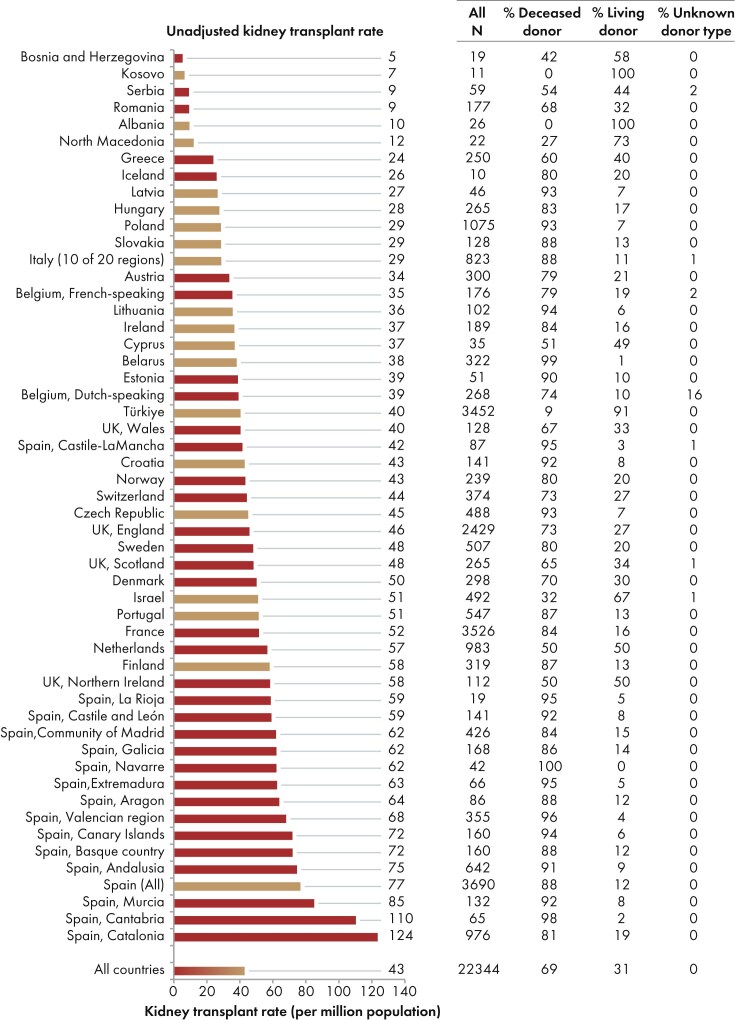
Kidney transplantations performed in 2023 counts (*N*) and per million population by country or region, unadjusted. Registries providing individual patient data are shown as red bars and registries providing aggregated data as orange bars.

**Figure 11:**
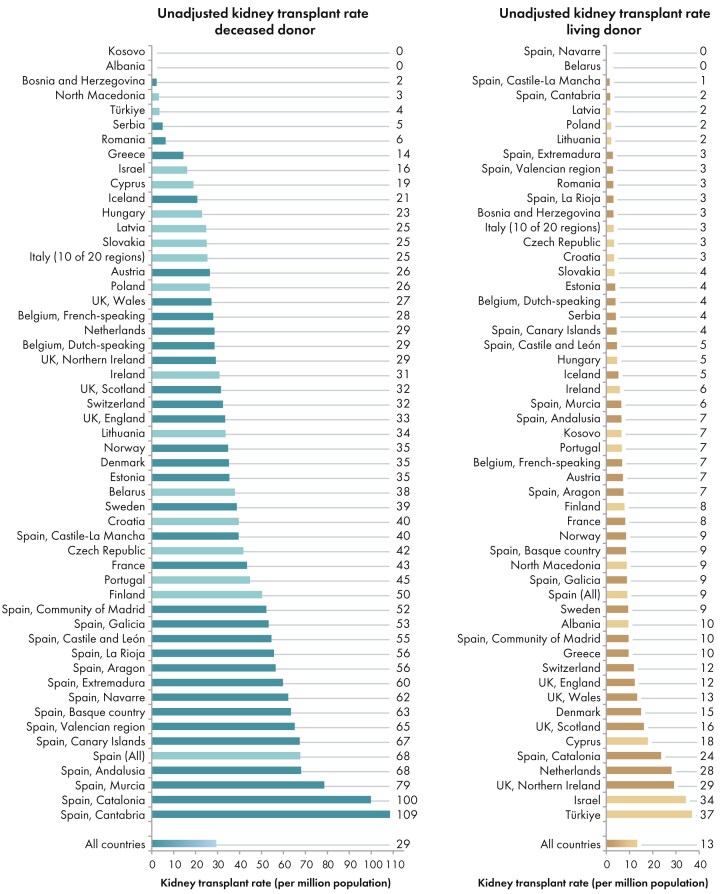
Kidney transplantations performed in 2023 per million population with kidneys from deceased donors (left panel) and living donors (right panel), by country or region, unadjusted. Registries providing individual patient data are shown as dark bars and registries providing aggregated data as light bars.

**Figure 12:**
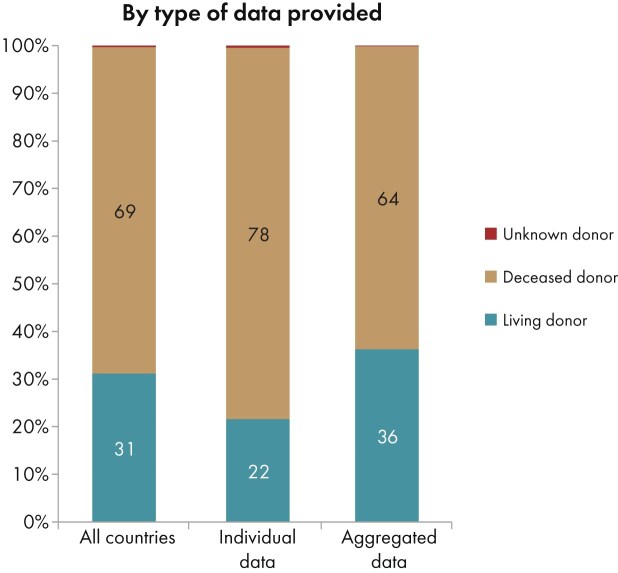
Donor type distribution for kidney transplantations performed in 2023 by type of data provided, unadjusted. See Appendix 1 for a list of countries and regions providing individual patient data or aggregated data.

In 2023, among all participating countries, 22 344 kidney transplantations were performed, of which the majority (69%) were from deceased donors (DD), and 31% from living donors (LD) (Fig. 10). The unadjusted kidney transplantation rate was 43 pmp or 1 in 23 300 inhabitants, representing a slight increase compared with 2022 (40 pmp; 1 in 25 000 inhabitants) [1]. The kidney transplantation rate was lowest in Bosnia and Herzegovina (5 pmp; 1 in 200 000 inhabitants) and Kosovo (7 pmp; 1 in 142 900 inhabitants), and highest in Cantabria, Spain (110 pmp; 1 in 9100 inhabitants) and Catalonia, Spain (124 pmp; 1 in 8100 inhabitants, Fig. 10). The distribution of donor types also varied between countries: in the Spanish region of Navarre all kidney transplants were from DD, whereas in Kosovo and Albania all kidney transplants came from LD (Fig. 10). Consistent with previous years [1, 3, 4], the overall DD kidney transplantation rate was at least twice as high as the LD rate (DD: 29 pmp; 1 in 34 500 inhabitants versus LD: 13 pmp; 1 in 76 900 inhabitants, Fig. 11). The Spanish region Cantabria had the highest DD kidney transplantation rate (109 pmp; 1 in 9200 inhabitants), while Türkiye had the highest LD kidney transplantation rate (37 pmp; 1 in 27 000 inhabitants, Fig. 11). In countries providing individual patient data, the proportion of DD kidney transplants (78%) was higher than in countries providing aggregated data (64%, Fig. 12).

### Survival probability of patients receiving KRT

**Figure 13:**
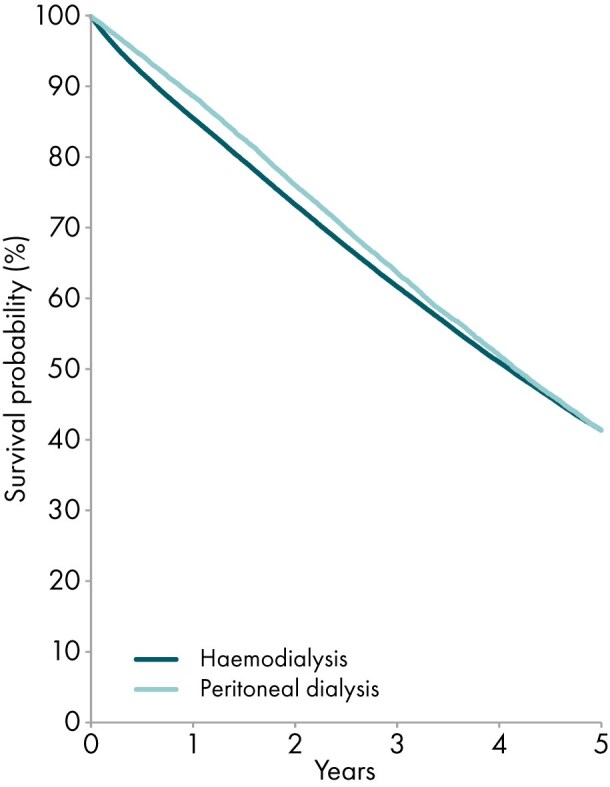
Patient surviv:al by modality (haemodialysis or peritoneal dialysis) for incident dialysis patients from day 91 onwards (cohort 2014–2018), unadjusted. See Appendix 2 for a list of countries and regions providing individual patient data included in the survival analyses.

**Figure 14:**
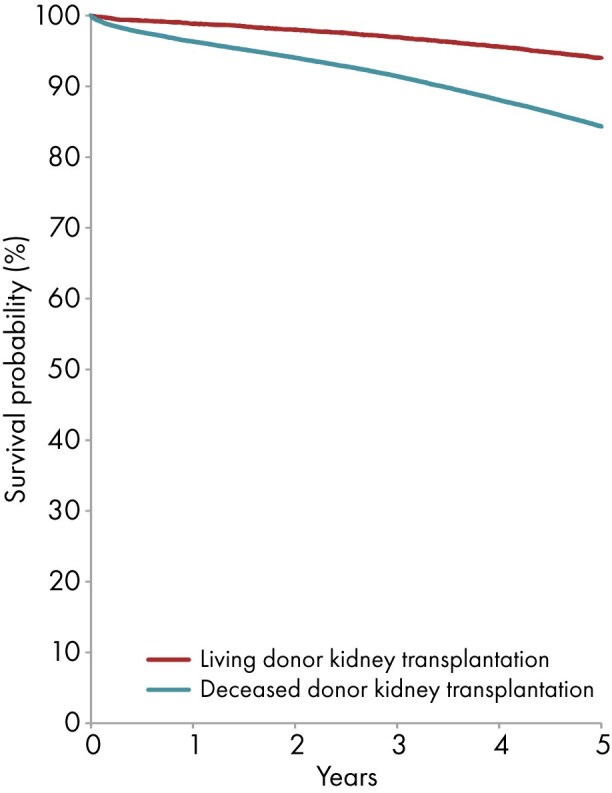
Patient survival in first-time kidney transplant recipients by donor type (deceased or living) from day of transplant (cohort 2014–2018), unadjusted. See Appendix 2 for a list of countries and regions providing individual patient data included in the survival analyses.

Among patients initiating KRT between 2014 and 2018, the unadjusted 5-year patient survival probability was 51.3% [95% confidence interval (CI): 51.1–51.6, Table 3]. For patients initiating dialysis, the unadjusted 5-year survival probability was 41.4% (95% CI 41.1–41.6, Table 3 and Fig. 13). Among patients receiving a first kidney transplant 84.3% (95% CI 84.0–84.7) for DD and 94.0% (95% CI 93.6–94.4) for LD (Table 3 and Fig. 14). The unadjusted 5-year graft survival probability was 75.4% (95% CI 75.0–75.8) after DD kidney transplantation and 88.0% (95% CI 87.4–88.5) after LD kidney transplantation (Table 3).

**Table 3:** One-, two-, and five-year survival probabilities by treatment modality and cohort from day 1 of the start of KRT, dialysis, or from the day of kidney transplantation.

	Survival probabilities as a percentage (95% confidence intervals)				
	Cohort: 2014–2018			Cohort: 2017–2021	
Survival type	1 year	2 year	5 year	1 year	2 year
Patient survival on KRT					
Unadjusted	85.5 (85.3–85.7)	75.5 (75.3–75.7)	51.3 (51.1–51.6)	85.7 (85.5–85.9)	75.7 (75.5–75.9)
Adjusted^a^	88.3 (88.2–88.5)	79.4 (79.2–79.6)	54.0 (53.8–54.3)	88.3 (88.2–88.5)	79.3 (79.1–79.5)
Patient survival on dialysis					
Unadjusted	84.4 (84.3–84.6)	72.8 (72.6–73.0)	41.4 (41.1–41.6)	84.7 (84.6–84.9)	73.3 (73.1–73.5)
Adjusted^a^	86.7 (86.6–86.9)	76.4 (76.2–76.6)	46.7 (46.4–47.0)	87.1 (87.0–87.3)	77.0 (76.8–77.2)
Patient survival after a first kidney transplantation (deceased donor)					
Unadjusted	96.3 (96.1–96.5)	94.1 (93.8–94.3)	84.3 (84.0–84.7)	95.8 (95.6–96.0)	93.0 (92.8–93.3)
Adjusted^b^	98.2 (98.1–98.3)	97.0 (96.9–97.2)	91.6 (91.3–91.9)	98.1 (98.0–98.2)	96.7 (96.5–96.9)
Graft survival after a first kidney transplantation (deceased donor)					
Unadjusted	91.1 (90.8–91.4)	87.7 (87.4–88.0)	75.4 (75.0–75.8)	90.9 (90.6–91.2)	87.2 (86.9–87.6)
Adjusted^b^	93.3 (93.0–93.5)	90.6 (90.4–90.9)	80.6 (80.2–81.0)	93.4 (93.1–93.6)	90.6 (90.3–90.9)
Patient survival after a first kidney transplantation (living donor)					
Unadjusted	98.8 (98.6–99.0)	98.0 (97.8–98.2)	94.0 (93.6–94.4)	98.8 (98.6–99.0)	97.9 (97.6–98.1)
Adjusted^b^	99.1 (99.0–99.3)	98.5 (98.3–98.7)	95.2 (94.8–95.6)	99.1 (98.9–99.3)	98.4 (98.1–98.6)
Graft survival after a first kidney transplantation (living donor)					
Unadjusted	96.5 (96.2–96.8)	95.0 (94.6–95.3)	88.0 (87.4–88.5)	96.9 (96.6–97.2)	95.3 (94.9–95.7)
Adjusted^b^	96.4 (96.1–96.8)	94.8 (94.4–95.2)	87.6 (87.0–88.2)	96.8 (96.5–97.2)	95.1 (94.7–95.5)

^a^Analyses were adjusted using fixed values: age (67 years), sex (63% male), and PRD (24% diabetes mellitus, 19% hypertension/renal vascular disease, 11% glomerulonephritis, and 46% other causes).

^b^Analyses were adjusted using fixed values: age (50 years), sex (63% male), and PRD (14% diabetes mellitus, 10% hypertension/renal vascular disease, 23% glomerulonephritis, and 53% other causes).

See Appendix 2 for a list of countries and regions providing individual patient data that were included in the survival analyses.

### Expected remaining lifetime

**Figure 15:**
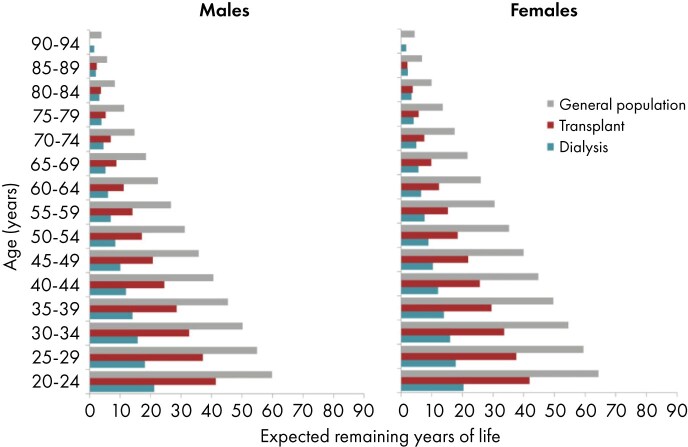
Expected remaining years of life in the general population and for prevalent dialysis and kidney transplant patients (cohort 2019–2023) for males (left panel) and females (right panel), by age. See Appendix 2 for a list of countries and regions providing individual patient data included in the expected remaining years of life analyses.

Between 2019 and 2023, for both males and females, the expected remaining lifetime of prevalent KRT patients was consistently lower than the general population across all age groups (Fig. 15). On average, life expectancy was 68% lower in males and 71% lower in females receiving dialysis compared with the general population. For patients living with a functioning graft, the average expected remaining lifetime was 45% lower in males and 49% in females (Fig. 15).

### Comparisons by age

#### Incidence

**Figure 16:**
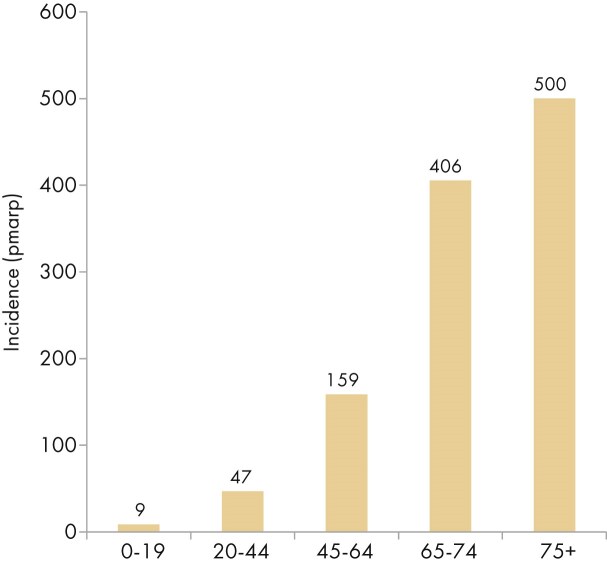
Incidence of KRT per million age-related population (pmarp) in 2023 on day 1 by age, unadjusted.

**Figure 17:**
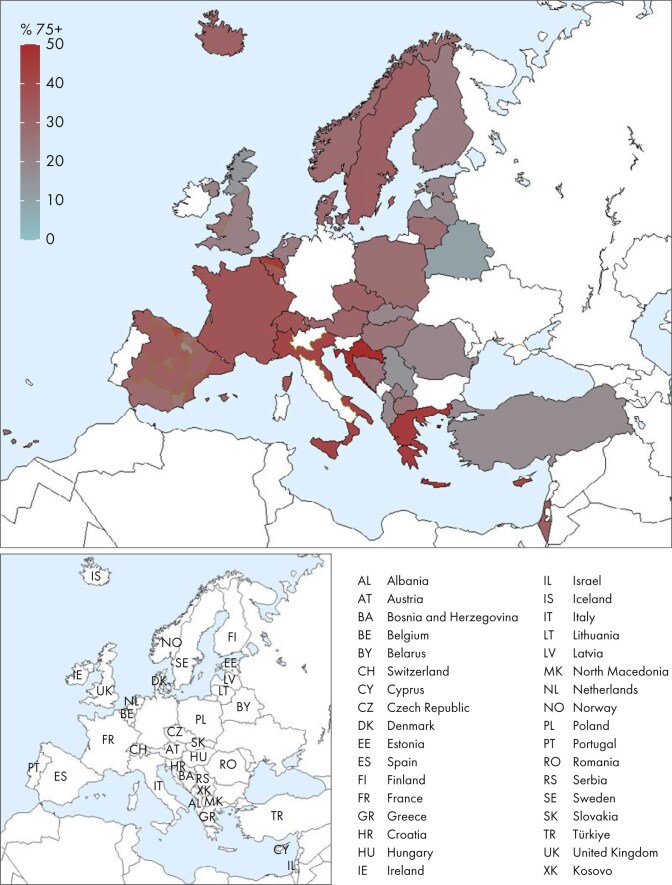
Percentage of incident patients aged ≥75 years among those accepted for KRT in 2023 on day 1 by country or region, unadjusted.

**Figure 18:**
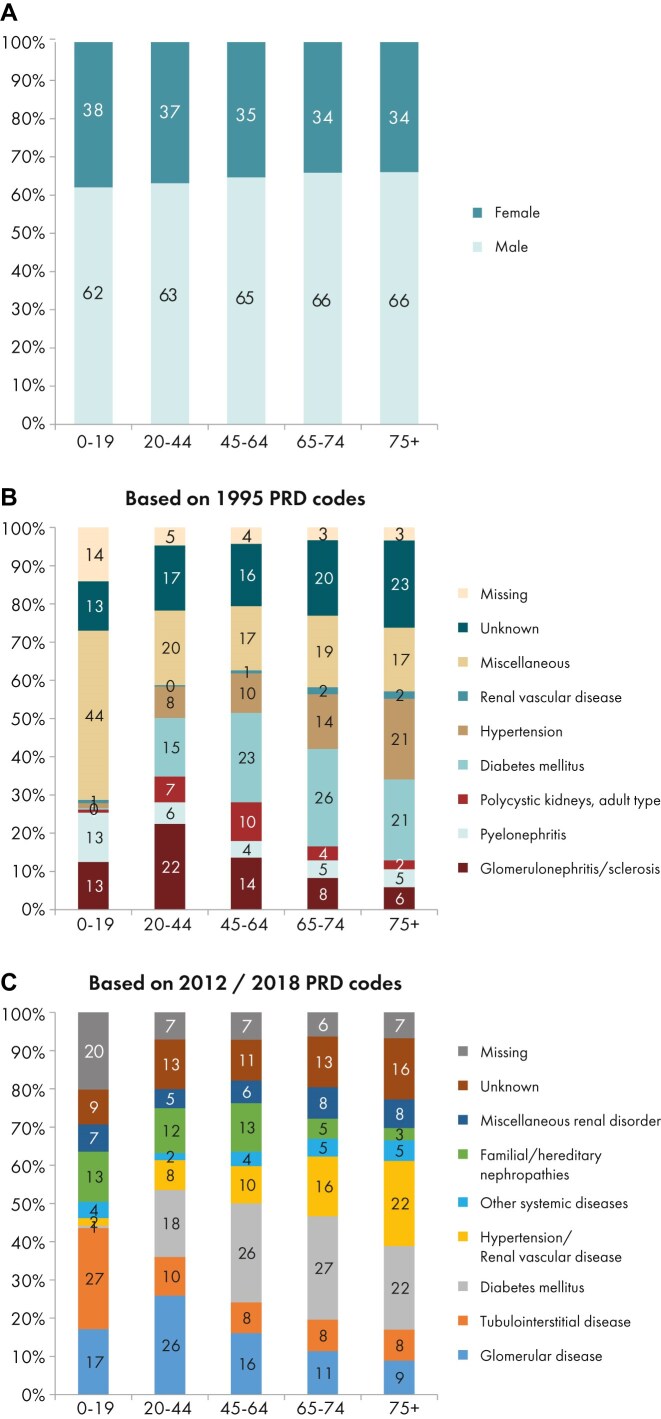
Distribution of (a) sex, (b) PRD (1995 ERA codes), and (c) PRD (2012/2018 ERA codes) by age for incident patients accepted for KRT in 2023 on day 1, unadjusted. This figure is only based on data from registries providing individual patient data (see Appendix 1). Bars may not add up to 100% due to rounding.

In this year’s annual report, additional comparisons by age are presented. In 2023, the KRT incidence increased with advancing age, ranging from 9 per million age-related population (pmarp) among individuals aged 0 to 19 years (1 in 111 100 inhabitants) to 500 pmarp among individuals aged ≥75 years (1 in 2000 inhabitants, Fig. 16). There was considerable variation between countries in the proportion of incident KRT patients aged ≥75 years, being nearly five times higher in Croatia (51%) than in Belarus (11%, Fig. 17). Among incident patients, the proportion of males increased slightly with advancing age, from 62% in patients aged 0 to 19 years to 66% in patients aged ≥75 years (Fig. 18). As expected, the distribution of PRD varied notably across age groups (Fig. 18). According to the 1995 ERA PRD codes, patients aged 0–19 years had the highest proportion of miscellaneous causes (44%), which included most of the inherited and congenital PRDs [5]. Hypertension as PRD increased steadily with age, from 1% in 0–19 year old patients reaching 21% in patients aged ≥75 years. Similarly, the proportion of patients with diabetes mellitus as PRD increased with age, peaking in patients aged 65–74 years (26%, Fig. 18). Findings using the updated ERA PRD codes from 2012–2018 for a limited set of countries, showed that patients aged 0–19 years had the highest proportion of tubulointerstitial disease (27%) and familial/hereditary nephropathies (13%), which both declined with advancing age. Glomerular disease as PRD was most frequent among patients aged 20 to 44 years (26%) and decreased with advancing age (Fig. 18). Furthermore, the distribution of initial KRT modalities varied across age groups, patients receiving pre-emptive kidney transplantation or PD decreased with age (Tx, 0–19 years 27%; ≥75 years 1%; and PD, 0–19 years 27%; ≥75 years 11%), whereas the proportion starting KRT with HD increased with age (HD: 0–19 years 45%; ≥75 years 88%, Fig. 4).

#### Prevalence

**Figure 19:**
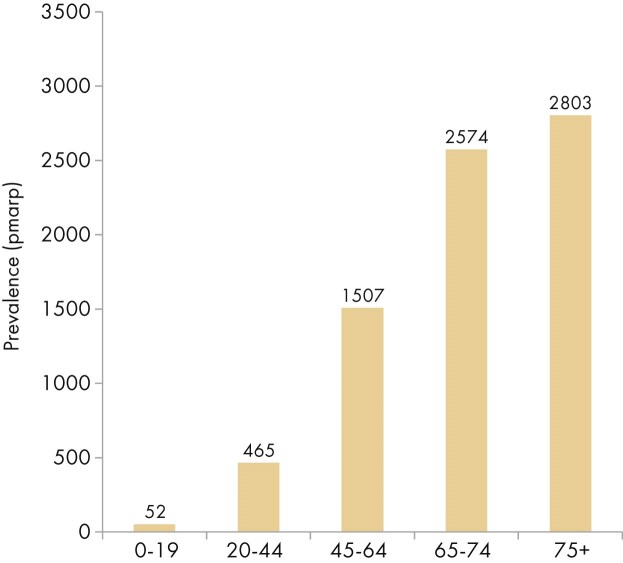
Prevalence of KRT per million age-related population (pmarp) on 31 December 2023 by age, unadjusted.

**Figure 20:**
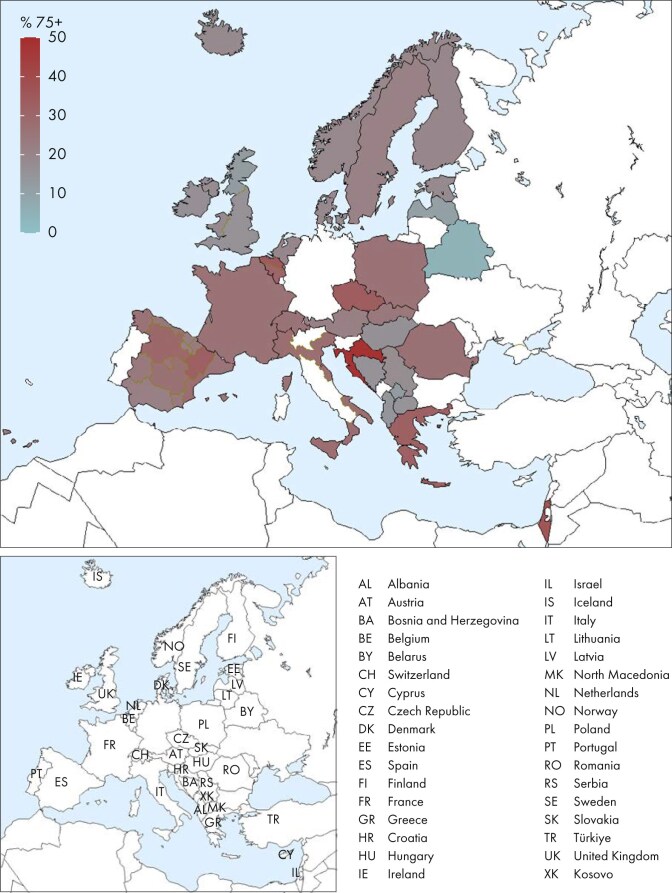
Percentage of prevalent patients aged ≥75 years among those on KRT on 31 December 2023, unadjusted.

**Figure 21:**
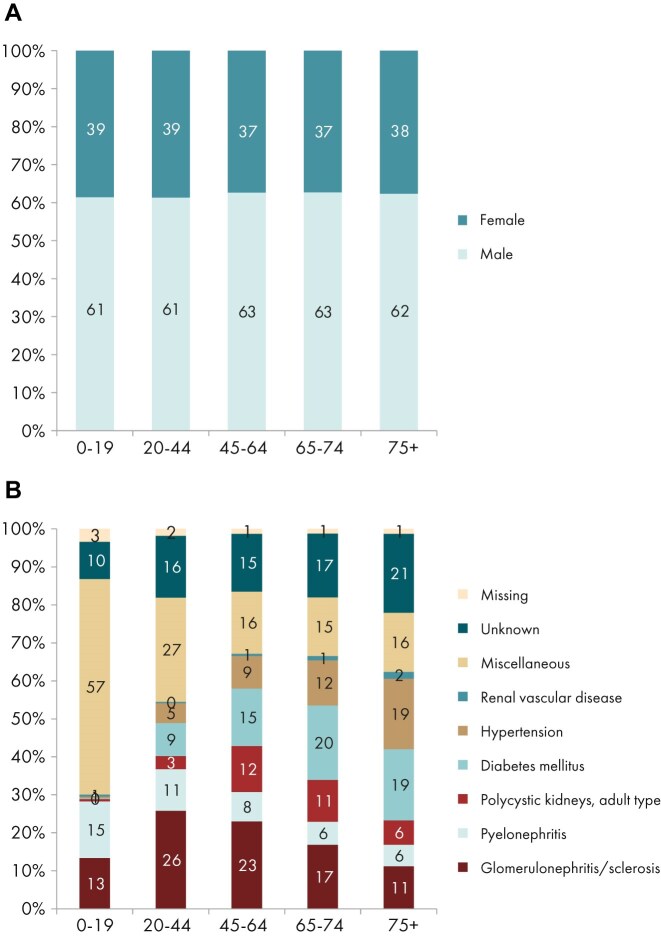
Distribution of (a) sex, and (b) PRD (1995 ERA codes) by age for prevalent patients on KRT on 31 December 2023, unadjusted. This figure is only based on data from registries providing individual patient data (see Appendix 1). Bars may not add up to 100% due to rounding.

The 2023 KRT prevalence also increased with advancing age, from 52 pmarp (1 in 19 200 inhabitants) among individuals aged 0 to 19 years to 2803 pmarp (1 in 360 inhabitants) among individuals aged ≥75 years (Fig. 19). The proportion of prevalent KRT patients aged ≥75 years varied substantially across countries, ranging from only 5% in Belarus to 48% in Croatia (Fig. 20). The sex distribution was similar across all age groups, with ∼62% of patients being male (Fig. 21). However, the distribution of PRDs varied across age groups, with decreasing proportions of patients with miscellaneous causes, pyelonephritis, and glomerulonephritis, and increasing proportions of patients with hypertension, diabetes mellitus, and unknown causes of kidney failure as age increased (Fig. 21). Furthermore, the distribution of treatment modalities varied across age groups. While there was a substantial increase in HD and a decrease in kidney transplantation with advancing age, the proportion of prevalent patients on PD remained relatively small across all age groups (Fig. 9).

#### Kidney transplantation

**Figure 22:**
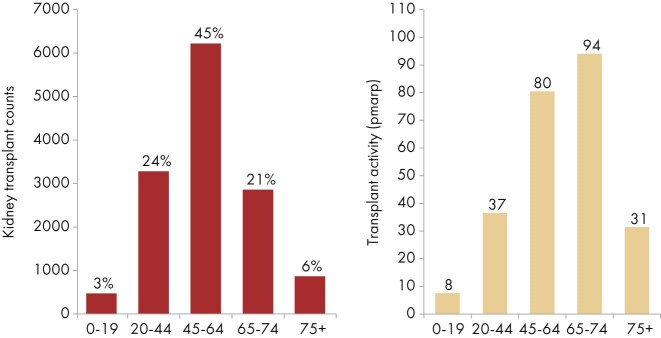
Kidney transplants counts and percentages (left panel) and transplant activity per million age-related population (pmarp; right panel) by age, unadjusted. This figure is only based on data from registries providing individual patient data (see Appendix 1).

**Figure 23:**
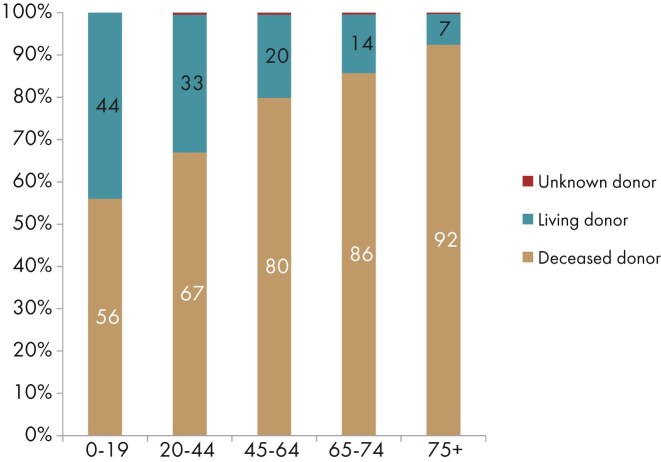
Donor type distribution by age in kidney transplant recipients. Bars may not add up to 100% due to rounding.

In 2023, almost half of all kidney transplantations were performed in patients aged 45 to 64 years (45%), while the youngest (0–19 years, 3%) and oldest (≥75 years, 6%) groups were only small fractions of the total (Fig. 22). Although the highest absolute number of transplantations was performed in patients aged 45 to 64 years, transplant activity (pmarp) was highest among patients aged 65 to 74 years (94 pmarp, Fig. 22). Nonetheless, transplant activity in patients aged 45–64 years was still relatively high (80 pmarp, Fig. 22). The distribution of donor type also varied by age, with the highest proportion of LD transplants among patients aged 0–19 years (44%), whereas patients aged ≥75 years received mostly kidneys from DD (92%, Fig. 23).

#### Survival

**Figure 24:**
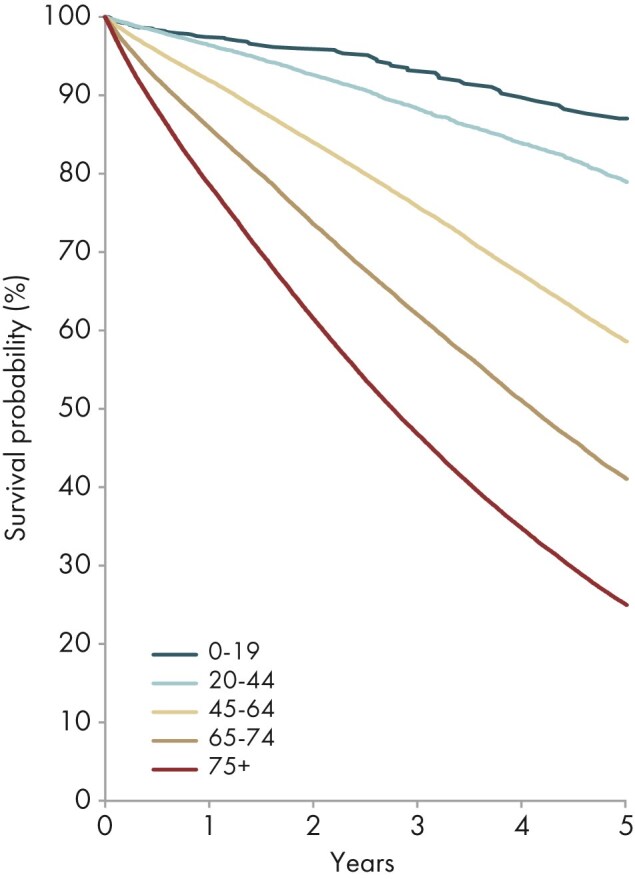
Patient survival in incident dialysis patients by age from day 91 (cohort 2014–2018), unadjusted. See Appendix 2 for a list of countries and regions providing individual patient data included in the survival analyses.

**Figure 25:**
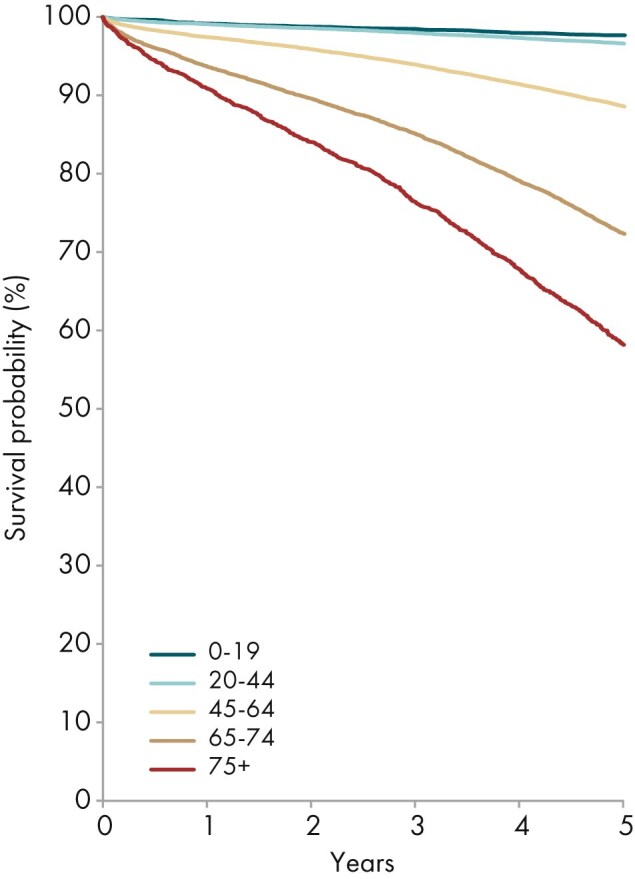
Patient survival in first-time kidney transplant recipients by age from day of transplant (cohort 2014–2018), unadjusted. See Appendix 2 for a list of countries and regions providing individual patient data included in the survival analyses.

The 5-year survival probability for dialysis patients ranged from 87% in patients aged 0 to 19 years to 25% in patients aged ≥75 years (Fig. 24). For patients receiving a first kidney transplant, the 5-year patient survival ranged from 98% in patients aged 0 to 19 years to 58% in patients aged ≥75 years (Fig. 25).

## AFFILIATED REGISTRIES

Albanian Renal Registry (E. Bolleke Likaj, A. Strakosha, and A. Idrizi); Austrian Dialysis and Transplant Registry (OEDTR) (G. Mayer, J. Kerschbaum and D. Kaiser-Feistmantl); Belarus Renal Registry (K. Kamisarau); Dutch-speaking Belgian Society of Nephrology (NBVN) (V. De Meyer and J. De Meester); French-speaking Belgian Society of Nephrology (GNFB) (J.M. des Grottes); Renal Registry Bosnia and Herzegovina (D. Rebic, N. Petkovic and M. Tomic); Croatian Renal Registry (D. Katicic and K. Altabas); Cyprus Renal Registry (I. Gregoriou and M. Athanasiadou); Czech Republic: Registry of Dialysis Patients (RDP) (I. Rychlík, M. Myslivecek and J. Potucek); Danish Nephrology Registry (DNS); Estonian Society of Nephrology (Ü. Pechter and J. Piel); Finnish Registry for Kidney Diseases (J. Helve and P. Finne); France: The Epidemiology and Information Network in Nephrology (REIN) (C. Couchoud); Hellenic Renal Registry (G. Moustakas); Hungarian Renal Registry (C. Ambrus, L. Wagner, and E. Ladanyi); Icelandic End-Stage Renal Disease Registry (R. Palsson); Ireland National Renal Office (H.T. Tee, M. Ganesan and J. Chevarria), Israel National Registry of Renal Replacement Therapy (L. Keinan-Boker and R. Dichtiar); Italian Registry of Dialysis and Transplantation (RIDT) (M. Nordio and P.M. Ferraro); Kosovo Renal Registry (M. Tolaj Avdiu and V. Godanci Kelmendi); Latvian Renal Registry (K. Racenis and J. Eke); Lithuanian Renal Registry (E. Žiginskiené, I. Nedzelskiene, and R. Gaidelyte); Dutch Renal Registry (Nefrodata) (P. Verschoor and L. Heuveling); North Macedonian Renal Registry (E. Babalj Bankoslieva and N. Misovska); Norwegian Renal Registry (A.V. Reisæter); Renal Registry of Poland (A. Debska-Slizien and P. Jagodzinski); Portuguese Renal Registry (E. Almeida); Romanian Renal Registry (RRR) (G. Mircescu, L. Garneata, and E. Podgoreanu); Renal Registry in Serbia (M. Lausevic, M. Kravljaca and all dialysis units in Serbia); Slovakian Renal Registry (I. Lajdová and J. Rosenberger); Spain Renal Registry (B. Mahillo Durán); Swedish Renal Registry (SRR) (K.G. Prütz, M. Evans, T. Lundgren, H. Rydell, and M. Segelmark); Swiss Dialysis Registry (P. Ambühl); Registry of the Nephrology, Dialysis and Transplantation in Türkiye (TSNNR) (I. Koçyigit and K. Ateş); UK Renal Registry (All the staff of the UK Renal Registry and of the renal units submitting data); Scottish Renal Registry (SRR) (All of the Scottish renal units); and the regional registries of Andalusia (SICATA) (P. Castro de la Nuez (on behalf of all users of SICATA)), Aragon (F. Arribas Monzón), Basque country (UNIPAR) (Á. Magaz, E. Corral, M. Rodrigo and I. Moina), Canary Islands (C. García Cantón and D. Marrero Miranda), Cantabria (J.C. Ruiz San Millán and M.O. Valentín Muños), Castile and León (M.E. Perea Rodríguez, M.A. Prieto Velasco and H. García López), Castile-La Mancha (G. Gutiérrez Ávila), Catalonia (RMRC) (J. Tort and M. Vázquez), Community of Madrid (A. Escribá Bárcenas and M. Marqués Vidas), Extremadura [all renal units (Nephrology and Dialysis) from Extremadura], Galicia (E. Bouzas-Caamaño and T. García Falcón), La Rioja (E. Huarte Loza and H. Hernández Vargas), Murcia (C. Santiuste de Pablos), Navarre (J. Manrique Escola), and the Valencian region (O.L. Rodríguez-Arévalo and A. Sarrión).

## ERA REGISTRY COMMITTEE MEMBERS

R. Torra, Spain (ERA President); A. Ortiz, Spain (Chair); M. Arnol, Slovenia; A. Åsberg, Norway; S. Bakkaloglu, Türkiye; P.M. Ferraro, Italy; J. Helve, Finland; J. Hogan, France; V. Kuzema, Latvia; B. Ponte, Switzerland; J.E. Sánchez-Álvarez, Spain; and M. Segelmark, Sweden.

## ERA REGISTRY OFFICE STAFF

V.S. Stel (Managing Director), M.E. Astley, R. Boenink, B.A. Boerstra, M. Bonthuis, N.C. Chesnaye, R. Cornet, M.W.F. Hoekstra, A. Kramer, A.C.L. Liem, I.R. Montez de Sousa (ESPN/ERA Registry staff), and A.J. Weerstra.

## Supplementary Material

sfag036_Supplemental_File

## Data Availability

The data underlying this article have been published in the ERA Registry Annual Report 2023 (Supplementary information).

## APPENDIX 1

### Countries or regions providing individual patient data to the ERA Registry

Austria, Belgium (Dutch-speaking), Belgium (French-speaking), Bosnia and Herzegovina, Denmark, Estonia, France, Greece, Iceland, Netherlands, Norway, Romania, Serbia, Spain (Andalusia), Spain (Aragon), Spain (Basque country), Spain (Canary Islands), Spain (Cantabria), Spain (Castile and León), Spain (Castile-La Mancha), Spain (Catalonia), Spain (Community of Madrid), Spain (Extremadura), Spain (Galicia), Spain (La Rioja), Spain (Murcia), Spain (Navarre), Spain (Valencian region), Sweden, Switzerland, UK (England, Northern Ireland, and Wales), and the UK (Scotland).

### Countries or regions providing aggregated data to the ERA Registry

Albania, Belarus, Croatia, Cyprus, Czech Republic, Finland, Hungary, Ireland, Israel, Italy, Kosovo, Latvia, Lithuania, North Macedonia, Poland, Portugal, Slovakia, Spain, and Türkiye.

### Countries part of the European Union (EU27) population as of 1 February 2020 (used as a reference population)

Austria, Belgium, Bulgaria, Croatia, Cyprus, Czech Republic, Denmark, Estonia, Finland, France, Germany, Greece, Hungary, Ireland, Italy, Latvia, Lithuania, Luxembourg, Malta, Netherlands, Poland, Portugal, Romania, Slovakia, Slovenia, Spain, and Sweden.

## APPENDIX 2

### Countries or regions included in the survival/expected remaining years of life analyses

Austria, Belgium (Dutch-speaking), Belgium (French-speaking), Bosnia and Herzegovina, Denmark, Estonia, France, Greece, Iceland, Netherlands, Norway, Spain (Andalusia), Spain (Aragon), Spain (Basque country), Spain (Canary Islands), Spain (Cantabria), Spain (Castille and León), Spain (Castille-La Mancha), Spain (Catalonia), Spain (Community of Madrid), Spain (Extremadura), Spain (Galicia), Spain (Murcia), Spain (Navarre), Spain (Valencian Region), Sweden, UK (England, Northern Ireland, and Wales), and UK (Scotland).

## References

[1] Boenink R, Bonthuis M, Boerstra BA et al. The ERA Registry Annual Report 2022: Epidemiology of Kidney Replacement Therapy in Europe, with a focus on sex comparisons. Clin Kidney J. 2024;18:sfae405. 10.1093/ckj/sfae405 40008269 PMC11852260

[2] Eurostat . https://ec.europa.eu/eurostat/data/database (February 2025, date last accessed).

[3] Boerstra BA, Boenink R, Astley ME et al. The ERA Registry Annual Report 2021: a summary. Clin Kidney J. 2023;17:sfad281. 10.1093/ckj/sfad281 38638342 PMC11024806

[4] Astley ME, Boenink R, Abd ElHafeez S et al. The ERA registry annual report 2020: a summary. Clin Kidney J. 2023;16:1330–54. 10.1093/ckj/sfad087 37529647 PMC10387405

[5] Ortiz A, Kramer A, Ariceta G et al. Inherited kidney disease and CAKUT are common causes of kidney failure requiring kidney replacement therapy: an ERA Registry study. Nephrol Dial Transplant. 2025;40:1020–31. 10.1093/ndt/gfae240 39508350 PMC12035533

