# Supplementary material for: The ERA Registry Annual Report 2023: epidemiology of kidney replacement therapy in Europe, with a focus on age comparisons
Source: Clin Kidney J. 2026 Feb 11;19(5):sfag036. doi: 10.1093/ckj/sfag036 (PMC13146735; doi:10.1093/ckj/sfag036)
Supplement: sfag036_Supplemental_File [file sfag036_supplemental_file.pdf]

REGISTRY

ANNUAL REPORT

2023

ERA
LEADING EUROPEAN NEPHROLOGY

Suggested Citation

Once the ERA Registry annual report 2023 is published as a supplement in the article "The ERA Registry Annual Report 2023: summary and age comparisons." in Clinical Kidney Journal (CKJ), the suggested citation will be the reference to this article in CKJ.
Until then the suggested citation is:
ERA Registry: ERA Registry Annual Report 2023. Amsterdam UMC, location AMC, Department of Medical Informatics, Amsterdam, Netherlands, 2025.

Disclamer

ERA, its Registry Committee, Amsterdam UMC; location AMC, and its department of Medical Informatics disclaim any express or implied warranty of fitness for the use of ERA Registry products and shall not be liable for any direct, indirect or consequential loss; personal injury; special or punitive damages; loss of profi ts, loss of savings and loss of revenue; loss of business, loss of reputation and loss of goodwill; and loss of data arising from the use of ERA Registry products.

ERA Registry

Amsterdam UMC, location AMC
Department of Medical Informatics
PO Box 22700
1100 DE Amsterdam
Netherlands

E-mail erareg@amsterdamumc.nl

Website www.era-online.org/research-education/era-registry

ERA Registry Committee

R Torra, Spain (ERA President)

A Ortiz, Spain (Chair)

M Arnol, Slovenia
A Åsberg, Norway
S Bakkaloglu, Türkiye
PM Ferraro, Italy
J Helve, Finland
J Hogan, France
V Kuzema, Latvia
B Ponte, Switzerland
JE Sánchez-Álvarez, Spain
M Segelmark, Sweden

ERA Registry Office Staff

VS Stel, epidemiologist (Director)

ME Astley, PhD student
R Boenink, epidemiologist
BA Boerstra, PhD student
M Bonthuis, epidemiologist
NC Chesnaye, epidemiologist
R Cornet, medical informatician (advisor)
MWF Hoekstra, PhD student
A Kramer, medical informatician
IR Montez de Sousa, PhD student (for paediatric section)
AJ Weerstra, software engineer

FIND

Acknowledgements

The ERA Registry would like to thank the patients and staff of all the dialysis and transplant units who have contributed data via their national and regional renal registries. In addition, we would like to thank the following persons and organizations for their contribution to the work of the ERA Registry.

For the provision of individual patient data

Registry	Contributors
Austria	G Mayer, J Kerschbaum, L Buchwinkler, and D Kaiser-Feistmantl
Belgium, Dutch-speaking	L Heylen, V De Meyer, and J De Meester
Belgium, French-speaking	JM des Grottes and L Weekers
Bosnia and Herzegovina	H Resic, D Rebic, N Petkovic, and M Tomic
Denmark	K Hommel
Estonia	Ü Pechter, M Ots-Rosenberg, and J Piel
France	C Couchoud and M Lassalle
Greece	G Moustakas
Iceland	OS Indriðason and R Pálsson
The Netherlands	P Verschoor, L Heuveling, and M ten Dam
Norway	AV Reisæter and A Åsberg
Romania	G Mircescu, L Garneata, and E Podgoreanu
Serbia	M Lausevic, M Kravljaca, and all dialysis units in Serbia
Spain, Andalusia	P Castro de la Nuez (on behalf of all users of SICATA)
Spain, Aragon	F Arribas Monzón
Spain, Basque country	Á Magaz, E Corral, M Rodrigo, and I Moina
Spain, Canary Islands	S Trujillo Alemán, C García Cantón, and D Marrero Miranda
Spain, Cantabria	JC Ruiz San Millán and MO Valentín Muñoz
Spain, Castile and León	ME Perea Rodríguez, MA Prieto Velasco, S Hernández Ramírez, and H García López
Spain, Castile-La Mancha	G Gutiérrez Ávila and I Moreno Alía
Spain, Catalonia	J Tort, J Comas, and M Vázquez
Spain, Community of Madrid	A Escribá Bárcenas and M Marqués Vidas
Spain, Extremadura	All the renal units (Nephrology and Dialysis) from Extremadura
Spain, Galicia	E Bouzas Caamaño and T García Falcón
Spain, La Rioja	E Huarte Loza, M Artamendi Larrañaga, and H Hernández Vargas
Spain, Murcia	I Marín Sánchez and C Santiuste de Pablos
Spain, Navarre	MF Slon Roblero and J Manrique Escola
Spain, Valencian region	OL Rodríguez-Arévalo and A Sarrión
Sweden	KG Prütz, M Stendahl, M Evans, T Lundgren, H Rydell, and M Segelmark
Switzerland	P Ambühl and R Guidotti
United Kingdom, England/Northern Ireland/Wales	All the staff of the UK Renal Registry and of the renal units submitting data
United Kingdom, Scotland	All of the Scottish renal units

For the provision of aggregated data

Registry	Contributors
Albania	E Bolleke Likaj, A Strakosha, and A Idrizi
Belarus	K Komissarov and K Kamisarau
Croatia	D Katicic and K Altabas
Cyprus	N Mistides, I Gregoriou, and M Athanasiadou
Czech Republic	I Rychlík, M Myslivecek, and J Potucek
Finland	J Helve and P Finne
Hungary	C Ambrus, L Wagner, and E Ladanyi
Ireland	HT Tee, M Ganesan, and J Chevarria
Israel	L Keinan-Boker, R Dichtiar, and P Beckerman
Italy (10 of 20 regions)	M Nordio and PM Ferraro
Italy (5 extra regions)	M Nordio and PM Ferraro
Kosovo	M Tolaj Avdiu, V Godanci Kelmendi, and F Smaili
Latvia	V Kuzema, K Racenis, and J Eke
Lithuania	E Ziginskiene, I Nedzelskiene, and R Gaidelyte
North Macedonia	N Gjorgjievski, E Babalj Bankoslieva, and N Misovska
Poland	A Debska-Slizien, P Jagodzinski, and R Gellert
Portugal	A Galvão and E Almeida
Slovakia	I Lajdová, A Okša, and J Rosenberger
Spain	B Mahillo Durán and Spanish Regional Registries
Türkiye	N Seyahi, I Koçyigit, and K Ateş

The ERA Registry is funded by the European Renal Association (ERA).

FIND

List of abbreviations

Abbreviation	Term
APD	Automated peritoneal dialysis
CAKUT	Congenital anomalies of the kidney and urinary tract
CAPD	Continuous ambulatory peritoneal dialysis
CI	Confidence interval
DM	Diabetes mellitus
ERA	European Renal Association
ESPN	European Society for Paediatric Nephrology
ESRD	End-stage renal disease
GN	Glomerulonephritis / sclerosis
HD	Haemodialysis
HDF	Haemodiafiltration
HF	Haemofiltration
HT	Hypertension
HUS	Haemolytic uraemic syndrome
KRT	Kidney replacement therapy
Misc	Miscellaneous
N	Number
P25	Refers to the 25th percentile
P75	Refers to the 75th percentile
PD	Peritoneal dialysis
PKD	Polycystic kidneys, adult type
Pmarp	Per million age-related population
Pmp	Per million population
PN	Pyelonephritis
PRD	Primary renal disease
RVD	Renal vascular disease
SD	Standard deviation
Tx	Transplant
Un	Unknown
Unkn	Unknown

Glossary

EU27

Refers to the 27 countries of which on 1 February 2020 the European Union was comprised of. These countries are: Austria, Belgium, Bulgaria, Croatia, Cyprus, Czech Republic, Denmark, Estonia, Finland, France, Germany, Greece, Hungary, Ireland, Italy, Latvia, Lithuania, Luxembourg, Malta, Netherlands, Poland, Portugal, Romania, Slovakia, Slovenia, Spain, and Sweden.

Eurostat

The statistical office of the European Union whose task is to provide the European Union with statistics at the European level that enable comparisons between countries and regions.

Expected remaining lifetime

Is defined as the average number of years of life remaining for those who have reached a given age.

Incidence

The number of new cases during a specific time period. In this annual report this equates to the number of patients commencing kidney replacement therapy for end-stage renal disease during the calendar year on which this annual report is based.

Kidney replacement therapy (KRT)

Therapy used to replace the normal blood-filtering function of the kidneys. In this annual report kidney replacement therapy refers to the various dialysis modalities and to kidney transplantation (see Modality).

Modality

The method of kidney replacement therapy. Kidney replacement therapy is comprised of the following modalities: haemodialysis (HD), haemofiltration (HF), haemodiafiltration (HDF), automated peritoneal dialysis (APD), continuous ambulatory peritoneal dialysis (CAPD), and kidney transplantation (Tx). Kidney transplantation can be performed with a kidney from a living donor (LD) or from a deceased donor (DD).

Per million age-related population (pmarp)

The incidence or prevalence pmarp is the observed incident or prevalent count for a specific age group divided by the general population of that age group and multiplied by one million.

Per million population (pmp)

The incidence or prevalence pmp is the observed incident or prevalent count divided by the general population in that year and multiplied by one million.

Percentage coverage

Coverage of the general population in a country or region by the registry of that country or region.

Prevalence

The number of people in a given population with a particular disease at a given time. In this annual report this equates to the number of patients receiving kidney replacement therapy for end-stage renal disease on 31st December 2023.

Survival probability

The probability that a person or a kidney transplant has survived up to a specified time point.

Brief Content

Introduction 14

Section A Summary data and comparisons by age distribution 16

A1 INCIDENT PATIENTS ACCEPTED FOR KRT IN 2023, AT DAY 1 17
A2 INCIDENT PATIENTS ACCEPTED FOR KRT IN 2023, AT DAY 91 23
A3 PREVALENT PATIENTS ON KRT IN 2023 24
A4 KIDNEY TRANSPLANTS IN 2023 29
A5 SURVIVAL PROBABILITY 32
A6 EXPECTED REMAINING LIFETIMES 34
A7 COMPARISONS BY AGE FOR 2023 35

Section B Individual patient data reference tables 44

B1 AFFILIATED REGISTRY INFORMATION FOR 2023 45
B2 INCIDENT PATIENTS ACCEPTED FOR KRT IN 2023, AT DAY 1 46
B3 INCIDENT PATIENTS ACCEPTED FOR KRT IN 2023, AT DAY 91 59
B4 PREVALENT PATIENTS ON KRT IN 2023 72
B5 KIDNEY TRANSPLANTS IN 2023 83
B6 SURVIVAL PROBABILITY 86
B7 EXPECTED REMAINING LIFETIMES 102

Section C Aggregated data reference tables 103

C1 AFFILIATED REGISTRY INFORMATION FOR 2023 104
C2 INCIDENT PATIENTS ACCEPTED FOR KRT IN 2023, AT DAY 1 105
C3 INCIDENT PATIENTS ACCEPTED FOR KRT IN 2023, AT DAY 91 113
C4 PREVALENT PATIENTS ON KRT IN 2023 121
C5 KIDNEY TRANSPLANTS IN 2023 129

Section D Paediatric data reference tables 131

D1 GENERAL POPULATION AGE DISTRIBUTION 132
D2 INCIDENT PATIENTS ACCEPTED FOR KRT, AT DAY 1 133
D3 PREVALENT PATIENTS ON KRT 135

Methods 137

Appendices 148

Table of Contents

Introduction 14

Section overview 15
New to this edition 15
ERA Registry website 15
ESPN/ERA Registry website 15

Section A Summary data and comparisons by age distribution 16

A1 INCIDENT PATIENTS ACCEPTED FOR KRT IN 2023, AT DAY 1

Table A.1.1 Summary data on the incidence of KRT in 2023, unadjusted 17
Figure A.1.1 Incidence per million population by country / region, unadjusted 18
Figure A.1.2 Incidence per million population by country / region, unadjusted and adjusted 19
Figure A.1.3 Age, sex, and primary renal disease distribution by type of data provided,unadjusted 20
Figure A.1.4 Treatment modality distribution by type of data provided, age, sex and primary renal disease, unadjusted 22

A2 INCIDENT PATIENTS ACCEPTED FOR KRT IN 2023, AT DAY 91

Figure A.2.1 Treatment modality distribution by type of data provided, age, sex and primary renal disease, unadjusted 21 23

A3 PREVALENT PATIENTS ON KRT IN 2023

Table A.3.1 Summary data on the prevalence of KRT on 31 December 2023, unadjusted 24
Figure A.3.1 Prevalence per million population by country / region, unadjusted 25
Figure A.3.2 Prevalence per million population by country / region, unadjusted and adjusted 26
Figure A.3.3 Age, sex, and primary renal disease distribution by type of data provided, unadjusted 27
Figure A.3.4 Treatment modality distribution by type of data provided, age, sex and primary renal disease, unadjusted 26 28

A4 KIDNEY TRANSPLANTS IN 2023

Figure A.4.1 Kidney transplant counts and per million population by country / region, unadjusted 29
Figure A.4.2 Kidney transplants per million population by donor type and by country /region, unadjusted 30
Figure A.4.3 Donor type distribution by type of data provided, unadjusted 31

A5 SURVIVAL PROBABILITY

Table A.5.1 One-, two- and five-year survival probabilities by treatment modality and cohort 32
Figure A.5.1 Patient survival by modality: Incident dialysis patients (cohort 2014-2018) 33
Figure A.5.2 Patient survival by donor type: Patients receiving a first kidney transplant (cohort 2014-2018) 33

A6 EXPECTED REMAINING LIFETIMES

Figure A.6.1 Expected remaining years of life in the general population and in prevalent dialysis and kidney transplant patients (cohort 2019-2023) 34

A7 COMPARISONS BY AGE FOR 2023

Figure A.7.1 Age distribution by country / region, unadjusted 35
Figure A.7.2 Incidence per million age-related population by age, unadjusted 36
Figure A.7.3 Sex, primary renal disease and treatment modality distribution by age in incident patients, unadjusted 37
Figure A.7.4 Age distribution by country / region, unadjusted 39

Table of Contents

A7 COMPARISONS BY AGE FOR 2023

Figure A.7.5 Prevalence per million age-related population by age, unadjusted 40
Figure A.7.6 Sex, primary renal disease and treatment modality distribution by age in prevalent patients, unadjusted 39 41
Figure A.7.7 Kidney transplant counts and percentages by recipient age and transplant activity per million age-related population by age, unadjusted 42
Figure A.7.8 Donor type distribution by age in kidney transplant recipients, unadjusted 42
Figure A.7.9 Patient survival probability by age: Incident dialysis patients (cohort 2014-2018) 43
Figure A.7.10 Patient survival probability by age: Patients receiving a first kidney transplant (cohort 2014-2018) 43

Section B Individual patient data reference tables 44

B1 AFFILIATED REGISTRY INFORMATION FOR 2023

Table B.1.1 General population data and number of renal centres 45

B2 INCIDENT PATIENTS ACCEPTED FOR KRT IN 2023, AT DAY 1

Table B.2.1 Incident counts and percentages by age and sex 46
Table B.2.2 Incidence per million (age-related) population by age and sex, unadjusted 47
Table B.2.3 Sex, mean age, and median age of incident patients 48
Table B.2.4 Incidence per million population, adjusted 49
Table B.2.5.A Incidence per million population and percentages by primary renal disease (1995 PRD codes), unadjusted 50
Table B.2.5.B Incidence per million population and percentages by primary renal disease (2012/2018 PRD codes), unadjusted 51
Table B.2.6.A Incidence per million population by primary renal disease (1995 PRD codes), adjusted 52
Table B.2.6.B Incidence per million population by primary renal disease (2012/2018 PRD codes), adjusted 53
Table B.2.7 Incident counts by treatment modality 54
Table B.2.8 Incidence per million population by treatment modality, unadjusted 55
Table B.2.9 Incidence per million population by treatment modality, adjusted 56
Table B.2.10 Treatment modality distribution, unadjusted 57
Table B.2.11 Treatment modality distribution by age, sex, and primary renal disease, unadjusted 58

B3 INCIDENT PATIENTS ACCEPTED FOR KRT IN 2023, AT DAY 91

Table B.3.1 Incident counts and percentages by age and sex 59
Table B.3.2 Incidence per million (age-related) population by age and sex, unadjusted 60
Table B.3.3 Sex, mean age, and median age of incident patients 61
Table B.3.4 Incidence per million population, adjusted 62
Table B.3.5.A Incidence per million population and percentages by primary renal disease (1995 PRD codes), unadjusted 63
Table B.3.5.B Incidence per million population and percentages by primary renal disease (2012/2018 PRD codes), unadjusted 64
Table B.3.6.A Incidence per million population by primary renal disease (1995 PRD codes), adjusted 65
Table B.3.6.B Incidence per million population by primary renal disease (2012/2018 PRD codes), adjusted 66
Table B.3.7 Incident counts by treatment modality 67
Table B.3.8 Incidence per million population by treatment modality, unadjusted 68
Table B.3.9 Incidence per million population by treatment modality, adjusted 69
Table B.3.10 Treatment modality distribution, unadjusted 70
Table B.3.11 Treatment modality distribution by age, sex, and primary renal disease, unadjusted 71

Table of Contents

B4 PREVALENT PATIENTS ON KRT IN 2023

Table B.4.1	Prevalent counts and percentages by age and sex	72
Table B.4.2	Prevalence per million (age-related) population by age and sex, unadjusted	73
Table B.4.3	Sex, mean age, and median age	74
Table B.4.4	Prevalence per million population, adjusted	75
Table B.4.5	Prevalence per million population and percentages by primary renal disease (1995 PRD codes), unadjusted	76
Table B.4.6	Prevalence per million population by primary renal disease (1995 PRD codes), adjusted	77
Table B.4.7	Prevalent counts by treatment modality	78
Table B.4.8	Prevalence per million population by treatment modality, unadjusted	79
Table B.4.9	Prevalence per million population by treatment modality, adjusted	80
Table B.4.10	Treatment modality distribution, unadjusted	81
Table B.4.11	Treatment modality distribution by age, sex, and primary renal disease, unadjusted	82

B5 KIDNEY TRANSPLANTS IN 2023

Table B.5.1	Kidney transplant counts and percentages by donor type	83
Table B.5.2	Kidney transplants per million population by donor type, unadjusted	84
Table B.5.3	Sex, mean age, and median age of kidney transplant recipients	85

B6 SURVIVAL PROBABILITY

PATIENT SURVIVAL ON KRT (COHORT 2014-2018 AND COHORT 2017-2021)

Table B.6.1	Incident KRT patients, from day 1, unadjusted	86
Table B.6.2	Incident KRT patients, from day 1, adjusted	87
Table B.6.3	Incident KRT patients, from day 91, unadjusted	88
Table B.6.4	Incident KRT patients, from day 91, adjusted	89

PATIENT SURVIVAL ON DIALYSIS (COHORT 2014-2018 AND COHORT 2017-2021)

Table B.6.5	Incident dialysis patients, from day 1, unadjusted	90
Table B.6.6	Incident dialysis patients, from day 1, adjusted	91
Table B.6.7	Incident dialysis patients, from day 91, unadjusted	92
Table B.6.8	Incident dialysis patients, from day 91, adjusted	93

PATIENT SURVIVAL AFTER FIRST KIDNEY TRANSPLANT (COHORT 2014-2018 AND COHORT 2017-2021)

Table B.6.9	First transplant patients (deceased donor), from day of transplant, unadjusted	94
Table B.6.10	First transplant patients (deceased donor), from day of transplant, adjusted	95
Table B.6.11	First transplant patients (living donor), from day of transplant, unadjusted	96
Table B.6.12	First transplant patients (living donor), from day of transplant, adjusted	97

GRAFT SURVIVAL AFTER FIRST KIDNEY TRANSPLANT (COHORT 2014-2018 AND COHORT 2017-2021)

Table B.6.13	First graft (deceased donor), from day of transplant, unadjusted	98
Table B.6.14	First graft (deceased donor), from day of transplant, adjusted	99
Table B.6.15	First graft (living donor), from day of transplant, unadjusted	100
Table B.6.16	First graft (living donor), from day of transplant, adjusted	101

B7 EXPECTED REMAINING LIFETIMES

Table B.7.1	Expected remaining years of life in the general population and in prevalent dialysis and kidney transplant patients (cohort 2019-2023)	102

Table of Contents

Section C Aggregated data reference tables 103

C1 AFFILIATED REGISTRY INFORMATION FOR 2023

Table C.1.1 General population data and number of renal centres 104

C2 INCIDENT PATIENTS ACCEPTED FOR KRT IN 2023, AT DAY 1

Table C.2.1 Incident counts and percentages by age and sex 105
Table C.2.2 Incidence per million (age-related) population by age and sex, unadjusted 106
Table C.2.3 Sex, mean age, and median age of incident patients 107
Table C.2.4 Incidence per million population, adjusted 108
Table C.2.5 Incidence per million population and percentages by primary renal disease (1995 PRD codes), unadjusted 109
Table C.2.6 Incident counts by treatment modality 110
Table C.2.7 Incidence per million population by treatment modality, unadjusted 111
Table C.2.8 Treatment modality distribution, unadjusted 112

C3 INCIDENT PATIENTS ACCEPTED FOR KRT IN 2023, AT DAY 91

Table C.3.1 Incident counts and percentages by age and sex 113
Table C.3.2 Incidence per million (age-related) population by age and sex, unadjusted 114
Table C.3.3 Sex, mean age, and median age of incident patients 115
Table C.3.4 Incidence per million population, adjusted 116
Table C.3.5 Incidence per million population and percentages by primary renal disease (1995 PRD codes), unadjusted 117
Table C.3.6 Incident counts by treatment modality 118
Table C.3.7 Incidence per million population by treatment modality, unadjusted 119
Table C.3.8 Treatment modality distribution, unadjusted 120

C4 PREVALENT PATIENTS ON KRT IN 2023

Table C.4.1 Prevalent counts and percentages by age and sex 121
Table C.4.2 Prevalence per million (age-related) population by age and sex, unadjusted 122
Table C.4.3 Sex, mean age, and median age 123
Table C.4.4 Prevalence per million population, adjusted 124
Table C.4.5 Prevalence per million population and percentages by primary renal disease (1995 PRD codes), unadjusted 125
Table C.4.6 Prevalent counts by treatment modality 126
Table C.4.7 Prevalence per million population by treatment modality, unadjusted 127
Table C.4.8 Treatment modality distribution, unadjusted 128

C5 KIDNEY TRANSPLANTS IN 2023

Table C.5.1 Kidney transplant counts and percentages by donor type 129
Table C.5.2 Kidney transplants per million population by donor type, unadjusted 130

Table of Contents

Section D Paediatric data reference tables — 131

D1 GENERAL POPULATION AGE DISTRIBUTION

Table D.1.1 Population covered (in thousands) — 132

D2 INCIDENT PATIENTS ACCEPTED FOR KRT, AT DAY 1

Table D.2.1 Incident counts by age and cohort — 133
Table D.2.2 Incident counts by age, treatment modality, and cohort — 133
Table D.2.3 Incident counts by age and primary renal disease — 133
Table D.2.4 Incidence per million age-related population by age and cohort — 134
Table D.2.5 Incidence per million age-related population by age, treatment modality, and cohort — 134
Table D.2.6 Incidence per million age-related population by age and primary renal disease — 134

D3 PREVALENT PATIENTS ON KRT

Table D.3.1 Prevalent counts by age and cohort — 135
Table D.3.2 Prevalent counts by age, treatment modality, and cohort — 135
Table D.3.3 Prevalent counts by age and primary renal disease — 135
Table D.3.4 Prevalence per million age-related population by age and cohort — 136
Table D.3.5 Prevalence per million age-related population by age, treatment modality, and cohort — 136
Table D.3.6 Prevalence per million age-related population by age and primary renal disease — 136

Methods — 137

Data collection and preparation — 138
Coding systems — 141
Statistical analyses — 142
Bibliography — 147

Appendices — 148

Appendix 1 Grouping of primary renal disease codes 1995 — 149
Appendix 2 Grouping of primary renal disease codes 2018 — 150
Appendix 3 Grouping of causes of death — 155
Appendix 4 Event type codes — 156
Appendix 5 Renal registries contributing data for the different types of analyses — 157

FIND

Introduction

Introduction

The ERA Registry collects data on kidney replacement therapy (KRT) via the national and regional renal registries in Europe and countries bordering the Mediterranean Sea. For this 2023 Annual Report data sets from 51 national or regional renal registries in 34 countries were used. The reference tables are presented in two parts: Section B relates to individual patient data from 32 national and regional registries from 16 countries (shown in red in Figure 1), whereas Section C relates to 19 national and regional registries from 19 countries providing aggregated data (shown in orange in Figure 1).

Section overview

In Section A, figures summarizing the data from all participating registries are presented. In addition, in Section A of this 2023 Annual Report, figures on comparisons by age are included. Section B contains data on the incidence and prevalence of KRT and on the kidney transplant activity, the survival and expected remaining lifetimes from registries providing individual patient data. Section C covers data on the incidence and prevalence and kidney transplant activity from the countries and regions providing aggregated data. The incidence and prevalence of KRT in paediatric patients are described in Section D, and are based on data from registries providing individual paediatric patient data. The Methods section describes the procedures used, including data collection and preparation, definitions, coding systems, and statistical methods.

New to this edition

This year for the first time, Ireland was included with aggregated data.

ERA Registry website

The ERA Registry annual reports, the reference tables and the slides based on the information of these annual reports can be downloaded from the ERA website: www.era-online.org/research-education/era-registry. In addition, the website includes a repository of educational publications and a link to the 2018 version of the ERA Registry Primary Renal Disease Codes.

ESPN/ERA Registry website

Information on the activities of the European Society for Paediatric Nephrology (ESPN)/ERA Registry and on paediatric registries in general can be found on the ESPN/ERA Registry website: www.espn-reg.org. In addition, the ESPN/ERA Registry annual reports can be downloaded from this website.

Figure 1: National and regional renal registries that contributed data to this annual report

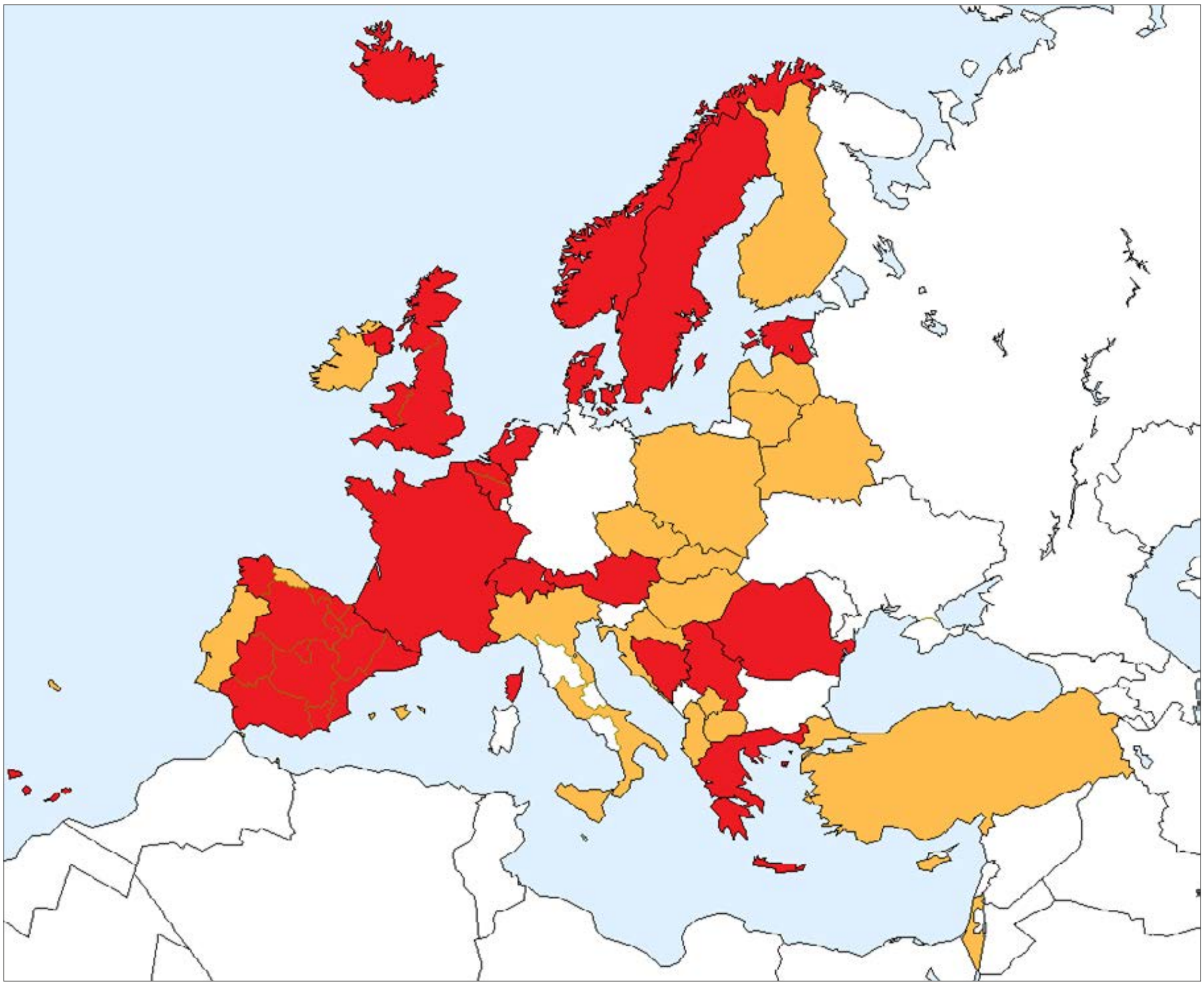

registries contributing individual patient data

registries contributing aggregated data

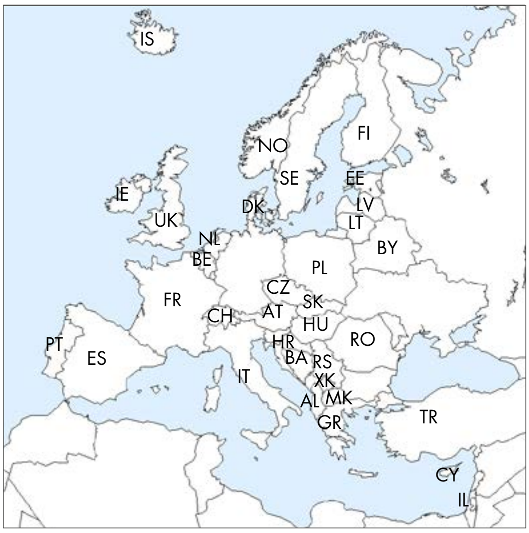

AL	Albania	IL	Israel
AT	Austria	IS	Iceland
BA	Bosnia and Herzegovina	IT	Italy
BE	Belgium	LT	Lithuania
BY	Belarus	LV	Latvia
CH	Switzerland	MK	North Macedonia
CY	Cyprus	NL	The Netherlands
CZ	Czech Republic	NO	Norway
DK	Denmark	PL	Poland
EE	Estonia	PT	Portugal
ES	Spain	RO	Romania
FI	Finland	RS	Serbia
FR	France	SE	Sweden
GR	Greece	SK	Slovakia
HR	Croatia	TR	Türkiye
HU	Hungary	UK	United Kingdom
IE	Ireland	XK	Kosovo

Section A

Summary data and comparisons by age

Table A.1.1

Summary data on the incidence of KRT in 2023, unadjusted

	General population covered by the registry in thousands	Incidence in 2023, at day 1					
		All N	All Pmp	Mean Age	Median Age	DM N	DM Pmp
Albania	2734	394	144	61.6	65.0	95	35
Austria §	8923	1032	116	64.5	68.2	200	22
Belarus | |	8444	883	105			198	23
Belgium, Dutch-speaking *	6813	1075	158	70.8	73.7	228	33
Belgium, French-speaking *	4967	932	188	68.3	70.7	178	36
Bosnia and Herzegovina	3531	424	120	65.1	68.3	112	32
Croatia †	3281	484	148	67.2	70.0	119	36
Cyprus	949	255	269	67.3	71.0	91	96
Czech Republic	10828	1744	161	66.0	69.0	363	34
Denmark	5947	722	121	64.5	67.8	187	31
Estonia	1302	84	65	63.5	64.4	15	12
Finland	5492	458	83	62.0	65.8	158	29
France	68372	10974	161	67.3	71.0	2467	36
Greece	10407	2602	250	70.8	73.3	604	58
Hungary	9600	1321	138	63.8	67.0	491	51
Iceland	386	50	130	64.8	65.8	6	16
Israel	9633	1807	188	66.2	69.5	697	72
Italy (10 of 20 regions)	28614	4860	170	67.4	71.3	557	19
Kosovo | |	1688	225	133	65.5	68.0	70	41
Latvia	1732	185	107	61.7	63.0	34	20
Lithuania	2857	368	129	63.6	65.2	60	21
Netherlands	16626	1958	118	62.8	66.6	421	25
North Macedonia	1826	423	232	65.3	67.0	109	60
Norway	5520	563	102	62.5	66.7	83	15
Poland †	37637	5655	150				
Portugal ¶	10640	2521	237			768	72
Romania	19061	3702	194	63.8	66.4	438	23
Serbia	6391	519	81	62.0	65.0	121	19
Slovakia †	4472	794	178	62.9	66.0	235	53
Spain (All)	48085	7287	152	63.5	68.3	1602	33
Spain, Andalusia	8608	1285	149	65.1	68.4	296	34
Spain, Aragon	1346	196	146	65.6	69.5	41	30
Spain, Basque country	2222	278	125	66.0	68.6	66	30
Spain, Canary Islands	2226	402	181	63.7	65.9	124	56
Spain, Cantabria*	590	100	170	70.0	73.7	18	31
Spain, Castile and León *	2384	347	146	67.7	69.5	84	35
Spain, Castile-La Mancha *	2094	260	124	65.9	67.8	66	32
Spain, Catalonia	7902	1455	184	66.8	70.4	338	43
Spain, Community of Madrid	6872	890	130	62.0	66.4	167	24
Spain, Extremadura	1054	167	158	68.1	70.4	45	43
Spain, Galicia	2703	423	157	67.0	70.0	98	36
Spain, La Rioja	323	42	130	61.5	62.2	10	31
Spain, Murcia	1552	227	146	64.1	67.4	52	34
Spain, Navarre *	675	90	133	64.6	66.8	13	19
Spain, Valencian region	5216	758	145	66.4	69.4	152	29
Sweden	10537	1142	108	64.7	68.4	283	27
Switzerland	8444	752	89	66.4	70.0	133	16
Türkiye ‡	85372	13641	160			2883	54
United Kingdom, England	53075	6599	124	60.2	62.7	1766	33
United Kingdom, Northern Ireland	1920	236	123	62.5	65.2	52	27
United Kingdom, Scotland	5490	603	110	60.4	63.1	180	33
United Kingdom, Wales	3164	464	147	62.6	65.3	131	41
All countries	514760	77738	151	65.0	68.4	16135	36

DM = diabetes mellitus as primary renal disease
When cells are left empty, the data are unavailable and could not be used for the calculation of the summary data
§ The incidence is underestimated by approximately 2% due to one haemodialysis centre not submitting data
| | Patients younger than 18 years of age are not reported
* Patients younger than 20 years of age are not reported
† Data include dialysis patients only
¶ Data on primary renal disease are available for dialysis patients only (N=2489, 98.7% of total)
‡ Data on DM are extrapolated from data of 8494 patients (62.3% of total)

Figure A.1.1

Incidence per million population by country / region, unadjusted

at day 1

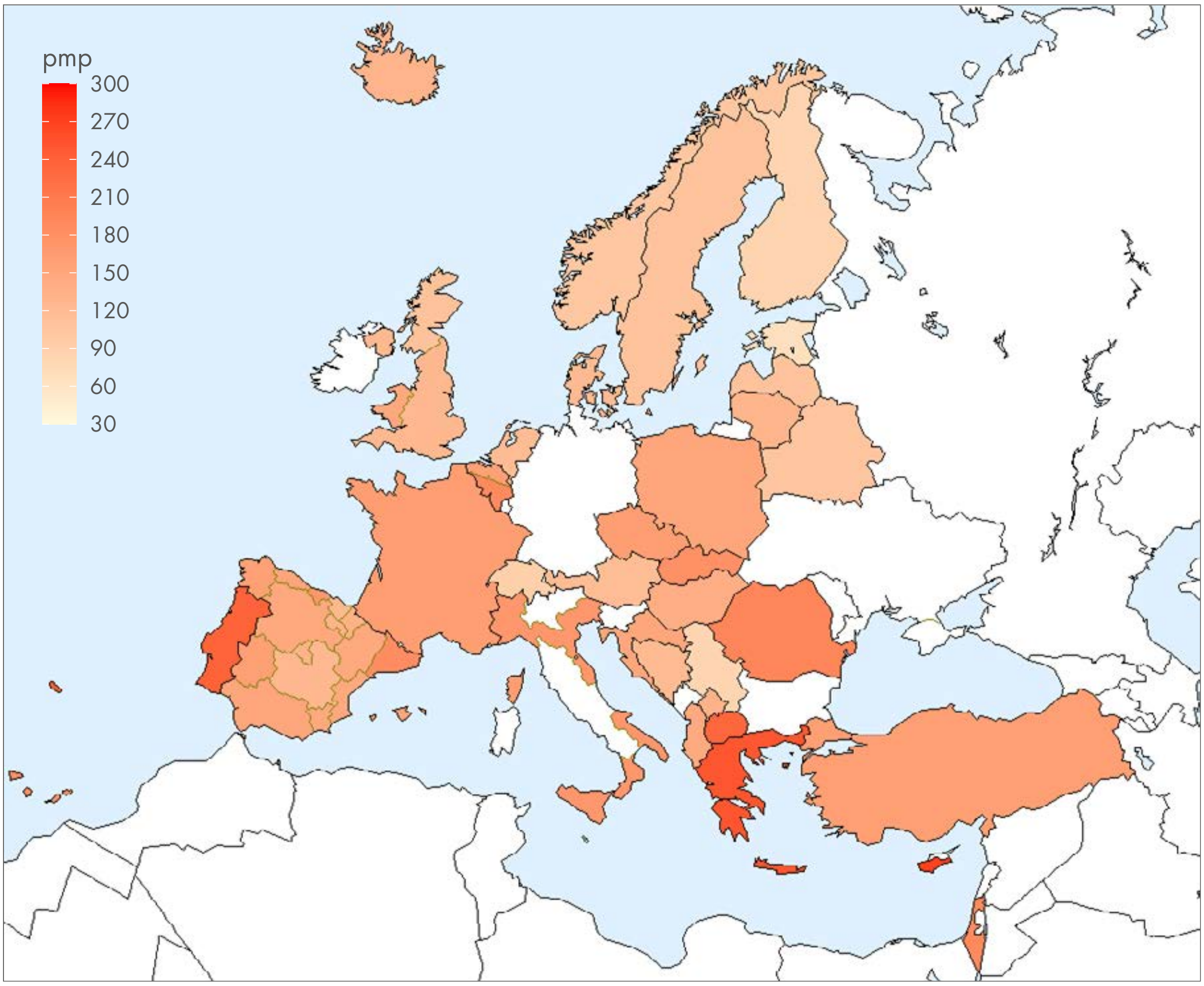

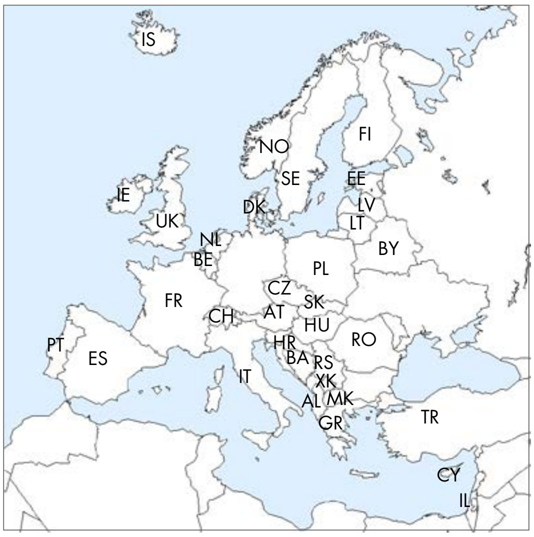

AL	Albania	IL	Israel
AT	Austria	IS	Iceland
BA	Bosnia and Herzegovina	IT	Italy
BE	Belgium	LT	Lithuania
BY	Belarus	LV	Latvia
CH	Switzerland	MK	North Macedonia
CY	Cyprus	NL	Netherlands
CZ	Czech Republic	NO	Norway
DK	Denmark	PL	Poland
EE	Estonia	PT	Portugal
ES	Spain	RO	Romania
FI	Finland	RS	Serbia
FR	France	SE	Sweden
GR	Greece	SK	Slovakia
HR	Croatia	TR	Türkiye
HU	Hungary	UK	United Kingdom
IE	Ireland	XK	Kosovo

Detailed information on the contents of this figure can be found in table B.2.2 for registries providing individual patient data, and in table C.2.2 for registries providing aggregated data.

Figure A.1.2

Incidence per million population by country / region, unadjusted and adjusted
at day 1

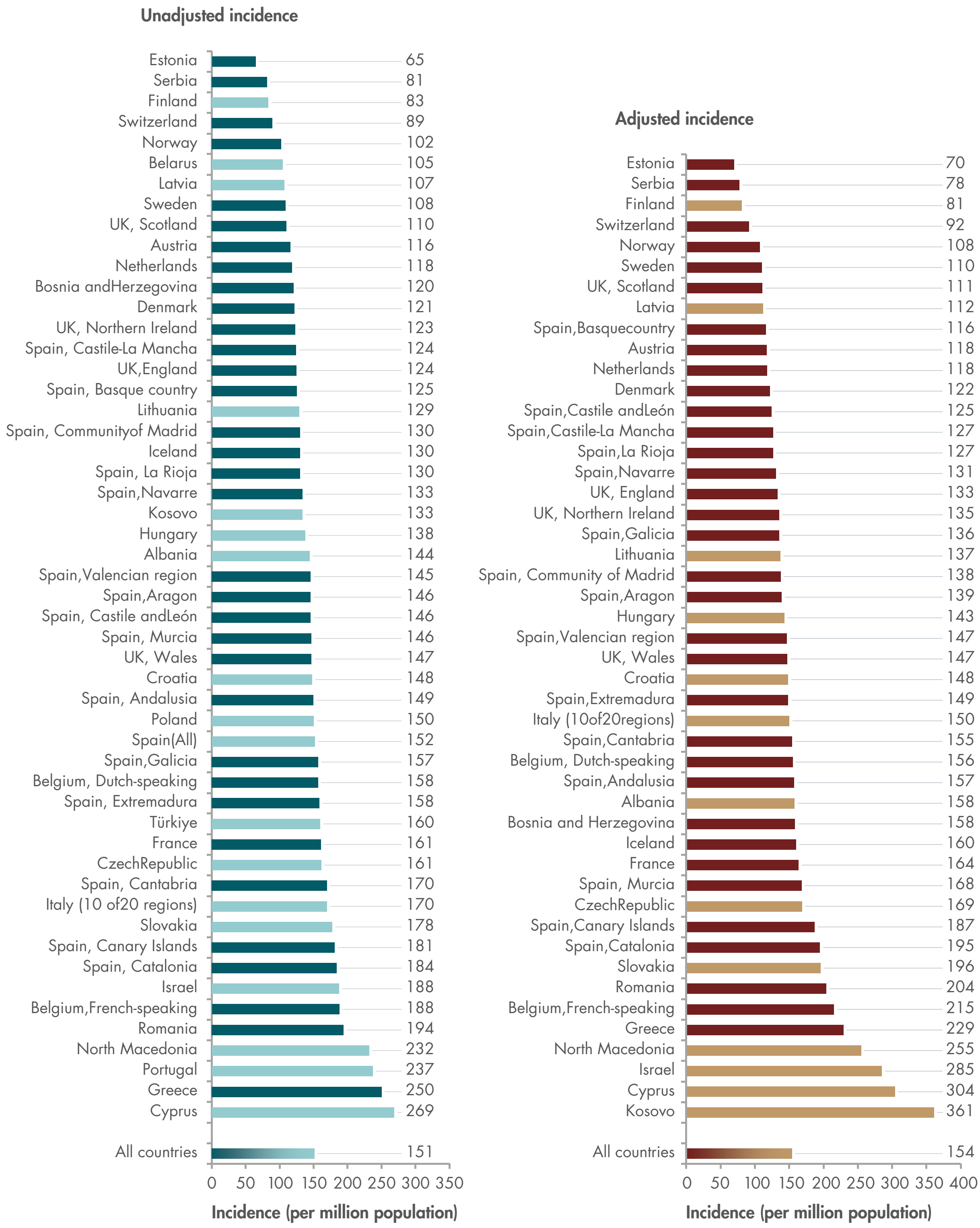

Registries providing individual patient data are shown as dark bars, and registries providing aggregated data as light bars.
Detailed information on the contents of this fi gure can be found in tables B.2.2 and B.2.4 for registries providing individual patient data, and in tables C.2.2 and C.2.4 for registries providing aggregated data. Adjustment of the incidence was performed by standardising the incidence to the age and sex distribution of the EU27 population.

FIND

Figure A.1.3

Age, sex, and primary renal disease distribution by type of data provided, unadjusted
at day 1

This figure summarizes the data presented in tables B.2.1 and C.2.1

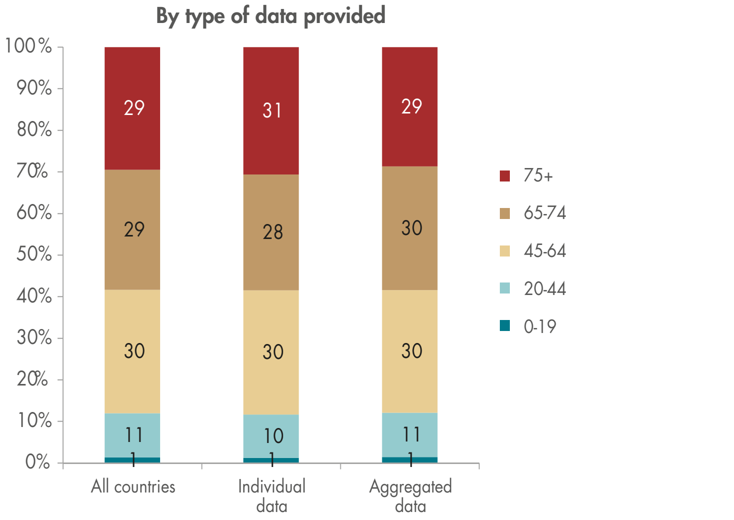

This figure summarizes the data presented in tables B.2.3 and C.2.3

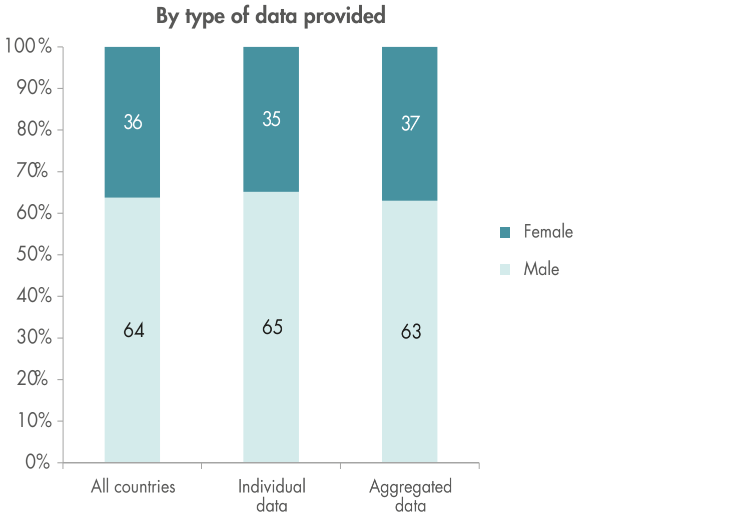

This figure summarizes the data presented in tables B.2.5.A and C.2.5

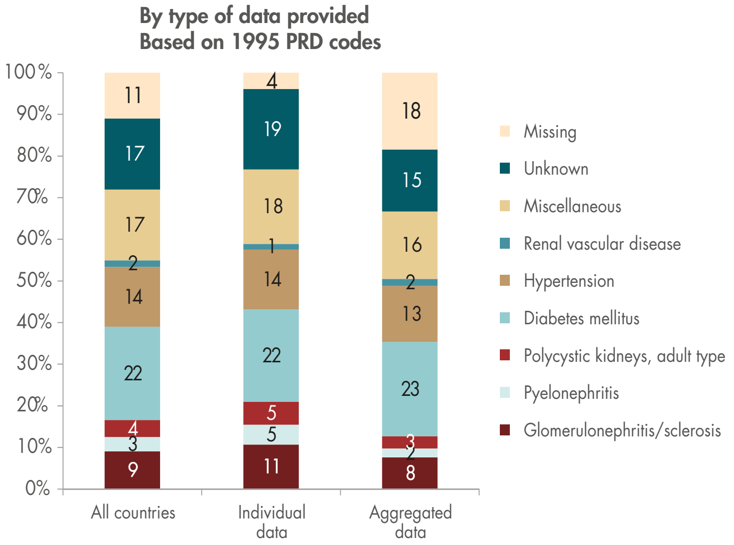

FIND

Figure A.1.3 (continued)

Age, sex, and primary renal disease distribution by type of data provided, unadjusted
at day 1

Based on 2012 / 2018 PRD codes

This figure summarizes the data presented in table B.2.5.B

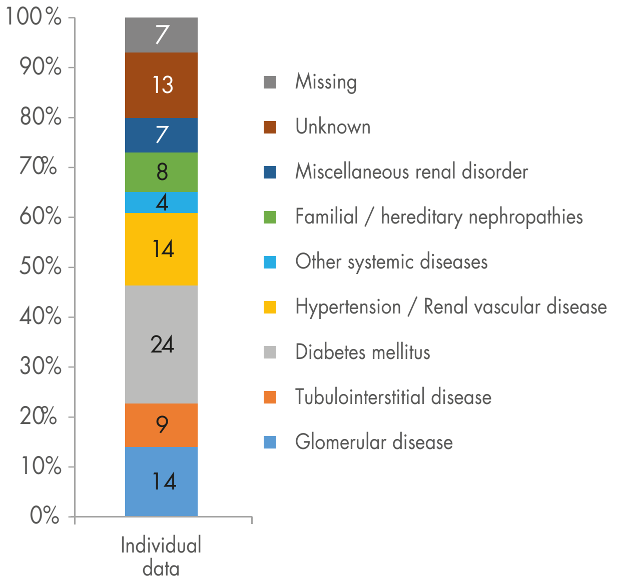

FIND

Figure A.1.4

Treatment modality distribution by type of data provided, age, sex and primary renal disease, unadjusted *at day 1*

This figure summarizes the data presented in tables B.2.10 and C.2.8

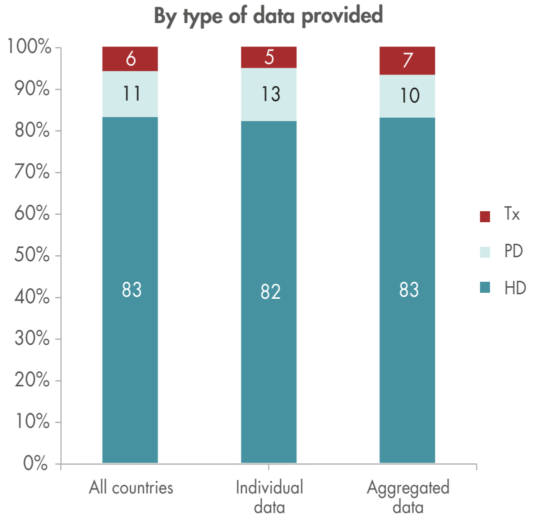

The figures below are only based on data from registries providing individual patient data.

These figures summarize the data presented in table B.2.11

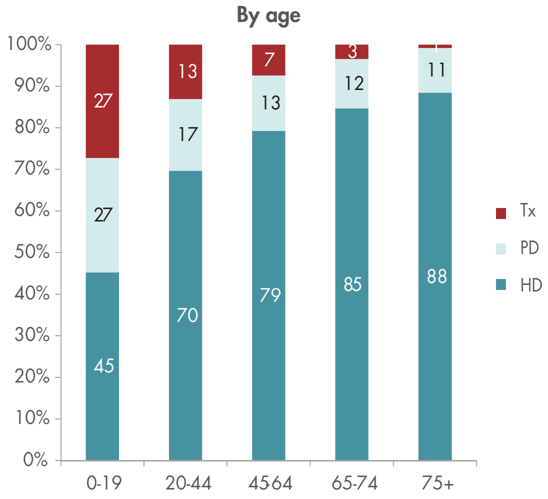

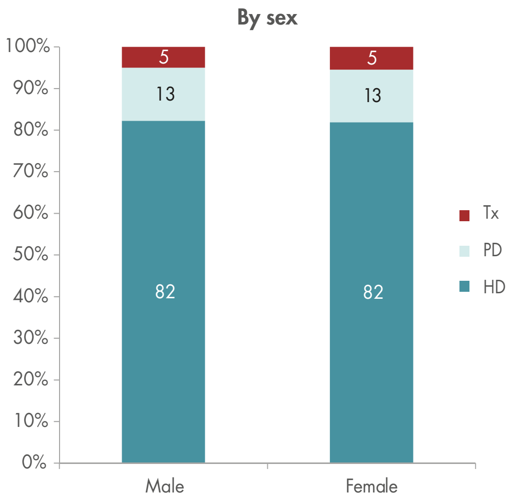

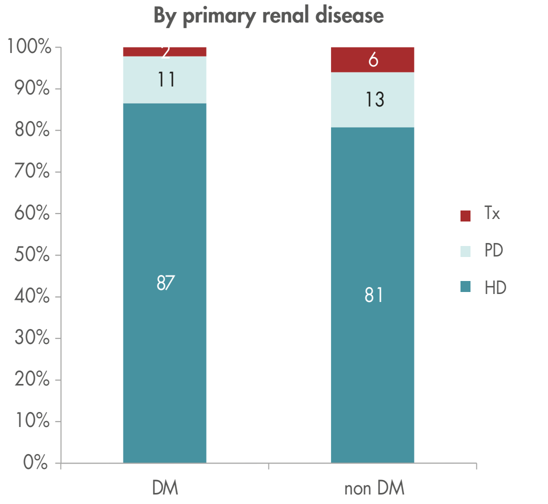

Abbreviations used: HD: haemodialysis; PD: peritoneal dialysis; Tx: transplant; DM: diabetes mellitus

Figure A.2.1

Treatment modality distribution by type of data provided, age, sex and primary renal disease, unadjusted *at day 91*

This figure summarizes the data presented in tables B.3.10 and C.3.8

Not all countries providing aggregated data provide information at treatment day 91. Therefore the percentages in this figure may not be comparable with the percentages in Figure A.1.4.A.

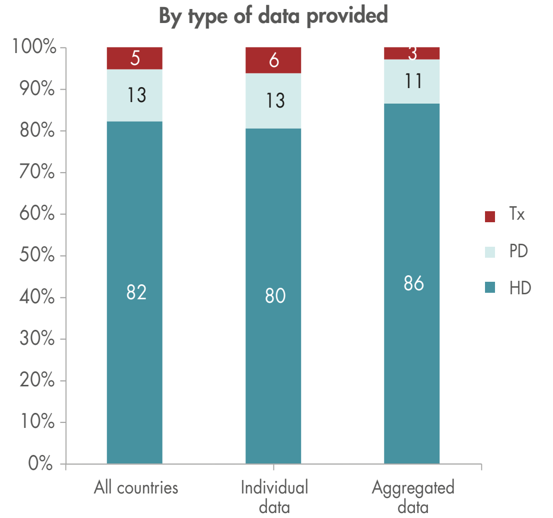

The figures below are only based on data from registries providing individual patient data.

These figures summarize the data presented in table B.3.11

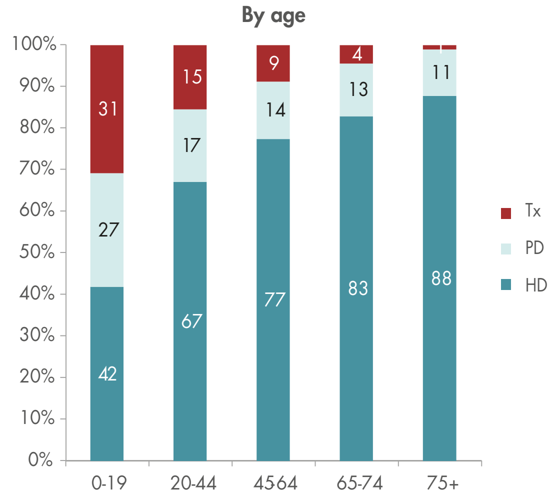

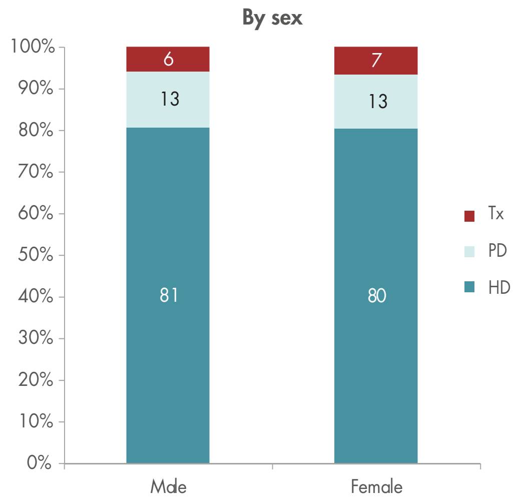

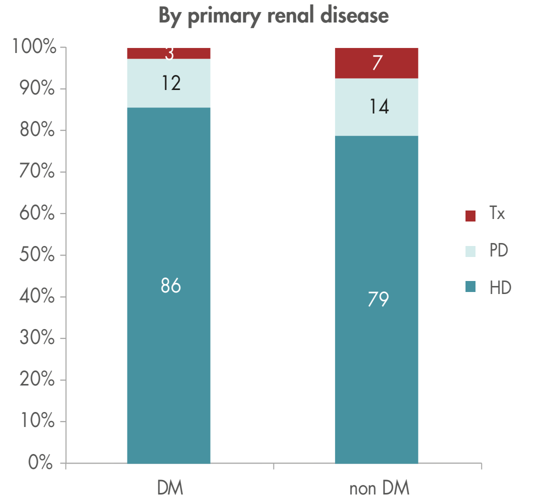

Abbreviations used: HD: haemodialysis; PD: peritoneal dialysis; Tx: transplant; DM: diabetes mellitus

FIND

Table A.3.1

Summary data on the prevalence of KRT on 31 December 2023, unadjusted

	General population covered by the registry in thousands	Prevalent patients on KRT in 2023					
		All N	All Pmp	Mean Age	Median Age	DM N	DM Pmp
Albania	2734	1724	631	52.8	52.9	455	166
Austria $	8923	8985	1007	62.3	64.0	1553	174
Belarus | |	8444	4374	518			532	63
Belgium, Dutch-speaking *	6813	8676	1273	66.8	68.7	1442	212
Belgium, French-speaking *	4967	7242	1458	66.6	68.5	1238	249
Bosnia and Herzegovina	3531	2482	703	60.7	62.5	472	134
Croatia&	3281	2159	658	66.7	69.0	528	161
Czech Republic &	10828	5937	548	67.0	70.0	1448	134
Denmark	5947	6056	1018	59.9	61.2	998	168
Estonia	1302	1092	839	60.6	61.9	187	144
Finland	5492	5292	964	60.2	62.9	1275	232
France	68372	96317	1409	63.6	65.9	15829	232
Greece	10407	15583	1497	66.5	68.5	2746	264
Hungary	9600	8862	923	59.3	61.0	2104	219
Iceland	386	340	882	58.3	59.6	41	106
Ireland	5149	5257	1021	57.9	59.0		
Israel &	9633	7087	736	68.3	70.9	3180	330
Italy (10 of 20 regions)	28614	34633	1210	62.2	65.7	3396	119
Kosovo | |	1688	1063	630	61.1	63.0	296	175
Latvia	1732	1145	661	56.8	59.0	142	82
Lithuania #	2857	2466	863			196	129
Netherlands	17162	18342	1069	61.3	63.3	2432	142
North Macedonia	1826	1779	974	60.2	62.0	334	183
Norway	5520	5605	1015	60.4	62.5	725	131
Poland &	37637	20536	546			4816	128
Portugal ¤	10640	21511	2022	67.7		3709	536
Romania	19061	25442	1335	65.0	67.0	2336	123
Serbia	6391	5881	920	61.8	63.8	1020	160
Slovakia &	4472	3183	712	64.1	67.0	876	196
Spain (All)	48085	67604	1406	61.4	65.0	10983	228
Spain, Andalusia	8608	11676	1356	62.1	63.6	1961	228
Spain, Aragon	1346	2086	1549	66.5	68.4	363	270
Spain, Basque country	2222	2964	1334	62.5	64.8	417	188
Spain, Canary Islands	2226	3581	1609	63.1	64.0	898	403
Spain, Cantabria *	590	751	1274	64.9	66.3	115	195
Spain, Castile and León *	2384	3300	1384	66.3	67.3	562	236
Spain, Castile-La Mancha *	2094	2628	1255	64.9	65.6	444	212
Spain, Catalonia	7902	12267	1552	63.6	65.3	1895	240
Spain, Community of Madrid	6872	8602	1252	63.0	64.6	1419	206
Spain, Extremadura	1054	1458	1383	64.4	65.5	239	227
Spain, Galicia	2703	4087	1512	64.5	65.9	672	249
Spain, La Rioja	323	399	1234	62.4	62.8	50	155
Spain, Murcia	1552	2239	1443	63.2	64.6	362	233
Spain, Navarre *	675	948	1404	63.6	65.4	156	231
Spain, Valencian region	5216	7753	1486	64.3	66.2	1127	216
Sweden	10537	10649	1011	60.7	62.6	1795	170
Switzerland	8444	8659	1025	63.5	65.5	1119	133
Türkiye ‡	85372	89527	1049			6131	350
United Kingdom, England	53075	55070	1038	58.4	59.9	10104	190
United Kingdom, Northern Ireland	1920	2123	1106	58.8	60.4	310	161
United Kingdom, Scotland	5490	5725	1043	58.0	59.7	961	175
United Kingdom, Wales	3164	3498	1105	58.8	60.0	651	206
All countries	519496	571906	1101	62.4	64.5	86360	196

DM = diabetes mellitus as primary renal disease
When cells are left empty, the data are unavailable and could not be used for the calculation of the summary data
$ The prevalence is underestimated by approximately 2% due to one haemodialysis centre not submitting data
| | Patients younger than 18 years of age are not reported
* Patients younger than 20 years of age are not reported
& Data on prevalence include dialysis patients only
# Data on DM are extrapolated from data of 1314 patients (53.3% of total)
¤ Data on DM are extrapolated from data of 13976 patients (65.0% of total)
‡ Data on DM are extrapolated from data of 18378 patients (20.5% of total)

Figure A.3.1

Prevalence per million population by country / region, unadjusted

prevalent patients on December 31

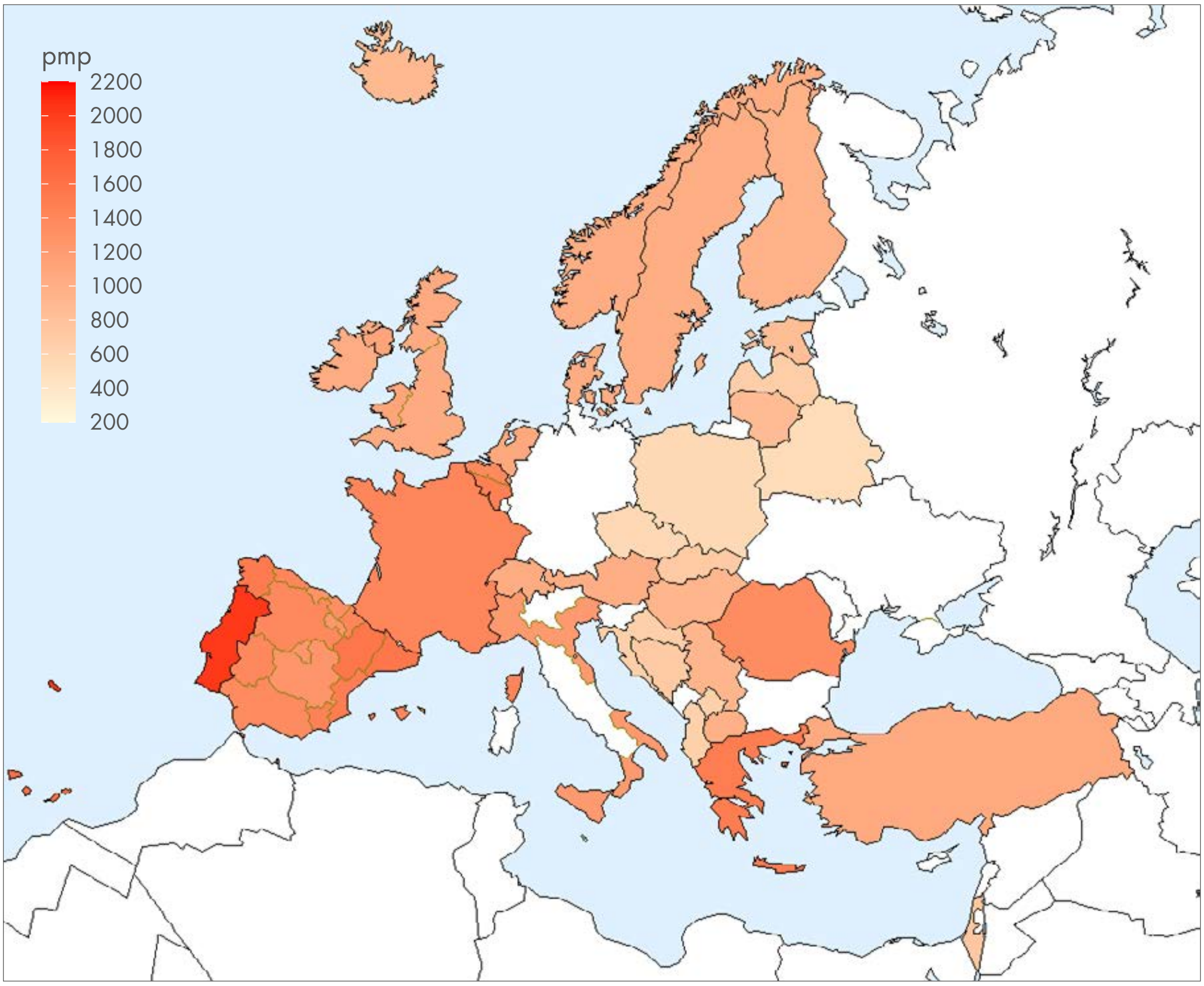

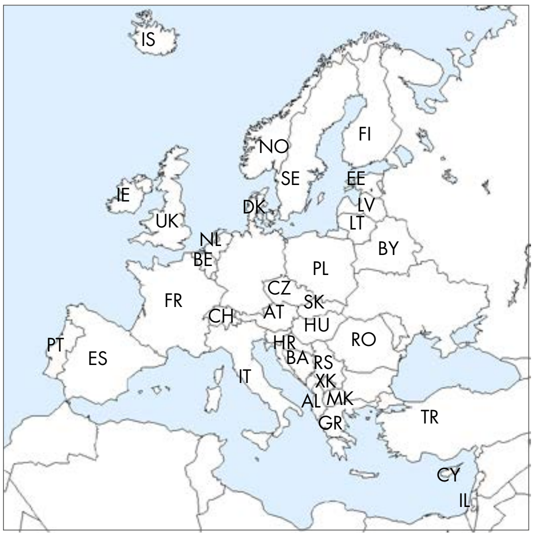

AL	Albania	IL	Israel
AT	Austria	IS	Iceland
BA	Bosnia and Herzegovina	IT	Italy
BE	Belgium	LT	Lithuania
BY	Belarus	LV	Latvia
CH	Switzerland	MK	North Macedonia
CY	Cyprus	NL	Netherlands
CZ	Czech Republic	NO	Norway
DK	Denmark	PL	Poland
EE	Estonia	PT	Portugal
ES	Spain	RO	Romania
FI	Finland	RS	Serbia
FR	France	SE	Sweden
GR	Greece	SK	Slovakia
HR	Croatia	TR	Türkiye
HU	Hungary	UK	United Kingdom
IE	Ireland	XK	Kosovo

Detailed information on the contents of this figure can be found in table B.4.2 for registries providing individual patient data, and in table C.4.2 for registries providing aggregated data.

Figure A.3.2

Prevalence per million population by country / region, unadjusted and adjusted

prevalent patients on December 31

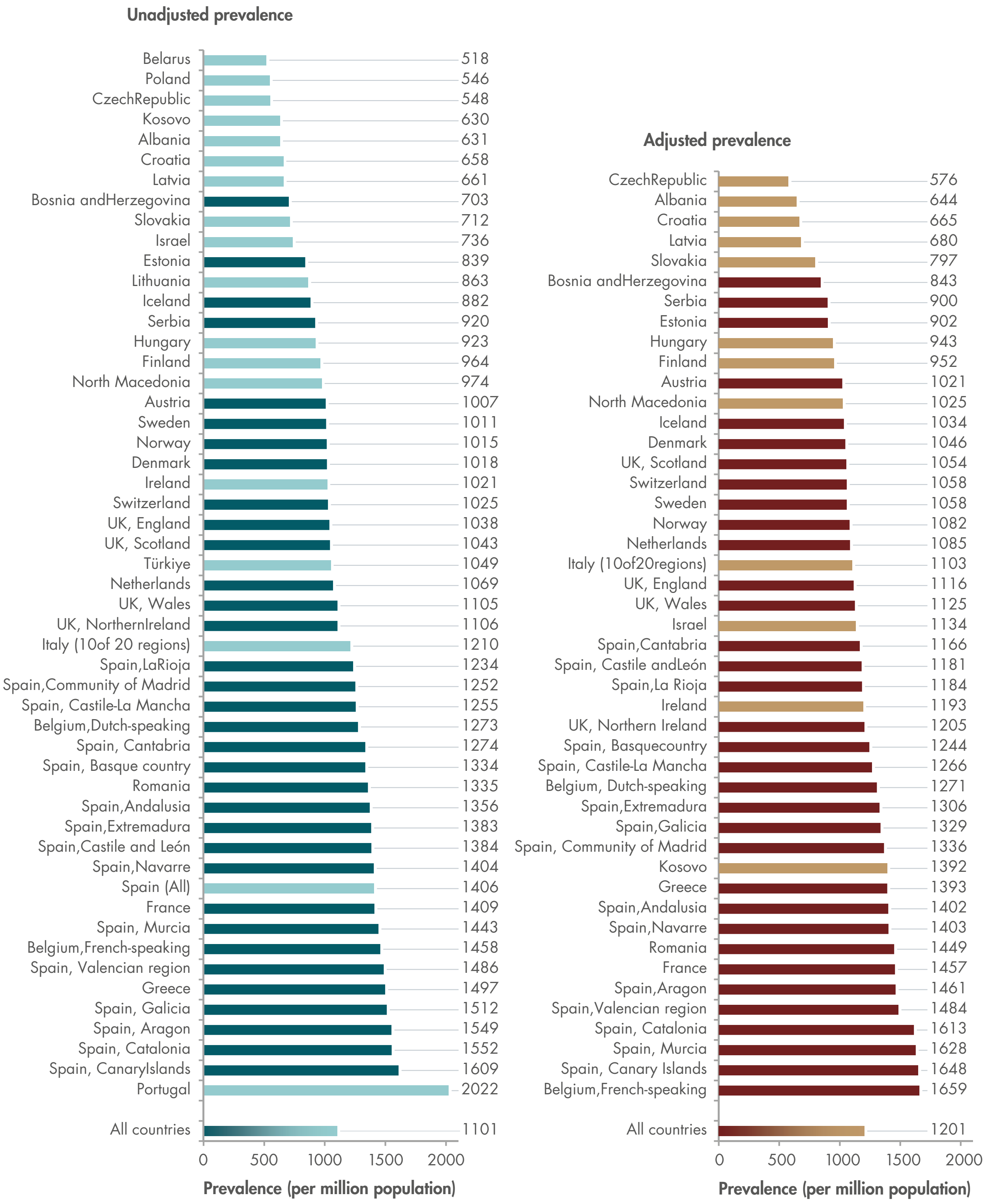

Registries providing individual patient data are shown as dark bars, and registries providing aggregated data as light bars.
Detailed information on the contents of this fi gure can be found in tables B.4.2 and B.4.4 for registries providing individual patient data, and in tables C.4.2 and C.4.4 for registries providing aggregated data.
Adjustment of the prevalence was performed by standardising the prevalence to the age and sex distribution of the EU27 population.

Figure A.3.3

Age, sex, and primary renal disease distribution by type of data provided, unadjusted

prevalent patients on December 31

This figure summarizes the data presented in tables B.4.1 and C.4.1

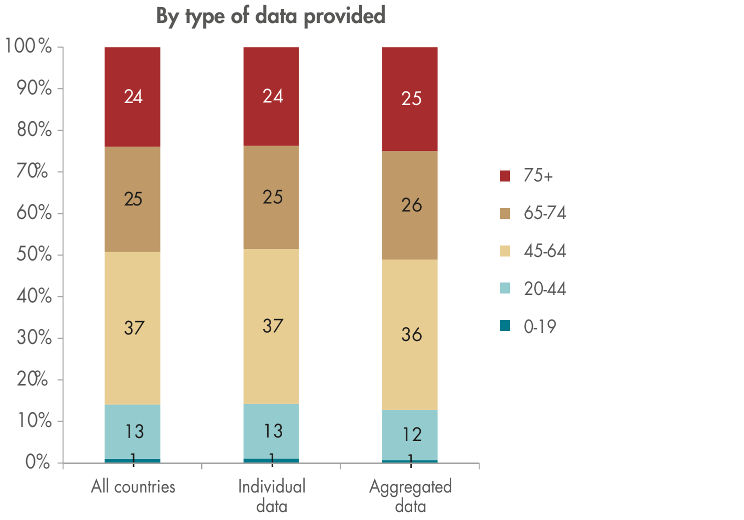

This figure summarizes the data presented in tables B.4.3 and C.4.3

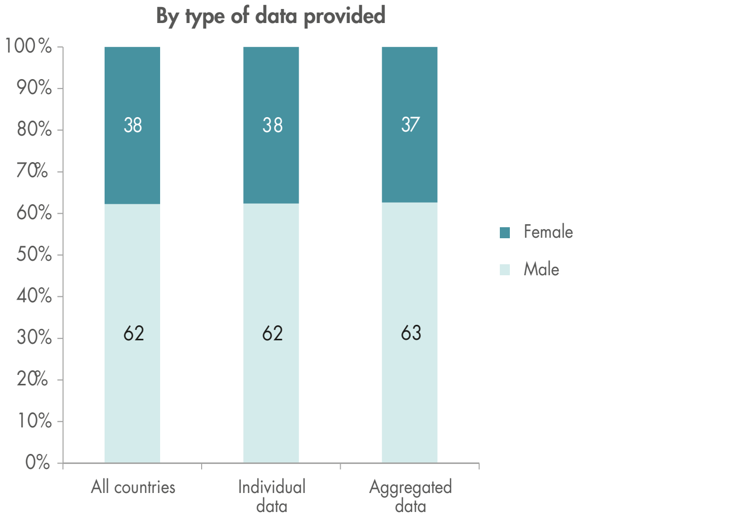

This figure summarizes the data presented in tables B.4.5 and C.4.5

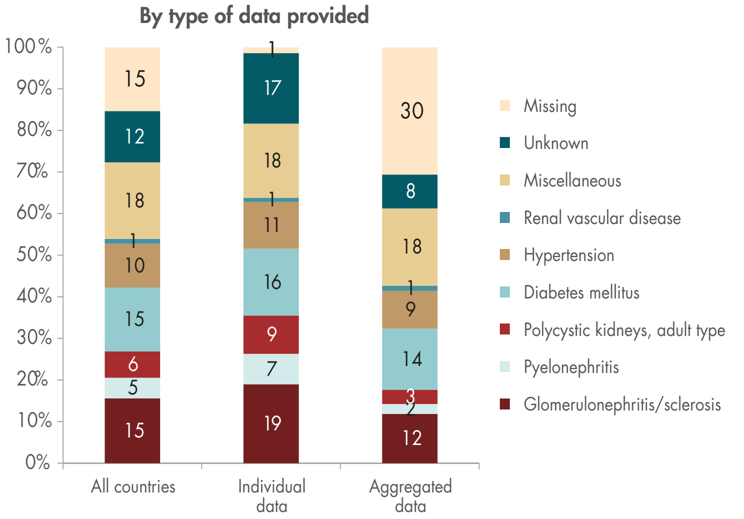

Figure A.3.4

Treatment modality distribution by type of data provided, age, sex and primary renal disease, unadjusted

prevalent patients on December 31

This figure summarizes the data presented in tables B.4.10 and C.4.8

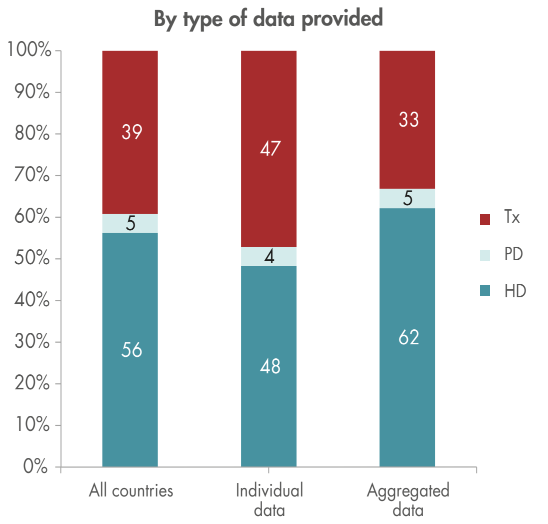

The figures below are only based on data from registries providing individual patient data.

These figures summarize the data presented in table B.4.11

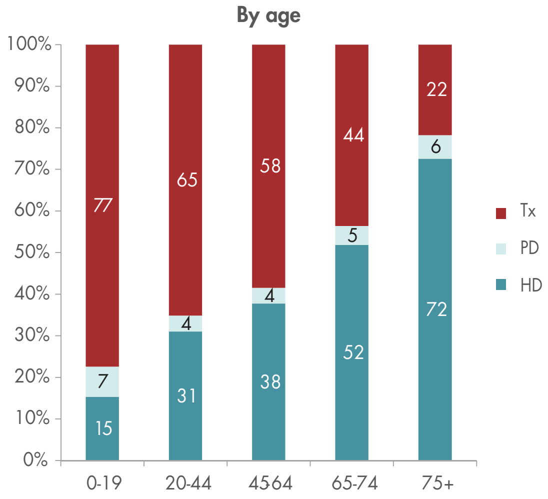

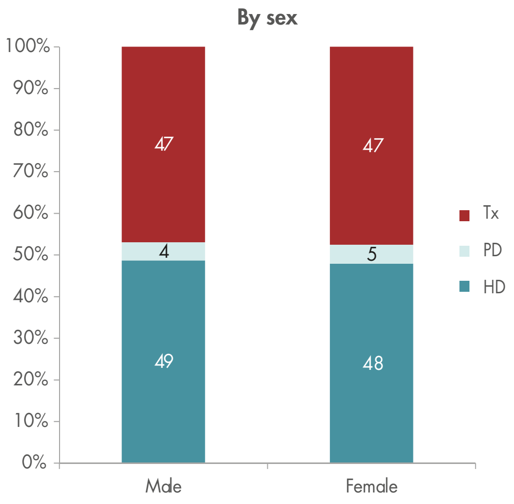

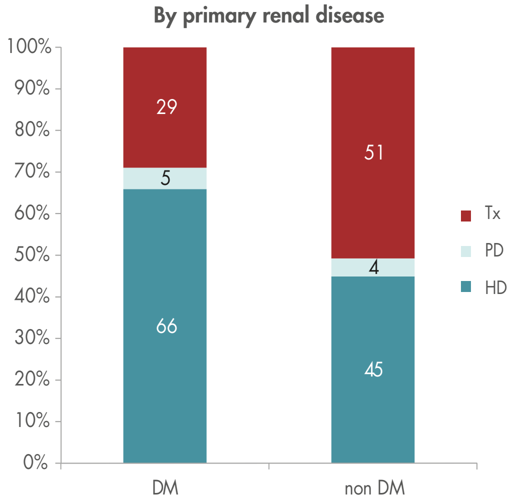

Abbreviations used: HD: haemodialysis; PD: peritoneal dialysis; Tx: transplant; DM: diabetes mellitus

FIND

Figure A.4.1

Kidney transplant counts and per million population by country / region, unadjusted

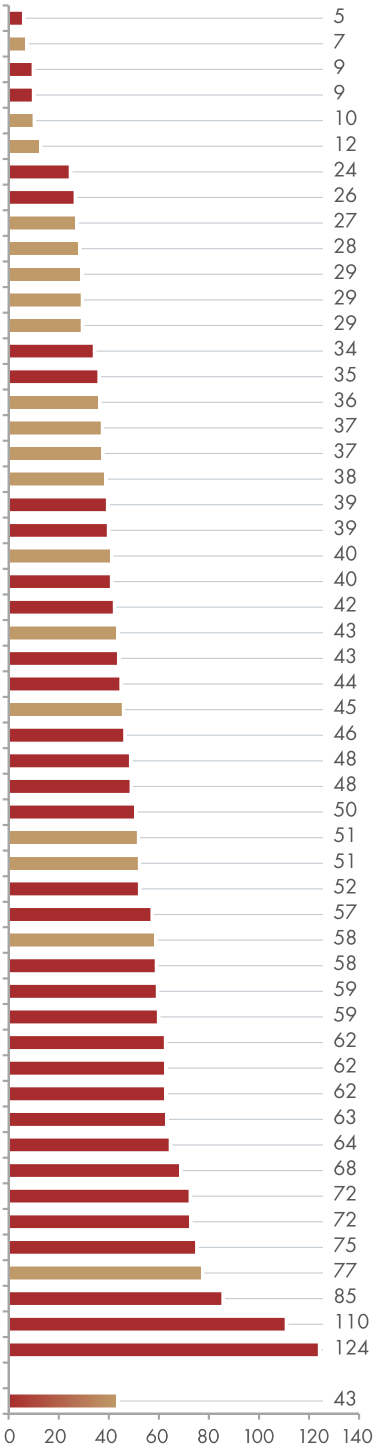

Unadjusted kidney transplant rate	Rate (pmp)	All N	% Deceased donor	% Living donor	% Unknown donor type
Bosnia and Herzegovina	5	19	42	58	0
Kosovo	7	11	0	100	0
Serbia	9	59	54	44	2
Romania	9	177	68	32	0
Albania	10	26	0	100	0
NorthMacedonia	12	22	27	73	0
Greece	24	250	60	40	0
Iceland	26	10	80	20	0
Latvia	27	46	93	7	0
Hungary	28	265	83	17	0
Poland	29	1075	93	7	0
Slovakia	29	128	88	13	0
Italy(10 of 20 regions)	29	823	88	11	1
Austria	34	300	79	21	0
Belgium, French-speaking	35	176	79	19	2
Lithuania	36	102	94	6	0
Ireland	37	189	84	16	0
Cyprus	37	35	51	49	0
Belarus	38	322	99	1	0
Estonia	39	51	90	10	0
Belgium,Dutch-speaking	39	268	74	10	16
Türkiye	40	3452	9	91	0
UK, Wales	40	128	67	33	0
Spain, Castile-LaMancha	42	87	95	3	1
Croatia	43	141	92	8	0
Norway	43	239	80	20	0
Switzerland	44	374	73	27	0
Czech Republic	45	488	93	7	0
UK,England	46	2429	73	27	0
Sweden	48	507	80	20	0
UK,Scotland	48	265	65	34	1
Denmark	50	298	70	30	0
Israel	51	492	32	67	1
Portugal	51	547	87	13	0
France	52	3526	84	16	0
Netherlands	57	983	50	50	0
Finland	58	319	87	13	0
UK, Northern Ireland	58	112	50	50	0
Spain, La Rioja	59	19	95	5	0
Spain, Castile and León	59	141	92	8	0
Spain,Community ofMadrid	62	426	84	15	0
Spain, Galicia	62	168	86	14	0
Spain, Navarre	62	42	100	0	0
Spain,Extremadura	63	66	95	5	0
Spain, Aragon	64	86	88	12	0
Spain, Valencian region	68	355	96	4	0
Spain, Canary Islands	72	160	94	6	0
Spain,Basque country	72	160	88	12	0
Spain, Andalusia	75	642	91	9	0
Spain (All)	77	3690	88	12	0
Spain, Murcia	85	132	92	8	0
Spain, Cantabria	110	65	98	2	0
Spain, Catalonia	124	976	81	19	0
All countries	43	22344	69	31	0

Kidney transplant rate (per million population): 0, 20, 40, 60, 80, 100, 120, 140

Registries providing individual patient data are shown as red bars, and registries providing aggregated data as orange bars. Detailed information on the contents of this fi gure can be found in tables B.5.1 and B.5.2 for registries providing individual patient data, and in tables C.5.1 and C.5.2 for registries providing aggregated data.

FIND

Figure A.4.2

Kidney transplants per million population by donor type and by country / region, unadjusted

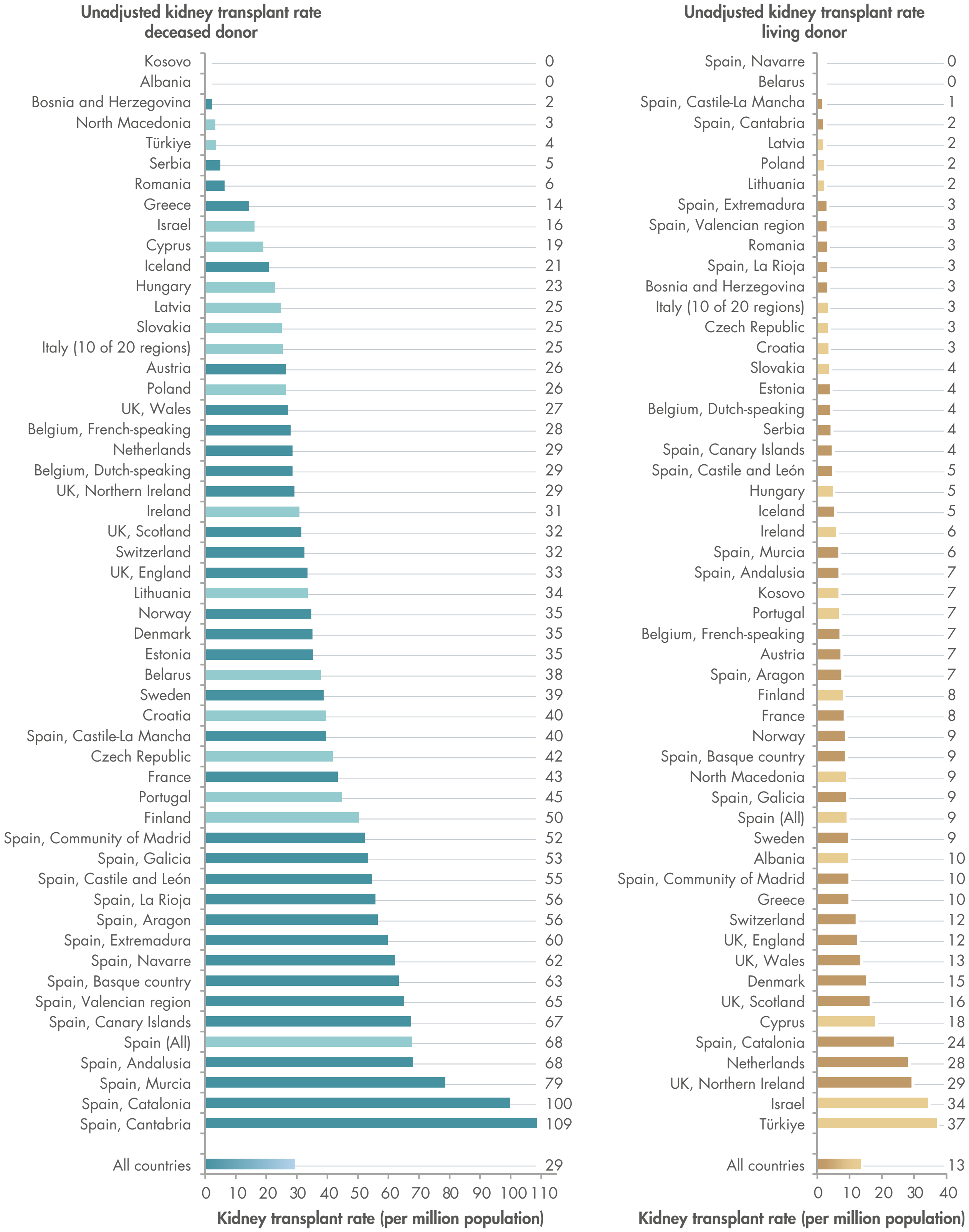

Registries providing individual patient data are shown as dark bars, and registries providing aggregated data as light bars. Detailed information on the contents of this fi gure can be found in table B.5.2 for registries providing individual patient data, and in table C.5.2 for registries providing aggregated data.

FIND

Figure A.4.3
Donor type distribution by type of data provided, unadjusted

This figure summarizes the data presented in table B.5.1 for registries providing individual patient data, and in table C.5.1 for registries providing aggregated data.

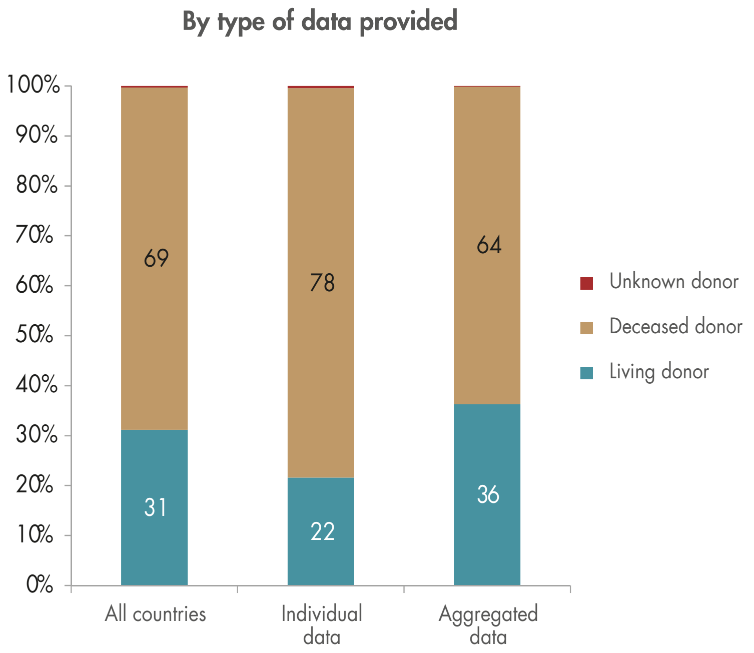

Table A.5.1

One-, two- and five-year survival probabilities by treatment modality and cohort

from day 1 of KRT / dialysis, or from day of transplant

	Survival probabilities as % (95% CI)				
	cohort 2014 - 2018			cohort 2017 - 2021	
	1 year	2 year	5 year	1 year	2 year
**Patient survival on KRT**					
Unadjusted	85.5 (85.3-85.7)	75.5 (75.3-75.7)	51.3 (51.1-51.6)	85.7 (85.5-85.9)	75.7 (75.5-75.9)
Adjusted †	88.3 (88.2-88.5)	79.4 (79.2-79.6)	54.0 (53.8-54.3)	88.3 (88.2-88.5)	79.3 (79.1-79.5)
**Patient survival on dialysis**					
Unadjusted	84.4 (84.3-84.6)	72.8 (72.6-73.0)	41.4 (41.1-41.6)	84.7 (84.6-84.9)	73.3 (73.1-73.5)
Adjusted †	86.7 (86.6-86.9)	76.4 (76.2-76.6)	46.7 (46.4-47.0)	87.1 (87.0-87.3)	77.0 (76.8-77.2)
**Patient survival after first kidney transplantation (deceased donor)**					
Unadjusted	96.3 (96.1-96.5)	94.1 (93.8-94.3)	84.3 (84.0-84.7)	95.8 (95.6-96.0)	93.0 (92.8-93.3)
Adjusted ††	98.2 (98.1-98.3)	97.0 (96.9-97.2)	91.6 (91.3-91.9)	98.1 (98.0-98.2)	96.7 (96.5-96.9)
**Graft survival after first kidney transplantation (deceased donor)**					
Unadjusted	91.1 (90.8-91.4)	87.7 (87.4-88.0)	75.4 (75.0-75.8)	90.9 (90.6-91.2)	87.2 (86.9-87.6)
Adjusted ††	93.3 (93.0-93.5)	90.6 (90.4-90.9)	80.6 (80.2-81.0)	93.4 (93.1-93.6)	90.6 (90.3-90.9)
**Patient survival after first kidney transplantation (living donor)**					
Unadjusted	98.8 (98.6-99.0)	98.0 (97.8-98.2)	94.0 (93.6-94.4)	98.8 (98.6-99.0)	97.9 (97.6-98.1)
Adjusted ††	99.1 (99.0-99.3)	98.5 (98.3-98.7)	95.2 (94.8-95.6)	99.1 (98.9-99.3)	98.4 (98.1-98.6)
**Graft survival after first kidney transplantation (living donor)**					
Unadjusted	96.5 (96.2-96.8)	95.0 (94.6-95.3)	88.0 (87.4-88.5)	96.9 (96.6-97.2)	95.3 (94.9-95.7)
Adjusted ††	96.4 (96.1-96.8)	94.8 (94.4-95.2)	87.6 (87.0-88.2)	96.8 (96.5-97.2)	95.1 (94.7-95.5)

† Analyses were adjusted using fixed values: age (67 years), sex (63% male) and PRD (24% diabetes mellitus, 19% hypertension/renal vascular disease, 11% glomerulonephritis and 46% other causes)
†† Analyses were adjusted using fixed values: age (50 years), sex (63% male) and PRD (14% diabetes mellitus, 10% hypertension/renal vascular disease, 23% glomerulonephritis and 53% other causes)

This table summarizes the survival data presented in the tables B.6.1 to B.6.16, and is based on data from the following registries providing individual patient data: Austria, Belgium (Dutch-speaking), Belgium (French-speaking), Bosnia and Herzegovina, Denmark, Estonia, France, Greece, Iceland, Netherlands, Norway, Spain (Andalusia), Spain (Aragon), Spain (Basque country), Spain (Canary Islands), Spain (Cantabria), Spain (Castile and León), Spain (Castile-La Mancha), Spain (Catalonia), Spain (Community of Madrid), Spain (Extremadura), Spain (Galicia), Spain (Murcia), Spain (Navarre), Spain (Valencian Region), Sweden, United Kingdom (England/Northern Ireland/Wales) and UnitedKingdom (Scotland).

Figure A.5.1

Patient survival by modality:
Incident dialysis patients
(cohort 2014-2018)
from day 91, unadjusted

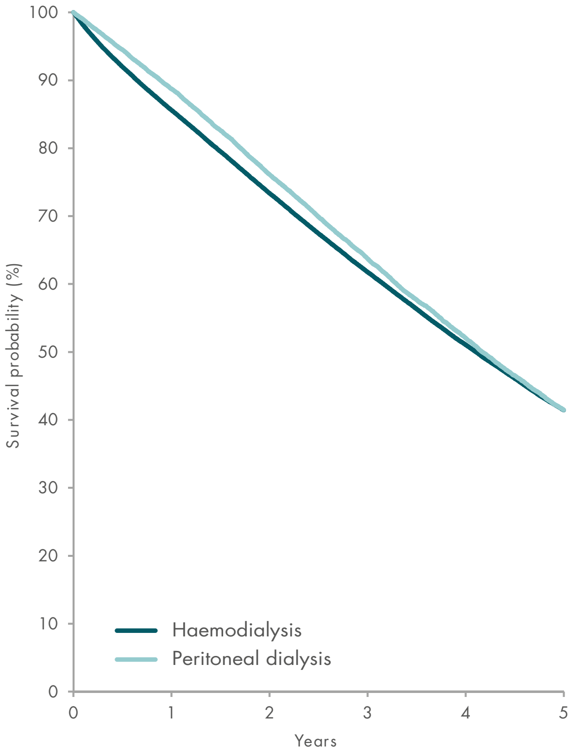

Figure A.5.2

Patient survival by donor type:
Patients receiving a fi rst kidney transplant
(cohort 2014-2018)
from day of transplant, unadjusted

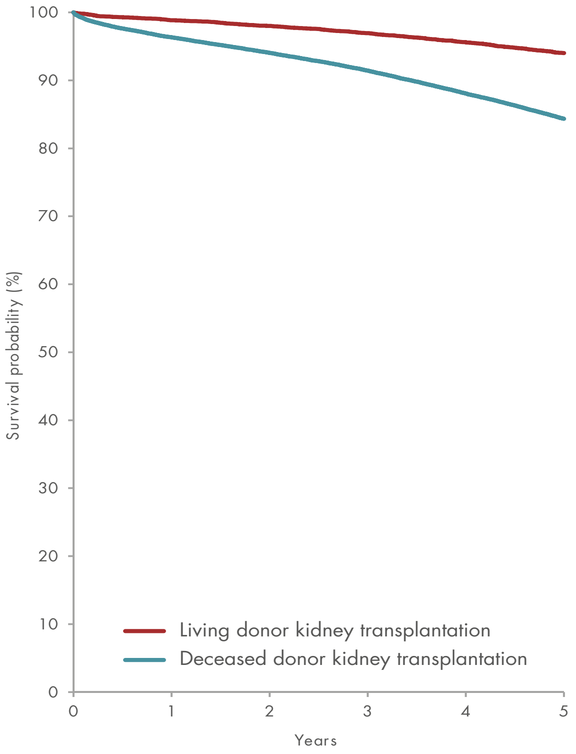

These figures are based on patients starting dialysis or receiving a kidney transplant between 2014 and 2018 from the following registries providing individual patient data: Austria, Belgium (Dutch-speaking), Belgium (French-speaking), Bosnia and Herzegovina, Denmark, Estonia, France, Greece, Iceland, Netherlands, Norway, Spain (Andalusia), Spain (Aragon), Spain (Basque country), Spain (Canary Islands), Spain (Cantabria), Spain (Castile and León), Spain (Castile-La Mancha), Spain (Catalonia), Spain (Community of Madrid), Spain (Extremadura), Spain (Galicia), Spain (Murcia), Spain (Navarre), Spain (Valencian Region), Sweden, United Kingdom (England/Northern Ireland/Wales) and United Kingdom (Scotland).

Figure A.6.1

Expected remaining years of life in the general population and in prevalent dialysis and kidney transplant patients (cohort 2019-2023)

by age

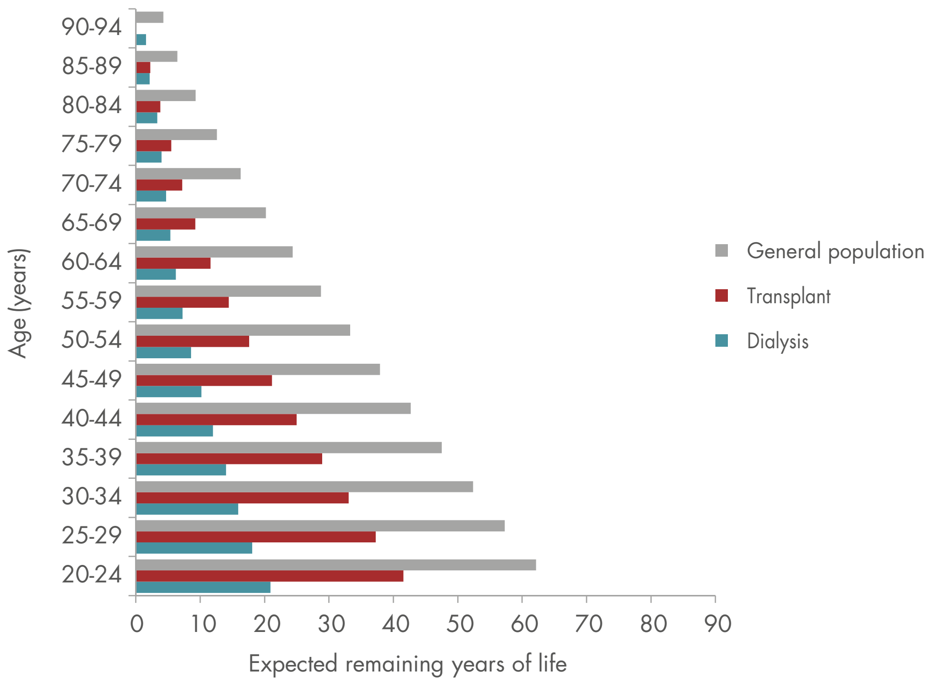

This figure summarizes the data presented in table B.7.1, and is based on data from the following registries providing individual patient data: Austria, Belgium (Dutch-speaking), Belgium (French-speaking), Bosnia and Herzegovina, Denmark, Estonia, France, Greece, Iceland, Netherlands, Norway, Spain (Andalusia), Spain (Aragon), Spain (Basque country), Spain (Canary Islands), Spain (Canta-bria), Spain (Castille and León), Spain (Castille-La Mancha), Spain (Catalonia), Spain (Communityof Madrid), Spain (Extremadura), Spain (Galicia), Spain (Murcia), Spain (Navarre), Spain (Valencian Region), Sweden, United Kingdom (England/Northern Ireland/Wales) and United Kingdom (Scotland).

Figure A.7.1

Age distribution by country / region, unadjusted

incident patients at day 1

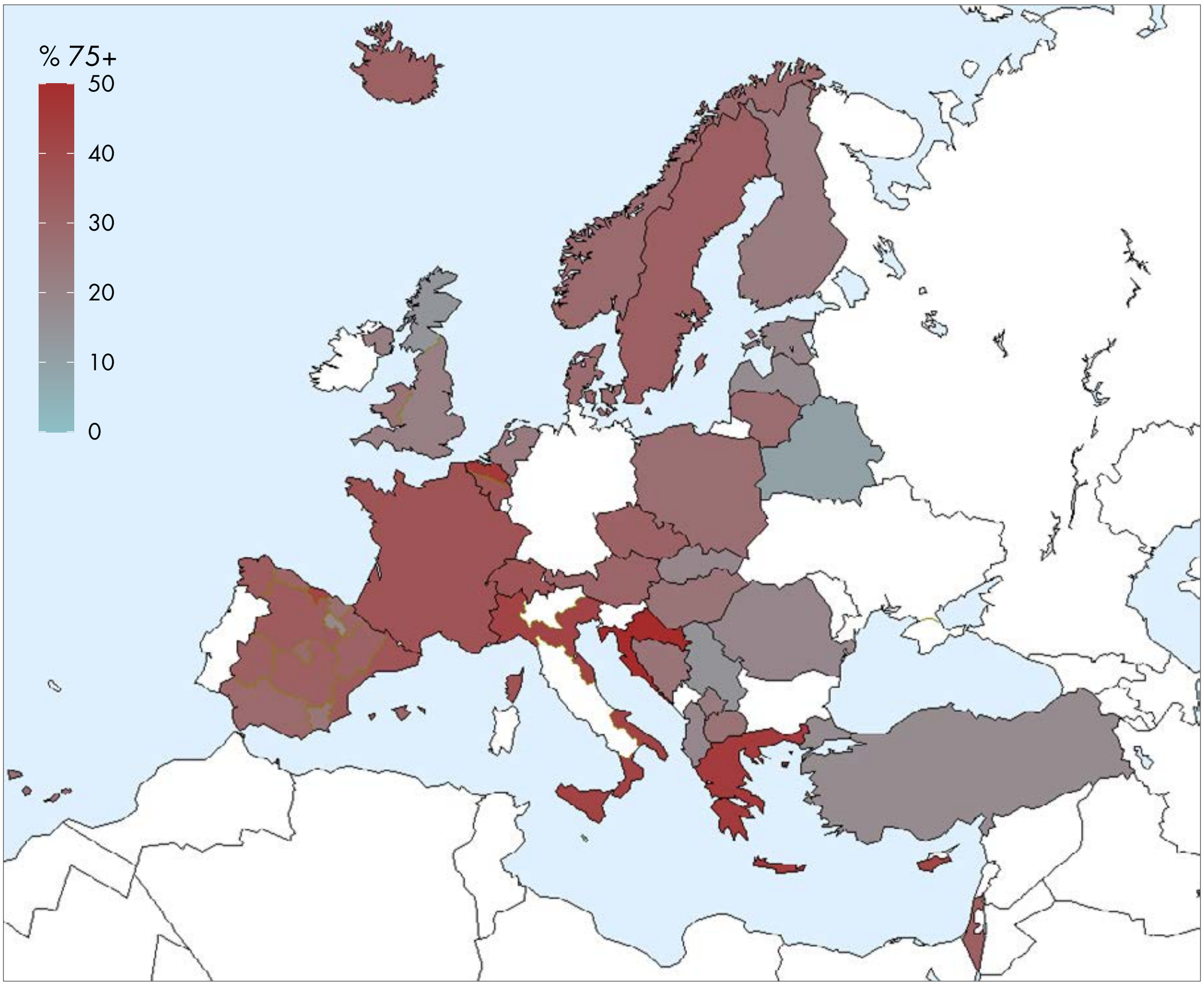

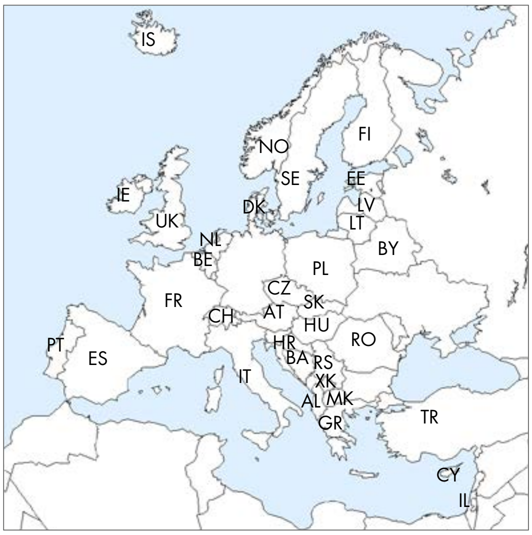

AL	Albania	IL	Israel
AT	Austria	IS	Iceland
BA	Bosnia and Herzegovina	IT	Italy
BE	Belgium	LT	Lithuania
BY	Belarus	LV	Latvia
CH	Switzerland	MK	North Macedonia
CY	Cyprus	NL	Netherlands
CZ	Czech Republic	NO	Norway
DK	Denmark	PL	Poland
EE	Estonia	PT	Portugal
ES	Spain	RO	Romania
FI	Finland	RS	Serbia
FR	France	SE	Sweden
GR	Greece	SK	Slovakia
HR	Croatia	TR	Türkiye
HU	Hungary	UK	United Kingdom
IE	Ireland	XK	Kosovo

Detailed information on the contents of this figure can be found in table B.2.1 for registries providing individual patient data, and in table C.2.1 for registries providing aggregated data.

FIND

Figure A.7.2

Incidence per million age-related population by age, unadjusted

at day 1

This figure summarizes the data presented in table B.2.2 and C.2.2

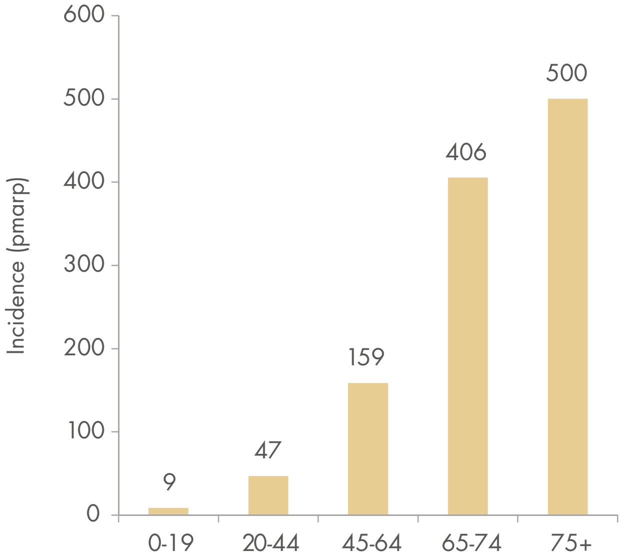

Figure A.7.3

Sex, primary renal disease and treatment modality distribution by age in incident patients, unadjusted *at day 1*

This figure summarizes the data presented in table B.2.1

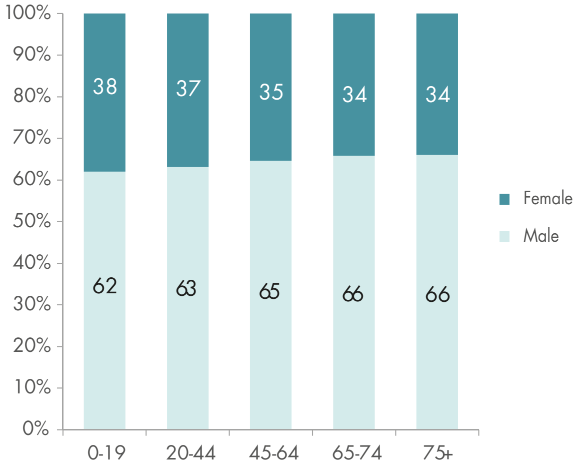

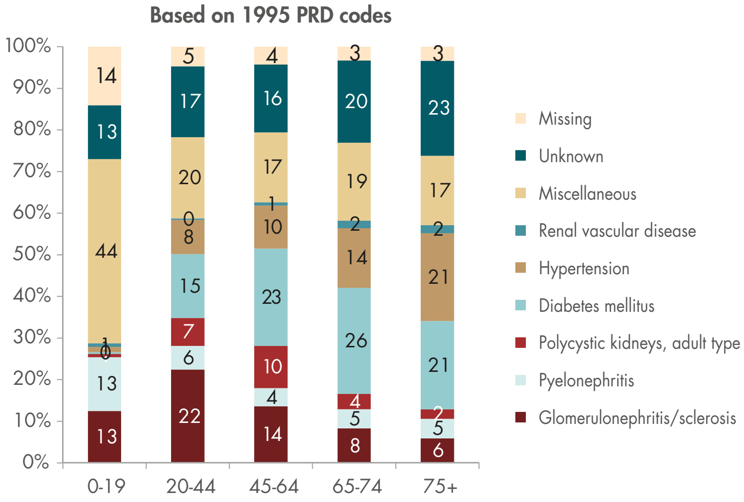

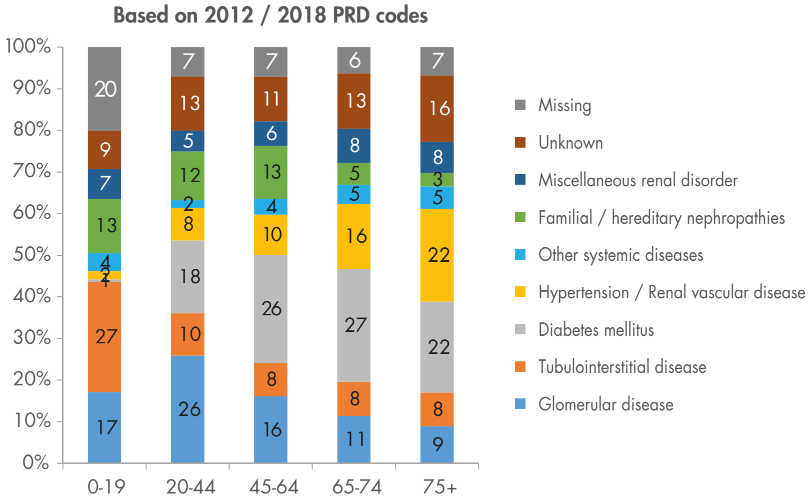

FIND

Figure A.7.3 (continued)

Sex, primary renal disease and treatment modality distribution by age in incident patients, unadjusted
at day 1

This figure summarizes the data presented in table B.2.11

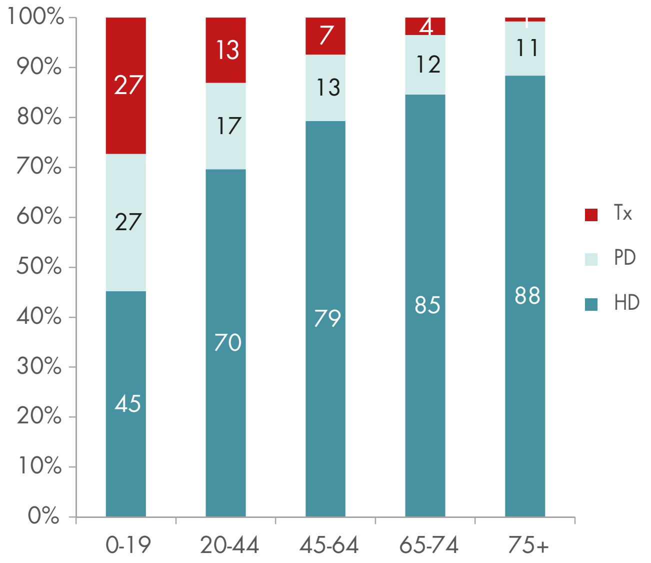

Abbreviations used: HD: haemodialysis; PD: peritoneal dialysis; Tx: transplant

Figure A.7.4

Age distribution by country / region, unadjusted

prevalent patients on December 31

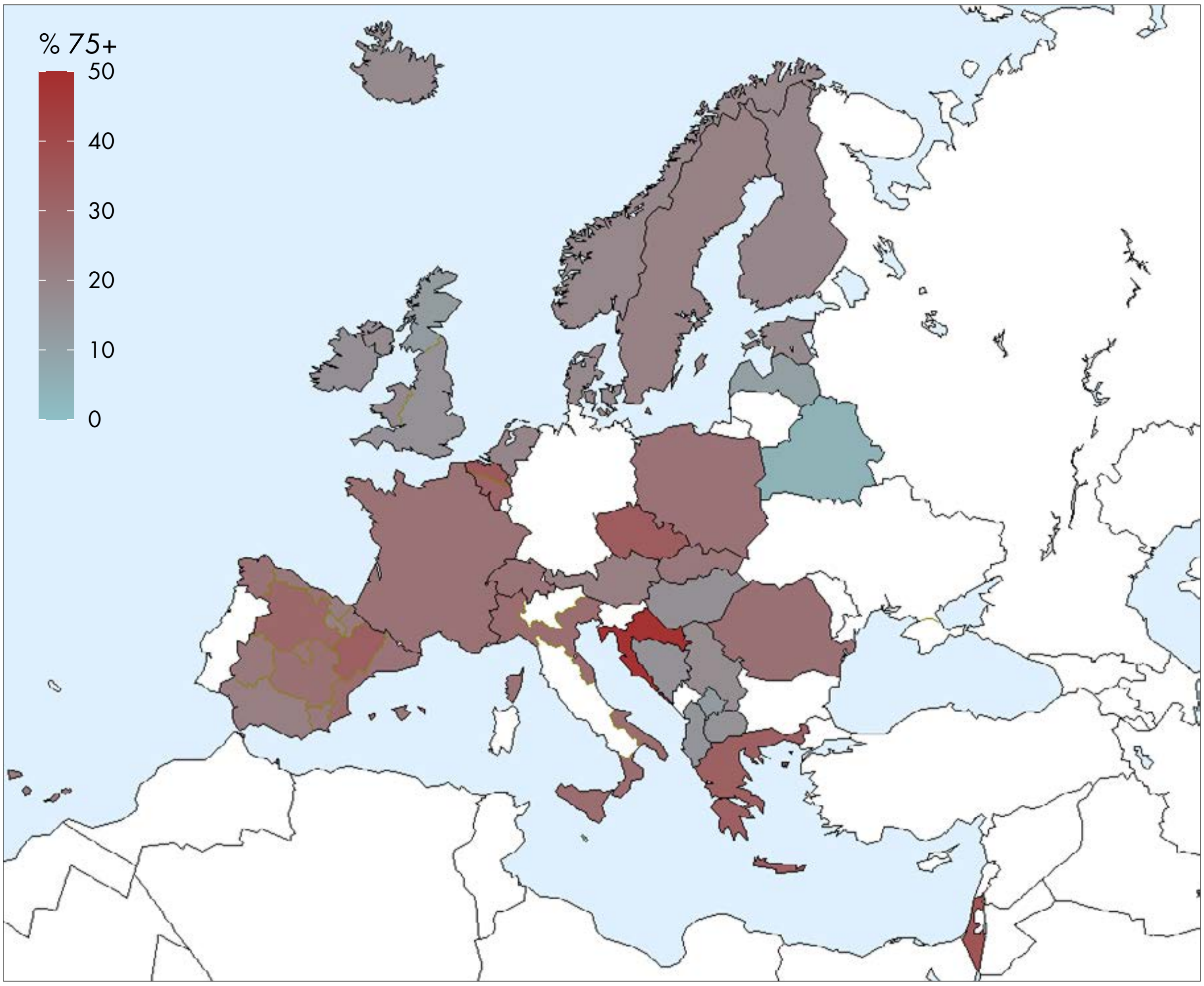

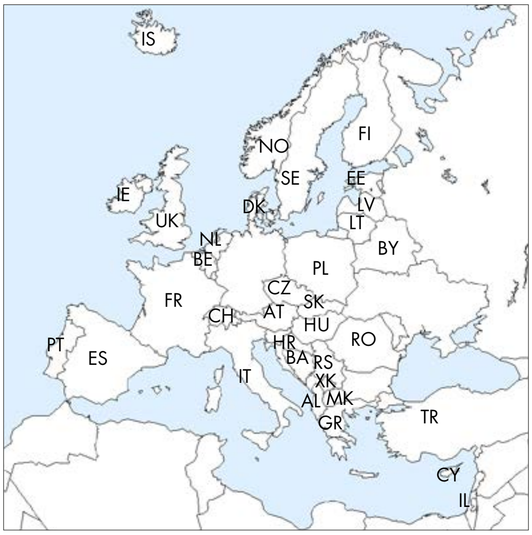

AL	Albania	IL	Israel
AT	Austria	IS	Iceland
BA	Bosnia and Herzegovina	IT	Italy
BE	Belgium	LT	Lithuania
BY	Belarus	LV	Latvia
CH	Switzerland	MK	North Macedonia
CY	Cyprus	NL	Netherlands
CZ	Czech Republic	NO	Norway
DK	Denmark	PL	Poland
EE	Estonia	PT	Portugal
ES	Spain	RO	Romania
FI	Finland	RS	Serbia
FR	France	SE	Sweden
GR	Greece	SK	Slovakia
HR	Croatia	TR	Türkiye
HU	Hungary	UK	United Kingdom
IE	Ireland	XK	Kosovo

Detailed information on the contents of this figure can be found in table B.4.1 for registries providing individual patient data, and in table C.4.1 for registries providing aggregated data.

Figure A.7.5

Prevalence per million age-related population by age, unadjusted

prevalent patients on December 31

This figure summarizes the data presented in tables B.4.2 and C.4.2

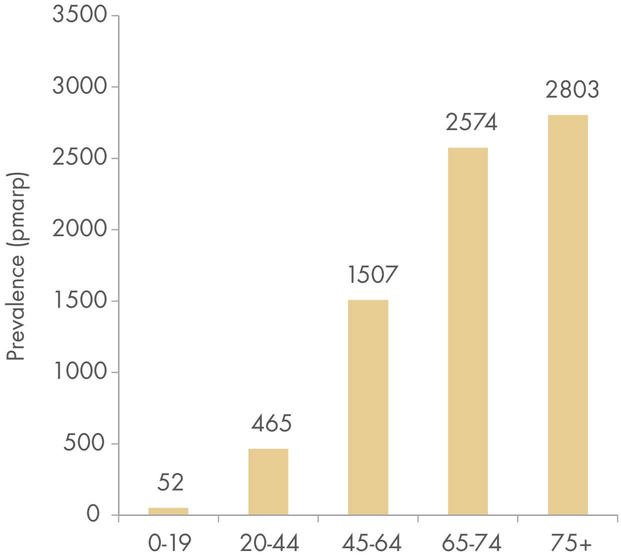

Figure A.7.6

Sex, primary renal disease and treatment modality distribution by age in prevalent patients, unadjusted

prevalent patients on December 31

This figure summarizes the data presented in table B.4.1

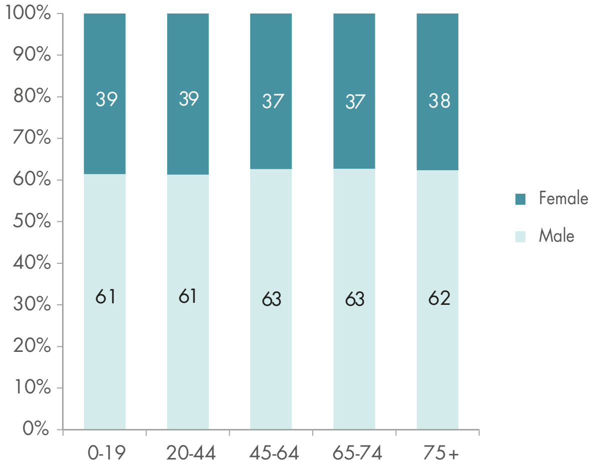

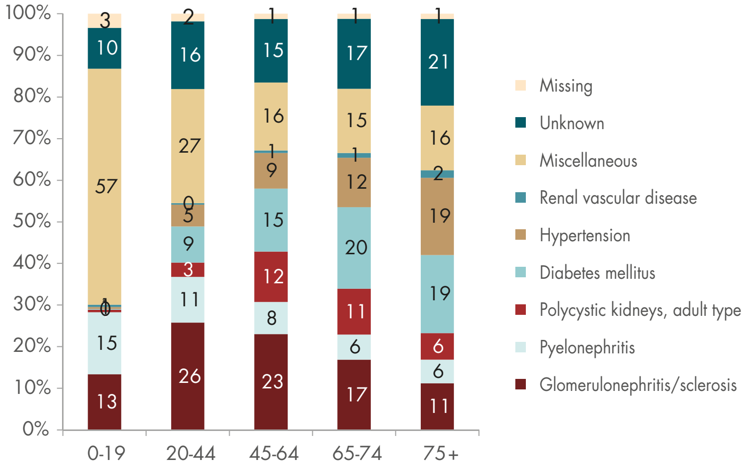

This figure summarizes the data presented in table B.4.11

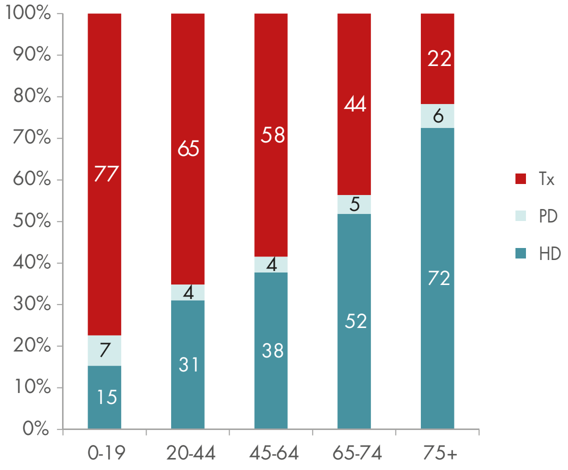

Abbreviations used: HD: haemodialysis; PD: peritoneal dialysis; Tx: transplant

Figure A.7.7

Kidney transplant counts and percentages by recipient age and transplant activity per million age-related population by age, unadjusted

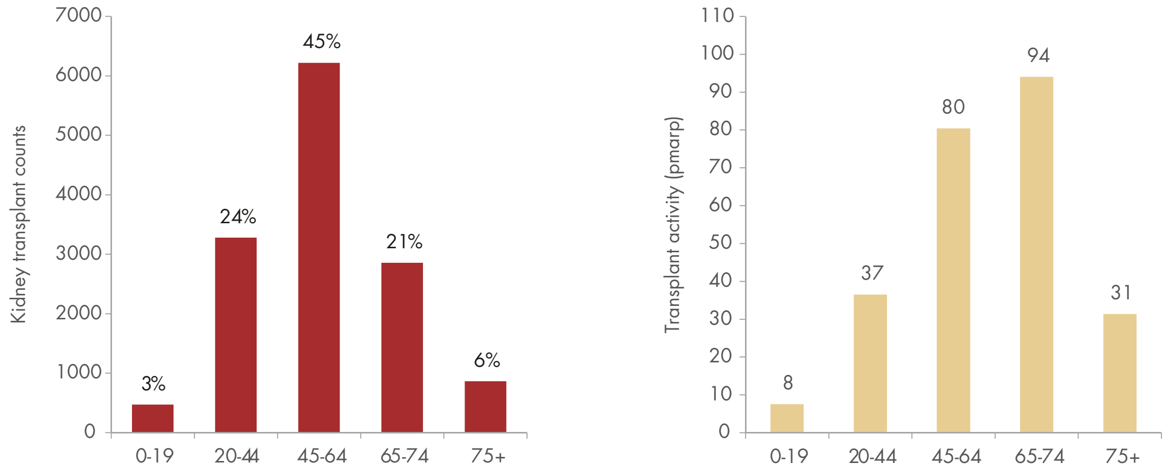

Figure A.7.8

Donor type distribution by age in kidney transplant recipients, unadjusted

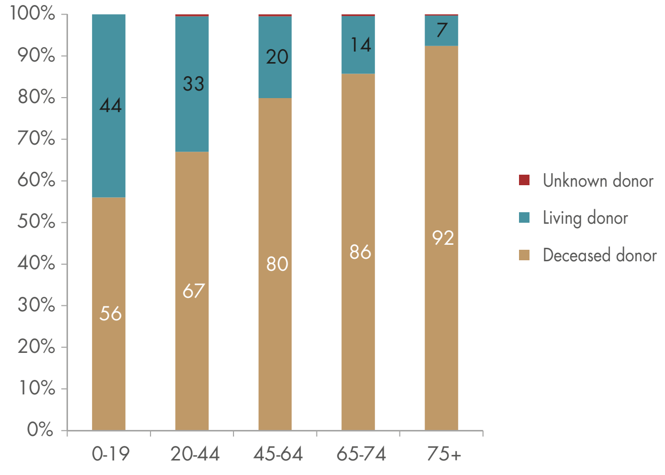

Figure A.7.9

Patient survival probability by age: Incident dialysis patients (cohort 2014-2018)

from day 91, unadjusted

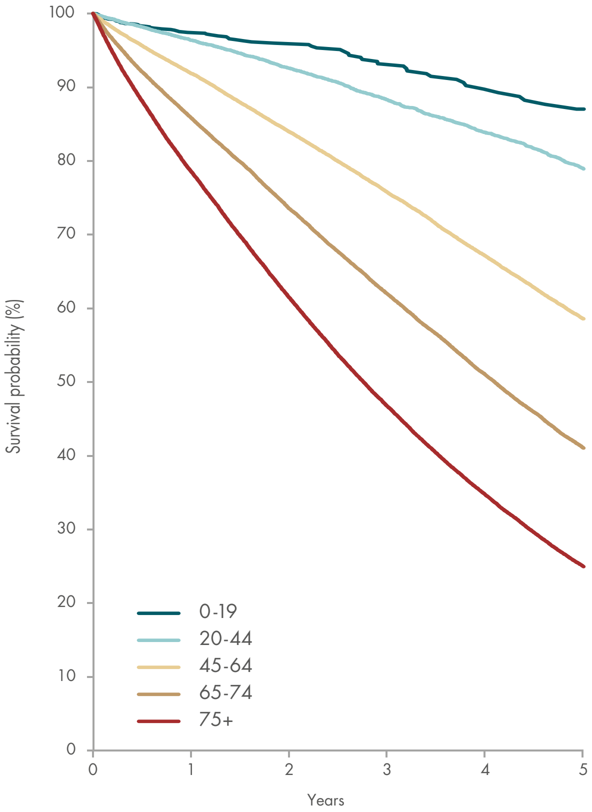

Figure A.7.10

Patient survival probability by age: Patients receiving a fi rst kidney transplant (cohort 2014-2018)

from day of transplant, unadjusted

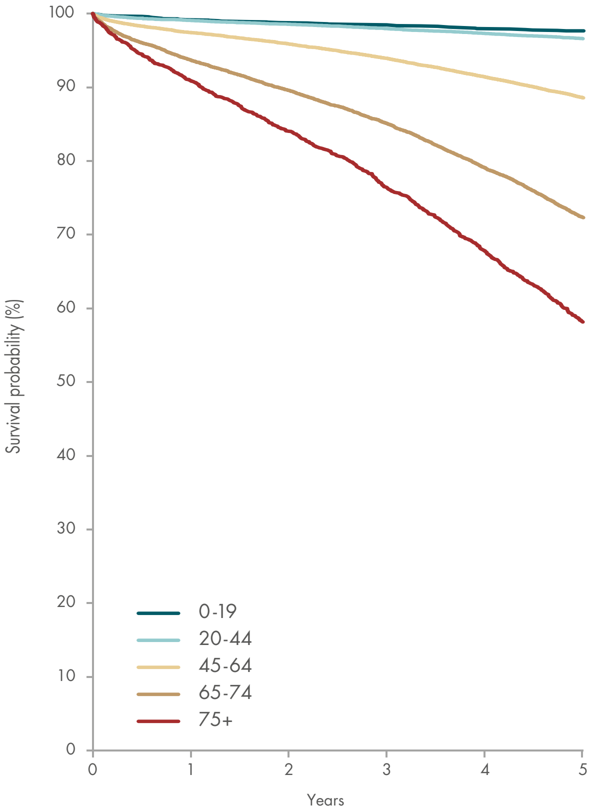

FIND

Section B

Individual patient data reference tables

Table B.1.1

General population data and number of renal centres

	General population of country/region in thousands	% Coverage of general population by the registry for individual patient data	Total number of renal centres in country/region	Number of renal centres collaborating with the registry for individual patient data
Austria §	9105	98.0	73	72
Belgium, Dutch-speaking	6813	100	27	27
Belgium, French-speaking	4967	100	32	32
Bosnia and Herzegovina	3531	100	30	30
Denmark	5947	100	14	14
Estonia	1370	95.0	3	3
France	68372	100	935	935
Greece	10407	100	211	211
Iceland	386	100	1	1
Netherlands &	17877	93 - 97	57	57
Norway	5520	100	28	28
Romania	19061	100	153	153
Serbia	6623	96.5	63	52
Spain, Andalusia	8608	100	63	63
Spain, Aragon	1346	100	10	10
Spain, Basque country	2222	100	10	10
Spain, Canary Islands $	2226	100	20	16
Spain, Cantabria	590	100	2	2
Spain, Castile and León	2384	100	22	22
Spain, Castile-La Mancha	2094	100	15	15
Spain, Catalonia	7902	100	49	49
Spain, Community of Madrid	6872	100	39	39
Spain, Extremadura	1054	100	13	13
Spain, Galicia	2703	100	26	26
Spain, La Rioja	323	100	3	3
Spain, Murcia	1552	100	14	14
Spain, Navarre	675	100	3	3
Spain, Valencian region	5216	100	49	49
Sweden	10537	100	67	67
Switzerland	8889	95.0	109	109
United Kingdom, England	57690	92.0	58	56
United Kingdom, Northern Ireland	1920	100	6	6
United Kingdom, Scotland	5490	100	10	10
United Kingdom, Wales	3164	100	6	6

§ No data from 1 centre treating haemodialysis patients only. It is estimated that most of these patients are covered by other centres
& The percentage coverage is different for the incidence (93%), prevalence (96%) and transplant activity (97%) calculations
$ No data from 4 centres which treat foreign patients only

Table B.2.1

Incident counts and percentages by age and sex

at day 1

	All			0-19						20-44						45-64						65-74						75+					
	All	Male	Female	All		Male		Female		All		Male		Female		All		Male		Female		All		Male		Female		All		Male		Female	
	N (100%)	N (100%)	N (100%)	N	%	N	%	N	%	N	%	N	%	N	%	N	%	N	%	N	%	N	%	N	%	N	%	N	%	N	%	N	%
Austria §	1032	684	348	19	2	11	2	8	2	123	12	78	11	45	13	289	28	201	29	88	25	286	28	182	27	104	30	315	31	212	31	103	30
Belgium, Dutch-speaking *	1075	704	371							73	7	47	7	26	7	219	20	140	20	79	21	295	27	198	28	97	26	488	45	319	45	169	46
Belgium, French-speaking *	932	593	339							71	8	39	7	32	9	226	24	141	24	85	25	302	32	199	34	103	30	333	36	214	36	119	35
Bosnia and Herzegovina	424	257	167	7	2	5	2	2	1	36	8	22	9	14	8	130	31	77	30	53	32	143	34	96	37	47	28	108	25	57	22	51	31
Denmark #	722	472	250	8	1	7	1			68	9	39	8	29	12	238	33	152	32	86	34	201	28	140	30	61	24	207	29	134	28	73	29
Estonia	84	47	37	0	0	0	0	0	0	13	15	8	17	5	14	31	37	17	36	14	38	23	27	12	26	11	30	17	20	10	21	7	19
France	10974	7192	3782	113	1	74	1	39	1	986	9	648	9	338	9	2770	25	1773	25	997	26	3069	28	2058	29	1011	27	4036	37	2639	37	1397	37
Greece	2602	1749	853	9	0	6	0	3	0	123	5	76	4	47	6	605	23	421	24	184	22	688	26	475	27	213	25	1177	45	771	44	406	48
Iceland	50	38	12	0	0	0	0	0	0	7	14	5	13	2	17	17	34	13	34	4	33	10	20	6	16	4	33	16	32	14	37	2	17
Netherlands &	1958	1267	691	36	2	27	2	9	1	230	12	142	11	88	13	636	32	394	31	242	35	596	30	379	30	217	31	460	23	325	26	135	20
Norway	563	389	174	12	2	9	2	3	2	81	14	52	13	29	17	168	30	117	30	51	29	139	25	93	24	46	26	163	29	118	30	45	26
Romania	3702	2190	1512	28	1	14	1	14	1	329	9	200	9	129	9	1325	36	848	39	477	32	1294	35	747	34	547	36	726	20	381	17	345	23
Serbia	519	341	178	9	2	7	2	2	1	52	10	36	11	16	9	199	38	128	38	71	40	179	34	123	36	56	31	80	15	47	14	33	19
Spain, Andalusia	1285	824	461	13	1	7	1	6	1	124	10	81	10	43	9	413	32	252	31	161	35	361	28	240	29	121	26	374	29	244	30	130	28
Spain, Aragon	196	143	53	4	2	2	1	2	4	17	9	9	6	8	15	55	28	38	27	17	32	58	30	45	31	13	25	62	32	49	34	13	25
Spain, Basque country	278	199	79	0	0	0	0	0	0	25	9	15	8	10	13	85	31	56	28	29	37	92	33	68	34	24	30	76	27	60	30	16	20
Spain, Canary Islands	402	270	132	2	0	1	0	1	1	45	11	28	10	17	13	148	37	105	39	43	33	101	25	68	25	33	25	106	26	68	25	38	29
Spain, Cantabria *	100	77	23							7	7	5	6	2	9	19	19	16	21	3	13	32	32	25	32	7	30	42	42	31	40	11	48
Spain, Castile and León *	347	249	98							20	6	14	6	6	6	109	31	78	31	31	32	100	29	72	29	28	29	118	34	85	34	33	34
Spain, Castile-La Mancha *	260	183	77							29	11	14	8	15	19	78	30	59	32	19	25	68	26	49	27	19	25	85	33	61	33	24	31
Spain, Catalonia	1455	1013	442	20	1	16	2	4	1	112	8	75	7	37	8	401	28	275	27	126	29	398	27	279	28	119	27	524	36	368	36	156	35
Spain, Community of Madrid	890	591	287	36	4	23	4	13	5	114	13	67	11	43	15	270	30	185	31	82	29	213	24	139	24	73	25	257	29	177	30	76	26
Spain, Extremadura	167	114	53	0	0	0	0	0	0	14	8	13	11	1	2	46	28	24	21	22	42	53	32	41	36	12	23	54	32	36	32	18	34
Spain, Galicia	423	301	122	0	0	0	0	0	0	32	8	20	7	12	10	127	30	83	28	44	36	122	29	94	31	28	23	142	34	104	35	38	31
Spain, La Rioja	42	26	16	0	0	0	0	0	0	5	12	4	15	1	6	19	45	13	50	6	38	10	24	5	19	5	31	8	19	4	15	4	25
Spain, Murcia	227	143	84	2	1	1	1	1	1	28	12	16	11	12	14	70	31	44	31	26	31	70	31	50	35	20	24	57	25	32	22	25	30
Spain, Navarre *	90	69	21							9	10	8	12	1	5	34	38	21	30	13	62	19	21	17	25	2	10	28	31	23	33	5	24
Spain, Valencian region	758	522	236	9	1	5	1	4	2	65	9	48	9	17	7	215	28	138	26	77	33	212	28	146	28	66	28	257	34	185	35	72	31
Sweden	1142	769	373	18	2	10	1	8	2	115	10	73	9	42	11	357	31	240	31	117	31	281	25	193	25	88	24	371	32	253	33	118	32
Switzerland	752	523	229	9	1	5	1	4	2	84	11	56	11	28	12	201	27	141	27	60	26	184	24	127	24	57	25	274	36	194	37	80	35
United Kingdom, England #	6599	4193	2406	150	2	84	2	66	3	1090	17	660	16	430	18	2376	36	1493	36	883	37	1554	24	984	23	570	24	1429	22	972	23	457	19
United Kingdom, Northern Ireland #	236	142	94							27	11	15	11	12	13	86	36	48	34	38	40	68	29	46	32	22	23	51	22	31	22	20	21
United Kingdom, Scotland	603	385	218	6	1	3	1	3	1	87	14	61	16	26	12	235	39	151	39	84	39	186	31	108	28	78	36	89	15	62	16	27	12
United Kingdom, Wales #	464	278	186							66	14	39	14	27	15	158	34	89	32	69	37	115	25	76	27	39	21	120	26	71	26	49	26

Categories may not add up due to missing values or rounding
§ The incidence is underestimated by approximately 2% due to one haemodialysis centre not submitting data
* Patients younger than 20 years of age are not reported
# Values based on 1 to 5 patients are omitted
& The incidence is underestimated by approximately 7%

Table B.2.2

Incidence per million (age-related) population by age and sex, unadjusted
at day 1

	All			0-19			20-44			45-64			65-74			75+		
	All	Male	Female	All	Male	Female	All	Male	Female	All	Male	Female	All	Male	Female	All	Male	Female
	Pmp	Pmp	Pmp	Pmarp	Pmarp	Pmarp	Pmarp	Pmarp	Pmarp	Pmarp	Pmarp	Pmarp	Pmarp	Pmarp	Pmarp	Pmarp	Pmarp	Pmarp
Austria	115.7	155.6	76.9	11.0	12.4	9.5	42.4	52.7	31.6	113.4	159.2	68.4	319.4	434.8	218.1	370.7	614.6	204.1
Belgium, Dutch-speaking *	157.8	208.5	108.0				34.9	44.6	25.1	121.1	153.7	88.0	396.4	544.7	254.7	695.9	1077.9	417.0
Belgium, French-speaking *	187.7	243.1	134.1				42.8	46.8	38.8	177.3	221.5	133.2	623.3	882.2	397.8	824.3	1363.0	481.8
Bosnia and Herzegovina	120.1	148.4	92.8	8.9	12.4	5.2	29.4	35.3	23.3	127.5	155.0	101.4	486.4	744.6	284.7	519.3	710.5	399.2
Denmark #	121.4	159.7	83.6	6.2	10.6		36.2	40.9	31.3	153.3	196.1	110.6	322.3	464.3	189.4	345.7	510.1	217.2
Estonia	64.5	76.2	54.0	0	0	0	31.3	37.3	24.8	91.4	103.2	80.2	160.3	207.3	128.6	139.8	281.0	81.4
France	160.5	217.0	107.4	7.1	9.1	5.0	48.1	63.9	32.6	159.1	208.0	112.2	402.9	582.6	247.6	582.1	948.5	336.5
Greece	250.0	343.4	160.5	4.7	6.1	3.2	40.7	49.3	31.8	197.4	281.1	117.3	577.2	850.7	336.1	967.7	1518.7	572.9
Iceland	129.6	191.7	64.0	0	0	0	49.3	66.5	30.0	188.2	283.2	90.0	293.2	352.8	233.9	641.9	1219.6	148.7
Netherlands	117.8	153.3	82.6	10.3	15.1	5.3	43.2	52.8	33.4	143.3	178.1	108.7	326.4	423.2	233.3	295.2	475.3	154.3
Norway	102.0	139.9	63.5	9.7	14.1	5.0	44.1	55.4	32.4	118.5	162.2	73.3	254.0	344.0	166.2	342.0	559.4	169.4
Romania	194.2	236.3	154.4	6.8	6.6	7.0	57.0	67.2	46.2	246.8	319.2	175.8	561.4	759.7	413.9	490.3	725.2	361.1
Serbia	81.2	109.7	54.2	7.2	10.9	3.3	26.7	36.5	16.7	111.9	147.7	77.9	199.5	304.0	113.7	151.7	224.4	103.8
Spain, Andalusia	149.3	194.4	105.5	7.5	7.9	7.2	46.3	59.7	32.6	158.0	194.8	121.9	428.1	601.3	272.4	500.5	808.8	291.7
Spain, Aragon	145.6	214.9	77.8	16.2	15.7	16.8	43.6	45.1	42.1	134.0	183.2	83.7	400.3	651.3	171.5	400.2	784.8	140.5
Spain, Basque country	125.1	184.2	69.2	0	0	0	41.0	48.6	33.2	122.7	163.0	83.0	355.6	562.2	174.3	285.0	574.2	98.6
Spain, Canary Islands	180.6	245.7	117.1	5.3	5.2	5.5	61.7	76.9	46.6	203.3	288.2	118.2	466.6	656.3	292.4	602.1	930.2	369.2
Spain, Cantabria *	169.6	269.5	75.7				43.5	61.8	25.0	100.7	172.1	31.4	441.0	729.0	182.9	622.4	1180.5	266.8
Spain, Castile and León *	145.6	212.2	81.0				32.0	43.8	19.7	145.1	206.6	82.9	338.4	492.3	187.6	351.6	622.3	165.8
Spain, Castile-La Mancha *	124.1	174.3	73.7				45.5	42.5	48.8	122.3	181.6	60.7	337.4	496.9	184.6	408.0	709.7	196.1
Spain, Catalonia	184.1	260.6	110.1	12.9	20.0	5.3	44.8	59.2	30.1	172.8	237.1	108.5	516.4	782.7	287.3	690.8	1221.0	341.3
Spain, Community of Madrid	129.5	179.7	80.1	27.0	33.6	20.0	51.2	61.2	38.0	132.1	188.3	77.2	334.0	490.2	206.1	408.7	730.7	196.6
Spain, Extremadura	158.4	218.5	99.5	0	0	0	45.5	82.8	6.6	140.4	145.6	135.2	469.7	736.3	210.0	459.8	762.9	256.2
Spain, Galicia	156.5	231.5	87.0	0	0	0	44.2	55.0	33.4	150.0	200.5	101.8	367.3	603.5	158.7	371.0	685.4	164.5
Spain, La Rioja	129.9	162.9	97.8	0	0	0	54.3	86.1	21.9	191.4	260.2	121.7	286.8	294.0	279.8	221.8	271.3	187.6
Spain, Murcia	146.3	183.8	108.5	5.8	5.6	6.0	55.6	61.6	49.2	155.2	192.8	116.7	525.7	793.3	285.2	473.0	649.6	350.9
Spain, Navarre *	133.3	206.4	61.6				45.2	79.3	10.2	168.5	205.9	130.2	275.8	510.7	56.2	399.3	805.5	120.3
Spain, Valencian region	145.3	203.4	89.0	9.0	9.7	8.2	41.3	60.3	21.8	135.1	173.9	96.5	388.9	571.6	227.8	511.5	902.5	242.1
Sweden	108.4	144.9	71.3	7.4	8.0	6.8	34.0	41.8	25.7	138.9	184.4	92.1	264.4	369.4	162.9	337.8	516.7	193.9
Switzerland	89.1	124.7	53.9	5.3	5.8	4.9	30.1	39.4	20.4	85.9	120.2	51.4	225.1	323.9	134.0	339.1	567.6	171.6
United Kingdom, England #	124.3	161.1	88.9	12.2	13.3	11.0	62.4	76.9	48.4	177.2	226.7	129.4	308.6	406.2	218.2	292.5	456.0	165.9
United Kingdom, Northern Ireland #	122.9	150.2	96.4				45.0	50.4	39.6	173.9	198.3	150.6	372.7	514.6	236.4	318.7	448.5	220.0
United Kingdom, Scotland	109.8	144.3	77.3	5.3	5.2	5.4	49.6	70.9	29.0	158.4	210.0	109.8	308.1	373.6	248.0	173.6	282.8	92.0
United Kingdom, Wales #	146.6	179.0	115.4				68.0	81.0	55.3	193.4	223.8	164.7	327.4	447.1	215.2	362.5	485.8	265.1

* Patients younger than 20 years of age are not reported
# Values based on 1 to 5 patients are omitted

Table B.2.3

Sex, mean age, and median age of incident patients
at day 1

	All						Male						Female					
	%	Mean (years)	SD	Median (years)	P25	P75	%	Mean (years)	SD	Median (years)	P25	P75	%	Mean (years)	SD	Median (years)	P25	P75
Austria	100	64.5	16.9	68.2	56.7	76.7	66.3	64.7	16.7	68.0	57.1	76.8	33.7	64.3	17.4	68.7	56.1	76.1
Belgium, Dutch-speaking *	100	70.8	14.0	73.7	64.0	81.1	65.5	70.8	13.8	73.5	64.4	80.9	34.5	70.8	14.3	73.9	63.0	82.2
Belgium, French-speaking *	100	68.3	14.1	70.7	60.5	78.1	63.6	68.7	13.6	71.6	61.0	78.2	36.4	67.7	14.9	70.1	59.9	77.8
Bosnia and Herzegovina	100	65.1	15.1	68.3	58.9	75.1	60.6	64.8	15.0	68.0	59.2	73.6	39.4	65.6	15.2	68.9	57.4	76.5
Denmark	100	64.5	15.6	67.8	56.6	76.2	65.4	65.0	15.5	68.4	57.7	76.3	34.6	63.5	15.8	65.9	53.5	76.0
Estonia	100	63.5	16.5	64.4	54.2	73.4	56.0	63.9	17.6	64.4	53.9	74.8	44.0	63.1	15.3	64.9	54.2	73.1
France	100	67.3	15.8	71.0	59.1	78.3	65.5	67.4	15.8	71.3	59.5	78.3	34.5	67.2	15.7	70.6	58.5	78.4
Greece	100	70.8	13.6	73.3	63.5	80.7	67.2	70.6	13.3	72.9	63.4	80.3	32.8	71.1	14.3	74.3	63.8	81.7
Iceland	100	64.8	15.3	65.8	55.3	79.0	76.0	65.6	15.8	65.8	55.3	80.5	24.0	62.6	14.2	66.6	54.9	69.7
Netherlands	100	62.8	15.9	66.6	53.6	74.6	64.7	63.4	16.2	67.5	54.6	75.3	35.3	61.8	15.3	65.6	52.2	73.6
Norway	100	62.5	17.7	66.7	51.4	76.2	69.1	62.9	17.9	67.0	52.7	76.2	30.9	61.7	17.3	65.6	48.8	76.4
Romania	100	63.8	14.1	66.4	55.5	73.4	59.2	63.1	13.7	65.5	55.0	72.4	40.8	64.8	14.5	67.8	56.3	74.5
Serbia	100	62.0	14.6	65.0	55.1	72.3	65.7	61.9	14.9	65.0	55.8	72.0	34.3	62.3	14.1	64.9	54.9	72.4
Spain, Andalusia	100	65.1	14.9	68.4	57.0	76.1	64.1	65.3	14.8	68.9	57.6	76.2	35.9	64.6	15.0	67.6	55.9	76.0
Spain, Aragon	100	65.6	15.8	69.5	58.4	76.9	73.0	67.6	14.3	70.6	59.5	77.3	27.0	60.2	18.5	63.9	50.2	74.6
Spain, Basque country	100	66.0	13.8	68.6	58.8	76.0	71.6	67.0	13.3	69.8	59.6	76.6	28.4	63.4	14.6	65.3	54.9	73.1
Spain, Canary Islands	100	63.7	14.7	65.9	53.9	75.5	67.2	63.7	14.4	65.7	54.6	75.2	32.8	63.8	15.2	66.8	52.5	76.4
Spain, Cantabria *	100	70.0	14.2	73.7	64.6	79.6	77.0	69.7	13.8	73.6	64.2	78.5	23.0	71.3	15.7	73.7	66.6	80.6
Spain, Castile and León *	100	67.7	12.9	69.5	60.0	77.4	71.8	67.7	13.4	69.7	60.0	77.9	28.2	67.5	11.7	69.5	60.7	76.9
Spain, Castile-La Mancha *	100	65.9	14.4	67.8	58.0	77.0	70.4	66.9	13.4	67.9	58.6	77.1	29.6	63.3	16.2	66.8	53.2	76.3
Spain, Catalonia	100	66.8	15.4	70.4	58.2	78.0	69.6	66.7	15.6	70.5	58.5	77.8	30.4	66.9	15.1	70.0	57.7	78.6
Spain, Community of Madrid	100	62.0	18.9	66.4	52.7	76.0	66.4	62.9	18.2	66.6	53.7	76.3	32.2	60.5	20.0	65.6	50.8	75.3
Spain, Extremadura	100	68.1	13.9	70.4	59.8	78.2	68.3	67.3	14.1	70.5	60.1	77.8	31.7	70.0	13.4	70.0	58.9	81.4
Spain, Galicia	100	67.0	13.6	70.0	59.2	77.3	71.2	67.7	13.0	70.3	61.5	77.1	28.8	65.2	14.8	68.0	54.0	77.8
Spain, La Rioja	100	61.5	13.6	62.2	51.5	70.7	61.9	59.1	13.6	60.2	51.4	67.2	38.1	65.4	13.2	67.6	58.5	75.1
Spain, Murcia	100	64.1	15.2	67.4	55.4	75.5	63.0	64.5	14.6	67.7	57.1	74.0	37.0	63.5	16.3	66.5	50.1	76.0
Spain, Navarre *	100	64.6	14.6	66.8	55.5	76.2	76.7	65.4	15.4	69.3	55.9	76.3	23.3	62.0	11.6	60.3	54.6	68.1
Spain, Valencian region	100	66.4	15.4	69.4	58.5	77.7	68.9	66.9	15.4	70.1	59.2	78.0	31.1	65.3	15.4	67.5	57.8	77.1
Sweden	100	64.7	16.4	68.4	55.8	77.2	67.3	65.1	16.2	68.9	56.1	77.5	32.7	63.8	16.8	67.3	54.8	76.7
Switzerland	100	66.4	16.4	70.0	57.2	79.0	69.5	66.8	16.2	70.0	57.9	79.2	30.5	65.5	17.1	69.9	56.3	78.2
United Kingdom, England	100	60.2	17.1	62.7	50.0	73.8	63.5	61.0	17.0	63.4	50.8	74.3	36.5	59.0	17.3	61.3	48.7	72.6
United Kingdom, Northern Ireland	100	62.5	15.7	65.2	52.1	74.3	60.2	63.2	15.3	67.3	52.1	74.4	39.8	61.2	16.3	62.8	53.2	73.9
United Kingdom, Scotland	100	60.4	15.1	63.1	52.1	71.7	63.8	60.3	15.3	62.2	52.1	71.5	36.2	60.7	14.7	64.0	52.0	71.7
United Kingdom, Wales	100	62.6	16.8	65.3	52.5	75.3	59.9	63.0	17.1	65.9	52.4	75.5	40.1	62.0	16.3	63.4	52.5	75.1

Categories may not add up due to missing values or rounding
** Patients younger than 20 years of age are not reported*

FIND

Table B.2.4

Incidence per million population, adjusted

at day 1, adjusted for age and sex

	All	Male	Female
	Pmp	Pmp	Pmp
Austria	117.7	158.1	79.1
Belgium, Dutch-speaking *	155.6	202.0	111.3
Belgium, French-speaking *	215.4	281.9	151.9
Bosnia and Herzegovina	158.4	202.1	116.7
Denmark	122.2	160.2	86.0
Estonia	69.6	86.3	53.7
France	163.9	219.2	111.0
Greece	229.3	312.3	149.9
Iceland	160.0	246.3	77.5
Netherlands	117.9	152.1	85.3
Norway	107.6	147.6	69.2
Romania	204.1	255.6	154.8
Serbia	77.5	105.4	50.9
Spain, Andalusia	157.4	205.4	111.4
Spain, Aragon	139.2	204.5	76.9
Spain, Basque country	116.2	169.4	65.3
Spain, Canary Islands	187.0	251.6	125.2
Spain, Cantabria *	155.3	242.9	71.5
Spain, Castile and León *	124.6	178.2	73.4
Spain, Castile-La Mancha *	126.7	178.0	77.7
Spain, Catalonia	194.7	276.2	116.9
Spain, Community of Madrid	137.9	192.9	85.3
Spain, Extremadura	148.7	206.3	93.6
Spain, Galicia	135.9	196.4	78.0
Spain, La Rioja	127.0	157.7	97.7
Spain, Murcia	168.5	215.4	123.5
Spain, Navarre *	130.8	204.3	60.5
Spain, Valencian region	146.9	205.6	90.7
Sweden	110.5	147.1	75.4
Switzerland	91.7	127.9	57.2
United Kingdom, England	133.1	171.1	96.8
United Kingdom, Northern Ireland	135.5	164.9	107.4
United Kingdom, Scotland	110.7	144.1	78.8
United Kingdom, Wales	147.2	178.1	117.7

* Patients younger than 20 years of age are not reported

Table B.2.5.A

Incidence per million population and percentages by primary renal disease (1995 PRD codes), unadjusted

at day 1

	All		GN		PN		PKD		DM						HT		RVD		Misc		Unkn		Missing	
									Type 1		Type 2		Total											
	Pmp	%	Pmp	%	Pmp	%	Pmp	%	Pmp	%	Pmp	%	Pmp	%	Pmp	%	Pmp	%	Pmp	%	Pmp	%	Pmp	%
Austria	115.7	100	10.4	9.0	5.0	4.4	5.9	5.1	3.5	3.0	18.9	16.4	22.4	19.4	16.7	14.4	2.6	2.2	27.9	24.1	24.0	20.7	0.7	0.6
Belgium, Dutch-speaking *	157.8	100	16.0	10.1	6.0	3.8	9.2	5.9	3.8	2.4	29.6	18.8	33.5	21.2	18.5	11.7	7.3	4.7	52.5	33.3	14.7	9.3	0	0
Belgium, French-speaking *	187.7	100	16.1	8.6	6.6	3.5	7.4	4.0	2.6	1.4	33.2	17.7	35.8	19.1	29.2	15.6	1.4	0.8	61.2	32.6	29.8	15.9	0	0
Bosnia and Herzegovina	120.1	100	5.4	4.5	15.9	13.2	5.1	4.2	5.4	4.5	26.3	21.9	31.7	26.4	7.1	5.9	4.2	3.5	21.2	17.7	29.5	24.5	0	0
Denmark #	121.4	100	13.6	11.2	6.4	5.3	10.6	8.7	7.4	6.1	24.0	19.8	31.4	25.9	12.9	10.7	2.0	1.7	20.0	16.5	24.4	20.1	0	0
Estonia	64.5	100	15.4	23.8	3.1	4.8	6.1	9.5					11.5	17.9	18.4	28.6	0	0	10.0	15.5	0	0	0	0
France	160.5	100	16.3	10.2	8.2	5.1	9.5	5.9					36.1	22.5	38.4	23.9	1.6	1.0	24.4	15.2	26.0	16.2	0	0
Greece	250.0	100	18.4	7.3	13.5	5.4	10.9	4.3	2.1	0.8	55.9	22.4	58.0	23.2	23.3	9.3	6.2	2.5	33.2	13.3	86.5	34.6	0	0
Iceland	129.6	100	15.6	12.0	5.2	4.0	13.0	10.0	0	0	15.6	12.0	15.6	12.0	13.0	10.0	49.3	38.0	18.2	14.0	0	0	0	0
Netherlands	117.8	100	12.9	11.0	4.3	3.6	6.6	5.6	4.1	3.5	21.2	18.0	25.3	21.5	18.6	15.8	4.2	3.5	23.8	20.2	21.9	18.6	0.2	0.2
Norway	102.0	100	15.9	15.6	3.6	3.6	8.0	7.8	3.6	3.6	11.4	11.2	15.0	14.7	31.7	31.1	1.4	1.4	23.0	22.6	2.7	2.7	0.5	0.5
Romania	194.2	100	26.3	13.5	6.2	3.2	3.2	1.6					23.0	11.8	13.1	6.8	1.5	0.8	33.6	17.3	87.3	44.9	0	0
Serbia	81.2	100	6.3	7.7	7.8	9.6	3.8	4.6					18.9	23.3	28.6	35.3	0.8	1.0	12.0	14.8	3.0	3.7	0	0
Spain, Andalusia	149.3	100	17.8	11.9	8.0	5.4	9.1	6.1					34.4	23.0	15.6	10.4	2.0	1.3	28.6	19.1	33.9	22.7	0	0
Spain, Aragon	145.6	100	19.3	13.3	8.2	5.6	8.9	6.1	2.2	1.5	28.2	19.4	30.5	20.9	20.1	13.8	0	0	32.7	22.4	26.0	17.9	0	0
Spain, Basque country	125.1	100	10.8	8.6	3.6	2.9	9.9	7.9	5.0	4.0	24.8	19.8	29.7	23.7	17.6	14.0	2.3	1.8	28.8	23.0	22.1	17.6	0.5	0.4
Spain, Canary Islands	180.6	100	18.9	10.4	7.2	4.0	13.9	7.7	8.5	4.7	47.2	26.1	55.7	30.8	11.7	6.5	0.4	0.2	42.2	23.4	30.5	16.9	0	0
Spain, Cantabria *	169.6	100	11.9	7.0	6.8	4.0	10.2	6.0	6.8	4.0	23.7	14.0	30.5	18.0	11.9	7.0	1.7	1.0	22.0	13.0	28.8	17.0	45.8	27.0
Spain, Castile and León * ‡	145.6	100	15.9	11.0	10.1	6.9	10.5	7.2	8.4	5.8	26.8	18.4	35.2	24.2	19.7	13.5	0.4	0.3	34.4	23.6	19.3	13.3	0	0
Spain, Castile-La Mancha *	124.1	100	17.7	14.2	5.3	4.2	11.9	9.6					31.5	25.4	7.2	5.8	0	0	24.8	20.0	25.3	20.4	0.5	0.4
Spain, Catalonia	184.1	100	15.4	8.4	6.8	3.7	10.6	5.8	4.2	2.3	38.1	20.7	42.8	23.2	11.8	6.4	2.7	1.4	14.8	8.0	37.5	20.3	41.8	22.7
Spain, Community of Madrid	129.5	100	12.4	9.6	3.5	2.7	5.8	4.5	3.5	2.7	20.8	16.1	24.3	18.8	7.4	5.7	0	0	18.3	14.2	14.3	11.0	43.5	33.6
Spain, Extremadura	158.4	100	13.3	8.4	9.5	6.0	7.6	4.8					42.7	26.9	7.6	4.8	0	0	41.7	26.3	33.2	21.0	2.8	1.8
Spain, Galicia	156.5	100	15.5	9.9	6.7	4.3	14.4	9.2	4.8	3.1	31.5	20.1	36.3	23.2	15.5	9.9	1.9	1.2	38.5	24.6	27.8	17.7	0	0
Spain, La Rioja	129.9	100	9.3	7.1	3.1	2.4	6.2	4.8	6.2	4.8	24.7	19.0	30.9	23.8	18.6	14.3	0	0	40.2	31.0	21.7	16.7	0	0
Spain, Murcia	146.3	100	26.4	18.1	6.4	4.4	11.0	7.5	2.6	1.8	30.9	21.1	33.5	22.9	11.6	7.9	0	0	30.9	21.1	26.4	18.1	0	0
Spain, Navarre *	133.3	100	19.3	14.4	1.5	1.1	14.8	11.1	1.5	1.1	17.8	13.3	19.3	14.4	23.7	17.8	0	0	29.6	22.2	25.2	18.9	0	0
Spain, Valencian region	145.3	100	18.0	12.4	6.1	4.2	9.8	6.7					29.1	20.1	21.1	14.5	1.5	1.1	32.2	22.2	27.4	18.9	0	0
Sweden ‡	108.4	100	16.1	14.9	4.3	3.9	8.4	7.8	6.8	6.3	20.0	18.5	26.9	24.8	21.8	20.1	0.6	0.5	21.4	19.7	8.9	8.2	0	0
Switzerland	89.1	100	12.2	13.7	1.5	1.7	5.1	5.7	1.9	2.1	13.9	15.6	15.8	17.7	22.7	25.5	2.0	2.3	17.1	19.1	7.7	8.6	5.0	5.6
United Kingdom, England #	124.3	100	12.5	10.0	7.0	5.6	6.6	5.3					33.3	26.8	9.1	7.3	1.0	0.8	21.3	17.1	17.3	13.9	16.3	13.1
United Kingdom, Northern Ireland #	122.9	100	12.5	10.2	4.7	3.8	9.9	8.1					27.1	22.0					34.4	28.0	23.4	19.1	8.9	7.2
United Kingdom, Scotland	109.8	100	16.9	15.4	6.7	6.1	8.7	8.0					32.8	29.9	6.7	6.1	2.4	2.2	21.7	19.7	10.0	9.1	3.8	3.5
United Kingdom, Wales #	146.6	100	21.5	14.7	10.1	6.9	6.6	4.5					41.4	28.2	8.2	5.6			35.4	24.1	22.1	15.1		

Abbreviations used: GN: glomerulonephritis/sclerosis; PN: pyelonephritis; PKD: polycystic kidneys, adult type; DM: diabetes mellitus; HT: hypertension; RVD: renal vascular disease; Misc: miscellaneous; Unkn: unknown
Categories may not add up due to rounding or a limited number of cases (<10%) with diabetes mellitus type unknown; When cells are left empty, (complete) data are unavailable
* Patients younger than 20 years of age are not reported
# Values based on 1 to 5 patients are omitted
‡ Mapping the 2018 PRD codes to the old PRD codes results in a different distribution of PRD groups

Table B.2.5.B

Incidence per million population and percentages by primary renal disease (2012/2018 PRD codes), unadjusted

at day 1

	All		Glomerular disease		Tubulo-interstitial disease		DM		HT / RVD		Other systemic diseases		Familial/hereditary nephropathies		Misc		Unkn		Missing	
	Pmp	%	Pmp	%	Pmp	%	Pmp	%	Pmp	%	Pmp	%	Pmp	%	Pmp	%	Pmp	%	Pmp	%
Austria	115.7	100	14.0	12.1	9.2	7.9	22.4	19.4	23.4	20.3	9.0	7.8	7.5	6.5	10.8	9.3	18.5	16.0	0.9	0.8
Belgium, Dutch-speaking *	157.8	100	20.4	12.9	10.7	6.8	33.5	21.2	35.2	22.3	21.7	13.8	12.6	8.0	15.1	9.6	8.5	5.4	0	0
Belgium, French-speaking *	187.7	100	20.9	11.2	20.1	10.7	35.8	19.1	32.4	17.3	17.1	9.1	12.3	6.5	29.2	15.6	19.7	10.5	0	0
Bosnia and Herzegovina	120.1	100	7.4	6.1	20.7	17.2	31.7	26.4	11.3	9.4	1.7	1.4	9.3	7.8	7.1	5.9	30.9	25.7	0	0
Denmark #	121.4	100	18.2	15.0	9.4	7.8	31.4	25.9	16.1	13.3	3.5	2.9	12.3	10.1	8.2	6.8	22.2	18.3	0	0
Iceland	129.6	100	20.7	16.0	13.0	10.0	15.6	12.0	62.2	48.0	2.6	2.0	15.6	12.0	0	0	0	0	0	0
Netherlands	117.8	100	18.1	15.4	9.0	7.7	25.3	21.5	24.4	20.7	4.2	3.5	9.6	8.1	12.3	10.4	14.8	12.6	0.2	0.2
Norway	102.0	100	20.3	19.9	10.0	9.8	15.0	14.7	33.5	32.9	3.8	3.7	12.1	11.9	4.2	4.1	2.4	2.3	0.7	0.7
Spain, Andalusia	149.3	100	22.4	15.0	14.9	10.0	34.4	23.0	20.4	13.7	6.5	4.4	12.3	8.2	6.0	4.0	32.3	21.6	0	0
Spain, Aragon	145.6	100	22.3	15.3	20.1	13.8	30.5	20.9	23.8	16.3	3.7	2.6	10.4	7.1	22.3	15.3	12.6	8.7	0	0
Spain, Basque country	125.1	100	15.8	12.6	12.2	9.7	29.7	23.7	23.9	19.1	5.0	4.0	12.6	10.1	4.5	3.6	21.2	16.9	0.5	0.4
Spain, Canary Islands	180.6	100	24.3	13.4	15.3	8.5	55.7	30.8	19.8	10.9	11.2	6.2	18.9	10.4	4.9	2.7	30.5	16.9	0	0
Spain, Cantabria *	169.6	100	17.0	10.0	13.6	8.0	30.5	18.0	17.0	10.0	3.4	2.0	13.6	8.0	3.4	2.0	25.4	15.0	45.8	27.0
Spain, Castile and León *	145.6	100	23.1	15.9	21.8	15.0	35.2	24.2	23.1	15.9	6.3	4.3	14.7	10.1	3.4	2.3	18.0	12.4	0	0
Spain, Castile-La Mancha *	124.1	100	22.9	18.5	11.5	9.2	31.5	25.4	10.0	8.1	4.3	3.5	13.8	11.2	5.7	4.6	23.9	19.2	0.5	0.4
Spain, Catalonia	184.1	100	17.3	9.4	9.5	5.2	41.3	22.4	14.6	7.9	5.3	2.9	12.4	6.7	5.8	3.2	36.2	19.7	41.8	22.7
Spain, Community of Madrid	129.5	100	14.6	11.2	9.2	7.1	24.3	18.8	9.6	7.4	2.8	2.1	8.4	6.5	7.6	5.8	9.6	7.4	43.5	33.6
Spain, Extremadura	158.4	100	14.2	9.0	13.3	8.4	42.7	26.9	23.7	15.0	3.8	2.4	15.2	9.6	12.3	7.8	30.4	19.2	2.8	1.8
Spain, Galicia	156.5	100	21.5	13.7	14.4	9.2	36.3	23.2	21.1	13.5	8.1	5.2	19.2	12.3	11.5	7.3	24.4	15.6	0	0
Spain, La Rioja	129.9	100	18.6	14.3	12.4	9.5	30.9	23.8	30.9	23.8	3.1	2.4	6.2	4.8	6.2	4.8	21.7	16.7	0	0
Spain, Murcia	146.3	100	32.2	22.0	12.9	8.8	33.5	22.9	21.3	14.5	1.9	1.3	13.5	9.3	5.2	3.5	25.8	17.6	0	0
Spain, Navarre *	133.3	100	22.2	16.7	10.4	7.8	19.3	14.4	29.6	22.2	7.4	5.6	14.8	11.1	4.4	3.3	25.2	18.9	0	0
Spain, Valencian region	145.3	100	22.0	15.2	14.6	10.0	29.1	20.1	25.7	17.7	9.4	6.5	12.5	8.6	7.7	5.3	24.3	16.8	0	0
Sweden	108.4	100	20.3	18.7	9.8	9.0	26.9	24.8	24.0	22.2	3.5	3.2	10.0	9.2	7.8	7.2	6.2	5.7	0	0
United Kingdom, England #	124.3	100	17.0	13.7	10.2	8.2	33.3	26.7	12.7	10.2	3.4	2.8	8.6	6.9	7.9	6.3	15.1	12.1	16.3	13.1
United Kingdom, Northern Ireland #	122.9	100	15.1	12.3	15.6	12.7	27.1	22.0	7.3	5.9	5.2	4.2	10.9	8.9	16.1	13.1	16.1	13.1	9.4	7.6
United Kingdom, Scotland	109.8	100	20.4	18.6	9.3	8.5	32.8	29.9	13.8	12.6	3.8	3.5	10.9	10.0	4.0	3.6	9.3	8.5	5.5	5.0
United Kingdom, Wales #	146.6	100	27.5	18.8	13.9	9.5	41.4	28.2	19.3	13.1	4.4	3.0	9.8	6.7	13.0	8.8	16.7	11.4		

Abbreviations used: DM: diabetes mellitus; HT / RVD: hypertension / renal vascular disease; Misc: miscellaneous renal disorders; Unkn: unknown
* Patients younger than 20 years of age are not reported
\# Values based on 1 to 5 patients are omitted

Table B.2.6.A

Incidence per million population by primary renal disease (1995 PRD codes), adjusted

at day 1, adjusted for age and sex

	All	GN	PN	PKD	DM			HT	RVD	Misc	Unkn	Missing
					Type 1	Type 2	Total					
	Pmp	Pmp	Pmp	Pmp	Pmp	Pmp	Pmp	Pmp	Pmp	Pmp	Pmp	Pmp
Austria	117.7	10.4	5.2	5.9	3.4	19.4	22.8	17.1	2.6	28.4	24.6	0.7
Belgium, Dutch-speaking *	155.6	16.0	5.9	9.5	3.9	29.2	33.1	18.1	7.1	51.2	14.7	0
Belgium, French-speaking *	215.4	17.7	7.6	8.4	2.8	38.4	41.2	34.7	1.7	70.3	33.9	0
Bosnia and Herzegovina	158.4	6.5	23.4	5.8	6.7	32.6	39.3	8.7	5.1	31.5	38.0	0
Denmark #	122.2	13.7	6.2	11.0	7.9	23.7	31.6	13.1	2.0	20.2	24.4	0
Estonia	69.6	15.7	3.2	6.2			12.8	21.2	0	10.5	0	0
France	163.9	17.0	8.3	10.0			36.8	38.8	1.7	24.9	26.3	0
Greece	229.3	17.4	12.4	10.2	2.0	51.4	53.4	21.2	5.7	30.8	78.2	0
Iceland	160.0	15.2	6.1	15.0	0	18.7	18.7	18.2	66.0	20.7	0	0
Netherlands	117.9	12.9	4.1	7.0	4.2	21.1	25.3	18.7	4.2	23.9	21.8	0.2
Norway	107.6	16.3	3.7	8.6	3.8	12.3	16.1	33.8	1.6	24.2	2.7	0.6
Romania	204.1	27.1	6.6	3.0			23.8	14.3	1.7	35.5	92.1	0
Serbia	77.5	6.1	7.5	3.7			17.9	27.3	0.8	11.5	2.9	0
Spain, Andalusia	157.4	18.0	8.3	8.9			36.4	17.1	2.2	30.1	36.3	0
Spain, Aragon	139.2	18.9	7.5	8.6	2.3	27.0	29.2	19.1	0	31.0	25.0	0
Spain, Basque country	116.2	10.4	3.1	9.4	4.8	22.8	27.5	16.1	1.9	27.0	20.4	0.4
Spain, Canary Islands	187.0	18.9	7.3	12.1	7.4	50.8	58.2	13.0	0.5	45.5	31.5	0
Spain, Cantabria	155.3	11.4	6.6	9.2	6.8	20.9	27.7	10.1	1.8	20.3	25.1	42.8
Spain, Castile and León * ‡	124.6	13.7	8.5	9.4	8.0	22.0	30.0	16.0	0.3	30.1	16.4	0
Spain, Castile-La Mancha *	126.7	17.2	5.4	11.9			32.4	6.8	0	25.9	26.6	0.6
Spain, Catalonia	194.7	15.8	7.2	10.7	3.9	40.9	45.3	12.6	2.9	15.9	40.1	44.3
Spain, Community of Madrid	137.9	12.6	3.7	6.1	3.5	22.9	26.4	8.4	0	19.7	15.6	45.3
Spain, Extremadura	148.7	13.0	8.9	7.0			40.5	7.5	0	38.8	30.3	2.5
Spain, Galicia	135.9	13.8	6.1	13.0	4.5	26.6	31.1	13.3	1.4	33.7	23.4	0
Spain, La Rioja	127.0	8.6	3.3	6.1	6.5	23.2	29.7	17.7	0	41.1	20.6	0
Spain, Murcia	168.5	29.0	7.3	11.4	2.4	37.6	40.0	13.8	0	34.1	32.7	0
Spain, Navarre	130.8	19.0	1.4	14.6	1.3	17.7	19.1	23.3	0	29.1	24.3	0
Spain, Valencian region	146.9	17.9	6.3	9.4			29.5	21.5	1.6	32.6	28.0	0
Sweden ‡	110.5	16.7	4.2	9.1	7.2	20.4	27.6	21.6	0.6	21.8	8.8	0
Switzerland	91.7	12.2	1.6	5.3	1.8	14.6	16.4	23.6	2.1	17.6	8.0	4.9
United Kingdom, England #	133.1	13.2	7.3	7.2			36.2	9.8	1.1	22.5	18.5	17.2
United Kingdom, Northern Ireland #	135.5	13.5	4.8	11.1			29.2			38.3	26.7	9.5
United Kingdom, Scotland	110.7	17.1	6.7	9.0			32.7	6.8	2.4	21.7	10.3	3.9
United Kingdom, Wales #	147.2	22.1	10.0	7.1			41.4	8.1		34.4	22.9	

Abbreviations used: GN: glomerulonephritis/sclerosis; PN: pyelonephritis; PKD: polycystic kidneys, adult type; DM: diabetes mellitus; HT: hypertension; RVD: renal vascular disease; Misc: miscellaneous; Unkn: unknown
Categories may not add up due to rounding or a limited number of cases (<10%) with diabetes mellitus type unknown; When cells are left empty, (complete) data are unavailable
* Patients younger than 20 years of age are not reported
# Values based on 1 to 5 patients are omitted
‡ Mapping the 2018 PRD codes to the old PRD codes results in a different distribution of PRD groups

Table B.2.6.B

Incidence per million population by primary renal disease (2012/2018 PRD codes), adjusted

at day 1, adjusted for age and sex

	All	Glomerular disease	Tubulo-interstitial disease	DM	HT / RVD	Other systemic diseases	Familial/ hereditary nephropathies	Misc	Unkn	Missing
	Pmp	Pmp	Pmp	Pmp	Pmp	Pmp	Pmp	Pmp	Pmp	Pmp
Austria	117.7	14.0	9.4	22.8	23.9	9.2	7.5	11.0	19.0	0.9
Belgium, Dutch-speaking *	155.6	20.3	10.6	33.1	34.1	21.2	12.9	14.9	8.6	0
Belgium, French-speaking *	215.4	23.0	22.6	41.2	38.7	20.1	13.8	34.0	22.2	0
Bosnia and Herzegovina	158.4	8.8	31.4	39.3	13.8	2.2	10.7	10.7	41.6	0
Denmark #	122.2	18.4	9.2	31.6	16.3	3.5	12.7	8.1	22.4	0
Iceland	160.0	21.5	15.8	18.7	84.2	3.0	16.8	0	0	0
Netherlands	117.9	18.1	8.8	25.3	24.4	4.1	10.0	12.4	14.7	0.2
Norway	107.6	20.8	10.4	16.1	35.8	4.0	12.8	4.6	2.4	0.7
Spain, Andalusia	157.4	22.8	15.5	36.4	22.5	6.9	12.3	6.4	34.6	0
Spain, Aragon	139.2	22.0	19.1	29.2	22.6	3.0	10.1	21.1	12.1	0
Spain, Basque country	116.2	15.3	10.9	27.5	21.7	4.7	12.0	4.1	19.5	0.4
Spain, Canary Islands	187.0	24.6	15.8	58.2	22.3	11.9	17.0	5.6	31.5	0
Spain, Cantabria	155.3	16.2	12.7	27.7	15.0	3.0	12.7	3.1	22.0	42.8
Spain, Castile and León	124.6	20.2	18.7	30.0	18.7	5.5	13.3	2.8	15.3	0
Spain, Castile-La Mancha	126.7	22.5	12.0	32.4	9.9	4.5	13.9	5.8	25.1	0.6
Spain, Catalonia	194.7	17.7	10.2	43.7	15.6	5.9	12.5	6.1	38.8	44.3
Spain, Community of Madrid	137.9	14.8	9.8	26.4	10.9	2.9	8.9	8.6	10.3	45.3
Spain, Extremadura	148.7	14.0	12.5	40.5	22.3	3.6	14.5	11.1	27.6	2.5
Spain, Galicia	135.9	18.9	13.3	31.1	17.8	7.1	17.6	9.8	20.4	0
Spain, La Rioja	127.0	17.3	12.7	29.7	30.8	3.0	6.1	6.8	20.6	0
Spain, Murcia	168.5	34.6	13.9	40.0	26.4	2.2	13.8	5.4	32.1	0
Spain, Navarre	130.8	21.7	10.1	19.1	29.0	7.4	14.6	4.5	24.3	0
Spain, Valencian region	146.9	22.0	14.7	29.5	26.3	9.7	12.0	7.9	24.8	0
Sweden	110.5	21.1	9.8	27.6	23.8	3.4	10.7	8.0	6.0	0
United Kingdom, England #	133.1	17.9	10.7	36.2	13.8	3.7	9.2	8.4	16.1	17.2
United Kingdom, Northern Ireland #	135.5	16.4	17.0	29.2	8.4	5.7	12.2	18.2	18.3	10.0
United Kingdom, Scotland	110.7	20.7	9.1	32.7	13.9	3.9	11.2	4.0	9.6	5.6
United Kingdom, Wales #	147.2	28.1	13.7	41.4	18.3	4.4	10.2	13.2	17.3	

Abbreviations used: DM: diabetes mellitus; HT / RVD: hypertension / renal vascular disease; Misc: miscellaneous renal disorders; Unkn: unknown
* Patients younger than 20 years of age are not reported
# Values based on 1 to 5 patients are omitted

Table B.2.7

Incident counts by treatment modality

at day 1

	All	Haemodialysis						Peritoneal dialysis				Preemptive kidney transplant				Unkn	Missing
		HD hospital/ centre	HD home	HD type Unkn	HF	HDF	Total HD/ HF/HDF	APD	CAPD	PD type Unkn	Total PD	Living donor	Deceased donor	Tx type Unkn	Total Tx		
	N	N	N	N	N	N	N	N	N	N	N	N	N	N	N	N	N
Austria §	1032	795	0	1	8	109	913	29	46	0	75	28	16	0	44	0	0
Belgium, Dutch-speaking *	1075	913	1	63			977			85	85	8	5	0	13	0	0
Belgium, French-speaking *	932	497	17	0	57	259	830	43	38	0	81	8	11	0	19	0	2
Bosnia and Herzegovina	424	376	1	1	0	32	410	0	12	0	12	1	0	0	1	1	0
Denmark #	722	449	0	0			449	64	110	10	184	33	56	0	89	0	0
Estonia	84	70	0	0	0	0	70	13	0	0	13	1	0	0	1	0	0
France	10974	7210	3	16	28	2222	9479	205	861	6	1072	187	236	0	423	0	0
Greece	2602	2467	0	0	0	24	2491	14	75	0	89	22	0	0	22	0	0
Iceland	50	35	0	0			35	0	9	0	9	0	6	0	6	0	0
Netherlands &	1958	1322	2	0		7	1331	150	200	0	350	234	41	0	275	2	0
Norway	563	312	0	0			312			197	197	20	34	0	54	0	0
Romania ||	3702	3664	0	0	0	2	3666	1	27	0	28	5	3	0	8	0	0
Serbia	519	430	0	1	0	37	468	4	42	0	46	4	0	0	4	1	0
Spain, Andalusia	1285	1019	6	0			1025			183	183	29	48	0	77	0	0
Spain, Aragon	196	124	1	0		29	154	10	21	0	31	5	6	0	11	0	0
Spain, Basque country	278	195	1	0			196	4	67	0	71	7	4	0	11	0	0
Spain, Canary Islands	402	291	1	0	0	0	292	0	0	100	100	6	4	0	10	0	0
Spain, Cantabria *	100	75	0	0			75	0	14	0	14	1	10	0	11	0	0
Spain, Castile and León *	347	269	2	0			271	13	53	0	66	7	3	0	10	0	0
Spain, Castile-La Mancha *	260	204	1	0			205	7	45	0	52	1	1	1	3	0	0
Spain, Catalonia	1455	752	16	0		314	1082	31	160	0	191	89	93	0	182	0	0
Spain, Community of Madrid	890	688	6	0		0	694	9	125	0	134	34	28	0	62	0	0
Spain, Extremadura	167	144	4	2			150	4	9	1	14	1	2	0	3	0	0
Spain, Galicia	423	319	6	0	0	0	325	17	66	0	83	12	3	0	15	0	0
Spain, La Rioja	42	30	0	0	0	0	30	5	7	0	12	0	0	0	0	0	0
Spain, Murcia	227	178	0	0	0	4	182	0	37	0	37	5	3	0	8	0	0
Spain, Navarre *	90	65	3	0			68	20	1	0	21	0	1	0	1	0	0
Spain, Valencian region	758	535	9	0			544	14	136	0	150	8	56	0	64	0	0
Sweden	1142	648	4	0			652			380	380	40	70	0	110	0	0
Switzerland	752	598	4	0			602	21	63	0	84	31	16	0	47	19	0
United Kingdom, England #	6599	4246	15	0	11	690	4962	550	629	30	1209	240	184		428	0	0
United Kingdom, Northern Ireland #	236	159	0	0			162	26			29	28	17	0	45	0	0
United Kingdom, Scotland	603	467	0	0			467	37	45	8	90	34	11	1	46	0	0
United Kingdom, Wales #	464	331		0	0	40	374	22	44	0	66	15	9	0	24	0	0

Abbreviations used: HD: haemodialysis; Unkn: unknown; HF: haemofiltration; HDF: haemodiafiltration; APD: automated peritoneal dialysis; CAPD: continuous ambulatory peritoneal dialysis; PD: peritoneal dialysis; Tx: transplant
When cells are left empty, (complete) data are unavailable
§ The incidence is underestimated by approximately 2% due to an estimated 2% underestimation of the incidence of haemodialysis
* Patients younger than 20 years of age are not reported
# Values based on 1 to 5 patients are omitted
& The incidence is underestimated by approximately 7%
|| The incidence of preemptive transplantation is underestimated by approximately 30%

Table B.2.8

Incidence per million population by treatment modality, unadjusted

at day 1

	All	Haemodialysis						Peritoneal dialysis				Preemptive kidney transplant				Unkn	Missing
		HD hospital/ centre	HD home	HD type Unkn	HF	HDF	Total HD/ HF/HDF	APD	CAPD	PD type Unkn	Total PD	Living donor	Deceased donor	Tx type Unkn	Total Tx		
	Pmp	Pmp	Pmp	Pmp	Pmp	Pmp	Pmp	Pmp	Pmp	Pmp	Pmp	Pmp	Pmp	Pmp	Pmp	Pmp	Pmp
Austria	115.7	89.1	0	0.1	0.9	12.2	102.3	3.3	5.2	0	8.4	3.1	1.8	0	4.9	0	0
Belgium, Dutch-speaking*	157.8	134.0	0.1	9.2			143.4			12.5	12.5	1.2	0.7	0	1.9	0	0
Belgium, French-speaking*	187.7	100.1	3.4	0	11.5	52.1	167.1	8.7	7.7	0	16.3	1.6	2.2	0	3.8	0	0.4
Bosnia and Herzegovina	120.1	106.5	0.3	0.3	0	9.1	116.1	0	3.4	0	3.4	0.3	0	0	0.3	0.3	0
Denmark #	121.4	75.5	0	0			75.5	10.8	18.5	1.7	30.9	5.5	9.4	0	15.0	0	0
Estonia	64.5	53.8	0	0	0	0	53.8	10.0	0	0	10.0	0.8	0	0	0.8	0	0
France	160.5	105.5	0	0.2	0.4	32.5	138.6	3.0	12.6	0.1	15.7	2.7	3.5	0	6.2	0	0
Greece	250.0	237.0	0	0	0	2.3	239.4	1.3	7.2	0	8.6	2.1	0	0	2.1	0	0
Iceland	129.6	90.8	0	0			90.8	0	23.3	0	23.3	0	15.6	0	15.6	0	0
Netherlands	117.8	79.5	0.1	0		0.4	80.1	9.0	12.0	0	21.1	14.1	2.5	0	16.5	0.1	0
Norway	102.0	56.5	0	0			56.5			35.7	35.7	3.6	6.2	0	9.8	0	0
Romania||	194.2	192.2	0	0	0	0.1	192.3	0.1	1.4	0	1.5	0.3	0.2	0	0.4	0	0
Serbia	81.2	67.3	0	0.2	0	5.8	73.2	0.6	6.6	0	7.2	0.6	0	0	0.6	0.2	0
Spain, Andalusia	149.3	118.4	0.7	0			119.1			21.3	21.3	3.4	5.6	0	8.9	0	0
Spain, Aragon	145.6	92.1	0.7	0		21.5	114.4	7.4	15.6	0	23.0	3.7	4.5	0	8.2	0	0
Spain, Basque country	125.1	87.8	0.5	0			88.2	1.8	30.2	0	32.0	3.2	1.8	0	5.0	0	0
Spain, Canary Islands	180.6	130.7	0.4	0	0	0	131.2	0	0	44.9	44.9	2.7	1.8	0	4.5	0	0
Spain, Cantabria*	169.6	127.2	0	0			127.2	0	23.7	0	23.7	1.7	17.0	0	18.7	0	0
Spain, Castile and León*	145.6	112.8	0.8	0			113.7	5.5	22.2	0	27.7	2.9	1.3	0	4.2	0	0
Spain, Castile-La Mancha*	124.1	97.4	0.5	0			97.9	3.3	21.5	0	24.8	0.5	0.5	0.5	1.4	0	0
Spain, Catalonia	184.1	95.2	2.0	0		39.7	136.9	3.9	20.2	0	24.2	11.3	11.8	0	23.0	0	0
Spain, Community of Madrid	129.5	100.1	0.9	0		0	101.0	1.3	18.2	0	19.5	4.9	4.1	0	9.0	0	0
Spain, Extremadura	158.4	136.6	3.8	1.9			142.3	3.8	8.5	0.9	13.3	0.9	1.9	0	2.8	0	0
Spain, Galicia	156.5	118.0	2.2	0	0	0	120.3	6.3	24.4	0	30.7	4.4	1.1	0	5.6	0	0
Spain, La Rioja	129.9	92.8	0	0	0	0	92.8	15.5	21.7	0	37.1	0	0	0	0	0	0
Spain, Murcia	146.3	114.7	0	0	0	2.6	117.3	0	23.8	0	23.8	3.2	1.9	0	5.2	0	0
Spain, Navarre*	133.3	96.3	4.4	0			100.7	29.6	1.5	0	31.1	0	1.5	0	1.5	0	0
Spain, Valencian region	145.3	102.6	1.7	0			104.3	2.7	26.1	0	28.8	1.5	10.7	0	12.3	0	0
Sweden	108.4	61.5	0.4	0			61.9			36.1	36.1	3.8	6.6	0	10.4	0	0
Switzerland	89.1	70.8	0.5	0			71.3	2.5	7.5	0	9.9	3.7	1.9	0	5.6	2.3	0
United Kingdom, England #	124.3	80.0	0.3	0	0.2	13.0	93.5	10.4	11.9	0.6	22.8	4.5	3.5		8.1	0	0
United Kingdom, Northern Ireland #	122.9	82.8	0	0			84.4	13.5			15.1	14.6	8.9	0	23.4	0	0
United Kingdom, Scotland	109.8	85.1	0	0			85.1	6.7	8.2	1.5	16.4	6.2	2.0	0.2	8.4	0	0
United Kingdom, Wales #	146.6	104.6		0	0	12.6	118.2	7.0	13.9	0	20.9	4.7	2.8	0	7.6	0	0

Abbreviations used: HD: haemodialysis; Unkn: unknown; HF: haemofiltration; HDF: haemodiafiltration; APD: automated peritoneal dialysis; CAPD: continuous ambulatory peritoneal dialysis; PD: peritoneal dialysis; Tx: transplant
Categories may not add up due to rounding; When cells are left empty, (complete) data are unavailable
* Patients younger than 20 years of age are not reported
# Values based on 1 to 5 patients are omitted
|| The incidence of preemptive transplantation is underestimated by approximately 30%

Table B.2.9

Incidence per million population by treatment modality, adjusted

at day 1, adjusted for age and sex

	All	Haemodialysis						Peritoneal dialysis				Preemptive kidney transplant				Unkn	Missing
		HD hospital/ centre	HD home	HD type Unkn	HF	HDF	Total HD/ HF/HDF	APD	CAPD	PD type Unkn	Total PD	Living donor	Deceased donor	Tx type Unkn	Total Tx		
	Pmp	Pmp	Pmp	Pmp	Pmp	Pmp	Pmp	Pmp	Pmp	Pmp	Pmp	Pmp	Pmp	Pmp	Pmp	Pmp	Pmp
Austria	117.7	90.9	0	0.1	0.9	12.5	104.5	3.2	5.0	0	8.3	3.1	1.8	0	4.9	0	0
Belgium, Dutch-speaking *	155.6	132.0	0.2	9.1			141.3			12.4	12.4	1.2	0.8	0	1.9	0	0
Belgium, French-speaking *	215.4	115.3	3.9	0	13.0	59.9	192.1	9.9	8.8	0	18.7	1.7	2.5	0	4.2	0	0.5
Bosnia and Herzegovina	158.4	142.8	0.3	1.1	0	10.3	154.4	0	3.5	0	3.5	0.2	0	0	0.2	0.3	0
Denmark #	122.2	75.8	0	0			75.8	10.9	18.3	1.7	30.9	5.6	9.9	0	15.5	0	0
Estonia	69.6	58.6	0	0	0	0	58.6	10.3	0	0	10.3	0.7	0	0	0.7	0	0
France	163.9	107.7	0	0.2	0.4	33.0	141.4	3.0	12.9	0.1	16.0	2.9	3.7	0	6.5	0	0
Greece	229.3	217.0	0	0	0	2.1	219.2	1.2	6.7	0	8.0	2.2	0	0	2.2	0	0
Iceland	160.0	112.4	0	0			112.4	0	29.5	0	29.5	0	18.1	0	18.1	0	0
Netherlands	117.9	79.5	0.1	0		0.4	80.0	9.0	12.1	0	21.1	14.2	2.5	0	16.7	0.1	0
Norway	107.6	59.7	0	0			59.7			38.0	38.0	3.6	6.2	0	9.9	0	0
Romania | |	204.1	202.1	0	0	0	0.1	202.2	0	1.4	0	1.5	0.3	0.1	0	0.4	0	0
Serbia	77.5	64.2	0	0.1	0	5.5	69.9	0.6	6.1	0	6.8	0.6	0	0	0.6	0.2	0
Spain, Andalusia	157.4	125.7	0.7	0			126.4			22.1	22.1	3.3	5.6	0	8.9	0	0
Spain, Aragon	139.2	87.4	0.7	0		20.9	109.0	7.3	14.7	0	21.9	3.6	4.7	0	8.3	0	0
Spain, Basque country	116.2	81.7	0.4	0			82.1	1.8	27.8	0	29.5	2.9	1.7	0	4.5	0	0
Spain, Canary Islands	187.0	136.1	0.4	0	0	0	136.4	0	0	46.4	46.4	2.5	1.7	0	4.2	0	0
Spain, Cantabria *	155.3	115.8	0	0			115.8	0	22.3	0	22.3	2.0	15.2	0	17.1	0	0
Spain, Castile and León *	124.6	95.2	0.7	0			95.9	5.2	19.7	0	24.9	2.7	1.0	0	3.7	0	0
Spain, Castile-La Mancha *	126.7	100.0	0.5	0			100.5	3.2	21.6	0	24.8	0.5	0.5	0.5	1.4	0	0
Spain, Catalonia	194.7	101.3	2.2	0		41.9	145.3	4.2	21.5	0	25.7	11.5	12.2	0	23.7	0	0
Spain, Community of Madrid	137.9	107.1	0.8	0		0	107.9	1.2	19.4	0	20.7	5.0	4.3	0	9.3	0	0
Spain, Extremadura	148.7	127.8	3.5	1.7			133.0	3.8	8.5	0.8	13.2	0.9	1.6	0	2.5	0	0
Spain, Galicia	135.9	100.7	2.0	0	0	0	102.7	5.8	21.8	0	27.7	4.4	1.1	0	5.5	0	0
Spain, La Rioja	127.0	91.1	0	0	0	0	91.1	15.0	21.0	0	36.0	0	0	0	0	0	0
Spain, Murcia	168.5	133.6	0	0	0	2.6	136.2	0	26.8	0	26.8	3.2	2.2	0	5.5	0	0
Spain, Navarre *	130.8	93.5	4.7	0			98.3	29.8	1.3	0	31.1	0	1.4	0	1.4	0	0
Spain, Valencian region	146.9	104.2	1.8	0			106.0	2.6	26.3	0	28.9	1.5	10.5	0	12.0	0	0
Sweden	110.5	62.5	0.4	0			62.9			36.3	36.3	4.0	7.2	0	11.2	0	0
Switzerland	91.7	73.4	0.5	0			73.9	2.5	7.6	0	10.1	3.6	1.9	0	5.5	2.3	0
United Kingdom, England #	133.1	86.0	0.3	0	0.2	14.0	100.6	10.9	12.7	0.6	24.1	4.6	3.7		8.4	0	0
United Kingdom, Northern Ireland #	135.5	92.0	0	0			93.6	15.0			16.7	15.4	9.8	0	25.2	0	0
United Kingdom, Scotland	110.7	85.7	0	0			85.7	6.6	8.1	1.4	16.2	6.5	2.1	0.2	8.8	0	0
United Kingdom, Wales #	147.2	104.2		0	0	13.0	118.0	7.1	14.1	0	21.2	4.9	3.1	0	8.0	0	0

Abbreviations used: HD: haemodialysis; Unkn: unknown; HF: haemofiltration; HDF: haemodiafiltration; APD: automated peritoneal dialysis; CAPD: continuous ambulatory peritoneal dialysis; PD: peritoneal dialysis; Tx: transplant
Categories may not add up due to rounding; When cells are left empty, (complete) data are unavailable
* Patients younger than 20 years of age are not reported
# Values based on 1 to 5 patients are omitted
| | The incidence of preemptive transplantation is underestimated by approximately 30%

Table B.2.10

Treatment modality distribution, unadjusted

at day 1

	All	Haemodialysis						Peritoneal dialysis				Preemptive kidney transplant				Unkn	Missing
		HD hospital/ centre	HD home	HD type Unkn	HF	HDF	Total HD/ HF/HDF	APD	CAPD	PD type Unkn	Total PD	Living donor	Deceased donor	Tx type Unkn	Total Tx		
	%	%	%	%	%	%	%	%	%	%	%	%	%	%	%	%	%
Austria	100	77.0	0	0.1	0.8	10.6	88.5	2.8	4.5	0	7.3	2.7	1.6	0	4.3	0	0
Belgium, Dutch-speaking *	100	84.9	0.1	5.9			90.9			7.9	7.9	0.7	0.5	0	1.2	0	0
Belgium, French-speaking *	100	53.3	1.8	0	6.1	27.8	89.1	4.6	4.1	0	8.7	0.9	1.2	0	2.0	0	0.2
Bosnia and Herzegovina	100	88.7	0.2	0.2	0	7.5	96.7	0	2.8	0	2.8	0.2	0	0	0.2	0.2	0
Denmark #	100	62.2	0	0			62.2	8.9	15.2	1.4	25.5	4.6	7.8	0	12.3	0	0
Estonia	100	83.3	0	0	0	0	83.3	15.5	0	0	15.5	1.2	0	0	1.2	0	0
France	100	65.7	0	0.1	0.3	20.2	86.4	1.9	7.8	0.1	9.8	1.7	2.2	0	3.9	0	0
Greece	100	94.8	0	0	0	0.9	95.7	0.5	2.9	0	3.4	0.8	0	0	0.8	0	0
Iceland	100	70.0	0	0			70.0	0	18.0	0	18.0	0	12.0	0	12.0	0	0
Netherlands	100	67.5	0.1	0		0.4	68.0	7.7	10.2	0	17.9	12.0	2.1	0	14.0	0.1	0
Norway	100	55.4	0	0			55.4			35.0	35.0	3.6	6.0	0	9.6	0	0
Romania ||	100	99.0	0	0	0	0.1	99.0	0	0.7	0	0.8	0.1	0.1	0	0.2	0	0
Serbia	100	82.9	0	0.2	0	7.1	90.2	0.8	8.1	0	8.9	0.8	0	0	0.8	0.2	0
Spain, Andalusia	100	79.3	0.5	0			79.8			14.2	14.2	2.3	3.7	0	6.0	0	0
Spain, Aragon	100	63.3	0.5	0		14.8	78.6	5.1	10.7	0	15.8	2.6	3.1	0	5.6	0	0
Spain, Basque country	100	70.1	0.4	0			70.5	1.4	24.1	0	25.5	2.5	1.4	0	4.0	0	0
Spain, Canary Islands	100	72.4	0.2	0	0	0	72.6	0	0	24.9	24.9	1.5	1.0	0	2.5	0	0
Spain, Cantabria *	100	75.0	0	0			75.0	0	14.0	0	14.0	1.0	10.0	0	11.0	0	0
Spain, Castile and León *	100	77.5	0.6	0			78.1	3.7	15.3	0	19.0	2.0	0.9	0	2.9	0	0
Spain, Castile-La Mancha *	100	78.5	0.4	0			78.8	2.7	17.3	0	20.0	0.4	0.4	0.4	1.2	0	0
Spain, Catalonia	100	51.7	1.1	0		21.6	74.4	2.1	11.0	0	13.1	6.1	6.4	0	12.5	0	0
Spain, Community of Madrid	100	77.3	0.7	0		0	78.0	1.0	14.0	0	15.1	3.8	3.1	0	7.0	0	0
Spain, Extremadura	100	86.2	2.4	1.2			89.8	2.4	5.4	0.6	8.4	0.6	1.2	0	1.8	0	0
Spain, Galicia	100	75.4	1.4	0	0	0	76.8	4.0	15.6	0	19.6	2.8	0.7	0	3.5	0	0
Spain, La Rioja	100	71.4	0	0	0	0	71.4	11.9	16.7	0	28.6	0	0	0	0	0	0
Spain, Murcia	100	78.4	0	0	0	1.8	80.2	0	16.3	0	16.3	2.2	1.3	0	3.5	0	0
Spain, Navarre *	100	72.2	3.3	0			75.6	22.2	1.1	0	23.3	0	1.1	0	1.1	0	0
Spain, Valencian region	100	70.6	1.2	0			71.8	1.8	17.9	0	19.8	1.1	7.4	0	8.4	0	0
Sweden	100	56.7	0.4	0			57.1			33.3	33.3	3.5	6.1	0	9.6	0	0
Switzerland	100	79.5	0.5	0			80.1	2.8	8.4	0	11.2	4.1	2.1	0	6.3	2.5	0
United Kingdom, England #	100	64.3	0.2	0	0.2	10.5	75.2	8.3	9.5	0.5	18.3	3.6	2.8		6.5	0	0
United Kingdom, Northern Ireland #	100	67.4	0	0			68.6	11.0			12.3	11.9	7.2	0	19.1	0	0
United Kingdom, Scotland	100	77.4	0	0			77.4	6.1	7.5	1.3	14.9	5.6	1.8	0.2	7.6	0	0
United Kingdom, Wales #	100	71.3		0	0	8.6	80.6	4.7	9.5	0	14.2	3.2	1.9	0	5.2	0	0

Abbreviations used: HD: haemodialysis; Unkn: unknown; HF: haemofiltration; HDF: haemodiafiltration; APD: automated peritoneal dialysis; CAPD: continuous ambulatory peritoneal dialysis; PD: peritoneal dialysis; Tx: transplant
Categories may not add up due to rounding; When cells are left empty, (complete) data are unavailable
* Patients younger than 20 years of age are not reported
# Values based on 1 to 5 patients are omitted
|| The incidence of preemptive transplantation is underestimated by approximately 30%

Table B.2.11

Treatment modality distribution by age, sex, and primary renal disease, unadjusted

at day 1

	All				0-19				20-44				45-64				65-74				75+				Male				Female				DM				Non-DM			
	HD	PD	Tx	Un	HD	PD	Tx	Un	HD	PD	Tx	Un	HD	PD	Tx	Un	HD	PD	Tx	Un	HD	PD	Tx	Un	HD	PD	Tx	Un	HD	PD	Tx	Un	HD	PD	Tx	Un	HD	PD	Tx	Un
	%	%	%	%	%	%	%	%	%	%	%	%	%	%	%	%	%	%	%	%	%	%	%	%	%	%	%	%	%	%	%	%	%	%	%	%	%	%	%	%
Austria	88	7	4	0	16	16	68	0	77	13	10	0	84	11	5	0	94	4	2	0	96	4	0	0	88	7	4	0	89	7	5	0	93	7	1	0	87	7	5	0
Belgium, Dutch-speaking *	91	8	1	0					82	10	8	0	84	13	2	0	89	10	1	0	96	4	0	0	91	8	1	0	91	8	1	0	92	7	1	0	91	8	1	0
Belgium, French-speaking *	89	9	2	0					87	7	4	1	87	9	4	0	90	9	1	0	90	8	1	0	90	8	2	0	88	9	2	0	89	11	0	0	89	8	3	0
Bosnia and Herzegovina	97	3	0	0	71	14	14	0	97	3	0	0	94	5	0	1	98	2	0	0	100	0	0	0	99	1	0	0	93	6	0	1	99	1	0	0	96	4	0	0
Denmark #	62	25	12	0				0	49	31	21	0	60	19	21	0	65	26	9	0	69	30		0	63	25	12	0	61	26	13	0	72	23	5	0	59	26	15	0
Estonia	83	15	1	0	0	0	0	0	62	31	8	0	81	19	0	0	87	13	0	0	100	0	0	0	87	13	0	0	78	19	3	0	87	13	0	0	83	16	1	0
France	86	10	4	0	56	31	13	0	75	11	13	0	83	10	7	0	89	9	2	0	90	10	0	0	87	10	4	0	86	10	4	0	90	8	2	0	85	10	4	0
Greece	96	3	1	0	56	0	44	0	86	4	10	0	95	4	1	0	96	4	0	0	97	3	0	0	96	3	1	0	96	3	1	0	98	2	0	0	95	4	1	0
Iceland	70	18	12	0	0	0	0	0	71	14	14	0	53	24	24	0	80	10	10	0	81	19	0	0	68	21	11	0	75	8	17	0	100	0	0	0	66	20	14	0
Netherlands	68	18	14	0	28	42	31	0	56	17	27	0	65	16	19	0	69	19	11	0	80	17	3	0	68	18	13	0	67	17	16	0	78	18	3	0	65	18	17	0
Norway	55	35	10	0	33	8	58	0	41	40	20	0	60	29	11	0	53	41	6	0	62	36	2	0	55	36	8	0	56	32	12	0	57	41	2	0	55	34	11	0
Romania | |	99	1	0	0	89	11	0	0	97	2	1	0	99	1	0	0	100	0	0	0	100	0	0	0	99	1	0	0	99	1	0	0	99	1	0	0	99	1	0	0
Serbia	90	9	1	0	67	33	0	0	79	13	8	0	92	8	0	0	92	8	0	0	91	8	0	1	92	7	1	0	87	13	1	0	89	9	2	0	90	9	1	0
Spain, Andalusia	80	14	6	0	62	15	23	0	59	25	16	0	77	15	8	0	82	14	4	0	89	10	2	0	79	14	7	0	82	14	4	0	89	8	3	0	77	16	7	0
Spain, Aragon	79	16	6	0	25	0	75	0	59	24	18	0	78	16	5	0	79	17	3	0	87	13	0	0	80	16	3	0	74	15	11	0	78	20	2	0	79	15	6	0
Spain, Basque country	71	26	4	0	0	0	0	0	68	24	8	0	69	25	6	0	72	25	3	0	71	28	1	0	70	26	4	0	71	24	5	0	74	24	2	0	69	26	5	0
Spain, Canary Islands	73	25	2	0	50	50	0	0	64	24	11	0	72	26	2	0	70	28	2	0	80	20	0	0	73	24	3	0	72	27	2	0	78	20	2	0	70	27	3	0
Spain, Cantabria *	75	14	11	0					43	29	29	0	74	5	21	0	75	13	13	0	81	17	2	0	78	13	9	0	65	17	17	0	67	17	17	0	77	13	10	0
Spain, Castile and León *	78	19	3	0					55	35	10	0	69	26	6	0	81	18	1	0	88	11	1	0	79	18	3	0	76	22	2	0	85	15	0	0	76	20	4	0
Spain, Castile-La Mancha *	79	20	1	0					52	45	3	0	76	23	1	0	85	13	1	0	86	14	0	0	81	17	1	0	73	26	1	0	86	14	0	0	76	22	2	0
Spain, Catalonia	74	13	13	0	30	15	55	0	60	17	23	0	71	14	15	0	70	15	14	0	85	10	5	0	74	13	13	0	75	13	12	0	80	14	6	0	73	13	15	0
Spain, Community of Madrid	78	15	7	0	47	6	47	0	70	18	12	0	75	18	7	0	79	17	4	0	88	11	1	0	79	14	7	0	75	17	8	0	88	10	2	0	76	16	8	0
Spain, Extremadura	90	8	2	0	0	0	0	0	50	43	7	0	85	11	4	0	96	4	0	0	98	2	0	0	89	8	3	0	91	9	0	0	98	0	2	0	87	11	2	0
Spain, Galicia	77	20	4	0	0	0	0	0	56	22	22	0	67	28	5	0	75	23	2	0	92	8	0	0	78	19	4	0	75	22	3	0	82	18	0	0	75	20	5	0
Spain, La Rioja	71	29	0	0	0	0	0	0	100	0	0	0	63	37	0	0	60	40	0	0	88	13	0	0	77	23	0	0	63	38	0	0	80	20	0	0	69	31	0	0
Spain, Murcia	80	16	4	0	50	50	0	0	75	14	11	0	74	20	6	0	81	17	1	0	89	11	0	0	83	15	2	0	76	18	6	0	81	17	2	0	80	16	4	0
Spain, Navarre *	76	23	1	0					56	33	11	0	71	29	0	0	74	26	0	0	89	11	0	0	78	20	1	0	67	33	0	0	77	23	0	0	75	23	1	0
Spain, Valencian region	72	20	8	0	67	11	22	0	66	22	12	0	65	20	15	0	67	25	7	0	82	15	3	0	71	21	8	0	73	17	10	0	80	16	5	0	70	21	9	0
Sweden	57	33	10	0	17	44	39	0	55	28	17	0	54	31	15	0	60	31	10	0	61	39	1	0	57	34	9	0	58	32	10	0	59	36	5	0	56	32	11	0
Switzerland	80	11	6	3	22	22	33	22	65	15	15	4	69	13	12	5	86	10	3	2	91	9	0	0	80	11	6	3	79	12	7	2	91	7	2	0	78	12	7	3
United Kingdom, England #	75	18	6	0	39	38	23	0	64	24	12	0	75	18	8	0	78	17	5	0	84	15	1	0	76	19	6	0	74	18	8	0	80	17	3	0	73	19	8	0
United Kingdom, Northern Ireland #	69	12	19	0		0		0	52		37	0	63	13	24	0	76		16	0	78	20		0	69	10	21	0	68	16	16	0	77	13		0	66	12	22	0
United Kingdom, Scotland	77	15	8	0	67	17	17	0	59	25	16	0	74	15	11	0	86	11	3	0	88	12	0	0	78	14	9	0	77	17	6	0	83	13	3	0	75	16	9	0
United Kingdom, Wales #	81	14	5	0				0	62	20	18	0	75	18	6	0	87	12		0	93	8	0	0	81	15	4	0	80	13	6	0	88	10		0	78	16	6	0

Abbreviations used: HD: haemodialysis; PD: peritoneal dialysis; Tx: transplant; Un: unknown; DM: diabetes mellitus
Categories may not add up due to missing values or rounding
* Patients younger than 20 years of age are not reported
# Values based on 1 to 5 patients are omitted
| | The incidence of preemptive transplantation is underestimated by approximately 30%

Table B.3.1

Incident counts and percentages by age and sex

at day 91

	All			0-19						20-44						45-64						65-74						75+					
	All	Male	Female	All		Male		Female		All		Male		Female		All		Male		Female		All		Male		Female		All		Male		Female	
	N (100%)	N (100%)	N (100%)	N	%	N	%	N	%	N	%	N	%	N	%	N	%	N	%	N	%	N	%	N	%	N	%	N	%	N	%	N	%
Austria §	959	639	320	14	1	7	1	7	2	120	13	76	12	44	14	266	28	185	29	81	25	270	28	173	27	97	30	289	30	198	31	91	28
Belgium, Dutch-speaking *	987	655	332							70	7	46	7	24	7	198	20	124	19	74	22	272	28	184	28	88	27	447	45	301	46	146	44
Belgium, French-speaking *	791	500	291							63	8	32	6	31	11	186	24	118	24	68	23	245	31	161	32	84	29	297	38	189	38	108	37
Bosnia and Herzegovina	344	210	134	4	1	3	1	1	1	36	10	23	11	13	10	114	33	66	31	48	36	114	33	74	35	40	30	76	22	44	21	32	24
Denmark #	692	457	235	8	1	7	2			68	10	39	9	29	12	230	33	148	32	82	35	196	28	138	30	58	25	190	27	125	27	65	28
Estonia †	83	47	36	0	0	0	0	0	0	13	16	8	17	5	14	30	36	17	36	13	36	23	28	12	26	11	31	17	21	10	21	7	20
France †	10241	6693	3547	112	1	73	1	39	1	934	9	619	9	315	9	2642	26	1662	25	980	28	2822	28	1894	28	928	26	3731	36	2446	37	1285	36
Greece	2472	1668	804	9	0	6	0	3	0	117	5	70	4	47	6	574	23	400	24	174	22	650	26	448	27	202	25	1122	45	744	45	378	47
Iceland	48	37	11	0	0	0	0	0	0	7	15	5	14	2	18	16	33	12	32	4	36	10	21	6	16	4	36	15	31	14	38	1	9
Netherlands &	1860	1194	666	34	2	25	2	9	1	222	12	138	12	84	13	606	33	372	31	234	35	564	30	354	30	210	32	434	23	305	26	129	19
Norway	542	374	168	12	2	9	2	3	2	79	15	52	14	27	16	163	30	111	30	52	31	137	25	94	25	43	26	151	28	108	29	43	26
Romania	3059	1802	1257	27	1	13	1	14	1	300	10	176	10	124	10	1140	37	733	41	407	32	1044	34	594	33	450	36	548	18	286	16	262	21
Serbia	507	336	171	9	2	7	2	2	1	51	10	36	11	15	9	191	38	124	37	67	39	174	34	121	36	53	31	82	16	48	14	34	20
Spain, Andalusia	1238	790	448	12	1	6	1	6	1	127	10	82	10	45	10	390	32	235	30	155	35	340	27	226	29	114	25	369	30	241	31	128	29
Spain, Aragon	194	141	53	4	2	2	1	2	4	16	8	8	6	8	15	55	28	38	27	17	32	57	29	44	31	13	25	62	32	49	35	13	25
Spain, Basque country	267	188	79	0	0	0	0	0	0	23	9	14	7	9	11	80	30	51	27	29	37	90	34	66	35	24	30	74	28	57	30	17	22
Spain, Canary Islands	400	269	131	2	1	1	0	1	1	44	11	28	10	16	12	142	36	100	37	42	32	100	25	70	26	30	23	112	28	70	26	42	32
Spain, Cantabria *	97	76	21							7	7	5	7	2	10	19	20	16	21	3	14	31	32	25	33	6	29	40	41	30	39	10	48
Spain, Castile and León * †	348	250	98							21	6	15	6	6	6	106	31	75	30	31	32	100	29	73	29	27	27	121	35	86	34	34	35
Spain, Castile-La Mancha *	230	163	67							28	12	13	8	15	22	70	30	54	33	16	24	54	23	41	25	13	19	78	34	55	34	23	34
Spain, Catalonia †	1386	962	423	17	1	15	2	3	1	113	8	75	8	38	9	384	28	260	27	123	29	381	27	270	28	111	26	491	35	343	36	148	35
Spain, Community of Madrid	864	577	276	35	4	22	4	13	5	112	13	66	11	42	15	264	31	180	31	81	29	198	23	128	22	69	25	255	30	181	31	71	26
Spain, Extremadura	157	106	51	0	0	0	0	0	0	15	10	14	13	1	2	45	29	23	22	22	43	47	30	36	34	11	22	50	32	33	31	17	33
Spain, Galicia	417	296	121	0	0	0	0	0	0	34	8	22	7	12	10	125	30	82	28	43	36	121	29	94	32	27	22	137	33	98	33	39	32
Spain, La Rioja	39	24	15	0	0	0	0	0	0	5	13	4	17	1	7	18	46	12	50	6	40	9	23	5	21	4	27	7	18	3	13	4	27
Spain, Murcia	221	139	82	4	2	3	2	1	1	28	13	16	12	12	15	68	31	42	30	26	32	64	29	46	33	18	22	57	26	32	23	25	30
Spain, Navarre *	88	68	20							9	10	8	12	1	5	31	35	19	28	12	60	21	24	19	28	2	10	27	31	22	32	5	25
Spain, Valencian region	729	507	222	9	1	5	1	4	2	64	9	47	9	17	8	207	28	134	26	73	33	199	27	138	27	61	27	250	34	183	36	67	30
Sweden	1050	716	334	18	2	10	1	8	2	113	11	72	10	41	12	332	32	224	31	108	32	251	24	181	25	70	21	336	32	229	32	107	32
Switzerland †	748	520	228	8	1	4	1	4	2	84	11	56	11	28	12	196	26	140	27	56	25	185	25	127	24	58	26	275	37	194	37	81	36
United Kingdom, England # †	6222	3957	2265	141	2	77	2	63	3	1054	17	643	16	411	18	2263	36	1432	36	831	37	1426	23	897	23	529	23	1338	22	908	23	430	19
United Kingdom, Northern Ireland # †	225	135	90							27	12	15	11	12	13	85	38	47	34	38	42	63	28	41	30	22	24	47	21	31	23	16	18
United Kingdom, Scotland †	584	382	202	6	1	3	1	3	1	86	15	61	16	25	12	232	40	150	39	83	41	165	28	103	27	62	31	96	16	66	17	30	15
United Kingdom, Wales # †	449	266	183							66	15	38	14	28	15	155	35	88	33	68	37	107	24	68	26	39	21	116	26	70	26	46	25

Categories may not add up due to missing values or rounding
§ The incidence is underestimated by approximately 2% due to one haemodialysis centre not submitting data
* Patients younger than 20 years of age are not reported
# Values based on 1 to 5 patients are omitted
† The incidence counts at day 91 are estimated (see methods) and could result in decimal numbers that may not add up if rounded
& The incidence is underestimated by approximately 7%

Table B.3.2

Incidence per million (age-related) population by age and sex, unadjusted

at day 91

	All			0-19			20-44			45-64			65-74			75+		
	All	Male	Female	All	Male	Female	All	Male	Female	All	Male	Female	All	Male	Female	All	Male	Female
	Pmp	Pmp	Pmp	Pmarp	Pmarp	Pmarp	Pmarp	Pmarp	Pmarp	Pmarp	Pmarp	Pmarp	Pmarp	Pmarp	Pmarp	Pmarp	Pmarp	Pmarp
Austria	107.5	145.4	70.7	8.1	7.9	8.4	41.3	51.3	30.9	104.4	146.5	63.0	301.5	413.3	203.4	340.1	574.0	180.3
Belgium, Dutch-speaking *	144.9	194.0	96.6				33.5	43.7	23.1	109.5	136.1	82.4	365.5	506.2	231.1	637.5	1017.1	360.3
Belgium, French-speaking *	159.3	205.0	115.1				38.0	38.4	37.6	145.9	185.4	106.6	505.7	713.7	324.4	735.2	1203.7	437.3
Bosnia and Herzegovina	97.4	121.2	74.5	5.1	7.4	2.6	29.4	36.9	21.6	111.8	132.9	91.9	387.7	574.0	242.3	365.4	548.5	250.5
Denmark #	116.4	154.6	78.6	6.2	10.6		36.2	40.9	31.3	148.1	191.0	105.4	314.3	457.7	180.1	317.3	475.8	193.4
Estonia †	63.5	76.2	52.1	0	0	0	31.3	37.3	24.8	87.4	103.2	72.6	160.3	207.3	128.6	139.8	281.0	81.4
France †	149.8	202.0	100.7	7.0	8.9	5.0	45.6	61.0	30.5	151.8	195.1	110.2	370.6	536.2	227.3	538.0	879.0	309.5
Greece	237.5	327.5	151.3	4.7	6.1	3.2	38.8	45.4	31.8	187.2	267.1	111.0	545.3	802.4	318.8	922.5	1465.5	533.4
Iceland	124.5	186.6	58.7	0	0	0	49.3	66.5	30.0	177.1	261.5	90.0	293.2	352.8	233.9	601.8	1219.6	74.4
Netherlands	111.9	144.5	79.6	9.8	14.0	5.3	41.7	51.3	31.9	136.5	168.1	105.1	308.9	395.2	225.7	278.5	446.1	147.5
Norway	98.2	134.5	61.3	9.7	14.1	5.0	43.0	55.4	30.1	115.0	153.8	74.7	250.4	347.7	155.3	316.8	512.0	161.8
Romania	160.5	194.4	128.4	6.5	6.1	7.0	52.0	59.1	44.4	212.3	276.0	150.0	453.0	604.1	340.5	370.1	544.4	274.2
Serbia	79.3	108.1	52.1	7.2	10.9	3.3	26.2	36.5	15.7	107.4	143.1	73.6	193.9	299.0	107.6	155.5	229.2	107.0
Spain, Andalusia	143.8	186.3	102.6	6.9	6.7	7.2	47.5	60.5	34.1	149.2	181.7	117.4	403.2	566.3	256.6	493.8	798.8	287.3
Spain, Aragon	144.1	211.9	77.8	16.2	15.7	16.8	41.1	40.1	42.1	134.0	183.2	83.7	393.4	636.8	171.5	400.2	784.8	140.5
Spain, Basque country	120.2	174.0	69.2	0	0	0	37.7	45.3	29.9	115.4	148.5	83.0	347.9	545.6	174.3	277.5	545.4	104.8
Spain, Canary Islands	179.7	244.8	116.2	5.3	5.2	5.5	60.4	76.9	43.8	195.0	274.5	115.4	462.0	675.6	265.8	636.2	957.6	408.0
Spain, Cantabria *	164.5	266.0	69.1				43.5	61.8	25.0	100.7	172.1	31.4	427.2	729.0	156.8	592.7	1142.4	242.6
Spain, Castile and León * †	146.1	213.3	81.0				34.2	48.0	19.7	141.5	199.6	82.9	338.4	501.4	178.7	359.5	632.1	172.5
Spain, Castile-La Mancha *	109.8	155.2	64.2				43.9	39.4	48.8	109.8	166.3	51.1	267.9	415.8	126.3	374.4	639.9	187.9
Spain, Catalonia †	175.4	247.6	105.4	11.2	18.3	3.5	45.4	59.2	31.1	165.3	224.5	106.2	494.0	756.5	268.0	646.9	1137.0	323.8
Spain, Community of Madrid	125.7	175.5	77.0	26.2	32.2	20.0	50.3	60.2	37.1	129.1	183.2	76.3	310.5	451.5	194.8	405.5	747.2	183.7
Spain, Extremadura	148.9	203.2	95.7	0	0	0	48.7	89.2	6.6	137.4	139.5	135.2	416.6	646.5	192.5	425.7	699.3	242.0
Spain, Galicia	154.3	227.6	86.3	0	0	0	47.0	60.5	33.4	147.7	198.1	99.4	364.3	603.5	153.0	357.9	645.8	168.8
Spain, La Rioja	120.7	150.4	91.6	0	0	0	54.3	86.1	21.9	181.4	240.2	121.7	258.1	294.0	223.9	194.1	203.5	187.6
Spain, Murcia	142.4	178.7	106.0	11.6	16.9	6.0	55.6	61.6	49.2	150.7	184.0	116.7	480.7	729.8	256.7	473.0	649.6	350.9
Spain, Navarre *	130.3	203.4	58.7				45.2	79.3	10.2	153.6	186.3	120.2	304.8	570.8	56.2	385.0	770.5	120.3
Spain, Valencian region	139.8	197.6	83.8	9.0	9.7	8.2	40.6	59.1	21.8	130.1	168.9	91.5	365.1	540.3	210.6	497.6	892.7	225.3
Sweden	99.7	135.0	63.8	7.4	8.0	6.8	33.4	41.3	25.1	129.1	172.1	85.1	236.2	346.5	129.5	306.0	467.7	175.8
Switzerland †	88.6	124.0	53.6	4.6	4.2	4.9	30.1	39.4	20.4	83.6	119.0	48.0	226.7	323.9	137.1	340.8	567.6	174.5
United Kingdom, England # †	117.2	152.1	83.7	11.5	12.3	10.6	60.4	74.9	46.3	168.7	217.4	121.8	283.2	370.4	202.3	273.9	426.0	156.3
United Kingdom, Northern Ireland # †	117.3	143.1	92.3				45.0	50.4	39.6	171.3	192.8	150.6	343.5	455.0	236.4	293.7	448.5	176.0
United Kingdom, Scotland †	106.4	143.3	71.6	5.3	5.2	5.4	48.8	70.9	27.6	156.6	208.2	108.0	272.8	355.2	197.1	186.6	301.0	101.1
United Kingdom, Wales # †	142.0	171.3	113.8				68.0	78.2	58.0	190.2	220.4	161.5	304.7	400.0	215.2	350.4	476.6	250.6

* Patients younger than 20 years of age are not reported
# Values based on 1 to 5 patients are omitted
† The incident counts at day 91, on which the data presented in this table are based, are estimated (see methods)

Table B.3.3

Sex, mean age, and median age of incident patients

at day 91

	All						Male						Female					
	%	Mean (years)	SD	Median (years)	P25	P75	%	Mean (years)	SD	Median (years)	P25	P75	%	Mean (years)	SD	Median (years)	P25	P75
Austria	100	64.5	17.0	68.0	56.4	76.8	66.6	64.8	16.7	67.9	57.1	77.1	33.4	63.8	17.7	68.3	55.3	76.1
Belgium, Dutch-speaking *	100	70.7	14.1	73.8	63.9	80.9	66.4	70.9	14.0	73.8	64.6	80.8	33.6	70.4	14.3	73.5	62.4	80.9
Belgium, French-speaking *	100	68.4	14.3	71.2	60.5	78.4	63.2	68.8	13.7	72.0	60.8	78.4	36.8	67.8	15.4	70.3	60.1	78.2
Bosnia and Herzegovina	100	64.0	14.8	66.8	57.0	73.8	61.0	64.0	15.1	67.7	57.8	73.5	39.0	63.9	14.2	65.9	56.5	74.8
Denmark	100	64.3	15.7	67.6	56.1	75.8	66.0	64.8	15.5	68.1	57.7	75.7	34.0	63.2	16.0	65.7	52.5	76.1
Estonia †	100	62.2	15.4	63.6	54.2	72.6	56.9	61.4	16.0	63.0	52.3	72.6	43.1	63.0	14.8	64.5	54.4	72.0
France †	100	67.0	15.9	70.6	58.7	78.2	65.4	67.1	16.1	70.8	58.9	78.2	34.6	67.0	15.6	70.4	58.3	78.3
Greece	100	70.8	13.7	73.4	63.3	80.9	67.5	70.8	13.4	73.1	63.4	80.6	32.5	71.0	14.5	74.0	63.3	81.9
Iceland	100	64.9	15.5	66.0	54.4	79.6	77.1	66.0	16.0	66.3	55.6	80.7	22.9	61.3	13.9	65.0	49.4	69.9
Netherlands	100	62.8	15.9	66.6	53.4	74.6	64.2	63.4	16.3	67.4	54.5	75.3	35.8	61.7	15.2	65.5	52.1	73.5
Norway	100	62.2	17.8	66.2	50.9	75.6	69.0	62.6	18.0	66.8	51.9	75.9	31.0	61.4	17.3	65.6	48.9	75.1
Romania	100	63.0	14.1	65.7	54.8	72.8	58.9	62.4	13.7	64.6	54.5	71.8	41.1	63.7	14.7	67.0	55.5	74.0
Serbia	100	62.2	14.7	65.3	55.3	72.5	66.3	62.1	15.0	65.4	55.7	72.3	33.7	62.5	14.2	65.3	54.9	72.9
Spain, Andalusia	100	64.9	15.1	68.2	56.3	76.2	63.8	65.2	14.9	68.9	57.3	76.4	36.2	64.4	15.4	66.8	55.3	75.9
Spain, Aragon	100	65.8	15.9	69.8	58.3	77.2	72.7	67.8	14.4	70.9	59.8	77.6	27.3	60.5	18.5	64.1	50.4	74.8
Spain, Basque country	100	66.1	13.7	68.6	59.1	76.2	70.4	67.1	13.3	69.9	60.2	76.4	29.6	63.6	14.6	65.5	55.2	73.3
Spain, Canary Islands	100	64.0	14.7	66.4	54.4	75.8	67.3	64.0	14.4	66.0	55.1	75.4	32.8	64.1	15.3	67.7	52.5	76.6
Spain, Cantabria *	100	70.1	14.4	73.9	64.7	79.4	78.4	69.7	13.8	73.7	64.3	78.7	21.6	71.3	16.4	73.9	66.9	80.8
Spain, Castile and León * †	100	67.6	13.7	70.4	59.5	78.0	71.9	67.1	14.6	70.1	59.0	78.6	28.1	68.7	11.2	71.1	61.9	77.2
Spain, Castile-La Mancha *	100	65.6	14.7	66.8	56.7	77.4	70.9	66.8	13.6	66.8	58.5	77.6	29.1	62.7	16.9	66.5	48.6	77.2
Spain, Catalonia †	100	66.7	15.6	70.6	57.5	78.1	69.4	66.5	15.5	70.6	57.5	77.6	30.6	67.0	15.6	70.5	57.5	79.0
Spain, Community of Madrid	100	62.1	18.9	66.3	52.5	76.1	66.8	63.0	18.2	66.8	53.9	76.5	31.9	60.3	20.0	65.6	50.2	75.3
Spain, Extremadura	100	67.7	14.1	70.0	59.0	78.4	67.5	66.8	14.5	70.1	58.9	78.0	32.5	69.7	13.2	69.2	59.0	81.5
Spain, Galicia	100	66.8	13.8	69.7	59.3	77.3	71.0	67.3	13.4	70.0	61.1	76.5	29.0	65.5	14.8	68.7	54.2	78.1
Spain, La Rioja	100	61.5	13.8	62.4	51.7	71.0	61.5	59.1	13.5	60.5	51.7	66.9	38.5	65.4	13.6	66.9	58.7	79.7
Spain, Murcia	100	63.5	16.0	66.7	54.3	75.1	62.9	63.6	15.8	67.2	55.3	74.2	37.1	63.4	16.3	66.5	49.8	76.2
Spain, Navarre *	100	64.7	14.7	67.1	55.6	76.5	77.3	65.5	15.5	69.3	56.0	76.6	22.7	62.1	11.9	59.9	52.6	72.1
Spain, Valencian region	100	66.3	15.5	69.4	58.5	77.6	69.5	66.8	15.4	70.1	59.2	77.9	30.5	65.2	15.6	67.5	57.4	77.0
Sweden	100	64.3	16.7	67.6	55.2	77.2	68.2	64.8	16.4	68.4	55.9	77.4	31.8	63.2	17.2	66.2	53.9	76.7
Switzerland †	100	66.2	16.8	69.8	57.1	78.7	69.6	66.4	16.5	69.4	57.3	78.8	30.4	65.5	17.5	70.1	57.1	78.4
United Kingdom, England †	100	60.1	17.0	62.4	49.7	73.6	63.6	60.7	16.8	62.8	50.4	74.2	36.4	59.0	17.3	61.5	48.4	72.7
United Kingdom, Northern Ireland †	100	62.6	15.1	64.6	53.5	74.0	60.1	63.1	15.2	66.4	52.1	74.5	39.9	61.8	15.0	63.0	55.4	73.9
United Kingdom, Scotland †	100	60.4	14.9	62.6	51.9	71.9	65.4	60.3	15.0	62.3	52.1	71.8	34.6	60.5	14.8	63.4	51.9	72.0
United Kingdom, Wales †	100	61.9	16.6	64.2	52.2	74.9	59.2	62.0	17.0	64.8	51.6	74.9	40.8	61.9	16.0	63.6	52.6	74.9

Categories may not add up due to missing values or rounding
* Patients younger than 20 years of age are not reported
† The incident counts at day 91, on which the data presented in this table are based, are estimated (see methods)

Table B.3.4

Incidence per million population, adjusted

at day 91, adjusted for age and sex

	All	Male	Female
	Pmp	Pmp	Pmp
Austria	109.4	147.9	72.5
Belgium, Dutch-speaking *	142.7	187.9	99.5
Belgium, French-speaking *	182.8	238.1	129.9
Bosnia and Herzegovina	124.2	163.1	87.1
Denmark	117.4	155.1	81.4
Estonia †	68.8	86.9	51.6
France †	153.0	204.1	104.2
Greece	217.3	297.1	141.1
Iceland	154.1	241.0	71.0
Netherlands	112.1	143.3	82.2
Norway	103.6	142.1	66.8
Romania	167.5	208.8	127.9
Serbia	75.8	103.9	49.0
Spain, Andalusia	151.5	196.8	108.2
Spain, Aragon	137.8	201.5	76.9
Spain, Basque country	111.6	160.2	65.3
Spain, Canary Islands	186.8	252.2	124.2
Spain, Cantabria *	150.6	239.5	65.6
Spain, Castile and León * †	125.3	179.6	73.3
Spain, Castile-La Mancha *	110.7	156.8	66.6
Spain, Catalonia †	185.2	262.1	111.7
Spain, Community of Madrid	133.7	187.9	81.9
Spain, Extremadura	140.1	191.6	90.8
Spain, Galicia	134.6	194.7	77.1
Spain, La Rioja	118.5	146.9	91.3
Spain, Murcia	163.4	208.3	120.5
Spain, Navarre *	128.2	201.5	58.0
Spain, Valencian region	141.1	199.4	85.4
Sweden	101.6	137.3	67.5
Switzerland †	91.3	127.2	56.9
United Kingdom, England †	125.4	161.3	91.1
United Kingdom, Northern Ireland †	129.3	157.1	102.8
United Kingdom, Scotland †	107.2	143.2	72.8
United Kingdom, Wales †	142.2	169.9	115.7

* Patients younger than 20 years of age are not reported
† The incident counts at day 91, on which the data presented in this table are based, are estimated (see methods)

Table B.3.5.A

Incidence per million population and percentages by primary renal disease (1995 PRD codes), unadjusted

at day 91

	All		GN		PN		PKD		DM						HT		RVD		Misc		Unkn		Missing	
									Type 1		Type 2		Total											
	Pmp	%	Pmp	%	Pmp	%	Pmp	%	Pmp	%	Pmp	%	Pmp	%	Pmp	%	Pmp	%	Pmp	%	Pmp	%	Pmp	%
Austria	107.5	100	10.0	9.3	4.3	4.0	5.9	5.5	3.3	3.0	17.9	16.7	21.2	19.7	16.4	15.2	2.4	2.2	24.8	23.0	22.0	20.4	0.7	0.6
Belgium, Dutch-speaking *	144.9	100	14.8	10.2	5.3	3.6	9.2	6.4	3.5	2.4	28.2	19.5	31.7	21.9	17.3	12.0	7.2	5.0	45.5	31.4	13.8	9.5	0	0
Belgium, French-speaking *	159.3	100	14.7	9.2	6.2	3.9	7.2	4.6	2.4	1.5	31.2	19.6	33.6	21.1	26.0	16.3	1.2	0.8	45.3	28.4	25.0	15.7	0	0
Bosnia and Herzegovina	97.4	100	5.1	5.2	13.0	13.4	5.1	5.2	3.7	3.8	23.8	24.4	27.5	28.2	6.2	6.4	4.0	4.1	16.1	16.6	20.4	20.9	0	0
Denmark #	116.4	100	13.1	11.3	5.7	4.9	10.6	9.1	7.2	6.2	23.0	19.8	30.3	26.0	12.4	10.7	1.5	1.3	19.7	16.9	23.0	19.8	0	0
Estonia †	63.5	100	15.4	24.2	3.1	4.8	6.1	9.7					11.5	18.1	17.4	27.4	0	0	10.0	15.7	0	0	0	0
France †	149.8	100	15.7	10.5	7.6	5.1	9.3	6.2					34.0	22.7	35.7	23.8	1.5	1.0	22.4	15.0	23.5	15.7	0	0
Greece	237.5	100	18.0	7.6	12.4	5.2	10.6	4.4	2.0	0.8	53.5	22.5	55.5	23.4	22.4	9.4	6.1	2.6	30.5	12.8	82.1	34.5	0	0
Iceland	124.5	100	15.6	12.5	5.2	4.2	13.0	10.4	0	0	13.0	10.4	13.0	10.4	13.0	10.4	46.7	37.5	18.2	14.6	0	0	0	0
Netherlands	111.9	100	12.6	11.2	4.1	3.7	6.6	5.9	4.0	3.5	20.6	18.4	24.6	22.0	17.9	16.0	3.7	3.3	20.9	18.7	21.4	19.1	0.2	0.2
Norway	98.2	100	15.4	15.7	3.6	3.7	8.0	8.1	3.6	3.7	10.9	11.1	14.5	14.8	30.1	30.6	1.3	1.3	22.1	22.5	2.7	2.8	0.5	0.6
Romania	160.5	100	21.9	13.6	4.2	2.6	3.1	2.0					19.0	11.9	11.0	6.8	1.2	0.7	25.4	15.9	74.6	46.5	0	0
Serbia	79.3	100	6.1	7.7	7.8	9.9	3.6	4.5					18.6	23.5	28.2	35.5	0.8	1.0	11.3	14.2	3.0	3.7	0	0
Spain, Andalusia	143.8	100	17.5	12.2	7.9	5.5	9.1	6.3					33.1	23.0	14.9	10.3	1.9	1.3	26.4	18.3	33.1	23.0	0	0
Spain, Aragon	144.1	100	19.3	13.4	8.2	5.7	8.9	6.2	2.2	1.5	28.2	19.6	30.5	21.1	20.1	13.9	0	0	31.2	21.6	26.0	18.0	0	0
Spain, Basque country	120.2	100	10.8	9.0	3.2	2.6	9.9	8.2	5.0	4.1	24.3	20.2	29.3	24.3	16.7	13.9	2.3	1.9	27.5	22.8	20.7	17.2	0	0
Spain, Canary Islands	179.7	100	18.9	10.5	7.2	4.0	13.9	7.8	8.5	4.8	47.2	26.3	55.7	31.0	11.7	6.5	0.4	0.3	42.2	23.5	29.7	16.5	0	0
Spain, Cantabria *	164.5	100	11.9	7.2	6.8	4.1	10.2	6.2	6.8	4.1	22.0	13.4	28.8	17.5	11.9	7.2	1.7	1.0	22.0	13.4	28.8	17.5	42.4	25.8
Spain, Castile and León * † ‡	146.1	100	17.1	11.7	10.1	6.9	10.5	7.2	8.4	5.7	26.8	18.4	35.2	24.1	19.7	13.5	0.4	0.3	33.8	23.2	19.3	13.2	0	0
Spain, Castile-La Mancha *	109.8	100	15.3	13.9	4.3	3.9	11.5	10.4					28.6	26.1	6.2	5.7	0	0	22.4	20.4	21.0	19.1	0.5	0.4
Spain, Catalonia †	175.4	100	15.3	8.7	6.7	3.8	10.3	5.9	4.2	2.4	36.6	20.9	41.3	23.5	11.3	6.4	2.5	1.4	13.5	7.7	35.3	20.1	39.4	22.5
Spain, Community of Madrid	125.7	100	11.8	9.4	3.5	2.8	5.7	4.5	3.5	2.8	20.5	16.3	24.0	19.1	7.1	5.7	0	0	17.8	14.1	13.7	10.9	42.2	33.6
Spain, Extremadura	148.9	100	13.3	8.9	7.6	5.1	6.6	4.5					39.8	26.8	7.6	5.1	0	0	41.7	28.0	30.4	20.4	1.9	1.3
Spain, Galicia	154.3	100	15.2	9.8	6.7	4.3	14.8	9.6	4.8	3.1	30.3	19.7	35.2	22.8	15.9	10.3	1.9	1.2	37.0	24.0	27.8	18.0	0	0
Spain, La Rioja	120.7	100	9.3	7.7	3.1	2.6	6.2	5.1	6.2	5.1	18.6	15.4	24.7	20.5	18.6	15.4	0	0	40.2	33.3	18.6	15.4	0	0
Spain, Murcia	142.4	100	25.8	18.1	6.4	4.5	11.0	7.7	2.6	1.8	29.0	20.4	31.6	22.2	11.6	8.1	0	0	31.6	22.2	24.5	17.2	0	0
Spain, Navarre *	130.3	100	19.3	14.8	1.5	1.1	14.8	11.4	1.5	1.1	16.3	12.5	17.8	13.6	23.7	18.2	0	0	29.6	22.7	23.7	18.2	0	0
Spain, Valencian region	139.8	100	17.3	12.3	6.1	4.4	9.6	6.9					28.4	20.3	20.5	14.7	1.3	1.0	29.7	21.3	26.8	19.2	0	0
Sweden ‡	99.7	100	15.5	15.5	4.1	4.1	8.2	8.2	6.5	6.5	17.7	17.8	24.2	24.3	20.2	20.3	0.5	0.5	18.5	18.6	8.5	8.6	0	0
Switzerland †	88.6	100	12.2	13.8	1.5	1.7	5.1	5.7	1.9	2.1	13.9	15.6	15.8	17.8	22.6	25.5	2.0	2.3	16.9	19.1	7.5	8.5	5.0	5.6
United Kingdom, England # †	117.2	100	12.1	10.3	6.6	5.6	6.5	5.6					31.6	26.9	8.7	7.4	1.0	0.8	19.7	16.8	16.6	14.2	14.4	12.3
United Kingdom, Northern Ireland # †	117.3	100	12.5	10.7	5.4	4.6	9.9	8.4					27.1	23.1					30.9	26.3	21.3	18.2	8.2	7.0
United Kingdom, Scotland †	106.4	100	16.7	15.7	6.7	6.3	9.2	8.7					31.3	29.4	6.7	6.3	2.1	2.0	20.0	18.8	9.8	9.2	3.8	3.6
United Kingdom, Wales # †	142.0	100	21.1	14.8	10.5	7.4	6.6	4.7					38.9	27.4	7.4	5.2			34.6	24.3	21.7	15.3		

Abbreviations used: GN: glomerulonephritis/sclerosis; PN: pyelonephritis; PKD: polycystic kidneys, adult type; DM: diabetes mellitus; HT: hypertension; RVD: renal vascular disease; Misc: miscellaneous; Unkn: unknown
Categories may not add up due to rounding or a limited number of cases (<10%) with diabetes mellitus type unknown; When cells are left empty, (complete) data are unavailable
* Patients younger than 20 years of age are not reported
# Values based on 1 to 5 patients are omitted
† The incident counts at day 91, on which the data presented in this table are based, are estimated (see methods)
‡ Mapping the 2018 PRD codes to the old PRD codes results in a different distribution of PRD groups

FIND

Table B.3.5.B

Incidence per million population and percentages by primary renal disease (2012/2018 PRD codes), unadjusted
at day 91

	All		Glomerular disease		Tubulo-interstitial disease		DM		HT / RVD		Other systemic diseases		Familial/hereditary nephropathies		Misc		Unkn		Missing	
	Pmp	%	Pmp	%	Pmp	%	Pmp	%	Pmp	%	Pmp	%	Pmp	%	Pmp	%	Pmp	%	Pmp	%
Austria	107.5	100	13.3	12.4	8.1	7.5	21.2	19.7	22.9	21.3	7.6	7.1	7.4	6.9	8.9	8.2	17.3	16.1	0.9	0.8
Belgium, Dutch-speaking *	144.9	100	18.6	12.9	9.5	6.6	31.7	21.9	33.2	22.9	18.6	12.9	12.6	8.7	12.5	8.6	8.1	5.6	0	0
Belgium, French-speaking *	159.3	100	19.3	12.1	17.9	11.3	33.6	21.1	29.0	18.2	14.1	8.8	11.7	7.3	16.7	10.5	16.9	10.6	0	0
Bosnia and Herzegovina	97.4	100	6.2	6.4	17.6	18.0	27.5	28.2	10.2	10.5	0.8	0.9	9.1	9.3	3.7	3.8	22.4	23.0	0	0
Denmark #	116.4	100	17.8	15.3	8.7	7.5	30.3	26.0	15.1	13.0	3.4	2.9	12.3	10.5	7.7	6.6	21.0	18.1	0	0
Iceland	124.5	100	20.7	16.7	13.0	10.4	13.0	10.4	59.6	47.9	2.6	2.1	15.6	12.5	0	0	0	0	0	0
Netherlands	111.9	100	17.4	15.5	8.5	7.6	24.6	22.0	23.1	20.6	3.4	3.0	9.6	8.5	10.8	9.7	14.4	12.8	0.2	0.2
Norway	98.2	100	19.6	19.9	9.4	9.6	14.5	14.8	31.7	32.3	3.6	3.7	12.1	12.4	4.2	4.2	2.4	2.4	0.7	0.7
Spain, Andalusia	143.8	100	21.8	15.2	14.6	10.2	33.1	23.0	19.2	13.3	6.2	4.3	12.1	8.4	5.2	3.6	31.6	22.0	0	0
Spain, Aragon	144.1	100	22.3	15.5	20.1	13.9	30.5	21.1	23.0	16.0	3.0	2.1	10.4	7.2	22.3	15.5	12.6	8.8	0	0
Spain, Basque country	120.2	100	15.8	13.1	11.3	9.4	29.3	24.3	22.5	18.7	4.5	3.7	12.6	10.5	4.5	3.7	19.8	16.5	0	0
Spain, Canary Islands	179.7	100	24.3	13.5	15.3	8.5	55.7	31.0	19.8	11.0	11.2	6.3	18.9	10.5	4.9	2.8	29.7	16.5	0	0
Spain, Cantabria *	164.5	100	17.0	10.3	13.6	8.2	28.8	17.5	17.0	10.3	3.4	2.1	13.6	8.2	3.4	2.1	25.4	15.5	42.4	25.8
Spain, Castile and León * †	146.1	100	24.2	16.6	21.3	14.5	35.2	24.1	23.1	15.8	6.3	4.3	14.7	10.0	3.4	2.3	18.0	12.3	0	0
Spain, Castile-La Mancha *	109.8	100	20.5	18.7	10.0	9.1	28.6	26.1	9.1	8.3	3.3	3.0	13.4	12.2	4.3	3.9	20.1	18.3	0.5	0.4
Spain, Catalonia †	175.4	100	17.2	9.8	9.2	5.2	40.1	22.9	13.5	7.7	4.6	2.6	11.9	6.8	5.7	3.2	33.8	19.3	39.4	22.5
Spain, Community of Madrid	125.7	100	14.0	11.1	9.2	7.3	24.0	19.1	9.2	7.3	2.6	2.1	8.1	6.5	7.1	5.7	9.3	7.4	42.2	33.6
Spain, Extremadura	148.9	100	14.2	9.6	12.3	8.3	39.8	26.8	23.7	15.9	3.8	2.5	14.2	9.6	10.4	7.0	28.5	19.1	1.9	1.3
Spain, Galicia	154.3	100	20.7	13.4	14.1	9.1	35.2	22.8	21.5	13.9	8.1	5.3	20.0	12.9	10.4	6.7	24.4	15.8	0	0
Spain, La Rioja	120.7	100	18.6	15.4	12.4	10.3	24.7	20.5	30.9	25.6	3.1	2.6	6.2	5.1	6.2	5.1	18.6	15.4	0	0
Spain, Murcia	142.4	100	31.6	22.2	14.2	10.0	31.6	22.2	20.0	14.0	1.9	1.4	13.5	9.5	5.8	4.1	23.8	16.7	0	0
Spain, Navarre *	130.3	100	22.2	17.0	10.4	8.0	17.8	13.6	29.6	22.7	7.4	5.7	14.8	11.4	4.4	3.4	23.7	18.2	0	0
Spain, Valencian region	139.8	100	21.1	15.1	14.2	10.2	28.4	20.3	24.7	17.7	8.6	6.2	12.3	8.8	6.5	4.7	24.0	17.1	0	0
Sweden	99.7	100	19.4	19.4	9.5	9.5	24.2	24.3	22.2	22.3	2.8	2.8	9.6	9.6	6.3	6.3	5.8	5.8	0	0
United Kingdom, England # †	117.2	100	16.4	14.0	9.8	8.3	31.5	26.9	12.1	10.3	3.1	2.7	8.4	7.1	7.0	6.0	14.4	12.3	14.4	12.3
United Kingdom, Northern Ireland # †	117.3	100	15.1	12.9	15.6	13.3	27.1	23.1	7.3	6.2	5.2	4.4	10.9	9.3	13.4	11.4	14.1	12.0	8.7	7.4
United Kingdom, Scotland †	106.4	100	20.2	18.9	9.0	8.5	31.3	29.4	13.1	12.3	3.1	2.9	11.7	11.0	3.5	3.3	9.0	8.5	5.5	5.1
United Kingdom, Wales # †	142.0	100	27.1	19.1	14.3	10.1	38.9	27.4	17.6	12.4	4.4	3.1	9.8	6.9	13.0	9.1	16.3	11.5		

Abbreviations used: DM: diabetes mellitus; HT / RVD: hypertension / renal vascular disease; Misc: miscellaneous renal disorders; Unkn: unknown
* Patients younger than 20 years of age are not reported
# Values based on 1 to 5 patients are omitted
† The incident counts at day 91, on which the data presented in this table are based, are estimated (see methods)

Table B.3.6.A

Incidence per million population by primary renal disease (1995 PRD codes), adjusted

at day 91, adjusted for age and sex

	All	GN	PN	PKD	DM			HT	RVD	Misc	Unkn	Missing
					Type 1	Type 2	Total					
	Pmp	Pmp	Pmp	Pmp	Pmp	Pmp	Pmp	Pmp	Pmp	Pmp	Pmp	Pmp
Austria	109.4	9.9	4.3	6.0	3.2	18.5	21.6	16.8	2.4	25.2	22.5	0.7
Belgium, Dutch-speaking *	142.7	14.9	5.2	9.5	3.6	27.7	31.3	16.9	6.9	44.2	13.8	0
Belgium, French-speaking *	182.8	16.2	7.1	8.2	2.7	36.1	38.8	30.9	1.5	51.8	28.3	0
Bosnia and Herzegovina	124.2	6.3	17.8	5.8	4.7	29.0	33.8	7.4	4.8	24.1	24.3	0
Denmark #	117.4	13.2	5.6	11.0	7.8	22.7	30.4	12.6	1.5	19.9	23.2	0
Estonia †	68.8	15.6	3.2	6.3			12.9	20.4	0	10.5	0	0
France †	153.0	16.4	7.7	9.9			34.7	36.1	1.5	22.9	23.8	0
Greece	217.3	17.0	11.3	9.9	2.0	48.9	50.8	20.3	5.6	28.2	74.2	0
Iceland	154.1	15.2	7.0	15.4	0	15.5	15.5	17.9	62.3	20.7	0	0
Netherlands	112.1	12.5	4.0	7.0	4.1	20.5	24.6	17.9	3.7	21.0	21.3	0.2
Norway	103.6	15.7	3.8	8.7	3.8	11.7	15.5	32.1	1.5	23.1	2.7	0.6
Romania	167.5	22.4	4.5	3.0			19.7	11.8	1.2	26.6	78.3	0
Serbia	75.8	5.9	7.5	3.5			17.6	26.9	0.8	10.8	2.8	0
Spain, Andalusia	151.5	17.8	8.2	8.9			35.0	16.3	2.0	27.8	35.5	0
Spain, Aragon	137.8	19.0	7.5	8.5	2.3	26.9	29.1	19.1	0	29.6	24.9	0
Spain, Basque country	111.6	10.4	2.7	9.4	4.7	22.6	27.3	15.1	1.8	25.8	19.1	0
Spain, Canary Islands	186.8	18.9	7.3	12.2	7.3	51.0	58.4	13.0	0.5	45.5	30.8	0
Spain, Cantabria *	150.6	11.4	6.6	9.2	6.8	19.6	26.4	10.1	1.8	20.3	25.1	39.4
Spain, Castile and León * † ‡	125.3	14.8	8.6	9.4	7.9	22.1	30.0	16.0	0.3	29.6	16.4	0
Spain, Castile-La Mancha *	110.7	15.0	4.3	11.4			29.0	5.7	0	23.3	21.4	0.6
Spain, Catalonia †	185.2	15.6	7.1	10.3	3.9	39.1	43.5	12.1	2.7	14.3	37.8	41.7
Spain, Community of Madrid	133.7	12.0	3.7	6.0	3.5	22.6	26.1	8.0	0	19.0	14.8	44.1
Spain, Extremadura	140.1	13.0	7.0	6.2			37.8	7.6	0	39.2	27.6	1.7
Spain, Galicia	134.6	13.4	6.1	13.4	4.5	25.8	30.3	13.8	1.4	32.8	23.3	0
Spain, La Rioja	118.5	8.6	3.3	6.1	6.5	17.2	23.7	17.7	0	41.1	18.1	0
Spain, Murcia	163.4	28.2	7.3	11.4	2.4	35.6	37.9	13.9	0	34.4	30.1	0
Spain, Navarre *	128.2	19.0	1.6	14.6	1.3	16.2	17.5	23.3	0	29.2	23.1	0
Spain, Valencian region	141.1	17.1	6.2	9.2			28.7	20.9	1.4	30.1	27.5	0
Sweden ‡	101.6	16.0	3.9	8.8	6.8	18.1	24.9	20.1	0.5	18.9	8.5	0
Switzerland †	91.3	12.3	1.6	5.3	1.8	14.6	16.4	23.4	2.1	17.4	7.9	4.9
United Kingdom, England # †	125.4	12.8	6.9	7.1			34.3	9.4	1.1	20.9	17.8	15.2
United Kingdom, Northern Ireland # †	129.3	13.5	5.7	11.1			29.1			34.5	24.3	8.8
United Kingdom, Scotland †	107.2	16.8	6.7	9.5			31.2	6.9	2.1	20.0	10.1	4.0
United Kingdom, Wales # †	142.2	21.5	10.4	7.1			38.7	7.3		33.7	22.5	

Abbreviations used: GN: glomerulonephritis/sclerosis; PN: pyelonephritis; PKD: polycystic kidneys, adult type; DM: diabetes mellitus; HT: hypertension; RVD: renal vascular disease; Misc: miscellaneous; Unkn: unknown
Categories may not add up due to rounding or a limited number of cases (<10%) with diabetes mellitus type unknown; When cells are left empty, (complete) data are unavailable
* Patients younger than 20 years of age are not reported
# Values based on 1 to 5 patients are omitted
† The incident counts at day 91, on which the data presented in this table are based, are estimated (see methods)
‡ Mapping the 2018 PRD codes to the old PRD codes results in a different distribution of PRD groups

FIND

Table B.3.6.B

Incidence per million population by primary renal disease (2012/2018 PRD codes), adjusted

at day 91, adjusted for age and sex

	All	Glomerular disease	Tubulo-interstitial disease	DM	HT / RVD	Other systemic diseases	Familial/hereditary nephropathies	Misc	Unkn	Missing
	Pmp	Pmp	Pmp	Pmp	Pmp	Pmp	Pmp	Pmp	Pmp	Pmp
Austria	109.4	13.3	8.2	21.6	23.3	7.8	7.4	9.2	17.6	0.9
Belgium, Dutch-speaking *	142.7	18.6	9.4	31.3	32.1	18.1	12.9	12.3	8.1	0
Belgium, French-speaking *	182.8	21.3	20.0	38.8	34.6	16.5	13.1	19.4	19.0	0
Bosnia and Herzegovina	124.2	7.5	26.5	33.8	12.2	1.0	10.3	5.4	27.6	0
Denmark #	117.4	18.1	8.6	30.4	15.3	3.4	12.7	7.6	21.3	0
Iceland	154.1	21.5	16.7	15.5	80.2	3.0	17.2	0	0	0
Netherlands	112.1	17.4	8.2	24.6	23.1	3.4	10.0	11.0	14.2	0.2
Norway	103.6	20.0	9.9	15.5	33.9	3.8	12.9	4.5	2.4	0.7
Spain, Andalusia	151.5	22.2	15.3	35.0	21.1	6.5	12.0	5.5	33.8	0
Spain, Aragon	137.8	22.1	19.2	29.1	21.9	2.4	10.0	21.2	12.0	0
Spain, Basque country	111.6	15.3	10.1	27.3	20.2	4.3	12.0	4.1	18.3	0
Spain, Canary Islands	186.8	24.7	15.7	58.4	22.3	12.0	17.1	5.7	30.8	0
Spain, Cantabria *	150.6	16.2	12.7	26.4	15.0	3.0	12.7	3.1	22.0	39.4
Spain, Castile and León * †	125.3	21.2	18.3	30.0	18.7	5.6	13.4	2.8	15.4	0
Spain, Castile-La Mancha *	110.7	20.3	10.5	29.0	8.9	3.5	13.4	4.1	20.5	0.6
Spain, Catalonia †	185.2	17.6	9.8	42.4	14.5	5.1	11.9	6.0	36.3	41.7
Spain, Community of Madrid	133.7	14.2	9.8	26.1	10.3	2.7	8.6	8.1	9.9	44.1
Spain, Extremadura	140.1	14.1	11.5	37.8	22.4	3.6	13.6	9.5	25.8	1.7
Spain, Galicia	134.6	18.3	13.1	30.3	18.2	7.1	18.4	8.9	20.3	0
Spain, La Rioja	118.5	17.3	12.7	23.7	30.8	3.0	6.1	6.8	18.1	0
Spain, Murcia	163.4	33.8	15.1	37.9	24.7	2.1	13.8	6.4	29.5	0
Spain, Navarre *	128.2	21.7	10.3	17.5	29.0	7.4	14.6	4.5	23.1	0
Spain, Valencian region	141.1	20.9	14.2	28.7	25.2	9.0	11.8	6.7	24.5	0
Sweden	101.6	20.1	9.5	24.9	22.1	2.6	10.2	6.4	5.7	0
United Kingdom, England # †	125.4	17.3	10.2	34.3	13.1	3.3	9.0	7.5	15.4	15.2
United Kingdom, Northern Ireland # †	129.3	16.4	17.1	29.1	8.4	5.7	12.1	15.6	15.9	9.4
United Kingdom, Scotland †	107.2	20.4	8.9	31.2	13.2	3.2	12.0	3.5	9.3	5.6
United Kingdom, Wales # †	142.2	27.5	14.2	38.7	16.7	4.4	10.3	13.3	17.0	

Abbreviations used: DM: diabetes mellitus; HT / RVD: hypertension / renal vascular disease; Misc: miscellaneous renal disorders; Unkn: unknown
* Patients younger than 20 years of age are not reported
\# Values based on 1 to 5 patients are omitted
† The incident counts at day 91, on which the data presented in this table are based, are estimated (see methods)

Table B.3.7

Incident counts by treatment modality

at day 91

	All	Haemodialysis						Peritoneal dialysis				Kidney transplant				Unkn	Missing
		HD hospital/ centre	HD home	HD type Unkn	HF	HDF	Total HD/ HF/HDF	APD	CAPD	PD type Unkn	Total PD	Living donor	Deceased donor	Tx type Unkn	Total Tx		
	N	N	N	N	N	N	N	N	N	N	N	N	N	N	N	N	N
Austria §	959	723	0	2	1	105	831	41	41	1	83	30	15	0	45	0	0
Belgium, Dutch-speaking *	987	824	2	59			885			84	84	9	9	0	18	0	0
Belgium, French-speaking *	791	393	29	0	14	252	688	50	32	0	82	8	11	0	19	0	2
Bosnia and Herzegovina	344	280	1	0	0	48	329	0	13	0	13	2	0	0	2	0	0
Denmark #	692	426	0	0			426	82	89	0	171	36	59	0	95	0	0
Estonia †	83	69	0	0	0	0	69	10	0	0	10	0	4	0	4	0	0
France †	10241	6213	12	9	20	2450	8704	318	746	5	1069	203	260	0	463	4	0
Greece	2472	2224	0	0	0	130	2354	41	53	0	94	24	0	0	24	0	0
Iceland	48	32	0	0			32	0	8	0	8	0	8	0	8	0	0
Netherlands &	1860	1117	9	0		95	1221	206	134	0	340	254	44	0	298	1	0
Norway	542	296	0	0			296			187	187	20	38	0	58	1	0
Romania | |	3059	3010	0	0	0	4	3014	1	29	0	30	10	5	0	15	0	0
Serbia	507	409	0	4	0	40	453	6	43	0	49	4	0	0	4	1	0
Spain, Andalusia	1238	963	6	0			969			165	165	29	75	0	104	0	0
Spain, Aragon	194	120	1	0		30	151	9	21	0	30	6	7	0	13	0	0
Spain, Basque country	267	186	1	0			187	5	59	0	64	7	9	0	16	0	0
Spain, Canary Islands	400	284	1	0	0	0	285	0	0	99	99	7	9	0	16	0	0
Spain, Cantabria *	97	72	0	0			72	1	9	0	10	1	14	0	15	0	0
Spain, Castile and León * †	348	265	5	0			270	17	49	0	66	7	6	0	13	0	0
Spain, Castile-La Mancha *	230	174	0	0			174	5	42	0	47	1	6	1	8	1	0
Spain, Catalonia †	1386	343	17	0		643	1003	39	139	0	178	96	109	0	205	0	0
Spain, Community of Madrid	864	662	6	0		0	668	7	123	0	130	34	30	1	65	1	0
Spain, Extremadura	157	110	5	23			138	5	9	2	16	1	2	0	3	0	0
Spain, Galicia	417	306	7	0	0	0	313	24	60	0	84	12	6	1	19	1	0
Spain, La Rioja	39	26	0	0	0	0	26	7	6	0	13	0	0	0	0	0	0
Spain, Murcia	221	127	0	0	0	49	176	0	35	0	35	6	4	0	10	0	0
Spain, Navarre *	88	64	4	0			68	17	2	0	19	0	1	0	1	0	0
Spain, Valencian region	729	500	10	0			510	14	131	0	145	8	65	0	73	1	0
Sweden	1050	570	4	0			574			352	352	41	82	0	123	1	0
Switzerland †	748	589	5	0			594	36	52	0	88	39	19	0	58	8	0
United Kingdom, England # †	6222	3634	38		6	889	4567	610	566	10	1186	253	212		468	0	0
United Kingdom, Northern Ireland # †	225	152	0	0			155	22		0	24	29	17	0	46	0	0
United Kingdom, Scotland †	584	446	5	0			451	45	28	3	75	34	15	6	55	3	0
United Kingdom, Wales # †	449	294		0	0	57	354	35	27	0	62	18	16	0	33	0	0

Abbreviations used: HD: haemodialysis; Unkn: unknown; HF: haemofiltration; HDF: haemodiafiltration; APD: automated peritoneal dialysis; CAPD: continuous ambulatory peritoneal dialysis; PD: peritoneal dialysis; Tx: transplant
Categories may not add up due to rounding; When cells are left empty, (complete) data are unavailable
§ The incidence is underestimated by approximately 2% due to an estimated 2% underestimation of the incidence of haemodialysis
* Patients younger than 20 years of age are not reported
\# Values based on 1 to 5 patients are omitted
† The incidence counts at day 91 are estimated (see methods) and could result in decimal numbers that may not add up if rounded
& The incidence is underestimated by approximately 7%
| | The incidence of preemptive transplantation is underestimated by approximately 30%

Table B.3.8

Incidence per million population by treatment modality, unadjusted

at day 91

	All	Haemodialysis						Peritoneal dialysis				Kidney transplant				Unkn	Missing
		HD hospital/ centre	HD home	HD type Unkn	HF	HDF	Total HD/ HF/HDF	APD	CAPD	PD type Unkn	Total PD	Living donor	Deceased donor	Tx type Unkn	Total Tx		
	Pmp	Pmp	Pmp	Pmp	Pmp	Pmp	Pmp	Pmp	Pmp	Pmp	Pmp	Pmp	Pmp	Pmp	Pmp	Pmp	Pmp
Austria	107.5	81.0	0	0.2	0.1	11.8	93.1	4.6	4.6	0.1	9.3	3.4	1.7	0	5.0	0	0
Belgium, Dutch-speaking *	144.9	120.9	0.3	8.7			129.9			12.3	12.3	1.3	1.3	0	2.6	0	0
Belgium, French-speaking *	159.3	79.1	5.8	0	2.8	50.7	138.5	10.1	6.4	0	16.5	1.6	2.2	0	3.8	0	0.4
Bosnia and Herzegovina	97.4	79.3	0.3	0	0	13.6	93.2	0	3.7	0	3.7	0.6	0	0	0.6	0	0
Denmark #	116.4	71.6	0	0			71.6	13.8	15.0	0	28.8	6.1	9.9	0	16.0	0	0
Estonia †	63.5	52.7	0	0	0	0	52.7	7.9	0	0	7.9	0	3.1	0	2.8	0	0
France †	149.8	90.9	0.2	0.1	0.3	35.8	127.3	4.7	10.9	0.1	15.6	3.0	3.8	0	6.8	0.1	0
Greece	237.5	213.7	0	0	0	12.5	226.2	3.9	5.1	0	9.0	2.3	0	0	2.3	0	0
Iceland	124.5	83.0	0	0			83.0	0	20.7	0	20.7	0	20.7	0	20.7	0	0
Netherlands	111.9	67.2	0.5	0		5.7	73.4	12.4	8.1	0	20.5	15.3	2.6	0	17.9	0.1	0
Norway	98.2	53.6	0	0			53.6			33.9	33.9	3.6	6.9	0	10.5	0.2	0
Romania ||	160.5	157.9	0	0	0	0.2	158.1	0.1	1.5	0	1.6	0.5	0.3	0	0.8	0	0
Serbia	79.3	64.0	0	0.6	0	6.3	70.9	0.9	6.7	0	7.7	0.6	0	0	0.6	0.2	0
Spain, Andalusia	143.8	111.9	0.7	0			112.6			19.2	19.2	3.4	8.7	0	12.1	0	0
Spain, Aragon	144.1	89.1	0.7	0		22.3	112.1	6.7	15.6	0	22.3	4.5	5.2	0	9.7	0	0
Spain, Basque country	120.2	83.7	0.5	0			84.2	2.3	26.6	0	28.8	3.2	4.1	0	7.2	0	0
Spain, Canary Islands	179.7	127.6	0.4	0	0	0	128.0	0	0	44.5	44.5	3.1	4.0	0	7.2	0	0
Spain, Cantabria *	164.5	122.1	0	0			122.1	1.7	15.3	0	17.0	1.7	23.7	0	25.4	0	0
Spain, Castile and León * †	146.1	111.2	2.0	0			113.1	7.1	20.6	0	27.7	2.9	2.4	0	5.3	0	0
Spain, Castile-La Mancha *	109.8	83.1	0	0			83.1	2.4	20.1	0	22.4	0.5	2.9	0.5	3.8	0.5	0
Spain, Catalonia †	175.4	43.4	2.2	0		81.4	127.0	4.9	17.5	0	22.5	12.1	13.8	0	25.9	0	0
Spain, Community of Madrid	125.7	96.3	0.9	0		0	97.2	1.0	17.9	0	18.9	4.9	4.4	0.1	9.5	0.1	0
Spain, Extremadura	148.9	104.3	4.7	21.8			130.9	4.7	8.5	1.9	15.2	0.9	1.9	0	2.8	0	0
Spain, Galicia	154.3	113.2	2.6	0	0	0	115.8	8.9	22.2	0	31.1	4.4	2.2	0.4	7.0	0.4	0
Spain, La Rioja	120.7	80.4	0	0	0	0	80.4	21.7	18.6	0	40.2	0	0	0	0	0	0
Spain, Murcia	142.4	81.8	0	0	0	31.6	113.4	0	22.6	0	22.6	3.9	2.6	0	6.4	0	0
Spain, Navarre *	130.3	94.8	5.9	0			100.7	25.2	3.0	0	28.1	0	1.5	0	1.5	0	0
Spain, Valencian region	139.8	95.9	1.9	0			97.8	2.7	25.1	0	27.8	1.5	12.5	0	14.0	0.2	0
Sweden	99.7	54.1	0.4	0			54.5			33.4	33.4	3.9	7.8	0	11.7	0.1	0
Switzerland †	88.6	69.7	0.6	0			70.3	4.2	6.2	0	10.4	4.6	2.2	0	6.8	1.0	0
United Kingdom, England # †	117.2	68.5	0.7		0.1	16.7	86.1	11.5	10.7	0.2	22.4	4.8	4.0		8.8	0	0
United Kingdom, Northern Ireland # †	117.3	79.3	0	0			80.9	11.5		0	12.3	15.3	8.9	0	24.1	0	0
United Kingdom, Scotland †	106.4	81.2	1.0	0			82.1	8.2	5.0	0.5	13.7	6.2	2.7	1.2	10.1	0.5	0
United Kingdom, Wales # †	142.0	92.8		0	0	18.1	111.9	11.2	8.4	0	19.6	5.6	5.0	0	10.5	0	0

Abbreviations used: HD: haemodialysis; Unkn: unknown; HF: haemofiltration; HDF: haemodiafiltration; APD: automated peritoneal dialysis; CAPD: continuous ambulatory peritoneal dialysis; PD: peritoneal dialysis; Tx: transplant
Categories may not add up due to rounding; When cells are left empty, (complete) data are unavailable
* Patients younger than 20 years of age are not reported
# Values based on 1 to 5 patients are omitted
† The incident counts at day 91, on which the data presented in this table are based, are estimated (see methods)
|| The incidence of preemptive transplantation is underestimated by approximately 30%

Table B.3.9

Incidence per million population by treatment modality, adjusted

at day 91, adjusted for age and sex

	All	Haemodialysis						Peritoneal dialysis				Kidney transplant				Unkn	Missing
		HD hospital/ centre	HD home	HD type Unkn	HF	HDF	Total HD/ HF/HDF	APD	CAPD	PD type Unkn	Total PD	Living donor	Deceased donor	Tx type Unkn	Total Tx		
	Pmp	Pmp	Pmp	Pmp	Pmp	Pmp	Pmp	Pmp	Pmp	Pmp	Pmp	Pmp	Pmp	Pmp	Pmp	Pmp	Pmp
Austria	109.4	82.9	0	0.3	0.1	12.0	95.2	4.5	4.5	0.1	9.2	3.3	1.7	0	5.0	0	0
Belgium, Dutch-speaking *	142.7	119.0	0.3	8.5			127.8			12.2	12.2	1.3	1.4	0	2.7	0	0
Belgium, French-speaking *	182.8	91.0	6.4	0	3.2	58.5	159.1	11.5	7.5	0	19.0	1.7	2.5	0	4.2	0	0.5
Bosnia and Herzegovina	124.2	102.3	0.3	0	0	17.3	119.9	0	3.8	0	3.8	0.5	0	0	0.5	0	0
Denmark #	117.4	72.0	0	0			72.0	13.9	14.9	0	28.8	6.2	10.5	0	16.6	0	0
Estonia †	68.8	57.8	0	0	0	0	57.8	8.3	0	0	8.3	0	3.2	0	3.2	0	0
France †	153.0	92.8	0.2	0.2	0.3	36.4	129.9	4.8	11.1	0.1	16.0	3.1	4.0	0	7.1	0.1	0
Greece	217.3	194.8	0	0	0	11.8	206.6	3.7	4.8	0	8.4	2.3	0	0	2.3	0	0
Iceland	154.1	103.3	0	0			103.3	0	26.8	0	26.8	0	24.0	0	24.0	0	0
Netherlands	112.1	67.1	0.5	0		5.7	73.3	12.4	8.1	0	20.5	15.5	2.6	0	18.1	0.1	0
Norway	103.6	56.7	0	0			56.7			36.1	36.1	3.6	7.0	0	10.6	0.2	0
Romania ||	167.5	164.9	0	0	0	0.2	165.1	0	1.5	0	1.6	0.5	0.2	0	0.8	0	0
Serbia	75.8	61.2	0	0.6	0	6.0	67.8	1.0	6.2	0	7.2	0.6	0	0	0.6	0.2	0
Spain, Andalusia	151.5	118.8	0.7	0			119.5			20.0	20.0	3.3	8.7	0	12.1	0	0
Spain, Aragon	137.8	84.4	0.6	0		21.9	106.9	6.5	14.7	0	21.2	4.2	5.5	0	9.7	0	0
Spain, Basque country	111.6	77.9	0.4	0			78.4	2.1	24.4	0	26.5	2.8	3.9	0	6.7	0	0
Spain, Canary Islands	186.8	133.3	0.4	0	0	0	133.7	0	0	46.3	46.3	2.9	3.8	0	6.8	0	0
Spain, Cantabria *	150.6	110.9	0	0			110.9	1.5	15.0	0	16.5	2.0	21.2	0	23.2	0	0
Spain, Castile and León * †	125.3	94.1	1.7	0			95.8	6.6	18.1	0	24.7	2.7	2.0	0	4.7	0	0
Spain, Castile-La Mancha *	110.7	84.1	0	0			84.1	2.3	20.0	0	22.3	0.5	2.9	0.5	3.8	0.5	0
Spain, Catalonia †	185.2	46.7	2.3	0		85.6	134.6	5.3	18.6	0	23.9	12.4	14.3	0	26.7	0	0
Spain, Community of Madrid	133.7	103.0	0.8	0		0	103.8	1.0	19.1	0	20.0	5.0	4.6	0.1	9.7	0.1	0
Spain, Extremadura	140.1	97.8	4.4	20.7			122.9	4.7	8.3	1.6	14.7	0.9	1.6	0	2.5	0	0
Spain, Galicia	134.6	96.9	2.4	0	0	0	99.4	8.1	19.7	0	27.8	4.5	2.1	0.5	7.1	0.4	0
Spain, La Rioja	118.5	79.6	0	0	0	0	79.6	20.9	18.0	0	38.9	0	0	0	0	0	0
Spain, Murcia	163.4	95.2	0	0	0	36.6	131.8	0	25.0	0	25.0	3.8	2.8	0	6.6	0	0
Spain, Navarre *	128.2	92.3	6.3	0			98.6	25.4	2.6	0	28.0	0	1.6	0	1.6	0	0
Spain, Valencian region	141.1	97.5	1.8	0			99.3	2.6	25.4	0	28.0	1.5	12.1	0	13.6	0.2	0
Sweden	101.6	55.1	0.4	0			55.5			33.5	33.5	4.1	8.5	0	12.6	0.1	0
Switzerland †	91.3	72.3	0.7	0			72.9	4.2	6.5	0	10.6	4.5	2.2	0	6.8	1.1	0
United Kingdom, England # †	125.4	73.5	0.8		0.1	18.1	92.5	12.1	11.4	0.2	23.7	4.9	4.3		9.2	0	0
United Kingdom, Northern Ireland # †	129.3	88.0	0	0			89.7	12.8		0	13.8	16.1	9.7	0	25.9	0	0
United Kingdom, Scotland †	107.2	81.6	1.0	0			82.6	8.1	5.2	0.6	13.6	6.5	2.7	1.3	10.5	0.5	0
United Kingdom, Wales # †	142.2	92.1		0	0	18.2	111.1	11.6	8.7	0	19.9	5.8	5.3	0	11.1	0	0

Abbreviations used: HD: haemodialysis; Unkn: unknown; HF: haemofiltration; HDF: haemodiafiltration; APD: automated peritoneal dialysis; CAPD: continuous ambulatory peritoneal dialysis; PD: peritoneal dialysis; Tx: transplant
Categories may not add up due to rounding; When cells are left empty, (complete) data are unavailable
* Patients younger than 20 years of age are not reported
\# Values based on 1 to 5 patients are omitted
† The incident counts at day 91, on which the data presented in this table are based, are estimated (see methods)
|| The incidence of preemptive transplantation is underestimated by approximately 30%

Table B.3.10

Treatment modality distribution, unadjusted

at day 91

	All	Haemodialysis						Peritoneal dialysis				Kidney transplant				Unkn	Missing
		HD hospital/ centre	HD home	HD type Unkn	HF	HDF	Total HD/ HF/HDF	APD	CAPD	PD type Unkn	Total PD	Living donor	Deceased donor	Tx type Unkn	Total Tx		
	%	%	%	%	%	%	%	%	%	%	%	%	%	%	%	%	%
Austria	100	75.4	0	0.2	0.1	10.9	86.7	4.3	4.3	0.1	8.7	3.1	1.6	0	4.7	0	0
Belgium, Dutch-speaking *	100	83.5	0.2	6.0			89.7			8.5	8.5	0.9	0.9	0	1.8	0	0
Belgium, French-speaking *	100	49.7	3.7	0	1.8	31.9	87.0	6.3	4.0	0	10.4	1.0	1.4	0	2.4	0	0.3
Bosnia and Herzegovina	100	81.4	0.3	0	0	14.0	95.6	0	3.8	0	3.8	0.6	0	0	0.6	0	0
Denmark #	100	61.6	0	0			61.6	11.8	12.9	0	24.7	5.2	8.5	0	13.7	0	0
Estonia †	100	83.1	0	0	0	0	83.1	12.5	0	0	12.5	0	4.8	0	4.4	0	0
France †	100	60.7	0.1	0.1	0.2	23.9	85.0	3.1	7.3	0	10.4	2.0	2.5	0	4.5	0	0
Greece	100	90.0	0	0	0	5.3	95.2	1.7	2.1	0	3.8	1.0	0	0	1.0	0	0
Iceland	100	66.7	0	0			66.7	0	16.7	0	16.7	0	16.7	0	16.7	0	0
Netherlands	100	60.1	0.5	0		5.1	65.6	11.1	7.2	0	18.3	13.7	2.4	0	16.0	0.1	0
Norway	100	54.6	0	0			54.6			34.5	34.5	3.7	7.0	0	10.7	0.2	0
Romania ǀǀ	100	98.4	0	0	0	0.1	98.5	0	0.9	0	1.0	0.3	0.2	0	0.5	0	0
Serbia	100	80.7	0	0.8	0	7.9	89.3	1.2	8.5	0	9.7	0.8	0	0	0.8	0.2	0
Spain, Andalusia	100	77.8	0.5	0			78.3			13.3	13.3	2.3	6.1	0	8.4	0	0
Spain, Aragon	100	61.9	0.5	0		15.5	77.8	4.6	10.8	0	15.5	3.1	3.6	0	6.7	0	0
Spain, Basque country	100	69.7	0.4	0			70.0	1.9	22.1	0	24.0	2.6	3.4	0	6.0	0	0
Spain, Canary Islands	100	71.0	0.3	0	0	0	71.3	0	0	24.8	24.8	1.8	2.3	0	4.0	0	0
Spain, Cantabria *	100	74.2	0	0			74.2	1.0	9.3	0	10.3	1.0	14.4	0	15.5	0	0
Spain, Castile and León * †	100	76.1	1.3	0			77.4	4.9	14.1	0	18.9	2.0	1.6	0	3.6	0	0
Spain, Castile-La Mancha *	100	75.7	0	0			75.7	2.2	18.3	0	20.4	0.4	2.6	0.4	3.5	0.4	0
Spain, Catalonia †	100	24.7	1.3	0		46.4	72.4	2.8	10.0	0	12.8	6.9	7.9	0	14.8	0	0
Spain, Community of Madrid	100	76.6	0.7	0		0	77.3	0.8	14.2	0	15.0	3.9	3.5	0.1	7.5	0.1	0
Spain, Extremadura	100	70.1	3.2	14.6			87.9	3.2	5.7	1.3	10.2	0.6	1.3	0	1.9	0	0
Spain, Galicia	100	73.4	1.7	0	0	0	75.1	5.8	14.4	0	20.1	2.9	1.4	0.2	4.6	0.2	0
Spain, La Rioja	100	66.7	0	0	0	0	66.7	17.9	15.4	0	33.3	0	0	0	0	0	0
Spain, Murcia	100	57.5	0	0	0	22.2	79.6	0	15.8	0	15.8	2.7	1.8	0	4.5	0	0
Spain, Navarre *	100	72.7	4.5	0			77.3	19.3	2.3	0	21.6	0	1.1	0	1.1	0	0
Spain, Valencian region	100	68.6	1.4	0			70.0	1.9	18.0	0	19.9	1.1	8.9	0	10.0	0.1	0
Sweden	100	54.3	0.4	0			54.7			33.5	33.5	3.9	7.8	0	11.7	0.1	0
Switzerland †	100	78.7	0.7	0			79.4	4.8	7.0	0	11.8	5.2	2.5	0	7.7	1.1	0
United Kingdom, England # †	100	58.4	0.6		0.1	14.3	73.4	9.8	9.1	0.2	19.1	4.1	3.4		7.5	0	0
United Kingdom, Northern Ireland # †	100	67.6	0	0			68.9	9.8		0	10.5	13.0	7.5	0	20.6	0	0
United Kingdom, Scotland †	100	76.3	0.9	0			77.2	7.7	4.7	0.5	12.9	5.8	2.6	1.1	9.5	0.5	0
United Kingdom, Wales # †	100	65.4		0	0	12.8	78.8	7.9	5.9	0	13.8	3.9	3.5	0	7.4	0	0

Abbreviations used: HD: haemodialysis; Unkn: unknown; HF: haemofiltration; HDF: haemodiafiltration; APD: automated peritoneal dialysis; CAPD: continuous ambulatory peritoneal dialysis; PD: peritoneal dialysis; Tx: transplant
Categories may not add up due to rounding; When cells are left empty, (complete) data are unavailable
* Patients younger than 20 years of age are not reported
# Values based on 1 to 5 patients are omitted
† The incident counts at day 91, on which the data presented in this table are based, are estimated (see methods)
ǀǀ The incidence of preemptive transplantation is underestimated by approximately 30%

FIND

Table B.3.11

Treatment modality distribution by age, sex, and primary renal disease, unadjusted

at day 91

	All				0-19				20-44				45-64				65-74				75+				Male				Female				DM				Non-DM			
	HD	PD	Tx	Un	HD	PD	Tx	Un	HD	PD	Tx	Un	HD	PD	Tx	Un	HD	PD	Tx	Un	HD	PD	Tx	Un	HD	PD	Tx	Un	HD	PD	Tx	Un	HD	PD	Tx	Un	HD	PD	Tx	Un
	%	%	%	%	%	%	%	%	%	%	%	%	%	%	%	%	%	%	%	%	%	%	%	%	%	%	%	%	%	%	%	%	%	%	%	%	%	%	%	%
Austria	87	9	5	0	0	21	79	0	73	16	11	0	82	12	5	0	92	5	3	0	95	5	0	0	87	9	5	0	87	8	5	0	92	8	0	0	85	9	6	0
Belgium, Dutch-speaking *	90	9	2	0					79	11	10	0	82	14	4	0	88	11	1	0	96	4	0	0	89	9	2	0	90	8	2	0	92	6	1	0	89	9	2	0
Belgium, French-speaking *	87	10	2	0					87	6	5	2	83	11	6	0	88	11	1	0	89	10	1	0	87	10	2	0	87	11	3	0	87	13	0	0	87	10	3	0
Bosnia and Herzegovina	96	4	1	0	75	25	0	0	94	3	3	0	92	7	1	0	97	3	0	0	100	0	0	0	99	1	0	0	91	8	1	0	99	1	0	0	94	5	1	0
Denmark #	62	25	14	0				0	53	25	22	0	60	18	22	0	64	26	10	0	67	31		0	63	25	13	0	60	25	16	0	73	22	6	0	58	26	17	0
Estonia †	83	13	4	0	0	0	0	0	72	21	8	0	75	16	9	0	87	13	0	0	100	0	0	0	87	10	3	0	78	16	7	0	87	4	9	0	82	14	3	0
France †	85	10	5	0	52	29	19	0	73	12	15	0	82	10	8	0	88	10	2	0	89	10	1	0	85	10	4	0	85	11	5	0	90	8	2	0	84	11	5	0
Greece	95	4	1	0	67	0	33	0	84	5	11	0	94	5	1	0	95	4	0	0	97	3	0	0	95	4	1	0	95	4	1	0	97	3	0	0	95	4	1	0
Iceland	67	17	17	0	0	0	0	0	71	0	29	0	44	31	25	0	80	0	20	0	80	20	0	0	65	19	16	0	73	9	18	0	100	0	0	0	63	19	19	0
Netherlands	66	18	16	0	21	41	38	0	51	18	31	0	61	18	21	0	68	19	13	0	80	16	4	0	65	19	15	0	66	17	17	0	78	19	4	0	62	18	20	0
Norway	55	35	11	0	25	17	50	8	38	39	23	0	61	27	12	0	50	41	9	0	62	36	2	0	55	36	9	0	55	31	14	0	55	41	4	0	55	33	12	0
Romania | |	99	1	0	0	85	11	4	0	94	2	4	0	98	1	0	0	100	0	0	0	100	0	0	0	99	1	0	0	98	1	1	0	99	1	0	0	98	1	1	0
Serbia	89	10	1	0	44	56	0	0	78	14	8	0	92	8	0	0	91	9	0	0	91	7	0	1	91	7	1	0	85	14	1	0	89	9	2	0	89	10	1	0
Spain, Andalusia	78	13	8	0	58	8	33	0	58	22	20	0	75	14	11	0	79	14	7	0	89	9	2	0	77	14	9	0	81	12	6	0	88	8	4	0	75	15	10	0
Spain, Aragon	78	15	7	0	25	0	75	0	56	19	25	0	76	16	7	0	79	18	4	0	87	13	0	0	79	16	5	0	74	15	11	0	78	20	2	0	78	14	8	0
Spain, Basque country	70	24	6	0	0	0	0	0	70	22	9	0	65	25	10	0	73	21	6	0	72	27	1	0	70	24	6	0	71	23	6	0	74	22	5	0	69	25	6	0
Spain, Canary Islands	71	25	4	0	50	50	0	0	61	18	20	0	71	26	3	0	67	31	2	0	79	20	1	0	71	25	3	0	71	24	5	0	78	19	2	0	68	27	5	0
Spain, Cantabria *	74	10	15	0					43	14	43	0	58	11	32	0	74	10	16	0	88	10	3	0	76	9	14	0	67	14	19	0	65	12	24	0	76	10	14	0
Spain, Castile and León * †	77	19	4	0					58	33	9	0	69	24	7	0	78	19	2	0	87	12	1	0	78	19	4	0	77	20	3	0	83	17	0	0	76	20	5	0
Spain, Castile-La Mancha *	76	20	3	0					50	36	11	4	73	23	4	0	81	17	2	0	83	15	1	0	78	20	1	1	70	21	9	0	88	12	0	0	71	24	5	1
Spain, Catalonia †	72	13	15	0	27	10	63	0	51	16	34	0	70	14	17	0	68	16	17	0	85	9	6	0	72	13	15	0	73	13	14	0	79	14	7	0	70	13	17	0
Spain, Community of Madrid	77	15	8	0	43	6	51	0	67	18	15	0	76	17	7	0	78	17	5	0	87	12	1	0	79	14	7	0	74	18	8	0	88	10	2	0	75	16	9	0
Spain, Extremadura	88	10	2	0	0	0	0	0	53	40	7	0	80	16	4	0	96	4	0	0	98	2	0	0	88	9	3	0	88	12	0	0	95	2	2	0	85	13	2	0
Spain, Galicia	75	20	5	0	0	0	0	0	53	21	26	0	66	29	5	1	75	21	3	0	89	11	0	0	76	19	4	0	72	23	5	0	79	21	0	0	74	20	6	0
Spain, La Rioja	67	33	0	0	0	0	0	0	100	0	0	0	56	44	0	0	56	44	0	0	86	14	0	0	71	29	0	0	60	40	0	0	75	25	0	0	65	35	0	0
Spain, Murcia	80	16	5	0	25	25	50	0	75	14	11	0	74	21	6	0	83	16	2	0	89	11	0	0	81	15	4	0	77	17	6	0	80	18	2	0	80	15	5	0
Spain, Navarre *	77	22	1	0					67	22	11	0	74	26	0	0	76	24	0	0	85	15	0	0	79	19	1	0	70	30	0	0	75	25	0	0	78	21	1	0
Spain, Valencian region	70	20	10	0	67	11	22	0	61	22	17	0	63	19	18	0	65	26	9	0	82	16	2	0	69	21	9	0	71	17	12	0	79	15	6	0	68	21	11	0
Sweden	55	34	12	0	0	61	39	0	54	27	19	0	52	29	19	0	55	33	11	0	60	39	1	0	55	34	11	0	55	32	13	0	58	36	6	0	53	33	14	0
Switzerland †	79	12	8	1	26	26	22	26	62	17	19	2	66	15	17	2	86	10	3	1	91	9	0	0	80	11	7	1	77	14	9	0	90	8	2	0	77	13	9	1
United Kingdom, England # †	73	19	8	0	38	35	27	0	62	24	13	0	73	18	9	0	77	18	6	0	83	16	1	0	74	19	7	0	72	18	9	0	79	17	4	0	71	20	9	0
United Kingdom, Northern Ireland # †	69	11	21	0		0		0	47		42	0	64	10	26	0	77		15	0	82	16		0	68	9	22	0	70	12	18	0	82			0	65	11	24	0
United Kingdom, Scotland †	77	13	9	0	67	17	17	0	63	21	16	0	72	13	14	1	83	11	5	0	93	7	0	0	78	11	11	1	77	16	7	0	86	10	4	0	74	14	12	1
United Kingdom, Wales # †	79	14	7	0				0	52	22	26	0	77	14	8	0	83	14		0	92	8	0	0	79	13	8	0	78	14	7	0	88	9		0	75	15	9	0

Abbreviations used: HD: haemodialysis; PD: peritoneal dialysis; Tx: transplant; Un: unknown; DM: diabetes mellitus
Categories may not add up due to missing values or rounding
* Patients younger than 20 years of age are not reported
# Values based on 1 to 5 patients are omitted
† The incident counts at day 91, on which the data presented in this table are based, are estimated (see methods)
| | The incidence of preemptive transplantation is underestimated by approximately 30%

Table B.4.1

Prevalent counts and percentages by age and sex

prevalent patients on December 31

	All			0-19						20-44						45-64						65-74						75+					
	All	Male	Female	All		Male		Female		All		Male		Female		All		Male		Female		All		Male		Female		All		Male		Female	
	N (100%)	N (100%)	N (100%)	N	%	N	%	N	%	N	%	N	%	N	%	N	%	N	%	N	%	N	%	N	%	N	%	N	%	N	%	N	%
Austria §	8985	5766	3219	88	1	56	1	32	1	1143	13	700	12	443	14	3561	40	2307	40	1254	39	2234	25	1444	25	790	25	1959	22	1259	22	700	22
Belgium, Dutch-speaking *	8676	5374	3302							832	10	498	9	334	10	2695	31	1693	32	1002	30	2251	26	1419	26	832	25	2898	33	1764	33	1134	34
Belgium, French-speaking *	7242	4442	2800							670	9	379	9	291	10	2317	32	1478	33	839	30	2006	28	1223	28	783	28	2249	31	1362	31	887	32
Bosnia and Herzegovina	2482	1531	951	14	1	8	1	6	1	369	15	241	16	128	13	1032	42	661	43	371	39	679	27	412	27	267	28	388	16	209	14	179	19
Denmark #	6056	3778	2278	74	1	50	1	24	1	955	16	590	16	365	16	2532	42	1580	42	952	42	1353	22	832	22	521	23	1142	19	726	19	416	18
Estonia	1092	634	458	11	1	5	1	6	1	172	16	100	16	72	16	470	43	285	45	185	40	238	22	127	20	111	24	201	18	117	18	84	18
France	96317	59914	36403	969	1	590	1	379	1	12175	13	7444	12	4731	13	33171	34	20603	34	12568	35	24702	26	15454	26	9248	25	25300	26	15823	26	9477	26
Greece	15583	10361	5220	72	0	49	0	23	0	1344	9	887	9	457	9	5080	33	3404	33	1676	32	4025	26	2723	26	1302	25	5060	32	3298	32	1762	34
Iceland	340	224	116	8	2	6	3	2	2	64	19	39	17	25	22	139	41	88	39	51	44	66	19	43	19	23	20	63	19	48	21	15	13
Netherlands &	18342	11202	7140	208	1	127	1	81	1	2589	14	1584	14	1005	14	7147	39	4368	39	2779	39	4737	26	2845	25	1892	26	3661	20	2278	20	1383	19
Norway	5605	3632	1973	86	2	55	2	31	2	854	15	537	15	317	16	2209	39	1399	39	810	41	1390	25	925	25	465	24	1066	19	716	20	350	18
Romania	25442	14677	10765	146	1	78	1	68	1	2645	10	1557	11	1088	10	8623	34	5308	36	3315	31	7220	28	4090	28	3130	29	6808	27	3644	25	3164	29
Serbia	5881	3675	2206	64	1	45	1	19	1	765	13	496	13	269	12	2276	39	1385	38	891	40	1749	30	1121	31	628	28	1024	17	625	17	399	18
Spain, Andalusia	11676	7236	4440	126	1	75	1	51	1	1458	12	892	12	566	13	4691	40	2959	41	1732	39	2807	24	1788	25	1019	23	2594	22	1522	21	1072	24
Spain, Aragon	2086	1398	688	16	1	9	1	7	1	173	8	115	8	58	8	693	33	432	31	261	38	519	25	371	27	148	22	685	33	471	34	214	31
Spain, Basque country	2964	1902	1062	32	1	17	1	15	1	361	12	218	11	143	13	1110	37	706	37	404	38	799	27	530	28	269	25	662	22	431	23	231	22
Spain, Canary Islands	3581	2360	1221	9	0	6	0	3	0	370	10	226	10	144	12	1486	41	986	42	500	41	913	25	618	26	295	24	803	22	524	22	279	23
Spain, Cantabria *	751	514	237							73	10	46	9	27	11	277	37	189	37	88	37	201	27	142	28	59	25	200	27	137	27	63	27
Spain, Castile and León *	3300	2195	1105							274	8	180	8	94	9	1168	35	765	35	403	36	866	26	586	27	280	25	992	30	664	30	328	30
Spain, Castile-La Mancha *	2628	1649	979							241	9	141	9	100	10	1040	40	656	40	384	39	640	24	430	26	210	21	707	27	422	26	285	29
Spain, Catalonia	12267	7877	4390	150	1	90	1	60	1	1258	10	783	10	475	11	4643	38	2994	38	1649	38	3058	25	1995	25	1063	24	3158	26	2015	26	1143	26
Spain, Community of Madrid	8602	5450	3133	121	1	83	2	35	1	1016	12	582	11	428	14	3245	38	2046	38	1194	38	2028	24	1320	24	707	23	2192	25	1419	26	769	25
Spain, Extremadura	1458	928	530	6	0	3	0	3	1	133	9	81	9	52	10	577	40	352	38	225	42	388	27	260	28	128	24	354	24	232	25	122	23
Spain, Galicia	4087	2647	1440	20	0	13	0	7	0	361	9	222	8	139	10	1555	38	1001	38	554	38	1087	27	704	27	383	27	1064	26	707	27	357	25
Spain, La Rioja	399	254	145	1	0	1	0	0	0	43	11	30	12	13	9	176	44	114	45	62	43	92	23	59	23	33	23	87	22	50	20	37	26
Spain, Murcia	2239	1443	796	22	1	17	1	5	1	242	11	143	10	99	12	885	40	579	40	306	38	550	25	357	25	193	24	540	24	347	24	193	24
Spain, Navarre *	948	616	332							113	12	59	10	54	16	350	37	227	37	123	37	262	28	184	30	78	23	223	24	146	24	77	23
Spain, Valencian region	7753	4940	2813	67	1	46	1	21	1	816	11	538	11	278	10	2754	36	1738	35	1016	36	1957	25	1243	25	714	25	2159	28	1375	28	784	28
Sweden	10649	6874	3775	166	2	93	1	73	2	1642	15	1050	15	592	16	4098	38	2649	39	1449	38	2504	24	1608	23	896	24	2239	21	1474	21	765	20
Switzerland	8659	5486	3173	94	1	60	1	34	1	1076	12	675	12	401	13	3053	35	1955	36	1098	35	2176	25	1354	25	822	26	2260	26	1442	26	818	26
United Kingdom, England #	55070	33907	21163	1054	2	631	2	423	2	10113	18	6142	18	3971	19	23431	43	14382	42	9049	43	11641	21	7185	21	4456	21	8831	16	5567	16	3264	15
United Kingdom, Northern Ireland #	2123	1299	824	38	2	27	2	11	1	387	18	233	18	154	19	914	43	567	44	347	42	405	19	246	19	159	19	379	18	226	17	153	19
United Kingdom, Scotland	5725	3478	2237	85	1	60	2	25	1	1030	18	648	19	382	17	2549	45	1509	43	1040	46	1324	23	803	23	521	23	727	13	458	13	269	12
United Kingdom, Wales #	3498	2194	1304	54	2	32	1	22	2	675	19	415	19	260	20	1394	40	866	39	528	40	738	21	469	21	269	21	637	18	412	19	225	17

Categories may not add up due to missing values or rounding
§ The prevalence is underestimated by approximately 2% due to one haemodialysis centre not submitting data
* Patients younger than 20 years of age are not reported
# Values based on 1 to 5 patients are omitted
& The prevalence is underestimated by approximately 4%

Table B.4.2

Prevalence per million (age-related) population by age and sex, unadjusted

prevalent patients on December 31

	All			0-19			20-44			45-64			65-74			75+		
	All	Male	Female	All	Male	Female	All	Male	Female	All	Male	Female	All	Male	Female	All	Male	Female
	Pmp	Pmp	Pmp	Pmarp	Pmarp	Pmarp	Pmarp	Pmarp	Pmarp	Pmarp	Pmarp	Pmarp	Pmarp	Pmarp	Pmarp	Pmarp	Pmarp	Pmarp
Austria	1007.0	1311.9	711.0	51.0	63.0	38.2	393.8	472.9	311.6	1397.0	1826.9	974.9	2494.9	3449.4	1656.9	2305.6	3650.0	1386.9
Belgium, Dutch-speaking *	1273.4	1591.5	960.8				398.0	472.9	322.0	1489.8	1858.1	1116.0	3024.5	3903.8	2185.0	4132.9	5960.7	2798.2
Belgium, French-speaking *	1458.2	1821.0	1108.0				404.0	454.6	352.8	1817.9	2321.6	1315.2	4140.4	5421.5	3024.2	5566.9	8674.6	3591.3
Bosnia and Herzegovina	702.9	883.8	528.7	17.8	19.8	15.7	301.6	387.2	213.0	1012.5	1330.6	710.0	2309.3	3195.8	1617.2	1865.6	2605.2	1401.2
Denmark #	1018.3	1277.9	761.7	57.3	75.4	38.1	508.1	618.3	394.5	1630.6	2038.6	1224.1	2169.8	2759.5	1617.8	1907.0	2763.7	1237.5
Estonia	838.9	1027.5	668.8	39.1	34.6	43.7	413.5	466.2	357.5	1385.4	1730.1	1060.1	1659.1	2194.2	1297.2	1653.5	3288.2	977.0
France	1408.7	1807.8	1033.3	60.9	72.3	48.9	594.1	734.1	456.9	1905.5	2417.5	1414.4	3243.2	4374.7	2264.5	3648.7	5687.2	2282.6
Greece	1497.3	2034.1	982.4	37.6	49.6	24.8	445.2	575.1	309.5	1657.1	2273.1	1068.8	3376.6	4876.9	2054.7	4160.1	6496.5	2486.4
Iceland	881.6	1129.9	619.0	84.8	123.2	43.8	450.9	518.8	374.4	1538.8	1917.4	1147.8	1935.3	2528.5	1345.2	2527.6	4181.5	1115.6
Netherlands	1068.8	1313.2	827.2	57.9	69.0	46.2	471.3	570.7	369.8	1559.5	1912.4	1208.8	2513.2	3077.2	1970.2	2275.6	3227.6	1531.6
Norway	1015.5	1306.2	720.3	69.2	86.1	51.3	465.2	571.7	353.6	1558.7	1939.1	1164.3	2540.3	3421.7	1679.7	2236.5	3394.4	1317.3
Romania	1334.8	1583.7	1099.2	35.3	36.7	33.8	458.6	523.1	389.7	1606.1	1998.3	1222.0	3132.5	4159.8	2368.3	4597.4	6935.7	3311.6
Serbia	920.1	1182.4	671.9	51.4	70.2	31.4	393.5	502.7	280.9	1280.3	1597.9	978.2	1949.1	2770.2	1274.7	1942.1	2984.6	1255.3
Spain, Andalusia	1356.4	1706.8	1016.4	73.0	84.3	60.9	544.8	657.8	428.8	1794.4	2287.5	1311.4	3328.4	4480.0	2293.8	3471.2	5044.8	2405.8
Spain, Aragon	1549.3	2100.8	1010.3	64.9	70.8	58.6	443.9	576.5	304.9	1688.3	2082.7	1285.4	3581.7	5369.7	1952.2	4421.1	7543.8	2313.4
Spain, Basque country	1333.9	1760.3	930.4	81.3	83.9	78.6	591.6	705.7	474.6	1601.7	2055.3	1155.9	3088.6	4381.5	1953.1	2482.1	4124.4	1424.1
Spain, Canary Islands	1608.8	2147.6	1083.4	23.9	30.9	16.4	507.6	621.0	394.6	2040.9	2706.5	1374.3	4218.0	5964.8	2614.2	4561.5	7168.2	2710.4
Spain, Cantabria *	1273.7	1798.9	779.9				454.1	569.0	337.8	1468.6	2033.4	919.9	2770.0	4140.9	1541.7	2963.6	5216.9	1528.2
Spain, Castile and León *	1384.4	1870.3	913.1				438.6	563.0	308.2	1554.3	2026.6	1077.6	2930.7	4007.0	1876.0	2955.5	4861.3	1647.8
Spain, Castile-La Mancha *	1254.9	1570.2	937.7				378.2	427.6	325.2	1631.0	2019.7	1227.4	3175.6	4360.4	2040.4	3393.6	4909.6	2328.9
Spain, Catalonia	1552.4	2026.4	1093.5	96.6	112.2	79.8	503.4	617.6	385.9	2000.6	2581.8	1420.2	3968.1	5596.4	2566.6	4163.4	6685.9	2500.4
Spain, Community of Madrid	1251.8	1657.4	874.2	90.7	121.3	53.8	456.3	531.2	378.4	1587.3	2082.2	1124.5	3180.2	4655.6	1996.2	3485.8	5857.9	1989.2
Spain, Extremadura	1382.9	1779.1	995.0	31.8	30.9	32.8	431.9	516.1	344.3	1761.2	2134.9	1382.5	3438.9	4669.0	2240.1	3014.3	4916.4	1736.6
Spain, Galicia	1512.2	2035.5	1026.9	47.8	60.4	34.5	499.2	610.4	386.6	1837.2	2417.7	1281.3	3272.3	4519.5	2171.0	2779.8	4659.1	1545.4
Spain, La Rioja	1234.4	1591.8	885.9	16.4	31.9	0	466.8	645.6	284.8	1773.4	2282.1	1257.8	2638.1	3469.8	1846.8	2412.3	3391.0	1735.5
Spain, Murcia	1442.9	1855.2	1028.6	64.1	95.7	30.2	480.6	550.9	405.8	1961.8	2536.8	1373.0	4130.6	5664.1	2752.2	4481.3	7043.8	2709.3
Spain, Navarre *	1403.9	1843.0	973.6				567.7	584.8	550.1	1734.1	2225.8	1231.9	3803.2	5527.8	2190.8	3180.2	5113.5	1852.3
Spain, Valencian region	1486.3	1925.2	1061.4	66.9	89.1	43.3	517.9	676.1	356.5	1730.4	2190.6	1272.9	3590.0	4866.2	2464.7	4297.3	6707.7	2636.0
Sweden	1010.7	1295.7	721.6	68.5	74.6	62.0	485.7	601.8	361.9	1594.0	2035.7	1141.2	2356.2	3078.1	1658.2	2038.8	3010.2	1257.1
Switzerland	1025.4	1307.8	746.7	55.8	69.3	41.6	385.0	474.8	292.0	1304.3	1666.3	940.5	2661.8	3453.2	1932.3	2797.3	4219.0	1754.9
United Kingdom, England #	1037.6	1303.1	782.2	85.8	100.3	70.7	579.1	715.4	447.3	1747.1	2183.4	1325.9	2312.0	2966.1	1705.5	1807.6	2611.9	1185.2
United Kingdom, Northern Ireland #	1105.5	1373.9	845.2	78.7	109.1	46.8	644.3	783.5	507.8	1848.7	2342.9	1374.9	2219.9	2752.2	1708.6	2368.2	3269.7	1682.8
United Kingdom, Scotland	1042.8	1303.1	792.9	75.0	103.2	45.2	586.7	753.1	426.7	1717.6	2099.1	1359.3	2193.3	2777.8	1656.2	1417.9	2088.8	916.7
United Kingdom, Wales #	1105.4	1412.8	809.2	77.7	89.5	65.1	695.9	861.6	532.4	1706.7	2177.3	1260.1	2101.3	2759.1	1484.3	1924.3	2818.8	1217.1

* Patients younger than 20 years of age are not reported
# Values based on 1 to 5 patients are omitted

Table B.4.3

Sex, mean age, and median age

prevalent patients on December 31

	All						Male						Female					
	%	Mean (years)	SD	Median (years)	P25	P75	%	Mean (years)	SD	Median (years)	P25	P75	%	Mean (years)	SD	Median (years)	P25	P75
Austria	100	62.3	15.4	64.0	53.4	73.8	64.2	62.4	15.4	64.1	53.8	73.7	35.8	62.0	15.6	63.7	52.5	73.9
Belgium, Dutch-speaking *	100	66.8	15.0	68.7	57.6	78.0	61.9	66.7	14.8	68.6	57.6	77.8	38.1	66.9	15.2	68.8	57.7	78.3
Belgium, French-speaking *	100	66.6	14.8	68.5	57.5	77.1	61.3	66.4	14.5	68.0	57.3	76.8	38.7	66.8	15.3	69.0	57.6	77.5
Bosnia and Herzegovina	100	60.7	14.6	62.5	51.0	71.4	61.7	59.9	14.6	62.0	50.2	70.4	38.3	62.0	14.6	63.6	52.4	73.0
Denmark	100	59.9	15.8	61.2	50.1	72.2	62.4	59.9	15.9	61.1	50.3	72.5	37.6	59.8	15.7	61.4	50.0	71.8
Estonia	100	60.6	15.8	61.9	50.9	72.1	58.1	60.5	15.6	61.9	51.0	72.0	41.9	60.6	16.0	62.4	50.7	72.4
France	100	63.6	16.1	65.9	53.6	75.5	62.2	63.7	16.0	66.0	53.7	75.5	37.8	63.5	16.2	65.6	53.4	75.5
Greece	100	66.5	15.0	68.5	57.0	77.7	66.5	66.3	14.8	68.5	57.2	77.4	33.5	66.8	15.3	68.7	56.9	78.5
Iceland	100	58.3	17.3	59.6	46.9	72.5	65.9	59.5	17.8	60.8	48.3	73.6	34.1	56.1	16.2	57.5	45.8	67.4
Netherlands	100	61.3	15.5	63.3	52.1	73.1	61.1	61.4	15.6	63.3	52.1	73.3	38.9	61.2	15.5	63.4	52.1	72.9
Norway	100	60.4	16.1	62.5	50.5	72.6	64.8	60.8	16.1	62.9	51.2	73.0	35.2	59.6	16.0	61.4	49.4	71.5
Romania	100	65.0	15.6	67.0	55.0	75.7	57.7	64.3	15.4	66.0	54.3	75.0	42.3	66.0	15.9	68.2	55.8	76.8
Serbia	100	61.8	15.1	63.8	52.3	72.5	62.5	61.6	15.4	64.0	51.8	72.4	37.5	62.0	14.7	63.5	53.1	72.6
Spain, Andalusia	100	62.1	15.3	63.6	52.6	73.9	62.0	62.0	15.0	63.5	52.6	73.4	38.0	62.4	15.7	63.8	52.5	74.7
Spain, Aragon	100	66.5	15.3	68.4	57.4	78.0	67.0	67.0	14.9	69.1	58.1	78.0	33.0	65.4	16.0	66.3	55.5	77.7
Spain, Basque country	100	62.5	15.3	64.8	52.5	74.1	64.2	63.0	15.2	65.2	53.5	74.3	35.8	61.8	15.5	63.7	51.5	73.8
Spain, Canary Islands	100	63.1	14.3	64.0	53.7	74.0	65.9	63.3	14.1	64.1	54.4	73.9	34.1	62.9	14.5	63.8	53.0	74.0
Spain, Cantabria *	100	64.9	13.9	66.3	55.6	75.8	68.4	65.2	13.8	66.9	56.1	75.9	31.6	64.2	14.1	65.5	54.8	75.7
Spain, Castile and León *	100	66.3	14.2	67.3	57.5	77.0	66.5	66.4	14.2	67.7	57.6	77.1	33.5	66.1	14.2	66.6	57.4	76.9
Spain, Castile-La Mancha *	100	64.9	14.1	65.6	55.8	75.8	62.7	65.0	13.6	65.7	56.0	75.4	37.3	64.7	14.8	65.3	55.0	76.6
Spain, Catalonia	100	63.6	15.5	65.3	53.9	75.3	64.2	63.7	15.4	65.4	54.0	75.3	35.8	63.5	15.8	65.1	53.8	75.5
Spain, Community of Madrid	100	63.0	16.1	64.6	53.5	75.2	63.4	63.4	15.8	65.0	54.3	75.3	36.4	62.3	16.4	63.8	51.9	74.9
Spain, Extremadura	100	64.4	14.1	65.5	55.6	74.8	63.6	64.8	13.8	66.1	56.4	75.0	36.4	63.7	14.7	63.7	54.6	74.2
Spain, Galicia	100	64.5	14.3	65.9	55.4	75.3	64.8	64.7	14.1	66.1	56.1	75.5	35.2	64.1	14.6	65.5	54.1	74.9
Spain, La Rioja	100	62.4	14.3	62.8	52.0	73.6	63.7	61.6	14.3	62.3	51.7	72.7	36.3	63.9	14.3	64.5	53.5	75.1
Spain, Murcia	100	63.2	15.2	64.6	54.0	74.5	64.4	63.2	15.1	64.6	54.0	74.6	35.6	63.0	15.4	64.6	54.2	74.4
Spain, Navarre *	100	63.6	14.3	65.4	53.9	74.5	65.0	64.4	13.8	66.2	54.8	74.7	35.0	62.0	15.2	63.2	51.9	73.9
Spain, Valencian region	100	64.3	15.2	66.2	54.6	75.9	63.7	64.2	15.4	66.1	54.5	75.9	36.3	64.6	15.0	66.3	55.0	75.9
Sweden	100	60.7	16.4	62.6	50.7	73.4	64.6	60.9	16.3	62.6	51.0	73.6	35.4	60.3	16.6	62.2	50.0	73.0
Switzerland	100	63.5	16.0	65.5	54.3	75.5	63.4	63.5	16.0	65.5	54.5	75.5	36.6	63.4	16.0	65.7	54.0	75.3
United Kingdom, England	100	58.4	16.2	59.9	48.4	70.5	61.6	58.6	16.2	60.1	48.6	70.6	38.4	58.1	16.2	59.6	48.0	70.1
United Kingdom, Northern Ireland	100	58.8	16.3	60.4	48.3	71.2	61.2	58.6	16.4	59.9	48.3	70.8	38.8	59.1	16.2	60.7	48.1	71.8
United Kingdom, Scotland	100	58.0	15.2	59.7	49.0	69.0	60.8	57.9	15.6	59.7	48.3	69.1	39.1	58.3	14.6	59.8	49.9	68.7
United Kingdom, Wales	100	58.8	16.5	60.0	48.2	71.6	62.7	59.1	16.7	60.2	48.7	72.0	37.3	58.4	16.3	59.7	47.8	71.0

Categories may not add up due to missing values or rounding
* Patients younger than 20 years of age are not reported

Table B.4.4

Prevalence per million population, adjusted

prevalent patients on December 31, adjusted for age and sex

	All	Male	Female
	Pmp	Pmp	Pmp
Austria	1020.9	1332.1	723.5
Belgium, Dutch-speaking *	1271.0	1563.2	991.6
Belgium, French-speaking *	1658.8	2083.6	1252.7
Bosnia and Herzegovina	843.5	1075.4	621.8
Denmark	1046.0	1306.9	796.5
Estonia	902.0	1141.7	672.9
France	1456.8	1855.7	1075.3
Greece	1393.5	1882.9	925.6
Iceland	1033.7	1352.9	728.6
Netherlands	1084.8	1320.2	859.7
Norway	1082.4	1388.6	789.6
Romania	1449.5	1768.4	1144.6
Serbia	900.2	1160.1	651.8
Spain, Andalusia	1401.7	1763.0	1056.2
Spain, Aragon	1461.3	1976.7	968.6
Spain, Basque country	1243.9	1625.9	878.8
Spain, Canary Islands	1647.9	2189.8	1129.9
Spain, Cantabria *	1166.1	1629.1	723.5
Spain, Castile and León *	1181.1	1566.0	813.0
Spain, Castile-La Mancha *	1266.0	1583.9	962.0
Spain, Catalonia	1612.9	2112.0	1135.7
Spain, Community of Madrid	1336.4	1775.6	916.5
Spain, Extremadura	1306.4	1666.1	962.5
Spain, Galicia	1329.0	1758.9	918.1
Spain, La Rioja	1183.8	1510.9	871.1
Spain, Murcia	1628.2	2115.7	1162.1
Spain, Navarre *	1402.8	1829.3	995.0
Spain, Valencian region	1484.4	1919.3	1068.7
Sweden	1058.0	1352.1	776.9
Switzerland	1057.9	1340.4	787.8
United Kingdom, England	1116.0	1393.0	851.2
United Kingdom, Northern Ireland	1205.1	1491.3	931.4
United Kingdom, Scotland	1053.9	1312.3	806.9
United Kingdom, Wales	1124.9	1430.1	833.2

* Patients younger than 20 years of age are not reported

Table B.4.5

Prevalence per million population and percentages by primary renal disease (1995 PRD codes), unadjusted

prevalent patients on December 31

	All		GN		PN		PKD		DM						HT		RVD		Misc		Unkn		Missing	
									Type 1		Type 2		Total											
	Pmp	%	Pmp	%	Pmp	%	Pmp	%	Pmp	%	Pmp	%	Pmp	%	Pmp	%	Pmp	%	Pmp	%	Pmp	%	Pmp	%
Austria	1007.0	100	217.1	21.6	65.9	6.5	84.7	8.4	49.3	4.9	124.7	12.4	174.1	17.3	111.1	11.0	27.7	2.7	209.0	20.8	116.1	11.5	1.3	0.1
Belgium, Dutch-speaking *	1273.4	100	228.5	17.9	76.9	6.0	140.2	11.0	48.1	3.8	163.5	12.8	211.6	16.6	108.9	8.6	28.5	2.2	345.3	27.1	133.4	10.5	0	0
Belgium, French-speaking *	1458.2	100	230.1	15.8	91.4	6.3	127.1	8.7	37.2	2.6	212.0	14.5	249.3	17.1	211.2	14.5	16.3	1.1	357.4	24.5	175.4	12.0	0	0
Bosnia and Herzegovina	702.9	100	130.6	18.6	84.1	12.0	40.2	5.7	39.1	5.6	94.6	13.5	133.7	19.0	86.9	12.4	10.2	1.5	106.5	15.1	110.7	15.8	0	0
Denmark #	1018.3	100	212.4	20.9	66.3	6.5	101.4	10.0	66.3	6.5	101.6	10.0	167.8	16.5	97.0	9.5	6.1	0.6	184.3	18.1	183.1	18.0	0	0
Estonia	838.9	100	224.3	26.7	86.0	10.3	86.8	10.3					143.7	17.1	139.0	16.6	38.4	4.6	119.8	14.3	0.8	0.1	0	0
France	1408.7	100	277.7	19.7	103.4	7.3	145.5	10.3					231.5	16.4	233.1	16.5	12.4	0.9	240.1	17.0	164.9	11.7	0	0
Greece	1497.3	100	242.9	16.2	90.6	6.1	123.7	8.3	24.8	1.7	239.1	16.0	263.9	17.6	139.3	9.3	20.9	1.4	170.2	11.4	445.7	29.8	0.2	0
Iceland	881.6	100	186.7	21.2	54.5	6.2	101.1	11.5	54.5	6.2	51.9	5.9	106.3	12.1	132.2	15.0	62.2	7.1	202.2	22.9	36.3	4.1	0	0
Netherlands	1068.8	100	176.9	16.6	61.3	5.7	93.4	8.7	39.7	3.7	102.0	9.5	141.7	13.3	131.2	12.3	40.8	3.8	185.8	17.4	220.5	20.6	17.2	1.6
Norway	1015.5	100	281.2	27.7	64.0	6.3	117.4	11.6					131.4	12.9	175.6	17.3	5.1	0.5	206.7	20.4	29.7	2.9	4.5	0.4
Romania	1334.8	100	212.3	15.9	87.9	6.6	48.8	3.7					122.6	9.2	73.1	5.5	7.7	0.6	209.3	15.7	573.1	42.9	0	0
Serbia	920.1	100	160.8	17.5	81.2	8.8	59.5	6.5					159.6	17.3	237.4	25.8	8.8	1.0	145.2	15.8	63.8	6.9	3.9	0.4
Spain, Andalusia	1356.4	100	270.7	20.0	113.8	8.4	132.4	9.8					227.8	16.8	108.3	8.0	6.2	0.5	223.5	16.5	273.7	20.2	0	0
Spain, Aragon	1549.3	100	311.2	20.1	82.4	5.3	137.4	8.9	62.4	4.0	207.2	13.4	269.6	17.4	177.5	11.5	3.7	0.2	203.5	13.1	363.9	23.5	0	0
Spain, Basque country	1333.9	100	219.6	16.5	104.0	7.8	163.4	12.2	37.4	2.8	150.3	11.3	187.7	14.1	136.8	10.3	3.6	0.3	226.4	17.0	291.2	21.8	1.4	0.1
Spain, Canary Islands	1608.8	100	244.4	15.2	82.7	5.1	102.9	6.4	45.8	2.8	357.6	22.2	403.4	25.1	69.2	4.3	4.0	0.3	352.2	21.9	343.2	21.3	6.7	0.4
Spain, Cantabria *	1273.7	100	198.4	15.6	88.2	6.9	142.5	11.2	42.4	3.3	152.6	12.0	195.0	15.3	96.7	7.6	1.7	0.1	164.5	12.9	291.7	22.9	95.0	7.5
Spain, Castile and León * ‡	1384.4	100	263.0	19.0	93.6	6.8	127.5	9.2	63.8	4.6	172.0	12.4	235.8	17.0	150.6	10.9	1.7	0.1	235.3	17.0	276.9	20.0	0	0
Spain, Castile-La Mancha *	1254.9	100	201.0	16.0	121.3	9.7	152.3	12.1					212.0	16.9	61.1	4.9	0	0	181.0	14.4	323.7	25.8	2.4	0.2
Spain, Catalonia	1552.4	100	299.5	19.3	125.7	8.1	166.8	10.7	40.2	2.6	195.6	12.6	239.8	15.4	114.5	7.4	28.6	1.8	168.9	10.9	324.5	20.9	84.0	5.4
Spain, Community of Madrid	1251.8	100	260.5	20.8	78.1	6.2	135.6	10.8	43.1	3.4	163.4	13.1	206.5	16.5	119.2	9.5	0	0	224.0	17.9	157.6	12.6	70.3	5.6
Spain, Extremadura	1382.9	100	209.6	15.2	91.1	6.6	128.0	9.3					226.7	16.4	85.4	6.2	0	0	202.0	14.6	436.3	31.6	3.8	0.3
Spain, Galicia	1512.2	100	276.8	18.3	87.7	5.8	190.9	12.6	62.2	4.1	186.5	12.3	248.6	16.4	119.9	7.9	5.6	0.4	284.2	18.8	298.6	19.7	0	0
Spain, La Rioja	1234.4	100	327.9	26.6	49.5	4.0	136.1	11.0	52.6	4.3	102.1	8.3	154.7	12.5	102.1	8.3	3.1	0.3	371.2	30.1	89.7	7.3	0	0
Spain, Murcia	1442.9	100	374.4	25.9	108.3	7.5	128.9	8.9	41.9	2.9	191.4	13.3	233.3	16.2	96.7	6.7	0.6	0	252.0	17.5	248.8	17.2	0	0
Spain, Navarre *	1403.9	100	253.2	18.0	42.9	3.1	188.1	13.4	78.5	5.6	152.5	10.9	231.0	16.5	159.9	11.4	1.5	0.1	299.2	21.3	228.1	16.2	0	0
Spain, Valencian region	1486.3	100	203.6	13.7	113.9	7.7	141.9	9.5					216.1	14.5	179.2	12.1	6.1	0.4	255.7	17.2	369.8	24.9	0	0
Sweden ‡	1010.7	100	254.5	25.2	50.3	5.0	109.8	10.9					170.4	16.9	118.3	11.7	3.6	0.4	209.9	20.8	93.9	9.3	0	0
Switzerland	1025.4	100	212.6	20.7	38.6	3.8	32.4	3.2	20.1	2.0	112.4	11.0	132.5	12.9	141.6	13.8	14.9	1.5	320.6	31.3	68.3	6.7	63.8	6.2
United Kingdom, England #	1037.6	100	190.5	18.4	94.4	9.1	96.4	9.3					190.4	18.3	71.1	6.9	7.7	0.7	186.1	17.9	157.6	15.2	43.4	4.2
United Kingdom, Northern Ireland #	1105.5	100	213.5	19.3	160.4	14.5	137.0	12.4					161.4	14.6	27.6	2.5	5.7	0.5	231.7	21.0	147.9	13.4	20.3	1.8
United Kingdom, Scotland	1042.8	100	219.7	21.1	110.2	10.6	118.0	11.3					175.0	16.8	39.3	3.8	7.5	0.7	206.9	19.8	104.4	10.0	61.7	5.9
United Kingdom, Wales #	1105.4	100	271.8	24.6	107.8	9.7	111.2	10.1					205.7	18.6	41.4	3.7	6.6	0.6	225.0	20.4	133.0	12.0	2.8	0.3

Abbreviations used: GN: glomerulonephritis/sclerosis; PN: pyelonephritis; PKD: polycystic kidneys, adult type; DM: diabetes mellitus; HT: hypertension; RVD: renal vascular disease; Misc: miscellaneous; Unkn: unknown
Categories may not add up due to rounding or a limited number of cases (<10%) with diabetes mellitus type unknown; When cells are left empty, (complete) data are unavailable
* Patients younger than 20 years of age are not reported
\# Values based on 1 to 5 patients are omitted
‡ Mapping the 2018 PRD codes to the old PRD codes results in a different distribution of PRD groups

FIND

Table B.4.6

Prevalence per million population by primary renal disease (1995 PRD codes), adjusted

prevalent patients on December 31, adjusted for age and sex

	All	GN	PN	PKD	DM			HT	RVD	Misc	Unkn	Missing
					Type 1	Type 2	Total					
	Pmp	Pmp	Pmp	Pmp	Pmp	Pmp	Pmp	Pmp	Pmp	Pmp	Pmp	Pmp
Austria	1020.9	217.3	66.2	86.1	49.4	129.1	178.5	113.6	28.7	211.0	118.2	1.4
Belgium, Dutch-speaking *	1271.0	229.4	77.5	142.1	49.1	160.6	209.6	107.4	27.5	343.2	134.3	0
Belgium, French-speaking *	1658.8	256.6	103.0	143.8	40.7	246.4	287.1	247.7	19.2	401.4	200.1	0
Bosnia and Herzegovina	843.5	140.2	103.5	46.7	44.1	118.8	162.8	105.7	12.2	138.5	134.0	0
Denmark #	1046.0	221.2	67.3	105.7	70.4	101.9	172.3	98.2	6.1	188.8	186.4	0
Estonia	902.0	239.2	91.5	89.9			150.7	160.8	42.6	126.3	0.9	0
France	1456.8	291.6	107.6	152.1			237.3	236.9	12.7	248.7	169.9	0
Greece	1393.5	232.2	85.5	117.4	23.8	218.3	242.1	127.3	19.0	161.2	408.7	0
Iceland	1033.7	211.0	66.7	123.2	61.4	61.2	122.6	169.0	85.1	219.1	36.9	0
Netherlands	1084.8	179.4	62.5	95.0	41.2	102.3	143.5	133.5	41.1	188.1	224.1	17.6
Norway	1082.4	296.7	67.5	129.8			141.0	189.5	5.6	216.5	31.2	4.6
Romania	1449.5	221.0	98.3	50.5			133.3	82.1	8.9	230.1	625.3	0
Serbia	900.2	156.4	80.4	57.3			153.4	231.7	8.6	143.3	64.8	4.2
Spain, Andalusia	1401.7	270.9	115.7	135.5			239.2	116.8	6.9	229.9	286.6	0
Spain, Aragon	1461.3	296.7	79.0	131.1	59.8	193.7	253.5	162.2	3.3	193.4	342.1	0
Spain, Basque country	1243.9	207.5	97.8	150.6	34.6	137.6	172.3	124.1	3.2	214.8	272.4	1.3
Spain, Canary Islands	1647.9	240.5	80.1	96.4	40.6	382.7	423.3	75.6	4.3	366.4	355.1	6.2
Spain, Cantabria *	1166.1	181.6	82.1	129.1	39.3	139.5	178.8	85.8	1.8	152.1	264.9	89.7
Spain, Castile and León * ‡	1181.1	231.5	81.5	112.0	58.5	138.0	196.6	120.4	1.4	205.1	232.6	0
Spain, Castile-La Mancha *	1266.0	200.6	119.3	156.7			215.8	60.4	0	183.1	327.5	2.5
Spain, Catalonia	1612.9	307.6	127.5	173.6	39.4	210.3	253.9	120.0	30.5	171.5	339.7	88.6
Spain, Community of Madrid	1336.4	274.9	80.5	146.4	42.6	184.4	227.0	130.8	0	232.4	170.7	73.7
Spain, Extremadura	1306.4	199.3	87.4	120.0			209.8	82.0	0	192.0	412.4	3.6
Spain, Galicia	1329.0	248.8	78.6	168.5	57.6	157.1	214.7	100.5	4.6	258.3	254.9	0
Spain, La Rioja	1183.8	310.8	45.6	131.3	48.7	98.0	146.7	97.3	2.5	362.9	86.8	0
Spain, Murcia	1628.2	415.3	117.7	138.8	40.1	230.0	270.1	116.3	0.8	281.7	287.5	0
Spain, Navarre *	1402.8	252.6	44.0	189.4	75.9	154.7	230.7	156.3	1.4	301.0	227.4	0
Spain, Valencian region	1484.4	201.0	112.7	139.7			216.5	182.6	6.2	255.0	370.6	0
Sweden ‡	1058.0	267.6	51.7	119.2			181.1	119.7	3.6	217.8	97.5	0
Switzerland	1057.9	216.9	39.8	34.3	20.6	116.8	137.5	146.5	15.5	331.8	70.8	64.8
United Kingdom, England #	1116.0	204.1	100.1	106.6			208.0	77.2	8.3	196.0	169.6	46.2
United Kingdom, Northern Ireland #	1205.1	231.8	171.0	151.0			177.0	31.3	6.5	250.5	164.0	22.0
United Kingdom, Scotland	1053.9	222.8	111.7	119.2			177.2	39.9	7.6	208.8	106.6	60.2
United Kingdom, Wales #	1124.9	278.4	112.0	114.1			209.9	41.6	6.6	224.6	134.7	2.9

Abbreviations used: GN: glomerulonephritis/sclerosis; PN: pyelonephritis; PKD: polycystic kidneys, adult type; DM: diabetes mellitus; HT: hypertension; RVD: renal vascular disease; Misc: miscellaneous; Unkn: unknown
Categories may not add up due to rounding or a limited number of cases (<10%) with diabetes mellitus type unknown; When cells are left empty, (complete) data are unavailable
* Patients younger than 20 years of age are not reported
# Values based on 1 to 5 patients are omitted
‡ Mapping the 2018 PRD codes to the old PRD codes results in a different distribution of PRD groups

Table B.4.7

Prevalent counts by treatment modality

prevalent patients on December 31

	All	Haemodialysis						Peritoneal dialysis				Kidney transplant				Unkn	Missing
		HD hospital/ centre	HD home	HD type Unkn	HF	HDF	Total HD/ HF/HDF	APD	CAPD	PD type Unkn	Total PD	Living donor	Deceased donor	Tx type Unkn	Total Tx		
	N	N	N	N	N	N	N	N	N	N	N	N	N	N	N	N	N
Austria §	8985	3093	1	9	7	1112	4222	175	122	4	301	860	3595	6	4461	1	0
Belgium, Dutch-speaking *	8676	4298	79	150			4527			337	337	359	3387	66	3812	0	0
Belgium, French-speaking *	7242	2128	165	0	46	1481	3820	176	140	0	316	316	2607	171	3094	10	2
Bosnia and Herzegovina	2482	1213	1	0	0	761	1975	1	62	3	66	278	155	8	441	0	0
Denmark #	6056	2009	170	0			2179	249	240	0	489	1299	2079	0	3378	10	0
Estonia	1092	392	0	0	0	0	392	33	11	0	44	35	621	0	656	0	0
France	96317	30161	766	81	79	18782	49869	1096	1839	13	2948	6398	35470	1153	43021	479	0
Greece	15583	8028	2	0	0	3993	12023	353	204	0	557	1277	1726	0	3003	0	0
Iceland	340	76	0	0			76	0	20	0	20	123	121	0	244	0	0
Netherlands &	18342	4072	215	0		837	5124	641	293	0	934	6723	5550	9	12282	2	0
Norway	5605	1360	42	0			1402			453	453	1347	2343	8	3698	52	0
Romania ||	25442	21091	0	0	23	1157	22271	53	944	0	997	588	1013	566	2167	7	0
Serbia	5881	3622	34	22	23	749	4450	56	455	4	515	476	412	6	894	16	6
Spain, Andalusia	11676	4584	56	0			4640			407	407	663	5955	11	6629	0	0
Spain, Aragon	2086	507	6	0		155	668	20	55	0	75	129	1212	0	1341	2	0
Spain, Basque country	2964	830	25	0			855	31	125	0	156	253	1700	0	1953	0	0
Spain, Canary Islands	3581	1610	5	0	0	0	1615	0	0	288	288	75	1602	0	1677	1	0
Spain, Cantabria *	751	238	9	0			247	5	23	0	28	23	453	0	476	0	0
Spain, Castile and León *	3300	1249	21	0			1270	85	85	0	170	121	1674	0	1795	65	0
Spain, Castile-La Mancha *	2628	996	2	0			998	24	132	0	156	47	1378	49	1474	0	0
Spain, Catalonia	12267	857	75	0		3215	4147	151	264	0	415	1686	6019	0	7705	0	0
Spain, Community of Madrid	8602	3260	57	0		0	3317	87	381	0	468	475	4322	4	4801	16	0
Spain, Extremadura	1458	732	16	25			773	23	23	1	47	28	412	196	636	2	0
Spain, Galicia	4087	1718	29	0	0	0	1747	128	148	0	276	319	1668	75	2062	2	0
Spain, La Rioja	399	132	1	0	0	0	133	17	6	0	23	16	227	0	243	0	0
Spain, Murcia	2239	440	0	0	0	606	1046	19	84	0	103	81	1009	0	1090	0	0
Spain, Navarre *	948	298	24	0			322	45	2	0	47	39	540	0	579	0	0
Spain, Valencian region	7753	3447	115	0			3562	80	364	0	444	175	3120	445	3740	7	0
Sweden	10649	3068	102	0			3170			931	931	2559	3986	0	6545	3	0
Switzerland	8659	3404	53	0			3457	152	131	0	283	1886	3023	1	4910	9	0
United Kingdom, England #	55070	15844	1106	8	15	4712	21685	1725	1221	37	2983	9722	19820	853	30395	7	0
United Kingdom, Northern Ireland #	2123	536	14	0	0	10	560	41	10		52	698	754	59	1511	0	0
United Kingdom, Scotland	5725	1977	50	0			2027	124	63	10	197	1196	2220	78	3494	7	0
United Kingdom, Wales #	3498	1146	127	0	0	147	1420	95	58	0	153	638	1267	20	1925	0	0

Abbreviations used: HD: haemodialysis; Unkn: unknown; HF: haemofiltration; HDF: haemodiafiltration; APD: automated peritoneal dialysis; CAPD: continuous ambulatory peritoneal dialysis; PD: peritoneal dialysis; Tx: transplant
When cells are left empty, (complete) data are unavailable
§ The prevalence is underestimated by approximately 2% due to an estimated 2% underestimation of the prevalence of haemodialysis
* Patients younger than 20 years of age are not reported
# Values based on 1 to 5 patients are omitted
& The prevalence is underestimated by approximately 4%
|| The prevalence of patients living with a functioning graft is underestimated by approximately 30%

Table B.4.8

Prevalence per million population by treatment modality, unadjusted

prevalent patients on December 31

	All	Haemodialysis						Peritoneal dialysis				Kidney transplant				Unkn	Missing
		HD hospital/ centre	HD home	HD type Unkn	HF	HDF	Total HD/ HF/HDF	APD	CAPD	PD type Unkn	Total PD	Living donor	Deceased donor	Tx type Unkn	Total Tx		
	Pmp	Pmp	Pmp	Pmp	Pmp	Pmp	Pmp	Pmp	Pmp	Pmp	Pmp	Pmp	Pmp	Pmp	Pmp	Pmp	Pmp
Austria	1007.0	346.6	0.1	1.0	0.8	124.6	473.2	19.6	13.7	0.4	33.7	96.4	402.9	0.7	500.0	0.1	0
Belgium, Dutch-speaking *	1273.4	630.8	11.6	22.0			664.4			49.5	49.5	52.7	497.1	9.7	559.5	0	0
Belgium, French-speaking *	1458.2	428.5	33.2	0	9.3	298.2	769.1	35.4	28.2	0	63.6	63.6	524.9	34.4	623.0	2.0	0.4
Bosnia and Herzegovina	702.9	343.5	0.3	0	0	215.5	559.3	0.3	17.6	0.8	18.7	78.7	43.9	2.3	124.9	0	0
Denmark #	1018.3	337.8	28.6	0			366.4	41.9	40.4	0	82.2	218.4	349.6	0	568.0	1.7	0
Estonia	838.9	301.1	0	0	0	0	301.1	25.4	8.5	0	33.8	26.9	477.0	0	503.9	0	0
France	1408.7	441.1	11.2	1.2	1.2	274.7	729.4	16.0	26.9	0.2	43.1	93.6	518.8	16.9	629.2	7.0	0
Greece	1497.3	771.4	0.2	0	0	383.7	1155.2	33.9	19.6	0	53.5	122.7	165.8	0	288.5	0	0
Iceland	881.6	197.1	0	0			197.1	0	51.9	0	51.9	318.9	313.7	0	632.7	0	0
Netherlands	1068.8	237.3	12.5	0		48.8	298.6	37.3	17.1	0	54.4	391.7	323.4	0.5	715.6	0.1	0
Norway	1015.5	246.4	7.6	0			254.0			82.1	82.1	244.0	424.5	1.4	670.0	9.4	0
Romania | |	1334.8	1106.5	0	0	1.2	60.7	1168.4	2.8	49.5	0	52.3	30.8	53.1	29.7	113.7	0.4	0
Serbia	920.1	566.7	5.3	3.4	3.6	117.2	696.3	8.8	71.2	0.6	80.6	74.5	64.5	0.9	139.9	2.5	0.9
Spain, Andalusia	1356.4	532.5	6.5	0			539.0			47.3	47.3	77.0	691.8	1.3	770.1	0	0
Spain, Aragon	1549.3	376.5	4.5	0		115.1	496.1	14.9	40.8	0	55.7	95.8	900.2	0	996.0	1.5	0
Spain, Basque country	1333.9	373.5	11.3	0			384.8	14.0	56.3	0	70.2	113.9	765.1	0	878.9	0	0
Spain, Canary Islands	1608.8	723.3	2.2	0	0	0	725.6	0	0	129.4	129.4	33.7	719.7	0	753.4	0.4	0
Spain, Cantabria *	1273.7	403.7	15.3	0			418.9	8.5	39.0	0	47.5	39.0	768.3	0	807.3	0	0
Spain, Castile and León *	1384.4	524.0	8.8	0			532.8	35.7	35.7	0	71.3	50.8	702.3	0	753.0	27.3	0
Spain, Castile-La Mancha *	1254.9	475.6	1.0	0			476.5	11.5	63.0	0	74.5	22.4	658.0	23.4	703.8	0	0
Spain, Catalonia	1552.4	108.5	9.5	0		406.9	524.8	19.1	33.4	0	52.5	213.4	761.7	0	975.1	0	0
Spain, Community of Madrid	1251.8	474.4	8.3	0		0	482.7	12.7	55.4	0	68.1	69.1	628.9	0.6	698.6	2.3	0
Spain, Extremadura	1382.9	694.3	15.2	23.7			733.2	21.8	21.8	0.9	44.6	26.6	390.8	185.9	603.2	1.9	0
Spain, Galicia	1512.2	635.7	10.7	0	0	0	646.4	47.4	54.8	0	102.1	118.0	617.2	27.8	763.0	0.7	0
Spain, La Rioja	1234.4	408.4	3.1	0	0	0	411.5	52.6	18.6	0	71.2	49.5	702.3	0	751.8	0	0
Spain, Murcia	1442.9	283.6	0	0	0	390.5	674.1	12.2	54.1	0	66.4	52.2	650.3	0	702.5	0	0
Spain, Navarre *	1403.9	441.3	35.5	0			476.9	66.6	3.0	0	69.6	57.8	799.7	0	857.5	0	0
Spain, Valencian region	1486.3	660.8	22.0	0			682.9	15.3	69.8	0	85.1	33.5	598.1	85.3	717.0	1.3	0
Sweden	1010.7	291.2	9.7	0			300.9			88.4	88.4	242.9	378.3	0	621.2	0.3	0
Switzerland	1025.4	403.1	6.3	0			409.4	18.0	15.5	0	33.5	223.3	358.0	0.1	581.5	1.1	0
United Kingdom, England #	1037.6	298.5	20.8	0.2	0.3	88.8	408.6	32.5	23.0	0.7	56.2	183.2	373.4	16.1	572.7	0.1	0
United Kingdom, Northern Ireland #	1105.5	279.1	7.3	0	0	5.2	291.6	21.3	5.2		27.1	363.5	392.6	30.7	786.8	0	0
United Kingdom, Scotland	1042.8	360.1	9.1	0			369.2	22.6	11.5	1.8	35.9	217.8	404.4	14.2	636.4	1.3	0
United Kingdom, Wales #	1105.4	362.2	40.1	0	0	46.5	448.7	30.0	18.3	0	48.4	201.6	400.4	6.3	608.3	0	0

Abbreviations used: HD: haemodialysis; Unkn: unknown; HF: haemofiltration; HDF: haemodiafiltration; APD: automated peritoneal dialysis; CAPD: continuous ambulatory peritoneal dialysis; PD: peritoneal dialysis; Tx: transplant
Categories may not add up due to rounding; When cells are left empty, (complete) data are unavailable
* Patients younger than 20 years of age are not reported
# Values based on 1 to 5 patients are omitted
| | The prevalence of patients living with a functioning graft is underestimated by approximately 30%

Table B.4.9

Prevalence per million population by treatment modality, adjusted

prevalent patients on December 31, adjusted for age and sex

	All	Haemodialysis						Peritoneal dialysis				Kidney transplant				Unkn	Missing
		HD hospital/ centre	HD home	HD type Unkn	HF	HDF	Total HD/ HF/HDF	APD	CAPD	PD type Unkn	Total PD	Living donor	Deceased donor	Tx type Unkn	Total Tx		
	Pmp	Pmp	Pmp	Pmp	Pmp	Pmp	Pmp	Pmp	Pmp	Pmp	Pmp	Pmp	Pmp	Pmp	Pmp	Pmp	Pmp
Austria	1020.9	356.2	0.1	1.0	0.8	126.1	484.2	19.6	13.7	0.5	33.7	95.3	407.0	0.7	502.9	0.1	0
Belgium, Dutch-speaking *	1271.0	620.5	11.8	21.7			654.0			49.4	49.4	54.1	503.6	9.9	567.5	0	0
Belgium, French-speaking *	1658.8	498.7	36.6	0	10.8	345.7	891.8	40.2	32.0	0	72.2	67.1	585.4	39.6	692.1	2.2	0.5
Bosnia and Herzegovina	843.5	422.0	0.3	0	0	272.4	694.6	0.4	21.4	1.0	22.8	77.7	46.0	2.3	126.0	0	0
Denmark #	1046.0	341.7	30.0	0			371.7	41.8	40.5	0	82.3	226.9	363.3	0	590.2	1.8	0
Estonia	902.0	333.9	0	0	0	0	333.9	27.6	9.0	0	36.5	27.7	504.0	0	531.6	0	0
France	1456.8	450.7	11.9	1.2	1.2	279.6	744.6	16.4	27.3	0.2	43.9	98.9	544.7	17.5	661.1	7.2	0
Greece	1393.5	698.6	0.2	0	0	364.3	1063.1	32.3	18.4	0	50.7	120.5	159.3	0	279.7	0	0
Iceland	1033.7	249.2	0	0			249.2	0	64.0	0	64.0	365.4	355.1	0	720.5	0	0
Netherlands	1084.8	241.9	12.8	0		49.8	304.5	37.7	17.3	0	55.0	397.1	327.6	0.5	725.2	0.1	0
Norway	1082.4	266.2	8.1	0			274.3			88.5	88.5	254.5	453.9	1.5	709.9	9.6	0
Romania ||	1449.5	1212.3	0	0	1.4	62.1	1275.8	3.1	57.4	0	60.5	30.4	52.5	30.0	112.9	0.3	0
Serbia	900.2	553.4	5.3	3.3	3.4	114.6	680.0	8.7	70.1	0.7	79.4	73.9	62.5	0.9	137.4	2.4	1.0
Spain, Andalusia	1401.7	573.8	6.6	0			580.4			50.1	50.1	75.0	694.9	1.3	771.2	0	0
Spain, Aragon	1461.3	350.3	3.9	0		108.6	462.8	14.1	36.7	0	50.8	93.6	852.7	0	946.2	1.4	0
Spain, Basque country	1243.9	344.9	11.3	0			356.2	13.1	51.2	0	64.3	110.2	713.2	0	823.4	0	0
Spain, Canary Islands	1647.9	775.8	2.1	0	0	0	777.9	0	0	134.4	134.4	30.6	704.6	0	735.2	0.5	0
Spain, Cantabria *	1166.1	366.6	15.2	0			381.8	8.3	36.2	0	44.5	37.5	702.3	0	739.8	0	0
Spain, Castile and León *	1181.1	425.9	7.7	0			433.5	31.2	31.0	0	62.2	48.5	619.2	0	667.8	17.5	0
Spain, Castile-La Mancha *	1266.0	481.4	0.9	0			482.3	11.1	64.1	0	75.2	22.2	662.6	23.7	708.5	0	0
Spain, Catalonia	1612.9	115.4	9.6	0		427.0	552.1	20.0	35.3	0	55.4	215.6	789.8	0	1005.4	0	0
Spain, Community of Madrid	1336.4	515.2	8.6	0		0	523.8	13.1	59.8	0	72.9	68.5	668.1	0.6	737.2	2.5	0
Spain, Extremadura	1306.4	651.7	14.6	22.6			689.0	21.3	21.4	0.8	43.5	26.3	370.6	175.2	572.0	1.9	0
Spain, Galicia	1329.0	533.7	9.8	0	0	0	543.5	42.8	46.7	0	89.5	110.0	555.3	30.0	695.4	0.7	0
Spain, La Rioja	1183.8	392.2	2.8	0	0	0	395.0	51.1	18.2	0	69.3	49.1	670.4	0	719.5	0	0
Spain, Murcia	1628.2	333.2	0	0	0	461.3	794.5	13.1	61.8	0	75.0	53.4	705.4	0	758.7	0	0
Spain, Navarre *	1402.8	440.2	36.7	0			476.8	66.2	2.7	0	68.8	57.0	800.1	0	857.1	0	0
Spain, Valencian region	1484.4	670.9	21.6	0			692.5	15.0	70.2	0	85.2	32.9	588.0	84.5	705.4	1.3	0
Sweden	1058.0	298.5	10.3	0			308.7			88.8	88.8	256.5	403.8	0	660.2	0.3	0
Switzerland	1057.9	419.0	6.4	0			425.4	18.2	16.2	0	34.3	226.8	370.1	0.1	597.0	1.1	0
United Kingdom, England #	1116.0	323.0	22.2	0.2	0.3	96.3	442.0	34.5	24.7	0.7	59.9	194.0	403.1	17.0	614.0	0.1	0
United Kingdom, Northern Ireland #	1205.1	314.0	7.8	0	0	5.8	327.6	23.9	6.2		30.7	386.5	427.4	32.9	846.8	0	0
United Kingdom, Scotland	1053.9	363.0	9.2	0			372.2	22.9	11.3	1.6	35.8	221.9	408.6	14.2	644.7	1.3	0
United Kingdom, Wales #	1124.9	360.5	40.7	0	0	45.9	447.1	30.2	18.2	0	48.5	209.3	413.0	7.1	629.3	0	0

Abbreviations used: HD: haemodialysis; Unkn: unknown; HF: haemofiltration; HDF: haemodiafiltration; APD: automated peritoneal dialysis; CAPD: continuous ambulatory peritoneal dialysis; PD: peritoneal dialysis; Tx: transplant
Categories may not add up due to rounding; When cells are left empty, (complete) data are unavailable
* Patients younger than 20 years of age are not reported
# Values based on 1 to 5 patients are omitted
|| The prevalence of patients living with a functioning graft is underestimated by approximately 30%

Table B.4.10

Treatment modality distribution, unadjusted

prevalent patients on December 31

	All	Haemodialysis						Peritoneal dialysis				Kidney transplant				Unkn	Missing
		HD hospital/ centre	HD home	HD type Unkn	HF	HDF	Total HD/ HF/HDF	APD	CAPD	PD type Unkn	Total PD	Living donor	Deceased donor	Tx type Unkn	Total Tx		
	%	%	%	%	%	%	%	%	%	%	%	%	%	%	%	%	%
Austria	100	34.4	0	0.1	0.1	12.4	47.0	1.9	1.4	0	3.4	9.6	40.0	0.1	49.6	0	0
Belgium, Dutch-speaking *	100	49.5	0.9	1.7			52.2			3.9	3.9	4.1	39.0	0.8	43.9	0	0
Belgium, French-speaking *	100	29.4	2.3	0	0.6	20.5	52.7	2.4	1.9	0	4.4	4.4	36.0	2.4	42.7	0.1	0
Bosnia and Herzegovina	100	48.9	0	0	0	30.7	79.6	0	2.5	0.1	2.7	11.2	6.2	0.3	17.8	0	0
Denmark #	100	33.2	2.8	0			36.0	4.1	4.0	0	8.1	21.4	34.3	0	55.8	0.2	0
Estonia	100	35.9	0	0	0	0	35.9	3.0	1.0	0	4.0	3.2	56.9	0	60.1	0	0
France	100	31.3	0.8	0.1	0.1	19.5	51.8	1.1	1.9	0	3.1	6.6	36.8	1.2	44.7	0.5	0
Greece	100	51.5	0	0	0	25.6	77.2	2.3	1.3	0	3.6	8.2	11.1	0	19.3	0	0
Iceland	100	22.4	0	0			22.4	0	5.9	0	5.9	36.2	35.6	0	71.8	0	0
Netherlands	100	22.2	1.2	0		4.6	27.9	3.5	1.6	0	5.1	36.7	30.3	0	67.0	0	0
Norway	100	24.3	0.7	0			25.0			8.1	8.1	24.0	41.8	0.1	66.0	0.9	0
Romania | |	100	82.9	0	0	0.1	4.5	87.5	0.2	3.7	0	3.9	2.3	4.0	2.2	8.5	0	0
Serbia	100	61.6	0.6	0.4	0.4	12.7	75.7	1.0	7.7	0.1	8.8	8.1	7.0	0.1	15.2	0.3	0.1
Spain, Andalusia	100	39.3	0.5	0			39.7			3.5	3.5	5.7	51.0	0.1	56.8	0	0
Spain, Aragon	100	24.3	0.3	0		7.4	32.0	1.0	2.6	0	3.6	6.2	58.1	0	64.3	0.1	0
Spain, Basque country	100	28.0	0.8	0			28.8	1.0	4.2	0	5.3	8.5	57.4	0	65.9	0	0
Spain, Canary Islands	100	45.0	0.1	0	0	0	45.1	0	0	8.0	8.0	2.1	44.7	0	46.8	0	0
Spain, Cantabria *	100	31.7	1.2	0			32.9	0.7	3.1	0	3.7	3.1	60.3	0	63.4	0	0
Spain, Castile and León *	100	37.8	0.6	0			38.5	2.6	2.6	0	5.2	3.7	50.7	0	54.4	2.0	0
Spain, Castile-La Mancha *	100	37.9	0.1	0			38.0	0.9	5.0	0	5.9	1.8	52.4	1.9	56.1	0	0
Spain, Catalonia	100	7.0	0.6	0		26.2	33.8	1.2	2.2	0	3.4	13.7	49.1	0	62.8	0	0
Spain, Community of Madrid	100	37.9	0.7	0		0	38.6	1.0	4.4	0	5.4	5.5	50.2	0	55.8	0.2	0
Spain, Extremadura	100	50.2	1.1	1.7			53.0	1.6	1.6	0.1	3.2	1.9	28.3	13.4	43.6	0.1	0
Spain, Galicia	100	42.0	0.7	0	0	0	42.7	3.1	3.6	0	6.8	7.8	40.8	1.8	50.5	0	0
Spain, La Rioja	100	33.1	0.3	0	0	0	33.3	4.3	1.5	0	5.8	4.0	56.9	0	60.9	0	0
Spain, Murcia	100	19.7	0	0	0	27.1	46.7	0.8	3.8	0	4.6	3.6	45.1	0	48.7	0	0
Spain, Navarre *	100	31.4	2.5	0			34.0	4.7	0.2	0	5.0	4.1	57.0	0	61.1	0	0
Spain, Valencian region	100	44.5	1.5	0			45.9	1.0	4.7	0	5.7	2.3	40.2	5.7	48.2	0.1	0
Sweden	100	28.8	1.0	0			29.8			8.7	8.7	24.0	37.4	0	61.5	0	0
Switzerland	100	39.3	0.6	0			39.9	1.8	1.5	0	3.3	21.8	34.9	0	56.7	0.1	0
United Kingdom, England #	100	28.8	2.0	0	0	8.6	39.4	3.1	2.2	0.1	5.4	17.7	36.0	1.5	55.2	0	0
United Kingdom, Northern Ireland #	100	25.2	0.7	0	0	0.5	26.4	1.9	0.5		2.4	32.9	35.5	2.8	71.2	0	0
United Kingdom, Scotland	100	34.5	0.9	0			35.4	2.2	1.1	0.2	3.4	20.9	38.8	1.4	61.0	0.1	0
United Kingdom, Wales #	100	32.8	3.6	0	0	4.2	40.6	2.7	1.7	0	4.4	18.2	36.2	0.6	55.0	0	0

Abbreviations used: HD: haemodialysis; Unkn: unknown; HF: haemofiltration; HDF: haemodiafiltration; APD: automated peritoneal dialysis; CAPD: continuous ambulatory peritoneal dialysis; PD: peritoneal dialysis; Tx: transplant
Categories may not add up due to rounding; When cells are left empty, (complete) data are unavailable
* Patients younger than 20 years of age are not reported
# Values based on 1 to 5 patients are omitted
| | The prevalence of patients living with a functioning graft is underestimated by approximately 30%

Table B.4.11

Treatment modality distribution by age, sex, and primary renal disease, unadjusted

prevalent patients on December 31

	All				0-19				20-44				45-64				65-74				75+				Male				Female				DM				Non-DM			
	HD	PD	Tx	Un	HD	PD	Tx	Un	HD	PD	Tx	Un	HD	PD	Tx	Un	HD	PD	Tx	Un	HD	PD	Tx	Un	HD	PD	Tx	Un	HD	PD	Tx	Un	HD	PD	Tx	Un	HD	PD	Tx	Un
	%	%	%	%	%	%	%	%	%	%	%	%	%	%	%	%	%	%	%	%	%	%	%	%	%	%	%	%	%	%	%	%	%	%	%	%	%	%	%	%
Austria	47	3	50	0	6	5	90	0	33	4	63	0	35	4	61	0	50	3	47	0	75	3	22	0	48	3	49	0	46	3	51	0	63	3	34	0	44	3	53	0
Belgium, Dutch-speaking *	52	4	44	0					31	6	64	0	32	4	64	0	46	5	49	0	81	3	15	0	52	4	44	0	52	4	44	0	70	4	26	0	49	4	48	0
Belgium, French-speaking *	53	4	43	0					38	5	57	0	38	4	58	0	53	4	42	0	72	5	23	0	52	4	43	0	53	5	42	0	69	4	26	0	49	4	46	0
Bosnia and Herzegovina	80	3	18	0	71	7	21	0	53	2	45	0	76	3	21	0	90	2	8	0	96	3	2	0	78	2	20	0	81	4	15	0	90	4	6	0	77	2	21	0
Denmark #	36	8	56	0			93	0	22	4	73		28	6	66	0	41	9	50		61	16	23	0	36	8	55	0	35	7	57		52	10	38		33	8	59	0
Estonia	36	4	60	0	0	9	91	0	23	2	74	0	28	3	69	0	38	5	57	0	64	6	30	0	36	4	60	0	35	4	60	0	39	5	56	0	35	4	61	0
France	52	3	45	0	17	6	77	0	32	2	66	0	39	2	58	0	56	3	41	1	75	5	19	1	52	3	44	0	51	3	45	1	73	3	23	0	48	3	49	1
Greece	77	4	19	0	28	7	65	0	47	4	49	0	66	4	30	0	81	4	14	0	93	3	4	0	78	3	19	0	75	4	20	0	92	3	5	0	74	4	22	0
Iceland	22	6	72	0	13	0	88	0	17	2	81	0	10	8	82	0	26	3	71	0	52	10	38	0	24	7	70	0	20	4	76	0	32	10	59	0	21	5	74	0
Netherlands	28	5	67	0	5	4	91	0	17	3	80	0	21	4	75	0	28	5	67	0	51	8	42	0	28	5	67	0	28	5	67	0	48	7	44	0	25	5	70	0
Norway	25	8	66	1	6	2	88	3	14	7	78	2	21	5	73	1	26	10	64	1	43	13	43	0	26	9	64	1	22	7	70	1	37	11	52	0	23	8	68	1
Romania | |	88	4	9	0	62	11	27	0	71	3	25	0	83	3	13	0	93	3	4	0	93	6	1	0	87	3	9	0	88	5	7	0	92	5	3	0	87	4	9	0
Serbia	76	9	15	0	23	36	41	0	58	7	34	0	70	9	20	0	85	8	7	0	89	9	2	0	77	7	15	0	73	11	15	0	79	14	7	0	75	8	17	0
Spain, Andalusia	40	3	57	0	10	2	89	0	25	4	71	0	27	3	70	0	42	4	54	0	70	4	25	0	39	3	57	0	41	4	56	0	59	4	37	0	36	3	61	0
Spain, Aragon	32	4	64	0	6	0	94	0	24	4	72	0	24	3	72	0	34	3	64	0	42	4	54	0	33	4	63	0	29	3	68	0	48	4	48	0	29	4	68	0
Spain, Basque country	29	5	66	0	6	0	94	0	26	3	70	0	22	4	74	0	27	6	67	0	45	8	47	0	30	6	64	0	27	4	69	0	53	6	41	0	25	5	70	0
Spain, Canary Islands	45	8	47	0	22	22	56	0	34	8	58	0	34	7	59	0	47	10	43	0	68	8	24	0	45	8	48	0	45	9	45	0	59	8	33	0	41	8	51	0
Spain, Cantabria *	33	4	63	0					21	3	77	0	17	3	80	0	27	3	69	0	65	6	30	0	34	3	63	0	31	5	64	0	36	5	59	0	32	3	64	0
Spain, Castile and León *	38	5	54	2					23	6	71	0	25	5	70	0	35	6	58	1	62	4	29	5	40	6	53	2	36	4	57	3	56	4	36	4	35	5	58	2
Spain, Castile-La Mancha *	38	6	56	0					22	10	67	0	27	6	67	0	38	6	55	0	59	4	37	0	40	6	55	0	35	6	58	0	55	7	38	0	34	6	60	0
Spain, Catalonia	34	3	63	0	9	2	89	0	23	3	74	0	22	3	75	0	31	4	65	0	59	4	37	0	35	4	62	0	32	3	64	0	49	4	47	0	31	3	66	0
Spain, Community of Madrid	39	5	56	0	13	2	84	0	28	6	66	0	29	5	66	0	38	6	56	0	60	5	35	0	40	6	54	0	36	5	59	0	58	4	38	0	35	6	59	0
Spain, Extremadura	53	3	44	0	33	0	67	0	28	6	66	0	40	3	56	0	57	3	40	0	79	2	19	0	55	3	42	0	49	3	47	0	71	3	27	0	50	3	47	0
Spain, Galicia	43	7	50	0	5	5	90	0	27	6	67	0	28	6	66	0	44	6	50	0	69	8	23	0	44	7	49	0	41	7	52	0	62	7	31	0	39	7	54	0
Spain, La Rioja	33	6	61	0	0	0	100	0	33	7	60	0	24	8	68	0	24	7	70	0	62	0	38	0	31	4	64	0	37	8	55	0	56	8	36	0	30	5	64	0
Spain, Murcia	47	5	49	0	14	0	86	0	36	6	58	0	32	5	63	0	47	5	48	0	76	4	19	0	47	4	49	0	46	5	49	0	66	6	28	0	43	4	53	0
Spain, Navarre *	34	5	61	0					24	7	69	0	23	3	74	0	35	6	59	0	55	5	39	0	37	5	59	0	29	5	65	0	51	5	44	0	31	5	65	0
Spain, Valencian region	46	6	48	0	15	6	79	0	25	5	70	0	33	5	62	0	46	7	47	0	72	6	22	0	47	6	47	0	45	5	50	0	65	6	30	0	43	6	51	0
Sweden	30	9	61	0	3	9	87	1	19	5	77	0	22	6	72	0	31	9	60	0	53	17	30	0	30	9	61	0	29	7	63	0	44	12	44	0	27	8	65	0
Switzerland	40	3	57	0	6	7	85	1	21	4	76	0	26	3	71	0	38	3	58	0	71	3	26	0	41	3	56	0	38	3	58	0	62	3	35	0	37	3	60	0
United Kingdom, England #	39	5	55	0	15	10	75	0	28	5	67		34	4	62	0	42	6	52	0	66	8	26	0	40	5	55		38	6	56		58	6	35	0	35	5	60	0
United Kingdom, Northern Ireland #	26	2	71	0			84	0	13		86	0	18	2	80	0	32		67	0	55	7	38	0	26	2	72	0	27	3	70	0	47	3	50	0	23	2	75	0
United Kingdom, Scotland	35	3	61	0	13	7	80	0	22	3	75	0	29	3	68	0	43	3	54	0	64	6	30	0	36	3	60	0	34	4	62	0	54	4	43	0	32	3	65	0
United Kingdom, Wales #	41	4	55	0	19		74	0	27	5	68	0	34	3	63	0	45	6	49	0	67	5	28	0	40	4	56	0	42	5	54	0	60	5	35	0	36	4	60	0

Abbreviations used: HD: haemodialysis; PD: peritoneal dialysis; Tx: transplant; Un: unknown; DM: diabetes mellitus
Categories may not add up due to missing values or rounding
* Patients younger than 20 years of age are not reported
# Values based on 1 to 5 patients are omitted
| | The prevalence of patients living with a functioning graft is underestimated by approximately 30%

Table B.5.1

Kidney transplant counts and percentages by donor type

	All		Living donor								Deceased donor		Donor type Unkn	
			Related		Unrelated		Type Unkn		All					
	N	%	N	%	N	%	N	%	N	%	N	%	N	%
Austria §	300	100	36	12.0	28	9.3	0	0	64	21.3	236	78.7	0	0
Belgium, Dutch-speaking *	268	100	14	5.2	8	3.0	5	1.9	27	10.1	197	73.5	44	16.4
Belgium, French-speaking *	176	100	13	7.4	21	11.9	0	0	34	19.3	139	79.0	3	1.7
Bosnia and Herzegovina	19	100	9	47.4	2	10.5	0	0	11	57.9	8	42.1	0	0
Denmark #	298	100	49	16.4	40	13.4	0	0	89	29.9	209	70.1	0	0
Estonia	51	100	5	9.8	0	0	0	0	5	9.8	46	90.2	0	0
France	3526	100	315	8.9	242	6.9	0	0	557	15.8	2967	84.1	2	0.1
Greece	250	100	70	28.0	30	12.0	0	0	100	40.0	150	60.0	0	0
Iceland	10	100	2	20.0	0	0	0	0	2	20.0	8	80.0	0	0
Netherlands &	983	100					487	49.5	487	49.5	496	50.5	0	0
Norway	239	100	30	12.6	17	7.1	0	0	47	19.7	192	80.3	0	0
Romania | |	177	100					57	32.2	57	32.2	120	67.8	0	0
Serbia ¤	59	100	22	37.3	0	0	4	6.8	26	44.1	32	54.2	1	1.7
Spain, Andalusia	642	100	37	5.8	19	3.0	0	0	56	8.7	586	91.3	0	0
Spain, Aragon	86	100	5	5.8	4	4.7	1	1.2	10	11.6	76	88.4	0	0
Spain, Basque country	160	100	16	10.0	3	1.9	0	0	19	11.9	141	88.1	0	0
Spain, Canary Islands	160	100					10	6.3	10	6.3	150	93.8	0	0
Spain, Cantabria *	65	100	1	1.5	0	0	0	0	1	1.5	64	98.5	0	0
Spain, Castile and León *	141	100	0	0	2	1.4	9	6.4	11	7.8	130	92.2	0	0
Spain, Castile-La Mancha *	87	100	0	0	0	0	3	3.4	3	3.4	83	95.4	1	1.1
Spain, Catalonia	976	100	96	9.8	91	9.3	0	0	187	19.2	789	80.8	0	0
Spain, Community of Madrid	426	100					66	15.5	66	15.5	359	84.3	1	0.2
Spain, Extremadura	66	100	2	3.0	0	0	1	1.5	3	4.5	63	95.5	0	0
Spain, Galicia	168	100	7	4.2	0	0	17	10.1	24	14.3	144	85.7	0	0
Spain, La Rioja	19	100					1	5.3	1	5.3	18	94.7	0	0
Spain, Murcia	132	100	5	3.8	5	3.8	0	0	10	7.6	122	92.4	0	0
Spain, Navarre *	42	100					0	0	0	0	42	100	0	0
Spain, Valencian region	355	100	0	0	0	0	15	4.2	15	4.2	340	95.8	0	0
Sweden	507	100	50	9.9	49	9.7	0	0	99	19.5	408	80.5	0	0
Switzerland	374	100	46	12.3	54	14.4	0	0	100	26.7	274	73.3	0	0
United Kingdom, England #	2429	100	356	14.7	244	10.0	48	2.0	648	26.7	1774	73.0	7	0.3
United Kingdom, Northern Ireland #	112	100	37	33.0	11	9.8	8	7.1	56	50.0	56	50.0	0	0
United Kingdom, Scotland	265	100	24	9.1	15	5.7	50	18.9	89	33.6	173	65.3	3	1.1
United Kingdom, Wales #	128	100	27	21.1	15	11.7	0	0	42	32.8	86	67.2	0	0

Categories may not add up due to rounding; When cells are left empty, (complete) data are unavailable
§ Data based on residents and non-residents
* Patients younger than 20 years of age are not reported
# Values based on 1 to 5 patients are omitted
& The transplant activity is underestimated by approximately 3%
| | The transplant activity is underestimated by approximately 30%
¤ The transplant activity is underestimated by approximately 16% for deceased donor transplants and by approximately 12% for living donor transplants due to centres not submitting complete data

Table B.5.2

Kidney transplants per million population by donor type, unadjusted

	All	Living donor				Deceased donor	Donor type Unkn
		Related	Unrelated	Type Unkn	All		
	Pmp	Pmp	Pmp	Pmp	Pmp	Pmp	Pmp
Austria §	33.6	4.0	3.1	0	7.2	26.4	0
Belgium, Dutch-speaking *	39.3	2.1	1.2	0.7	4.0	28.9	6.5
Belgium, French-speaking *	35.4	2.6	4.2	0	6.8	28.0	0.6
Bosnia and Herzegovina	5.4	2.5	0.6	0	3.1	2.3	0
Denmark #	50.1	8.2	6.7	0	15.0	35.1	0
Estonia	39.2	3.8	0	0	3.8	35.3	0
France	51.6	4.6	3.5	0	8.1	43.4	0
Greece	24.0	6.7	2.9	0	9.6	14.4	0
Iceland	25.9	5.2	0	0	5.2	20.7	0
Netherlands	56.7			28.1	28.1	28.6	0
Norway	43.3	5.4	3.1	0	8.5	34.8	0
Romania | |	9.3			3.0	3.0	6.3	0
Serbia ¤	9.2	3.4	0	0.6	4.1	5.0	0.2
Spain, Andalusia	74.6	4.3	2.2	0	6.5	68.1	0
Spain, Aragon	63.9	3.7	3.0	0.7	7.4	56.4	0
Spain, Basque country	72.0	7.2	1.4	0	8.6	63.5	0
Spain, Canary Islands	71.9			4.5	4.5	67.4	0
Spain, Cantabria *	110.2	1.7	0	0	1.7	108.5	0
Spain, Castile and León *	59.2	0	0.8	3.8	4.6	54.5	0
Spain, Castile-La Mancha *	41.5	0	0	1.4	1.4	39.6	0.5
Spain, Catalonia	123.5	12.1	11.5	0	23.7	99.8	0
Spain, Community of Madrid	62.0			9.6	9.6	52.2	0.1
Spain, Extremadura	62.6	1.9	0	0.9	2.8	59.8	0
Spain, Galicia	62.2	2.6	0	6.3	8.9	53.3	0
Spain, La Rioja	58.8			3.1	3.1	55.7	0
Spain, Murcia	85.1	3.2	3.2	0	6.4	78.6	0
Spain, Navarre *	62.2			0	0	62.2	0
Spain, Valencian region	68.1	0	0	2.9	2.9	65.2	0
Sweden	48.1	4.7	4.7	0	9.4	38.7	0
Switzerland	44.3	5.4	6.4	0	11.8	32.4	0
United Kingdom, England #	45.8	6.7	4.6	0.9	12.2	33.4	0.1
United Kingdom, Northern Ireland #	58.3	19.3	5.7	4.2	29.2	29.2	0
United Kingdom, Scotland	48.3	4.4	2.7	9.1	16.2	31.5	0.5
United Kingdom, Wales #	40.4	8.5	4.7	0	13.3	27.2	0

Categories may not add up due to rounding; When cells are left empty, (complete) data are unavailable
§ The number of transplants may differ from table B.5.1 as non-residents are excluded from this analysis
* Patients younger than 20 years of age are not reported
# Values based on 1 to 5 patients are omitted
| | The transplant activity is underestimated by approximately 30%
¤ The transplant activity is underestimated by approximately 16% for deceased donor transplants and by approximately 12% for living donor transplants due to centres not submitting complete data

Table B.5.3

Sex, mean age, and median age of kidney transplant recipients
at time of transplantation

	All						Male						Female					
	%	Mean (years)	SD	Median (years)	P25	P75	%	Mean (years)	SD	Median (years)	P25	P75	%	Mean (years)	SD	Median (years)	P25	P75
Austria	100	51.2	16.6	55.4	40.0	63.8	65.0	52.3	16.9	56.4	43.6	64.7	35.0	49.2	15.9	52.0	37.7	62.9
Belgium, Dutch-speaking *	100	55.1	12.6	57.4	47.2	64.2	63.8	55.3	12.5	57.7	47.9	63.9	36.2	54.8	12.7	57.1	45.3	65.2
Belgium, French-speaking *	100	55.8	13.2	58.0	47.0	64.7	61.9	55.2	13.7	57.7	45.6	64.3	38.1	56.9	12.4	58.8	48.4	65.5
Bosnia and Herzegovina	100	44.7	12.8	44.3	33.8	58.2	68.4	43.4	13.2	40.6	37.3	47.1	31.6	47.5	12.7	48.4	33.5	58.2
Denmark	100	52.1	15.2	53.8	44.0	62.6	67.1	52.0	15.2	54.4	44.1	61.6	32.9	52.3	15.2	52.8	42.6	64.1
Estonia	100	51.1	13.1	52.6	40.2	61.5	52.9	50.7	13.2	52.6	40.2	61.3	47.1	51.7	13.2	52.0	40.7	63.6
France	100	54.4	16.5	56.1	44.1	67.2	63.2	54.7	16.8	56.6	44.3	67.8	36.8	53.8	16.0	55.5	43.5	65.9
Greece	100	47.5	15.1	49.0	39.7	58.2	72.0	47.7	15.3	50.1	39.2	58.2	28.0	47.2	14.7	46.6	41.7	56.9
Iceland	100	50.1	17.3	48.1	32.8	66.5	90.0	52.0	17.2	52.9	39.4	66.5	10.0	32.8	0	32.8	32.8	32.8
Netherlands	100	54.1	16.2	56.9	44.4	66.7	61.5	54.6	16.4	57.7	45.2	67.7	38.5	53.2	15.9	56.0	44.0	66.0
Norway	100	52.2	17.1	54.1	40.5	65.4	61.9	51.3	17.9	52.0	39.0	65.2	38.1	53.6	15.7	57.3	43.7	65.6
Romania	100	43.5	12.8	44.6	35.2	52.2	68.9	43.6	12.4	44.6	35.8	52.1	31.1	43.2	13.7	44.7	31.6	54.5
Serbia	100	39.5	16.7	40.1	24.4	55.6	76.3	40.0	18.1	40.1	22.3	55.9	23.7	37.9	11.6	38.0	29.6	45.4
Spain, Andalusia	100	55.7	14.5	57.3	46.6	66.8	63.4	55.9	14.4	57.3	47.3	67.0	36.6	55.5	14.6	57.3	45.3	66.7
Spain, Aragon	100	55.5	16.8	58.3	46.3	66.9	62.8	56.0	16.6	58.3	47.7	66.5	37.2	54.6	17.4	56.0	45.1	68.7
Spain, Basque country	100	57.5	17.5	61.2	49.4	71.1	62.5	60.6	15.5	64.2	51.3	72.0	37.5	52.4	19.4	55.3	42.5	66.2
Spain, Canary Islands	100	54.5	14.2	56.8	45.2	64.8	68.1	56.5	13.2	58.3	49.2	65.9	31.9	50.2	15.4	51.8	38.3	61.5
Spain, Cantabria *	100	58.2	13.5	61.7	49.7	69.2	73.8	59.6	13.8	63.7	51.5	69.6	26.2	54.5	12.1	56.1	44.2	64.5
Spain, Castile and León *	100	58.0	12.5	58.7	49.8	68.1	69.5	58.4	12.4	59.6	51.7	68.8	30.5	57.0	12.8	57.8	48.2	63.3
Spain, Castile-La Mancha *	100	57.3	13.8	59.3	51.9	67.0	62.1	58.5	13.9	61.6	53.4	67.9	37.9	55.2	13.6	56.0	47.7	63.8
Spain, Catalonia	100	59.4	15.9	62.5	49.6	71.6	63.5	59.8	16.1	63.3	50.3	72.6	36.5	58.7	15.5	61.6	48.5	70.5
Spain, Community of Madrid	100	52.9	18.0	56.8	43.6	66.0	65.5	53.7	17.9	57.7	44.3	66.5	34.0	51.9	18.1	53.9	42.4	65.2
Spain, Extremadura	100	58.4	12.6	59.4	52.0	66.8	60.6	58.1	11.5	60.5	54.3	63.5	39.4	58.8	14.2	58.4	50.3	73.4
Spain, Galicia	100	58.4	12.5	59.3	50.6	68.0	61.3	58.7	12.3	59.5	50.7	68.0	38.7	57.8	13.0	58.1	49.7	68.0
Spain, La Rioja	100	60.9	15.6	66.2	50.9	70.5	68.4	61.2	18.0	67.8	50.9	70.5	31.6	60.3	9.7	63.5	52.7	66.2
Spain, Murcia	100	54.7	15.8	56.9	47.3	67.4	60.6	56.1	15.5	56.8	49.3	68.1	39.4	52.6	16.1	56.9	40.3	63.8
Spain, Navarre *	100	58.9	13.8	59.8	50.5	68.2	54.8	60.2	15.1	61.7	57.3	69.5	45.2	57.4	12.2	59.2	46.2	68.2
Spain, Valencian region	100	56.6	13.7	58.3	49.0	66.4	64.5	56.1	13.8	57.9	48.6	65.6	35.5	57.4	13.5	59.2	49.4	67.5
Sweden	100	52.9	15.4	55.3	43.3	65.0	62.5	53.3	15.5	55.6	43.6	65.2	37.5	52.1	15.2	53.7	42.7	64.1
Switzerland	100	54.0	15.2	56.7	43.8	66.0	63.6	54.2	15.7	57.4	43.6	66.3	36.4	53.6	14.3	56.1	44.4	65.0
United Kingdom, England	100	50.0	16.4	52.9	38.6	62.7	60.6	50.7	16.7	53.7	38.8	63.6	39.4	49.0	15.9	51.2	38.3	61.2
United Kingdom, Northern Ireland	100	52.5	14.8	53.4	43.7	63.4	71.4	52.1	15.8	52.3	43.3	64.8	28.6	53.6	12.2	54.3	43.9	61.6
United Kingdom, Scotland	100	52.3	15.2	54.2	42.3	64.8	63.4	50.4	15.7	52.9	39.7	62.2	36.2	55.5	13.7	56.4	48.8	67.2
United Kingdom, Wales	100	49.3	13.5	50.3	40.5	59.0	64.8	50.1	14.1	51.5	41.6	61.0	35.2	47.7	12.4	48.1	38.3	57.2

Categories may not add up due to missing values or rounding
* Patients younger than 20 years of age are not reported

Table B.6.1

Incident KRT patients, from day 1, unadjusted

by age, sex, and primary renal disease

	Patient survival probabilities as % (95% CI)						
	cohort 2014 - 2018				cohort 2017 - 2021		
	90 day	1 year	2 year	5 year	90 day	1 year	2 year
0-19	99.5 (99.1-99.7)	98.0 (97.4-98.5)	97.2 (96.4-97.8)	95.0 (94.0-95.8)	99.3 (98.8-99.5)	97.8 (97.1-98.3)	97.1 (96.4-97.7)
20-44	99.2 (99.0-99.3)	97.0 (96.8-97.3)	94.8 (94.5-95.1)	89.0 (88.5-89.5)	99.1 (99.0-99.2)	96.8 (96.6-97.1)	94.3 (93.9-94.6)
45-64	97.5 (97.4-97.7)	92.0 (91.8-92.2)	85.8 (85.5-86.1)	69.1 (68.7-69.5)	97.6 (97.5-97.8)	92.2 (92.0-92.4)	85.9 (85.6-86.2)
65-74	95.2 (95.0-95.4)	84.9 (84.6-85.2)	74.1 (73.7-74.5)	46.1 (45.6-46.5)	95.1 (94.9-95.3)	85.0 (84.7-85.3)	74.2 (73.8-74.6)
75+	91.5 (91.3-91.7)	75.8 (75.4-76.1)	60.0 (59.6-60.4)	25.4 (25.0-25.8)	91.6 (91.3-91.8)	76.4 (76.0-76.7)	60.8 (60.4-61.2)
Male	95.2 (95.1-95.3)	85.3 (85.1-85.5)	75.0 (74.7-75.2)	50.1 (49.8-50.3)	95.1 (95.0-95.3)	85.4 (85.2-85.6)	75.0 (74.8-75.3)
Female	95.2 (95.0-95.3)	85.9 (85.6-86.2)	76.5 (76.1-76.8)	53.7 (53.3-54.1)	95.2 (95.0-95.4)	86.3 (86.0-86.6)	76.9 (76.5-77.2)
Diabetes	96.1 (95.9-96.3)	86.5 (86.2-86.8)	74.8 (74.3-75.2)	44.5 (44.0-45.0)	96.0 (95.8-96.2)	86.4 (86.1-86.7)	74.5 (74.1-74.9)
Hypertension / renal vascular disease	95.0 (94.8-95.2)	84.4 (84.0-84.7)	72.6 (72.1-73.1)	43.4 (42.9-44.0)	95.1 (94.9-95.3)	84.8 (84.4-85.2)	73.0 (72.5-73.5)
Glomerulonephritis	97.9 (97.7-98.0)	93.2 (92.8-93.5)	88.1 (87.6-88.5)	72.6 (72.0-73.3)	98.1 (97.9-98.2)	93.7 (93.3-94.0)	88.4 (88.0-88.9)
Other causes	94.4 (94.2-94.5)	83.9 (83.7-84.2)	74.3 (74.0-74.6)	53.1 (52.8-53.5)	94.4 (94.2-94.6)	84.3 (84.0-84.5)	74.8 (74.5-75.1)
All	95.2 (95.1-95.3)	85.5 (85.3-85.7)	75.5 (75.3-75.7)	51.3 (51.1-51.6)	95.2 (95.1-95.3)	85.7 (85.5-85.9)	75.7 (75.5-75.9)

Based on data from Austria, Belgium (Dutch-speaking), Belgium (French-speaking), Bosnia and Herzegovina, Denmark, Estonia, France, Greece, Iceland, Netherlands, Norway, Spain (Andalusia), Spain (Aragon), Spain (Basque country), Spain (Canary Islands), Spain (Cantabria), Spain (Castile and León), Spain (Castile-La Mancha), Spain (Catalonia), Spain (Community of Madrid), Spain (Extremadura), Spain (Galicia), Spain (Murcia), Spain (Navarre), Spain (Valencian Region), Sweden, United Kingdom (England/Northern Ireland/Wales) and United Kingdom (Scotland)

Table B.6.2

Incident KRT patients, from day 1, adjusted

adjusted for age, sex, and primary renal disease

	Patient survival probabilities as % (95% CI)						
	cohort 2014 - 2018				cohort 2017 - 2021		
	90 day	1 year	2 year	5 year	90 day	1 year	2 year
0-19	99.5 (99.2-99.8)	97.9 (97.3-98.5)	97.0 (96.3-97.7)	94.7 (93.7-95.6)	99.2 (98.9-99.6)	97.7 (97.0-98.3)	97.0 (96.3-97.7)
20-44	99.2 (99.1-99.3)	97.0 (96.7-97.2)	94.7 (94.3-95.0)	88.6 (88.1-89.1)	99.1 (99.0-99.3)	96.8 (96.5-97.1)	94.2 (93.8-94.5)
45-64	97.7 (97.6-97.8)	92.3 (92.1-92.5)	86.2 (85.9-86.5)	69.4 (69.0-69.8)	97.8 (97.7-97.9)	92.5 (92.3-92.8)	86.3 (86.0-86.6)
65-74	95.5 (95.4-95.7)	85.7 (85.4-86.0)	75.2 (74.8-75.6)	47.4 (46.9-47.9)	95.4 (95.2-95.6)	85.7 (85.4-86.0)	75.2 (74.9-75.6)
75+	91.8 (91.6-92.1)	76.5 (76.1-76.8)	60.9 (60.5-61.3)	26.2 (25.8-26.6)	92.0 (91.8-92.2)	77.3 (76.9-77.6)	61.9 (61.5-62.3)
Male	96.3 (96.2-96.4)	88.1 (87.9-88.3)	78.8 (78.6-79.0)	52.5 (52.2-52.8)	96.2 (96.1-96.3)	88.0 (87.8-88.2)	78.6 (78.4-78.9)
Female	96.3 (96.1-96.4)	88.6 (88.4-88.9)	80.2 (79.9-80.5)	56.5 (56.1-56.9)	96.3 (96.1-96.4)	88.8 (88.5-89.0)	80.2 (79.9-80.5)
Diabetes	96.7 (96.5-96.8)	88.4 (88.1-88.7)	77.6 (77.3-78.0)	46.6 (46.1-47.1)	96.6 (96.4-96.8)	88.3 (88.0-88.5)	77.4 (77.0-77.8)
Hypertension / renal vascular disease	96.9 (96.8-97.1)	90.0 (89.7-90.3)	81.6 (81.3-82.0)	55.9 (55.4-56.4)	96.8 (96.7-97.0)	89.7 (89.4-90.0)	81.0 (80.6-81.4)
Glomerulonephritis	97.6 (97.3-97.8)	92.0 (91.6-92.4)	85.8 (85.3-86.3)	65.6 (64.9-66.3)	97.8 (97.6-98.0)	92.6 (92.3-93.0)	86.3 (85.8-86.8)
Other causes	95.3 (95.2-95.5)	86.2 (86.0-86.4)	77.1 (76.8-77.4)	53.8 (53.5-54.2)	95.3 (95.2-95.4)	86.3 (86.1-86.5)	77.3 (77.0-77.6)
All	96.3 (96.2-96.4)	88.3 (88.2-88.5)	79.4 (79.2-79.6)	54.0 (53.8-54.3)	96.2 (96.1-96.3)	88.3 (88.2-88.5)	79.3 (79.1-79.5)

Based on data from Austria, Belgium (Dutch-speaking), Belgium (French-speaking), Bosnia and Herzegovina, Denmark, Estonia, France, Greece, Iceland, Netherlands, Norway, Spain (Andalusia), Spain (Aragon), Spain (Basque country), Spain (Canary Islands), Spain (Cantabria), Spain (Castile and León), Spain (Castile-La Mancha), Spain (Catalonia), Spain (Community of Madrid), Spain (Extremadura), Spain (Galicia), Spain (Murcia), Spain (Navarre), Spain (Valencian Region), Sweden, United Kingdom (England/Northern Ireland/Wales) and United Kingdom (Scotland)

Table B.6.3

Incident KRT patients, from day 91, unadjusted

by age, sex, and primary renal disease

	Patient survival probabilities as % (95% CI)				
	cohort 2014 - 2018			cohort 2017 - 2021	
	1 year	2 year	5 year	1 year	2 year
0-19	98.3 (97.7-98.8)	97.6 (96.9-98.1)	95.4 (94.4-96.1)	98.2 (97.6-98.7)	97.7 (97.0-98.3)
20-44	97.2 (97.0-97.4)	95.0 (94.7-95.3)	89.4 (88.9-89.8)	97.1 (96.8-97.3)	94.6 (94.2-94.9)
45-64	92.8 (92.6-93.1)	86.6 (86.3-86.9)	69.7 (69.3-70.1)	92.8 (92.6-93.0)	86.6 (86.3-86.9)
65-74	86.5 (86.2-86.8)	75.1 (74.7-75.5)	46.6 (46.1-47.0)	86.7 (86.4-87.0)	75.6 (75.2-76.0)
75+	78.7 (78.4-79.1)	61.8 (61.4-62.3)	25.8 (25.4-26.2)	79.4 (79.1-79.8)	62.7 (62.3-63.1)
Male	87.0 (86.8-87.2)	76.3 (76.0-76.5)	51.1 (50.8-51.4)	87.1 (86.9-87.3)	76.5 (76.2-76.7)
Female	87.9 (87.7-88.2)	78.2 (77.9-78.6)	55.0 (54.6-55.4)	88.3 (88.1-88.6)	78.7 (78.4-79.0)
Diabetes	87.1 (86.8-87.5)	74.9 (74.4-75.3)	44.4 (43.9-44.9)	87.2 (86.9-87.6)	74.9 (74.4-75.3)
Hypertension / renal vascular disease	85.9 (85.5-86.2)	73.7 (73.2-74.2)	43.9 (43.3-44.5)	86.1 (85.7-86.5)	74.2 (73.7-74.7)
Glomerulonephritis	94.0 (93.6-94.3)	88.7 (88.2-89.1)	73.1 (72.5-73.8)	94.1 (93.7-94.4)	88.9 (88.5-89.3)
Other causes	86.5 (86.3-86.8)	76.6 (76.3-76.9)	55.1 (54.8-55.5)	86.8 (86.6-87.1)	77.1 (76.8-77.4)
All	87.4 (87.2-87.5)	77.0 (76.8-77.2)	52.5 (52.2-52.7)	87.5 (87.4-87.7)	77.3 (77.1-77.5)

Based on data from Austria, Belgium (Dutch-speaking), Belgium (French-speaking), Bosnia and Herzegovina, Denmark, Estonia, France, Greece, Iceland, Netherlands, Norway, Spain (Andalusia), Spain (Aragon), Spain (Basque country), Spain (Canary Islands), Spain (Cantabria), Spain (Castile and León), Spain (Castile-La Mancha), Spain (Catalonia), Spain (Community of Madrid), Spain (Extremadura), Spain (Galicia), Spain (Murcia), Spain (Navarre), Spain (Valencian Region), Sweden, United Kingdom (England/Northern Ireland/Wales) and United Kingdom (Scotland)

Table B.6.4

Incident KRT patients, from day 91, adjusted

adjusted for age, sex, and primary renal disease

	Patient survival probabilities as % (95% CI)				
	cohort 2014 - 2018			cohort 2017 - 2021	
	1 year	2 year	5 year	1 year	2 year
0-19	98.2 (97.7-98.8)	97.4 (96.8-98.1)	95.0 (94.1-96.0)	98.1 (97.5-98.7)	97.6 (96.9-98.2)
20-44	97.1 (96.8-97.3)	94.8 (94.5-95.2)	88.8 (88.4-89.3)	97.0 (96.7-97.2)	94.4 (94.1-94.8)
45-64	93.0 (92.8-93.3)	86.9 (86.6-87.2)	69.9 (69.5-70.3)	93.0 (92.8-93.2)	86.9 (86.6-87.2)
65-74	87.2 (86.8-87.5)	76.2 (75.8-76.6)	47.9 (47.4-48.4)	87.3 (87.0-87.6)	76.5 (76.2-76.9)
75+	79.3 (79.0-79.7)	62.7 (62.3-63.1)	26.6 (26.2-27.0)	80.1 (79.7-80.4)	63.7 (63.3-64.1)
Male	89.3 (89.1-89.5)	79.6 (79.4-79.9)	52.9 (52.5-53.2)	89.2 (89.1-89.4)	79.6 (79.3-79.8)
Female	90.1 (89.9-90.3)	81.4 (81.1-81.7)	57.1 (56.7-57.5)	90.2 (89.9-90.4)	81.4 (81.1-81.7)
Diabetes	88.8 (88.6-89.1)	77.5 (77.1-77.9)	46.1 (45.6-46.6)	88.9 (88.6-89.2)	77.5 (77.1-77.9)
Hypertension / renal vascular disease	90.9 (90.7-91.2)	82.3 (81.9-82.6)	55.9 (55.3-56.4)	90.6 (90.3-90.8)	81.6 (81.2-82.0)
Glomerulonephritis	92.9 (92.5-93.3)	86.3 (85.8-86.8)	65.7 (65.0-66.5)	93.0 (92.6-93.4)	86.7 (86.2-87.2)
Other causes	88.2 (88.0-88.4)	78.8 (78.5-79.0)	55.0 (54.6-55.4)	88.4 (88.1-88.6)	79.1 (78.8-79.4)
All	89.6 (89.5-89.8)	80.3 (80.1-80.5)	54.5 (54.2-54.7)	89.6 (89.5-89.7)	80.3 (80.1-80.5)

Based on data from Austria, Belgium (Dutch-speaking), Belgium (French-speaking), Bosnia and Herzegovina, Denmark, Estonia, France, Greece, Iceland, Netherlands, Norway, Spain (Andalusia), Spain (Aragon), Spain (Basque country), Spain (Canary Islands), Spain (Cantabria), Spain (Castile and León), Spain (Castile-La Mancha), Spain (Catalonia), Spain (Community of Madrid), Spain (Extremadura), Spain (Galicia), Spain (Murcia), Spain (Navarre), Spain (Valencian Region), Sweden, United Kingdom (England/Northern Ireland/Wales) and United Kingdom (Scotland)

Table B.6.5

Incident dialysis patients, from day 1, unadjusted

by age, sex, and primary renal disease

	Patient survival probabilities as % (95% CI)						
	cohort 2014 - 2018				cohort 2017 - 2021		
	90 day	1 year	2 year	5 year	90 day	1 year	2 year
0-19	99.4 (98.9-99.7)	97.3 (96.3-98.0)	95.5 (94.1-96.6)	87.1 (83.1-90.2)	99.3 (98.8-99.6)	97.2 (96.3-97.9)	96.2 (95.0-97.1)
20-44	99.1 (98.9-99.2)	96.4 (96.1-96.7)	92.8 (92.3-93.2)	79.6 (78.6-80.5)	99.0 (98.8-99.2)	96.2 (95.8-96.5)	92.3 (91.8-92.7)
45-64	97.4 (97.2-97.5)	91.2 (90.9-91.4)	83.6 (83.2-83.9)	58.9 (58.4-59.4)	97.5 (97.4-97.6)	91.4 (91.2-91.7)	83.9 (83.5-84.2)
65-74	95.1 (94.9-95.3)	84.4 (84.0-84.7)	72.8 (72.3-73.2)	41.0 (40.5-41.5)	94.9 (94.7-95.1)	84.4 (84.1-84.7)	72.9 (72.5-73.4)
75+	91.5 (91.2-91.7)	75.7 (75.3-76.0)	59.7 (59.3-60.1)	24.7 (24.3-25.0)	91.5 (91.3-91.7)	76.2 (75.9-76.6)	60.5 (60.1-60.9)
Male	94.9 (94.8-95.1)	84.2 (84.0-84.5)	72.3 (72.0-72.6)	39.9 (39.6-40.3)	94.9 (94.8-95.0)	84.5 (84.2-84.7)	72.7 (72.4-72.9)
Female	94.9 (94.7-95.0)	84.8 (84.5-85.1)	73.8 (73.4-74.2)	44.0 (43.5-44.4)	94.9 (94.7-95.1)	85.3 (85.0-85.5)	74.4 (74.1-74.8)
Diabetes	96.0 (95.8-96.2)	86.1 (85.8-86.4)	73.5 (73.1-73.9)	38.6 (38.1-39.2)	95.9 (95.7-96.1)	86.1 (85.7-86.4)	73.5 (73.1-73.9)
Hypertension / renal vascular disease	94.9 (94.7-95.2)	83.9 (83.5-84.3)	71.3 (70.8-71.9)	37.8 (37.3-38.4)	95.0 (94.8-95.3)	84.4 (84.0-84.8)	71.8 (71.3-72.4)
Glomerulonephritis	97.6 (97.4-97.9)	92.2 (91.8-92.6)	85.4 (84.9-86.0)	59.6 (58.7-60.5)	97.9 (97.7-98.1)	92.8 (92.5-93.2)	86.0 (85.5-86.5)
Other causes	93.9 (93.8-94.1)	82.3 (82.0-82.6)	70.7 (70.3-71.0)	41.6 (41.2-42.0)	94.0 (93.8-94.2)	82.8 (82.5-83.1)	71.6 (71.2-71.9)
All	94.9 (94.8-95.0)	84.4 (84.3-84.6)	72.8 (72.6-73.0)	41.4 (41.1-41.6)	94.9 (94.8-95.0)	84.7 (84.6-84.9)	73.3 (73.1-73.5)

Based on data from Austria, Belgium (Dutch-speaking), Belgium (French-speaking), Bosnia and Herzegovina, Denmark, Estonia, France, Greece, Iceland, Netherlands, Norway, Spain (Andalusia), Spain (Aragon), Spain (Basque country), Spain (Canary Islands), Spain (Cantabria), Spain (Castile and León), Spain (Castile-La Mancha), Spain (Catalonia), Spain (Community of Madrid), Spain (Extremadura), Spain (Galicia), Spain (Murcia), Spain (Navarre), Spain (Valencian Region), Sweden, United Kingdom (England/Northern Ireland/Wales) and United Kingdom (Scotland)

Table B.6.6

Incident dialysis patients, from day 1, adjusted

adjusted for age, sex, and primary renal disease

	Patient survival probabilities as % (95% CI)						
	cohort 2014 - 2018				cohort 2017 - 2021		
	90 day	1 year	2 year	5 year	90 day	1 year	2 year
0-19	99.4 (99.0-99.8)	97.2 (96.3-98.0)	95.3 (94.1-96.6)	86.7 (83.2-90.4)	99.3 (98.9-99.7)	97.1 (96.2-98.0)	96.0 (94.9-97.1)
20-44	99.1 (99.0-99.3)	96.4 (96.1-96.7)	92.7 (92.2-93.2)	79.3 (78.4-80.3)	99.0 (98.9-99.2)	96.2 (95.8-96.5)	92.2 (91.8-92.7)
45-64	97.5 (97.4-97.7)	91.5 (91.2-91.7)	84.0 (83.6-84.3)	59.5 (59.0-60.1)	97.7 (97.6-97.8)	91.8 (91.6-92.1)	84.4 (84.0-84.7)
65-74	95.4 (95.2-95.6)	85.0 (84.7-85.4)	73.6 (73.2-74.1)	42.1 (41.7-42.7)	95.3 (95.1-95.5)	85.1 (84.8-85.4)	73.9 (73.5-74.3)
75+	91.8 (91.5-92.0)	76.2 (75.9-76.6)	60.4 (60.0-60.8)	25.2 (24.8-25.6)	91.9 (91.7-92.1)	77.0 (76.7-77.4)	61.4 (61.0-61.8)
Male	95.8 (95.7-95.9)	86.5 (86.3-86.7)	75.8 (75.5-76.1)	45.1 (44.7-45.4)	95.8 (95.7-95.9)	86.8 (86.6-87.0)	76.4 (76.1-76.6)
Female	95.7 (95.6-95.9)	87.0 (86.8-87.3)	77.3 (77.0-77.7)	49.5 (49.0-49.9)	95.9 (95.7-96.0)	87.6 (87.4-87.9)	78.1 (77.7-78.4)
Diabetes	96.4 (96.2-96.6)	87.4 (87.1-87.7)	75.7 (75.3-76.1)	41.6 (41.1-42.1)	96.4 (96.2-96.6)	87.5 (87.2-87.8)	76.0 (75.6-76.4)
Hypertension / renal vascular disease	96.4 (96.2-96.6)	88.4 (88.1-88.7)	78.6 (78.2-79.1)	49.0 (48.4-49.6)	96.5 (96.3-96.6)	88.6 (88.3-88.9)	78.8 (78.4-79.2)
Glomerulonephritis	97.3 (97.1-97.6)	91.2 (90.8-91.7)	84.0 (83.4-84.6)	57.5 (56.5-58.4)	97.6 (97.4-97.9)	92.0 (91.6-92.4)	84.6 (84.0-85.2)
Other causes	94.7 (94.5-94.8)	84.3 (84.0-84.5)	73.7 (73.4-74.0)	45.9 (45.5-46.3)	94.8 (94.7-95.0)	84.9 (84.6-85.1)	74.7 (74.4-75.0)
All	95.8 (95.7-95.9)	86.7 (86.6-86.9)	76.4 (76.2-76.6)	46.7 (46.4-47.0)	95.9 (95.8-96.0)	87.1 (87.0-87.3)	77.0 (76.8-77.2)

Based on data from Austria, Belgium (Dutch-speaking), Belgium (French-speaking), Bosnia and Herzegovina, Denmark, Estonia, France, Greece, Iceland, Netherlands, Norway, Spain (Andalusia), Spain (Aragon), Spain (Basque country), Spain (Canary Islands), Spain (Cantabria), Spain (Castile and León), Spain (Castile-La Mancha), Spain (Catalonia), Spain (Community of Madrid), Spain (Extremadura), Spain (Galicia), Spain (Murcia), Spain (Navarre), Spain (Valencian Region), Sweden, United Kingdom (England/Northern Ireland/Wales) and United Kingdom (Scotland)

Table B.6.7

Incident dialysis patients, from day 91, unadjusted

by age, sex, and primary renal disease

	Patient survival probabilities as % (95% CI)				
	cohort 2014 - 2018			cohort 2017 - 2021	
	1 year	2 year	5 year	1 year	2 year
0-19	97.4 (96.4-98.1)	96.0 (94.6-97.0)	87.0 (82.8-90.3)	97.3 (96.3-98.1)	96.6 (95.3-97.5)
20-44	96.4 (96.0-96.7)	92.6 (92.1-93.1)	78.9 (77.9-79.9)	96.2 (95.9-96.5)	92.3 (91.8-92.7)
45-64	91.9 (91.6-92.1)	84.0 (83.6-84.3)	58.6 (58.0-59.1)	91.9 (91.6-92.1)	84.3 (83.9-84.6)
65-74	85.9 (85.5-86.2)	73.5 (73.1-74.0)	41.1 (40.5-41.6)	86.1 (85.8-86.4)	74.2 (73.7-74.6)
75+	78.6 (78.2-79.0)	61.5 (61.1-61.9)	25.0 (24.6-25.4)	79.3 (78.9-79.6)	62.3 (61.9-62.8)
Male	85.8 (85.6-86.0)	73.1 (72.8-73.4)	40.0 (39.6-40.3)	86.1 (85.8-86.3)	73.7 (73.4-74.0)
Female	86.7 (86.4-87.0)	75.2 (74.8-75.5)	44.3 (43.9-44.8)	87.2 (86.9-87.5)	75.9 (75.5-76.3)
Diabetes	86.6 (86.3-87.0)	73.3 (72.9-73.8)	38.0 (37.5-38.5)	86.8 (86.5-87.1)	73.6 (73.2-74.0)
Hypertension / renal vascular disease	85.3 (84.9-85.7)	72.2 (71.7-72.8)	37.7 (37.1-38.3)	85.6 (85.2-86.0)	72.8 (72.3-73.3)
Glomerulonephritis	92.9 (92.5-93.3)	85.5 (84.9-86.1)	59.0 (58.1-60.0)	93.1 (92.7-93.5)	86.1 (85.5-86.6)
Other causes	84.8 (84.5-85.1)	72.5 (72.1-72.8)	42.4 (41.9-42.8)	85.3 (85.0-85.5)	73.5 (73.1-73.8)
All	86.1 (86.0-86.3)	73.8 (73.6-74.1)	41.5 (41.2-41.8)	86.4 (86.3-86.6)	74.5 (74.3-74.7)

Based on data from Austria, Belgium (Dutch-speaking), Belgium (French-speaking), Bosnia and Herzegovina, Denmark, Estonia, France, Greece, Iceland, Netherlands, Norway, Spain (Andalusia), Spain (Aragon), Spain (Basque country), Spain (Canary Islands), Spain (Cantabria), Spain (Castile and León), Spain (Castile-La Mancha), Spain (Catalonia), Spain (Community of Madrid), Spain (Extremadura), Spain (Galicia), Spain (Murcia), Spain (Navarre), Spain (Valencian Region), Sweden, United Kingdom (England/Northern Ireland/Wales) and United Kingdom (Scotland)

Table B.6.8

Incident dialysis patients, from day 91, adjusted

adjusted for age, sex, and primary renal disease

	Patient survival probabilities as % (95% CI)				
	cohort 2014 - 2018			cohort 2017 - 2021	
	1 year	2 year	5 year	1 year	2 year
0-19	97.3 (96.4-98.2)	95.7 (94.5-97.0)	86.4 (82.6-90.4)	97.2 (96.3-98.1)	96.3 (95.2-97.5)
20-44	96.3 (95.9-96.6)	92.4 (91.9-93.0)	78.6 (77.6-79.7)	96.2 (95.9-96.5)	92.2 (91.7-92.7)
45-64	92.1 (91.8-92.3)	84.3 (84.0-84.7)	59.2 (58.7-59.8)	92.1 (91.9-92.4)	84.7 (84.3-85.0)
65-74	86.4 (86.1-86.7)	74.4 (74.0-74.8)	42.2 (41.7-42.8)	86.6 (86.3-86.9)	75.0 (74.6-75.4)
75+	79.0 (78.7-79.4)	62.1 (61.7-62.5)	25.5 (25.1-25.9)	79.8 (79.5-80.2)	63.1 (62.7-63.6)
Male	87.7 (87.5-87.9)	76.4 (76.1-76.6)	44.8 (44.4-45.2)	88.0 (87.8-88.2)	77.1 (76.8-77.3)
Female	88.5 (88.3-88.8)	78.3 (78.0-78.7)	49.5 (49.0-50.0)	89.0 (88.7-89.2)	79.0 (78.7-79.4)
Diabetes	87.8 (87.5-88.1)	75.4 (75.0-75.8)	40.7 (40.1-41.2)	88.1 (87.8-88.4)	75.9 (75.5-76.3)
Hypertension / renal vascular disease	89.2 (88.9-89.6)	79.1 (78.7-79.5)	48.4 (47.8-49.1)	89.4 (89.1-89.7)	79.3 (78.9-79.7)
Glomerulonephritis	92.0 (91.6-92.5)	84.2 (83.6-84.8)	57.0 (56.0-58.0)	92.3 (91.9-92.7)	84.8 (84.2-85.4)
Other causes	86.4 (86.1-86.6)	75.2 (74.8-75.5)	46.4 (45.9-46.8)	86.9 (86.7-87.2)	76.3 (75.9-76.6)
All	88.0 (87.9-88.2)	77.1 (76.9-77.4)	46.6 (46.3-46.9)	88.4 (88.2-88.5)	77.8 (77.6-78.0)

Based on data from Austria, Belgium (Dutch-speaking), Belgium (French-speaking), Bosnia and Herzegovina, Denmark, Estonia, France, Greece, Iceland, Netherlands, Norway, Spain (Andalusia), Spain (Aragon), Spain (Basque country), Spain (Canary Islands), Spain (Cantabria), Spain (Castile and León), Spain (Castile-La Mancha), Spain (Catalonia), Spain (Community of Madrid), Spain (Extremadura), Spain (Galicia), Spain (Murcia), Spain (Navarre), Spain (Valencian Region), Sweden, United Kingdom (England/Northern Ireland/Wales) and United Kingdom (Scotland)

Table B.6.9

First transplant patients (deceased donor), from day of transplant, unadjusted

by age, sex, and primary renal disease

	Patient survival probabilities as % (95% CI)				
	cohort 2014 - 2018			cohort 2017 - 2021	
	1 year	2 year	5 year	1 year	2 year
0-19	99.1 (98.3-99.5)	98.5 (97.6-99.1)	97.1 (95.9-98.0)	98.7 (97.8-99.2)	98.3 (97.4-98.9)
20-44	98.8 (98.6-99.0)	98.1 (97.8-98.4)	95.7 (95.3-96.1)	99.0 (98.7-99.2)	98.1 (97.8-98.4)
45-64	97.0 (96.8-97.3)	95.3 (95.0-95.6)	87.2 (86.8-87.7)	96.7 (96.5-97.0)	94.7 (94.4-95.0)
65-74	93.1 (92.6-93.6)	88.8 (88.1-89.4)	70.4 (69.5-71.4)	92.5 (91.9-93.0)	87.5 (86.8-88.2)
75+	90.7 (89.2-91.9)	83.4 (81.6-85.0)	57.5 (55.2-59.7)	89.1 (87.8-90.3)	80.3 (78.6-81.9)
Male	96.3 (96.1-96.6)	93.7 (93.4-94.0)	83.3 (82.9-83.8)	95.7 (95.5-96.0)	92.6 (92.3-93.0)
Female	96.3 (96.0-96.6)	94.6 (94.2-95.0)	86.2 (85.6-86.7)	96.1 (95.7-96.4)	93.7 (93.3-94.1)
Diabetes	94.5 (93.9-95.0)	90.8 (90.1-91.4)	75.7 (74.7-76.7)	93.3 (92.7-93.9)	88.7 (87.9-89.4)
Hypertension / renal vascular disease	95.6 (95.0-96.1)	92.0 (91.2-92.7)	79.1 (77.9-80.2)	94.9 (94.3-95.5)	90.9 (90.1-91.7)
Glomerulonephritis	97.4 (97.1-97.7)	95.9 (95.5-96.3)	88.7 (88.0-89.3)	97.3 (96.9-97.6)	95.4 (94.9-95.8)
Other causes	96.7 (96.5-97.0)	94.9 (94.6-95.2)	86.9 (86.4-87.3)	96.4 (96.2-96.7)	94.2 (93.9-94.5)
All	96.3 (96.1-96.5)	94.1 (93.8-94.3)	84.3 (84.0-84.7)	95.8 (95.6-96.0)	93.0 (92.8-93.3)

Based on data from Austria, Belgium (Dutch-speaking), Belgium (French-speaking), Bosnia and Herzegovina, Denmark, Estonia, France, Greece, Iceland, Netherlands, Norway, Spain (Andalusia), Spain (Aragon), Spain (Basque country), Spain (Canary Islands), Spain (Cantabria), Spain (Castile and León), Spain (Castile-La Mancha), Spain (Catalonia), Spain (Community of Madrid), Spain (Extremadura), Spain (Galicia), Spain (Murcia), Spain (Navarre), Spain (Valencian Region), Sweden, United Kingdom (England/Northern Ireland/Wales) and United Kingdom (Scotland)

Table B.6.10

First transplant patients (deceased donor), from day of transplant, adjusted

adjusted for age, sex, and primary renal disease

	Patient survival probabilities as % (95% CI)				
	cohort 2014 - 2018			cohort 2017 - 2021	
	1 year	2 year	5 year	1 year	2 year
0-19	99.0 (98.3-99.6)	98.4 (97.6-99.2)	96.8 (95.7-97.9)	98.6 (97.8-99.3)	98.1 (97.3-99.0)
20-44	98.8 (98.6-99.1)	98.2 (97.9-98.5)	95.8 (95.4-96.2)	99.0 (98.8-99.2)	98.2 (97.9-98.4)
45-64	97.2 (97.0-97.5)	95.6 (95.4-95.9)	88.0 (87.6-88.5)	97.0 (96.7-97.2)	95.1 (94.8-95.4)
65-74	93.8 (93.3-94.2)	89.8 (89.2-90.4)	72.7 (71.7-73.6)	93.2 (92.7-93.7)	88.7 (88.0-89.3)
75+	91.5 (90.3-92.7)	84.8 (83.3-86.4)	60.2 (58.0-62.4)	90.3 (89.1-91.4)	82.3 (80.8-83.8)
Male	98.2 (98.1-98.4)	97.0 (96.8-97.1)	91.3 (91.0-91.6)	98.1 (98.0-98.2)	96.7 (96.5-96.8)
Female	98.0 (97.9-98.2)	97.1 (96.9-97.3)	92.1 (91.7-92.5)	98.0 (97.8-98.2)	96.8 (96.5-97.0)
Diabetes	97.3 (97.0-97.5)	95.3 (95.0-95.7)	86.7 (86.0-87.4)	96.9 (96.6-97.2)	94.7 (94.3-95.1)
Hypertension / renal vascular disease	98.3 (98.0-98.5)	96.8 (96.4-97.1)	90.8 (90.2-91.4)	98.1 (97.8-98.3)	96.5 (96.1-96.8)
Glomerulonephritis	98.4 (98.2-98.7)	97.5 (97.2-97.8)	92.8 (92.3-93.3)	98.4 (98.2-98.6)	97.3 (97.1-97.6)
Other causes	98.1 (98.0-98.3)	97.1 (96.9-97.3)	92.1 (91.7-92.4)	98.1 (97.9-98.2)	96.9 (96.6-97.1)
All	98.2 (98.1-98.3)	97.0 (96.9-97.2)	91.6 (91.3-91.9)	98.1 (98.0-98.2)	96.7 (96.5-96.9)

Based on data from Austria, Belgium (Dutch-speaking), Belgium (French-speaking), Bosnia and Herzegovina, Denmark, Estonia, France, Greece, Iceland, Netherlands, Norway, Spain (Andalusia), Spain (Aragon), Spain (Basque country), Spain (Canary Islands), Spain (Cantabria), Spain (Castile and León), Spain (Castile-La Mancha), Spain (Catalonia), Spain (Community of Madrid), Spain (Extremadura), Spain (Galicia), Spain (Murcia), Spain (Navarre), Spain (Valencian Region), Sweden, United Kingdom (England/Northern Ireland/Wales) and United Kingdom (Scotland)

Table B.6.11

First transplant patients (living donor), from day of transplant, unadjusted

by age, sex, and primary renal disease

	Patient survival probabilities as % (95% CI)				
	cohort 2014 - 2018			cohort 2017 - 2021	
	1 year	2 year	5 year	1 year	2 year
0-19	99.2 (98.4-99.6)	99.0 (98.1-99.5)	98.3 (97.2-98.9)	99.4 (98.6-99.8)	99.0 (98.1-99.5)
20-44	99.6 (99.4-99.7)	99.3 (99.0-99.5)	98.4 (98.0-98.7)	99.7 (99.5-99.9)	99.4 (99.0-99.6)
45-64	98.8 (98.5-99.0)	97.9 (97.4-98.2)	93.3 (92.6-94.0)	98.7 (98.3-99.0)	97.6 (97.1-98.0)
65-74	97.0 (96.1-97.8)	94.6 (93.3-95.6)	83.4 (81.4-85.2)	97.2 (96.2-97.9)	95.1 (93.9-96.1)
75+	94.6 (89.0-97.4)	93.0 (87.0-96.3)	68.0 (59.1-75.3)	93.9 (89.0-96.7)	90.9 (85.4-94.4)
Male	98.6 (98.4-98.9)	97.8 (97.5-98.1)	93.8 (93.2-94.3)	98.6 (98.3-98.8)	97.6 (97.3-98.0)
Female	99.2 (98.9-99.4)	98.3 (97.9-98.6)	94.5 (93.7-95.1)	99.3 (98.9-99.5)	98.3 (97.9-98.7)
Diabetes	97.3 (96.1-98.2)	94.8 (93.3-96.0)	84.2 (81.8-86.4)	97.9 (96.7-98.7)	95.5 (93.9-96.7)
Hypertension / renal vascular disease	97.9 (96.9-98.7)	96.2 (94.8-97.2)	89.4 (87.3-91.2)	97.1 (95.8-98.0)	95.8 (94.3-96.9)
Glomerulonephritis	99.0 (98.6-99.3)	98.6 (98.1-99.0)	96.2 (95.4-96.8)	99.3 (98.9-99.6)	98.8 (98.3-99.1)
Other causes	99.1 (98.9-99.3)	98.5 (98.2-98.8)	95.2 (94.7-95.7)	99.0 (98.7-99.2)	98.1 (97.8-98.4)
All	98.8 (98.6-99.0)	98.0 (97.8-98.2)	94.0 (93.6-94.4)	98.8 (98.6-99.0)	97.9 (97.6-98.1)

Based on data from Austria, Belgium (Dutch-speaking), Belgium (French-speaking), Bosnia and Herzegovina, Denmark, Estonia, France, Greece, Iceland, Netherlands, Norway, Spain (Andalusia), Spain (Aragon), Spain (Basque country), Spain (Canary Islands), Spain (Cantabria), Spain (Castile and León), Spain (Castile-La Mancha), Spain (Catalonia), Spain (Community of Madrid), Spain (Extremadura), Spain (Galicia), Spain (Murcia), Spain (Navarre), Spain (Valencian Region), Sweden, United Kingdom (England/Northern Ireland/Wales) and United Kingdom (Scotland)

Table B.6.12

First transplant patients (living donor), from day of transplant, adjusted

adjusted for age, sex, and primary renal disease

	Patient survival probabilities as % (95% CI)				
	cohort 2014 - 2018			cohort 2017 - 2021	
	1 year	2 year	5 year	1 year	2 year
0-19	99.2 (98.6-99.8)	99.0 (98.2-99.7)	98.0 (97.0-99.0)	99.3 (98.7-99.9)	98.9 (98.2-99.7)
20-44	99.6 (99.4-99.8)	99.3 (99.0-99.5)	98.3 (97.9-98.7)	99.7 (99.5-99.9)	99.3 (99.0-99.6)
45-64	98.8 (98.5-99.1)	97.9 (97.6-98.3)	93.6 (92.9-94.2)	98.7 (98.4-99.0)	97.6 (97.2-98.1)
65-74	97.4 (96.6-98.1)	95.1 (94.1-96.2)	84.9 (83.2-86.7)	97.5 (96.8-98.3)	95.7 (94.7-96.7)
75+	95.2 (91.8-98.7)	93.9 (90.0-97.8)	70.6 (63.4-78.7)	94.5 (91.3-97.9)	91.8 (87.8-95.9)
Male	99.0 (98.8-99.2)	98.4 (98.2-98.7)	95.3 (94.8-95.7)	98.9 (98.7-99.2)	98.2 (97.9-98.5)
Female	99.3 (99.1-99.6)	98.6 (98.2-98.9)	95.1 (94.5-95.8)	99.4 (99.1-99.6)	98.6 (98.3-98.9)
Diabetes	98.4 (97.8-99.0)	96.9 (96.1-97.7)	90.1 (88.5-91.6)	98.8 (98.3-99.4)	97.4 (96.6-98.3)
Hypertension / renal vascular disease	98.9 (98.4-99.4)	97.9 (97.2-98.6)	94.0 (92.8-95.2)	98.5 (97.9-99.1)	97.8 (97.1-98.5)
Glomerulonephritis	99.1 (98.7-99.4)	98.6 (98.2-99.0)	96.1 (95.4-96.8)	99.3 (99.1-99.6)	98.8 (98.4-99.2)
Other causes	99.2 (99.1-99.4)	98.7 (98.5-99.0)	95.8 (95.3-96.3)	99.1 (98.9-99.3)	98.4 (98.1-98.7)
All	99.1 (99.0-99.3)	98.5 (98.3-98.7)	95.2 (94.8-95.6)	99.1 (98.9-99.3)	98.4 (98.1-98.6)

Based on data from Austria, Belgium (Dutch-speaking), Belgium (French-speaking), Bosnia and Herzegovina, Denmark, Estonia, France, Greece, Iceland, Netherlands, Norway, Spain (Andalusia), Spain (Aragon), Spain (Basque country), Spain (Canary Islands), Spain (Cantabria), Spain (Castile and León), Spain (Castile-La Mancha), Spain (Catalonia), Spain (Community of Madrid), Spain (Extremadura), Spain (Galicia), Spain (Murcia), Spain (Navarre), Spain (Valencian Region), Sweden, United Kingdom (England/Northern Ireland/Wales) and United Kingdom (Scotland)

Table B.6.13

First graft (deceased donor), from day of transplant, unadjusted

by age, sex, and primary renal disease

	Graft survival probabilities as % (95% CI)				
	cohort 2014 - 2018			cohort 2017 - 2021	
	1 year	2 year	5 year	1 year	2 year
0-19	94.8 (93.2-95.9)	92.3 (90.6-93.8)	84.9 (82.6-86.9)	94.4 (92.8-95.6)	91.8 (90.0-93.3)
20-44	94.5 (94.0-94.9)	92.5 (92.0-93.0)	85.2 (84.5-85.9)	95.3 (94.9-95.7)	93.2 (92.7-93.7)
45-64	92.2 (91.8-92.5)	89.3 (88.9-89.8)	78.8 (78.3-79.4)	92.1 (91.7-92.5)	89.3 (88.8-89.7)
65-74	86.5 (85.7-87.2)	81.3 (80.5-82.1)	62.0 (61.0-63.0)	86.4 (85.7-87.0)	80.9 (80.1-81.7)
75+	83.1 (81.3-84.7)	75.1 (73.1-77.0)	50.1 (47.8-52.4)	81.7 (80.0-83.2)	72.2 (70.3-73.9)
Male	90.9 (90.6-91.3)	87.4 (86.9-87.7)	74.5 (73.9-75.0)	90.6 (90.2-90.9)	86.7 (86.3-87.1)
Female	91.4 (90.9-91.8)	88.4 (87.9-88.9)	77.1 (76.4-77.7)	91.6 (91.1-92.0)	88.2 (87.6-88.7)
Diabetes	89.5 (88.8-90.2)	85.1 (84.3-85.9)	68.6 (67.5-69.7)	88.1 (87.4-88.9)	82.9 (82.0-83.7)
Hypertension / renal vascular disease	89.4 (88.5-90.2)	84.7 (83.6-85.6)	69.0 (67.7-70.3)	89.6 (88.7-90.4)	84.6 (83.6-85.6)
Glomerulonephritis	91.8 (91.2-92.3)	89.0 (88.3-89.6)	78.3 (77.4-79.2)	92.1 (91.5-92.7)	89.2 (88.6-89.9)
Other causes	91.8 (91.4-92.1)	88.9 (88.5-89.3)	78.2 (77.6-78.7)	91.8 (91.4-92.2)	88.7 (88.3-89.1)
All	91.1 (90.8-91.4)	87.7 (87.4-88.0)	75.4 (75.0-75.8)	90.9 (90.6-91.2)	87.2 (86.9-87.6)

Based on data from Austria, Belgium (Dutch-speaking), Belgium (French-speaking), Bosnia and Herzegovina, Denmark, Estonia, France, Greece, Iceland, Netherlands, Norway, Spain (Andalusia), Spain (Aragon), Spain (Basque country), Spain (Canary Islands), Spain (Cantabria), Spain (Castile and León), Spain (Castile-La Mancha), Spain (Catalonia), Spain (Community of Madrid), Spain (Extremadura), Spain (Galicia), Spain (Murcia), Spain (Navarre), Spain (Valencian Region), Sweden, United Kingdom (England/Northern Ireland/Wales) and United Kingdom (Scotland)

Table B.6.14

First graft (deceased donor), from day of transplant, adjusted

adjusted for age, sex, and primary renal disease

	Graft survival probabilities as % (95% CI)				
	cohort 2014 - 2018			cohort 2017 - 2021	
	1 year	2 year	5 year	1 year	2 year
0-19	94.3 (92.9-95.7)	91.7 (90.0-93.4)	83.6 (81.4-86.0)	93.9 (92.4-95.4)	91.1 (89.4-92.9)
20-44	94.5 (94.1-95.0)	92.6 (92.1-93.1)	85.3 (84.6-86.1)	95.4 (94.9-95.8)	93.3 (92.7-93.8)
45-64	92.4 (92.1-92.8)	89.7 (89.3-90.1)	79.5 (78.9-80.0)	92.4 (92.0-92.8)	89.7 (89.2-90.1)
65-74	87.2 (86.5-87.9)	82.3 (81.6-83.1)	63.6 (62.6-64.6)	87.1 (86.5-87.8)	81.9 (81.1-82.6)
75+	84.0 (82.4-85.7)	76.4 (74.5-78.3)	52.0 (49.7-54.3)	82.7 (81.3-84.3)	73.7 (71.9-75.5)
Male	93.3 (93.0-93.6)	90.6 (90.2-90.9)	80.2 (79.7-80.7)	93.3 (93.0-93.5)	90.4 (90.1-90.8)
Female	93.2 (92.8-93.6)	90.8 (90.3-91.2)	81.2 (80.6-81.8)	93.5 (93.2-93.9)	90.8 (90.4-91.3)
Diabetes	92.2 (91.7-92.8)	88.8 (88.2-89.5)	75.3 (74.4-76.3)	91.6 (91.0-92.1)	87.6 (86.9-88.3)
Hypertension / renal vascular disease	93.0 (92.4-93.6)	89.7 (89.0-90.4)	78.2 (77.2-79.2)	93.3 (92.7-93.9)	90.0 (89.2-90.7)
Glomerulonephritis	93.1 (92.6-93.6)	90.7 (90.1-91.3)	81.3 (80.5-82.1)	93.6 (93.1-94.0)	91.2 (90.6-91.7)
Other causes	93.3 (93.0-93.6)	90.9 (90.5-91.3)	81.7 (81.1-82.2)	93.5 (93.2-93.8)	91.0 (90.6-91.4)
All	93.3 (93.0-93.5)	90.6 (90.4-90.9)	80.6 (80.2-81.0)	93.4 (93.1-93.6)	90.6 (90.3-90.9)

Based on data from Austria, Belgium (Dutch-speaking), Belgium (French-speaking), Bosnia and Herzegovina, Denmark, Estonia, France, Greece, Iceland, Netherlands, Norway, Spain (Andalusia), Spain (Aragon), Spain (Basque country), Spain (Canary Islands), Spain (Cantabria), Spain (Castile and León), Spain (Castile-La Mancha), Spain (Catalonia), Spain (Community of Madrid), Spain (Extremadura), Spain (Galicia), Spain (Murcia), Spain (Navarre), Spain (Valencian Region), Sweden, United Kingdom (England/Northern Ireland/Wales) and United Kingdom (Scotland)

Table B.6.15

First graft (living donor), from day of transplant, unadjusted

by age, sex, and primary renal disease

	Graft survival probabilities as % (95% CI)				
	cohort 2014 - 2018			cohort 2017 - 2021	
	1 year	2 year	5 year	1 year	2 year
0-19	96.0 (94.5-97.1)	94.2 (92.4-95.5)	89.3 (87.1-91.2)	97.5 (96.2-98.4)	95.7 (94.1-96.9)
20-44	96.8 (96.3-97.3)	95.7 (95.1-96.3)	90.0 (89.0-90.8)	97.6 (97.0-98.0)	96.3 (95.6-96.8)
45-64	96.7 (96.2-97.1)	95.2 (94.6-95.7)	88.8 (88.0-89.7)	96.8 (96.3-97.3)	95.2 (94.6-95.8)
65-74	95.5 (94.3-96.4)	92.7 (91.3-93.9)	80.2 (78.1-82.1)	95.8 (94.7-96.7)	93.2 (91.8-94.3)
75+	94.6 (89.0-97.4)	92.2 (86.1-95.8)	66.4 (57.5-73.9)	93.3 (88.3-96.3)	90.3 (84.7-93.9)
Male	96.4 (96.0-96.8)	95.0 (94.5-95.5)	88.3 (87.6-89.0)	96.9 (96.4-97.2)	95.3 (94.8-95.8)
Female	96.7 (96.2-97.2)	94.9 (94.2-95.5)	87.4 (86.4-88.4)	97.1 (96.5-97.6)	95.2 (94.5-95.8)
Diabetes	94.9 (93.3-96.1)	91.5 (89.6-93.0)	79.6 (76.9-81.9)	96.1 (94.6-97.2)	93.0 (91.1-94.5)
Hypertension / renal vascular disease	96.0 (94.6-97.0)	93.1 (91.4-94.5)	82.9 (80.4-85.1)	95.4 (93.8-96.5)	92.9 (91.0-94.4)
Glomerulonephritis	96.6 (95.9-97.2)	95.5 (94.7-96.2)	88.5 (87.3-89.6)	97.2 (96.5-97.7)	95.8 (95.0-96.5)
Other causes	96.8 (96.4-97.2)	95.5 (95.0-96.0)	89.8 (89.0-90.4)	97.1 (96.7-97.5)	95.7 (95.1-96.1)
All	96.5 (96.2-96.8)	95.0 (94.6-95.3)	88.0 (87.4-88.5)	96.9 (96.6-97.2)	95.3 (94.9-95.7)

Based on data from Austria, Belgium (Dutch-speaking), Belgium (French-speaking), Bosnia and Herzegovina, Denmark, Estonia, France, Greece, Iceland, Netherlands, Norway, Spain (Andalusia), Spain (Aragon), Spain (Basque country), Spain (Canary Islands), Spain (Cantabria), Spain (Castile and León), Spain (Castile-La Mancha), Spain (Catalonia), Spain (Community of Madrid), Spain (Extremadura), Spain (Galicia), Spain (Murcia), Spain (Navarre), Spain (Valencian Region), Sweden, United Kingdom (England/Northern Ireland/Wales) and United Kingdom (Scotland)

Table B.6.16

First graft (living donor), from day of transplant, adjusted

adjusted for age, sex, and primary renal disease

	Graft survival probabilities as % (95% CI)				
	cohort 2014 - 2018			cohort 2017 - 2021	
	1 year	2 year	5 year	1 year	2 year
0-19	95.4 (94.0-96.9)	93.2 (91.5-95.0)	87.7 (85.5-90.1)	97.1 (95.9-98.3)	95.0 (93.5-96.6)
20-44	96.7 (96.2-97.3)	95.6 (95.0-96.2)	89.5 (88.6-90.4)	97.4 (96.9-97.9)	96.0 (95.4-96.7)
45-64	96.7 (96.2-97.2)	95.2 (94.6-95.8)	88.9 (88.1-89.7)	96.8 (96.3-97.3)	95.2 (94.5-95.8)
65-74	95.8 (94.8-96.8)	93.1 (91.8-94.4)	81.1 (79.1-83.1)	96.1 (95.1-97.0)	93.6 (92.3-94.8)
75+	94.9 (91.3-98.7)	92.8 (88.5-97.2)	67.7 (60.1-76.2)	93.5 (89.8-97.3)	90.5 (86.2-95.1)
Male	96.4 (96.0-96.8)	95.0 (94.5-95.5)	88.1 (87.4-88.8)	96.8 (96.4-97.2)	95.3 (94.8-95.8)
Female	96.5 (96.0-97.1)	94.5 (93.8-95.2)	86.6 (85.5-87.6)	96.9 (96.3-97.4)	94.8 (94.1-95.5)
Diabetes	95.3 (94.0-96.5)	92.1 (90.5-93.7)	80.9 (78.6-83.3)	96.5 (95.3-97.7)	93.7 (92.1-95.2)
Hypertension / renal vascular disease	96.3 (95.2-97.4)	93.7 (92.3-95.1)	84.1 (82.0-86.4)	95.9 (94.7-97.1)	93.7 (92.2-95.2)
Glomerulonephritis	96.4 (95.7-97.0)	95.1 (94.3-95.9)	87.6 (86.4-88.8)	97.0 (96.3-97.7)	95.5 (94.7-96.3)
Other causes	96.7 (96.3-97.1)	95.4 (94.9-95.9)	89.4 (88.6-90.1)	97.0 (96.6-97.4)	95.5 (95.0-96.0)
All	96.4 (96.1-96.8)	94.8 (94.4-95.2)	87.6 (87.0-88.2)	96.8 (96.5-97.2)	95.1 (94.7-95.5)

Based on data from Austria, Belgium (Dutch-speaking), Belgium (French-speaking), Bosnia and Herzegovina, Denmark, Estonia, France, Greece, Iceland, Netherlands, Norway, Spain (Andalusia), Spain (Aragon), Spain (Basque country), Spain (Canary Islands), Spain (Cantabria), Spain (Castile and León), Spain (Castile-La Mancha), Spain (Catalonia), Spain (Community of Madrid), Spain (Extremadura), Spain (Galicia), Spain (Murcia), Spain (Navarre), Spain (Valencian Region), Sweden, United Kingdom (England/Northern Ireland/Wales) and United Kingdom (Scotland)

Table B.7.1

Expected remaining years of life in the general population and in prevalent dialysis and kidney transplant patients (cohort 2019-2023)

by age and sex

General population				Dialysis				Transplant			
Age	All	Male	Female	Age	All	Male	Female	Age	All	Male	Female
20-24	62.2	59.9	64.4	20-24	20.9	21.3	20.4	20-24	41.6	41.5	41.8
25-29	57.3	55.0	59.5	25-29	18.1	18.3	17.8	25-29	37.3	37.1	37.6
30-34	52.4	50.2	54.6	30-34	15.9	15.9	15.9	30-34	33.1	32.7	33.7
35-39	47.5	45.3	49.7	35-39	14.0	14.0	14.1	35-39	29.0	28.6	29.6
40-44	42.7	40.6	44.8	40-44	12.0	12.0	12.1	40-44	25.0	24.5	25.7
45-49	37.9	35.8	40.0	45-49	10.2	10.1	10.4	45-49	21.2	20.8	21.9
50-54	33.3	31.2	35.2	50-54	8.6	8.4	8.9	50-54	17.6	17.2	18.4
55-59	28.7	26.8	30.6	55-59	7.3	7.0	7.7	55-59	14.5	14.0	15.2
60-64	24.4	22.5	26.1	60-64	6.2	6.1	6.6	60-64	11.6	11.3	12.3
65-69	20.2	18.6	21.7	65-69	5.4	5.2	5.7	65-69	9.2	8.9	9.8
70-74	16.3	14.8	17.5	70-74	4.7	4.6	5.0	70-74	7.2	7.0	7.6
75-79	12.6	11.4	13.6	75-79	4.0	3.9	4.2	75-79	5.5	5.4	5.7
80-84	9.3	8.3	10.0	80-84	3.3	3.3	3.5	80-84	3.8	3.7	3.9
85-89	6.4	5.8	6.9	85-89	2.2	2.1	2.2	85-89	2.3	2.4	2.1
90-94	4.3	3.9	4.5	90-94	1.6	1.5	1.7				

Based on data from Austria, Belgium (Dutch-speaking), Belgium (French-speaking), Bosnia and Herzegovina, Denmark, Estonia, France, Greece, Iceland, Netherlands, Norway, Spain (Andalusia), Spain (Aragon), Spain (Basque country), Spain (Canary Islands), Spain (Cantabria), Spain (Castile and León), Spain (Castile-La Mancha), Spain (Catalonia), Spain (Community of Madrid), Spain (Extremadura), Spain (Galicia), Spain (Murcia), Spain (Navarre), Spain (Valencian Region), Sweden, United Kingdom (England/Northern Ireland/Wales) and United Kingdom (Scotland)

Section C

Aggregated data reference tables

Table C.1.1

General population data and number of renal centres

	General population of country/region in thousands	% Coverage of general population by the registry	Total number of renal centres in country/region	Number of renal centres collaborating with the registry
Albania	2762	99.0	14	14
Belarus ‡	9178	92.0	54	50
Croatia	3860	85.0	46	43
Cyprus	949	100	11	11
Czech Republic	10828	100	114	114
Finland	5604	98.0	30	30
Hungary	9600	100	68	68
Ireland	5149	100	25	25
Israel	9633	100	79	79
Italy (10 of 20 regions) §	58997	48.5	780	320
Italy (5 extra regions) # &	17602	18.5	208	208
Italy (15 of 20 regions) ○ &	58997	67.0	780	528
Kosovo	1740	97.0	9	8
Latvia	1883	92.0	33	28
Lithuania	2857	100	66	66
North Macedonia	1826	100	23	23
Poland	37637	100	278	278
Portugal	10640	100	139	139
Slovakia	5427	82.4	74	61
Spain	48085	100	361	357
Türkiye	85372	100	1143	1143

When cells are left empty, the data are unavailable
* Patients younger than 18 years of age are not reported
† Data include dialysis patients only
○ Preemptive transplantations are not reported for Lazio
§ Data presented in column 0-19 refer to patients aged 0-14 years and data presented in column 20-44 refer to patients aged 15-44 years
# Including 255 patients (3.4%) with unknown sex and 243 patients (3.3%) with unknown age

Table C.2.1

Incident counts and percentages by age and sex

at day 1

	All			0-19						20-44						45-64						65-74						75+					
	All	Male	Female	All		Male		Female		All		Male		Female		All		Male		Female		All		Male		Female		All		Male		Female	
	N (100%)	N (100%)	N (100%)	N	%	N	%	N	%	N	%	N	%	N	%	N	%	N	%	N	%	N	%	N	%	N	%	N	%	N	%	N	%
Albania	394	260	134	1	0	1	0	0	0	26	7	21	8	5	4	167	42	116	45	51	38	124	31	74	28	50	37	76	19	48	18	28	21
Belarus *	883	507	376	2	0	1	0	1	0	161	18	96	19	65	17	369	42	231	46	138	37	257	29	134	26	123	33	94	11	45	9	49	13
Croatia †	484	294	190	0	0	0	0	0	0	20	4	11	4	9	5	91	19	47	16	44	23	128	26	86	29	42	22	245	51	150	51	95	50
Cyprus	255	178	77	2	1	2	1	0	0	28	11	20	11	8	10	61	24	36	20	25	32	60	24	45	25	15	19	104	41	75	42	29	38
Czech Republic	1744	1139	605	5	0	4	0	1	0	152	9	96	8	56	9	486	28	358	31	128	21	552	32	357	31	195	32	549	31	324	28	225	37
Finland	458	311	147	9	2	5	2	4	3	67	15	48	15	19	13	162	35	105	34	57	39	114	25	77	25	37	25	106	23	76	24	30	20
Hungary	1321	727	594	14	1	9	1	5	1	128	10	75	10	53	9	429	32	265	36	164	28	412	31	224	31	188	32	338	26	154	21	184	31
Ireland																																	
Israel	1807	1159	648	30	2	19	2	11	2	158	9	91	8	67	10	498	28	338	29	160	25	530	29	329	28	201	31	591	33	382	33	209	32
Italy (10 of 20 regions)	4860	3209	1651	24	0	16	0	8	0	321	7	207	6	114	7	1206	25	791	25	415	25	1261	26	839	26	422	26	2048	42	1356	42	692	42
Italy (5 extra regions) ¤	3065	2068	997	29	1					239	8					862	28					767	25					1168	38				
Italy (15 of 20 regions) ¤	7925	5277	2648	53	1					560	7					2068	26					2028	26					3216	41				
Kosovo *	225	113	112	1	0	1	1	0	0	17	8	10	9	7	6	73	32	45	40	28	25	82	36	36	32	46	41	52	23	21	19	31	28
Latvia	185	100	85	1	1	0	0	1	1	24	13	12	12	12	14	73	39	43	43	30	35	54	29	30	30	24	28	33	18	15	15	18	21
Lithuania	368	224	144	5	1	3	1	2	1	44	12	26	12	18	13	133	36	86	38	47	33	83	23	50	22	33	23	103	28	59	26	44	31
North Macedonia	423	241	182	3	1	3	1	0	0	28	7	18	7	10	5	148	35	81	34	67	37	134	32	84	35	50	27	110	26	55	23	55	30
Poland †	5655																					1984	35					1558	28				
Portugal	2521																																
Slovakia †	794	477	317	4	1	1	0	3	1	76	10	45	9	31	10	296	37	177	37	119	38	265	33	164	34	101	32	153	19	90	19	63	20
Spain § #	7287	4814	2218	65	1	42	1	23	1	699	10	451	9	244	11	2117	29	1406	29	708	32	1927	26	1358	28	568	26	2236	31	1557	32	675	30
Türkiye	13641	7790	5153	378	3	197	3	181	4	2064	15	1336	17	728	14	4825	35	3100	40	1725	33	3256	24	1883	24	1373	27	2420	18	1274	16	1146	22

Categories may not add up due to missing values or rounding
When cells are left empty, the data are unavailable
* Patients younger than 18 years of age are not reported
† Data include dialysis patients only
¤ Preemptive transplantations are not reported for Lazio
§ Data presented in column 0-19 refer to patients aged 0-14 years and data presented in column 20-44 refer to patients aged 15-44 years
# Including 255 patients (3.4%) with unknown sex and 243 patients (3.3%) with unknown age

Table C.2.2

Incidence per million (age-related) population by age and sex, unadjusted

at day 1

	All			0-19			20-44			45-64			65-74			75+		
	All	Male	Female	All	Male	Female	All	Male	Female	All	Male	Female	All	Male	Female	All	Male	Female
	Pmp	Pmp	Pmp	Pmarp	Pmarp	Pmarp	Pmarp	Pmarp	Pmarp	Pmarp	Pmarp	Pmarp	Pmarp	Pmarp	Pmarp	Pmarp	Pmarp	Pmarp
Albania	144.1	192.1	97.0	1.6	3.2	0	27.2	43.6	10.5	232.5	333.6	137.6	446.7	557.9	345.0	440.9	587.5	308.8
Belarus *	104.6	130.0	82.7	1.1	1.1	1.1	56.7	67.9	45.5	157.6	216.7	108.2	279.1	383.8	215.1	187.7	351.9	131.4
Croatia †	147.5	185.0	112.3	0	0	0	20.1	21.6	18.6	100.2	106.2	94.4	292.2	433.5	175.2	784.5	1304.7	481.4
Cyprus	268.7	382.7	159.1	10.3	20.1	0	79.4	113.7	45.3	255.4	320.3	197.7	654.7	1011.5	318.1	1428.2	2293.8	722.8
Czech Republic	161.1	214.5	109.6	2.2	3.4	0.9	45.4	56.2	34.2	164.0	240.4	86.8	436.0	619.6	282.7	582.8	917.6	382.1
Finland	83.4	114.4	53.0	8.0	8.7	7.3	38.7	53.7	22.7	119.6	154.3	84.6	170.4	242.4	105.3	172.6	301.9	82.8
Hungary	137.6	157.2	119.4	7.4	9.3	5.5	42.0	47.9	35.8	158.8	198.7	119.9	353.0	454.8	278.6	420.2	576.9	342.4
Ireland																		
Israel	187.6	242.5	133.5	8.9	11.0	6.7	50.3	57.6	42.9	266.7	371.0	167.3	751.6	1004.0	532.5	1043.2	1589.0	640.9
Italy (10 of 20 regions)	169.8	229.6	112.8	4.8	6.3	3.3	40.6	51.3	29.4	136.1	181.6	92.1	376.1	529.5	238.6	581.0	941.8	331.9
Italy (5 extra regions) ¤	174.1	240.1	110.9	9.3			48.7			156.8			387.3			552.9		
Italy (15 of 20 regions) ¤	172.2	234.7	112.5	6.6			44.2			144.9			376.8			561.7		
Kosovo *	133.3	133.0	133.7	1.6	3.0	0	26.3	30.9	21.7	255.8	319.9	193.5	1085.7	985.5	1179.6	1377.3	1278.6	1453.3
Latvia	106.8	124.6	91.4	2.7	0	5.6	45.8	44.7	46.9	152.6	192.7	117.6	276.2	396.3	200.3	196.7	322.7	148.4
Lithuania	128.8	167.7	94.6	8.9	10.4	7.3	49.0	56.6	41.1	160.8	221.4	107.1	268.3	406.1	177.3	392.6	759.7	238.2
North Macedonia	231.6	266.4	197.5	7.4	14.3	0	47.4	60.0	34.5	296.7	330.3	264.2	637.4	849.6	449.0	909.7	1069.8	791.2
Poland †	150.3												428.6			533.3		
Portugal	236.9																	
Slovakia †	177.6	218.1	138.8	4.3	2.1	6.6	50.9	58.8	42.7	240.1	288.4	192.3	521.3	733.3	354.8	507.9	867.6	318.9
Spain § #	151.5	204.3	90.5	7.1	9.0	5.2	47.5	60.8	33.5	145.0	194.1	96.3	393.0	590.0	218.4	467.3	814.4	235.0
Türkiye	159.8	182.3	120.9	15.3	15.5	15.0	64.1	81.9	45.9	244.2	314.2	174.3	582.9	715.5	464.8	771.4	1020.4	606.8

When cells are left empty, the data are unavailable
* Patients younger than 18 years of age are not reported
† Data include dialysis patients only
¤ Preemptive transplantations are not reported for Lazio
§ Data presented in column 0-19 refer to patients aged 0-14 years and data presented in column 20-44 refer to patients aged 15-44 years
# Including 255 patients (3.4%) with unknown sex and 243 patients (3.3%) with unknown age

Table C.2.3

Sex, mean age, and median age of incident patients
at day 1

	All						Male						Female					
	%	Mean (years)	SD	Median (years)	P25	P75	%	Mean (years)	SD	Median (years)	P25	P75	%	Mean (years)	SD	Median (years)	P25	P75
Albania	100	61.6	15.3	65.0	54.0	72.0	66.0	62.3	12.3	65.0	54.0	71.0	34.0	60.2	16.4	64.0	57.0	73.0
Belarus *	100						57.4						42.6					
Croatia †	100	67.2	13.5	70.0	60.0	78.0	60.7	67.5	12.4	70.0	60.0	78.0	39.3	66.9	15.0	70.5	64.0	79.0
Cyprus	100	67.3	16.0	71.0	59.5	79.0	69.8	67.6	16.1	72.0	61.0	79.0	30.2	66.6	15.8	70.0	58.0	78.0
Czech Republic	100	66.0	14.1	69.0	58.0	76.0	65.3	65.3	13.8	68.0	58.0	75.0	34.7	67.4	14.6	71.0	61.0	77.0
Finland	100	62.0	16.5	65.8	54.1	75.5	67.9	62.5	16.2	67.1	55.4	75.4	32.1	60.8	17.2	64.1	50.2	74.7
Hungary	100	63.8	15.2	67.0	55.0	75.0	55.0	62.4	14.8	65.0	53.0	73.0	45.0	65.6	15.5	69.0	56.3	76.0
Ireland																		
Israel	100	66.2	16.3	69.5	58.2	77.4	64.1	66.3	15.9	69.6	58.9	77.3	35.9	65.9	17.0	69.2	57.4	77.9
Italy (10 of 20 regions)	100	67.4	14.5	71.3	58.7	77.0	66.0	67.5	14.4	71.3	58.9	77.0	34.0	67.3	14.6	71.2	58.3	77.0
Italy (5 extra regions)																		
Italy (15 of 20 regions)																		
Kosovo *	100	65.5	12.6	68.0	57.0	70.0	50.2	63.3	13.2	65.0	54.0	70.0	49.8	67.7	11.7	70.0	60.0	73.0
Latvia	100	61.7	15.3	63.0	53.0	72.0	54.1	62.2	14.5	62.0	54.8	71.3	45.9	61.2	16.3	64.0	51.0	74.0
Lithuania	100	63.6	16.6	65.2	54.1	76.7	60.9	63.1	16.3	64.8	52.6	75.9	39.1	64.4	17.3	66.0	55.1	78.0
North Macedonia	100	65.3	13.0	67.0	58.8	75.0	57.0	64.9	13.1	67.0	59.0	74.0	43.0	65.9	12.9	68.5	57.3	75.0
Poland																		
Portugal																		
Slovakia †	100	62.9	14.2	66.0	54.0	73.0	60.1	62.6	14.2	66.0	53.0	73.0	39.9	63.4	14.2	65.0	56.0	73.0
Spain #	100	63.5	15.5	68.3	54.4	77.4	66.1	63.9	15.3	68.8	55.1	77.6	30.4	62.6	15.9	67.4	53.1	77.1
Türkiye	100						57.1						37.8					

Categories may not add up due to missing values or rounding
When cells are left empty, the data are unavailable
* Patients younger than 18 years of age are not reported
† Data include dialysis patients only
# Sex was unknown for 3.4% and age was unknown for 3.3% of incident patients

Table C.2.4
Incidence per million population, adjusted
at day 1, adjusted for age and sex

	All	Male	Female
	Pmp	Pmp	Pmp
Albania	157.9	208.0	110.0
Belarus			
Croatia †	148.5	189.3	109.5
Cyprus	304.3	422.4	191.4
Czech Republic	168.7	228.1	111.9
Finland	81.3	110.7	53.2
Hungary	143.3	169.4	118.3
Ireland			
Israel	285.1	371.0	203.0
Italy (10 of 20 regions)	150.3	200.7	102.2
Italy (5 extra regions)			
Italy (15 of 20 regions)			
Kosovo *	360.9	336.3	384.4
Latvia	111.9	136.9	88.0
Lithuania	137.4	185.9	91.0
North Macedonia	254.9	287.3	223.9
Poland			
Portugal			
Slovakia †	195.7	247.9	145.8
Spain			
Türkiye			

When cells are left empty, the data are unavailable
* Patients younger than 18 years of age are not reported
† Data include dialysis patients only

FIND

Table C.2.5

Incidence per million population and percentages by primary renal disease (1995 PRD codes), unadjusted

at day 1

	All		GN		PN		PKD		DM						HT		RVD		Misc		Unkn		Missing	
									Type 1		Type 2		Total											
	Pmp	%	Pmp	%	Pmp	%	Pmp	%	Pmp	%	Pmp	%	Pmp	%	Pmp	%	Pmp	%	Pmp	%	Pmp	%	Pmp	%
Albania	144.1	100	29.6	20.6	18.3	12.7	9.1	6.3	3.3	2.3	31.5	21.8	34.7	24.1	25.6	17.8	2.6	1.8	12.8	8.9	9.5	6.6	1.8	1.3
Belarus *	104.6	100	20.4	19.5	9.8	9.4	7.5	7.1	5.6	5.3	17.9	17.1	23.4	22.4	13.3	12.7	8.1	7.7	21.8	20.8	0.4	0.3	0	0
Croatia †	147.5	100	13.1	8.9	1.8	1.2	12.8	8.7	2.7	1.9	33.5	22.7	36.3	24.6	36.3	24.6	3.4	2.3	30.5	20.7	8.2	5.6	5.2	3.5
Cyprus	268.7	100	15.8	5.9	0	0	11.6	4.3					95.9	35.7	49.5	18.4	8.4	3.1	48.5	18.0	39.0	14.5	0	0
Czech Republic $ ||	161.1	100	15.9	9.9	3.8	2.4	7.6	4.7					33.5	20.8			24.1	15.0	24.2	15.0	48.4	30.0	3.6	2.2
Finland	83.4	100	8.2	9.8	1.5	1.7	9.1	10.9	13.3	15.9	15.5	18.6	28.8	34.5	6.2	7.4	0.4	0.4	10.4	12.4	15.5	18.6	3.5	4.1
Hungary	137.6	100	10.2	7.4	17.5	12.7	9.2	6.7	2.7	2.0	48.4	35.2	51.1	37.2	19.3	14.0	0.8	0.6	1.8	1.3	3.3	2.4	24.4	17.7
Ireland																								
Israel	187.6	100	7.5	4.0	2.8	1.5	4.5	2.4	4.3	2.3	68.1	36.3	72.4	38.6	17.9	9.5	1.2	0.7	17.1	9.1	52.5	28.0	11.7	6.3
Italy (10 of 20 regions)	169.8	100	15.0	8.8	5.4	3.2	7.2	4.2					19.5	11.5	17.2	10.1	4.5	2.7	0.7	0.4	86.3	50.8	14.1	8.3
Italy (5 extra regions) ¤	174.1	100	4.0	2.3	1.6	0.9	4.0	2.3					12.3	7.0	0	0	8.0	4.6	10.5	6.0	14.1	8.1	119.8	68.8
Italy (15 of 20 regions) ¤	172.2	100	10.9	6.3	4.0	2.3	6.0	3.5					16.8	9.8	10.7	6.2	5.9	3.4	4.4	2.6	59.0	34.3	54.6	31.7
Kosovo *	133.3	100	11.3	8.4	34.4	25.8	6.5	4.9	1.2	0.9	40.3	30.2	41.5	31.1	19.0	14.2	4.7	3.6	11.3	8.4	4.7	3.6	0	0
Latvia	106.8	100	19.0	17.8	9.2	8.6	11.5	10.8	5.2	4.9	14.4	13.5	19.6	18.4	13.9	13.0	1.2	1.1	31.2	29.2	1.2	1.1	0	0
Lithuania	128.8	100	17.1	13.3	14.7	11.4	10.8	8.4					21.0	16.3	28.0	21.7	4.9	3.8	22.4	17.4	9.8	7.6	0	0
North Macedonia	231.6	100	13.1	5.7	8.2	3.5	14.2	6.1	0	0	59.7	25.8	59.7	25.8	84.3	36.4	0.5	0.2	33.4	14.4	18.1	7.8	0	0
Poland																								
Portugal ‡ + &	236.9	100	27.6	11.7			14.1	6.0					72.2	30.5	25.8	10.9			52.0	21.9	38.5	16.3	3.7	1.5
Slovakia †	177.6	100	21.0	11.8	14.8	8.3	3.8	2.1					52.6	29.6	13.4	7.6	10.3	5.8	60.4	34.0	1.3	0.8	0	0
Spain ¶ §	151.5	100	19.3	12.7									33.3	22.0	17.9	11.8			61.8	40.8			19.2	12.6
Türkiye	159.8	100	3.4	2.1	0.8	0.5	2.9	1.8	9.7	6.1	24.1	15.1	33.8	21.1	27.3	17.1	0.7	0.4	14.1	8.8	16.5	10.3	60.3	37.7

Abbreviations used: GN: glomerulonephritis/sclerosis; PN: pyelonephritis; PKD: polycystic kidneys, adult type; DM: diabetes mellitus; HT: hypertension; RVD: renal vascular disease; Misc: miscellaneous; Unkn: unknown
Categories may not add up due to rounding
When cells are left empty, the data are unavailable
* Patients younger than 18 years of age are not reported
† Data include dialysis patients only
$ Hypertension is not reported separately, but is included in renal vascular disease
|| Total DM includes: 36 patients with type 1 DM, 259 patients with type 2 DM and 68 patients with unspecified type of DM
¤ Preemptive transplantations are not reported for Lazio
‡ Data on primary renal disease are available for dialysis patients only (N=2489, 98.7% of total)
+ Renal vascular disease is not reported separately, but is included in miscellaneous
& Pyelonephritis is not reported separately, but is included in miscellaneous
¶ Renal vascular disease is not reported separately, but is included in hypertension
§ Miscellaneous renal disorders include: Tubulointerstitial disease (n=604); Other systemic diseases affecting the kidney (n=294), Familial/Hereditary nephropathies (n=603), and Miscellaneous renal disorders (n=1472)

Table C.2.6
Incident counts by treatment modality
at day 1

	All	Haemodialysis						Peritoneal dialysis				Preemptive kidney transplant				Missing
		HD hospital/ centre	HD home	HD type Unkn	HF	HDF	Total HD/ HF/HDF	APD	CAPD	PD type Unkn	Total PD	Living donor	Deceased donor	Tx type Unkn	Total Tx	
	N	N	N	N	N	N	N	N	N	N	N	N	N	N	N	N
Albania	394	360	0	0	0	0	360	0	13	0	13	2	0	0	2	19
Belarus *	883	794	0	0	0	48	842	4	13	0	17	0	24	0	24	0
Croatia †	484	362	0	0	0	82	444	0	24	1	25					15
Cyprus	255	154	0	0	0	93	247	0	2	1	3	2	1	2	5	0
Czech Republic	1744			1573			1573			77	77	7	87	0	94	0
Finland	458	277	9	0	0	23	309	34	74	0	108	8	33	0	41	0
Hungary	1321	1093	0	0	17	35	1145			148	148	8	7	11	26	2
Ireland																
Israel	1807	1447	0	0	0	87	1534	86	92	2	180	89	4	0	93	0
Italy (10 of 20 regions)	4860	3156	6	153	73	720	4108	290	369	9	668	32	40	12	84	0
Italy (5 extra regions) ¤	3065	1824	6	125	8	604	2567	170	234	38	442	8	15	33	56	0
Italy (15 of 20 regions) ¤	7925	4980	12	278	81	1324	6675	460	603	47	1110	40	55	45	140	0
Kosovo *	225	223	0	0	0	0	223	0	0	0	0	2	0	0	2	0
Latvia	185	151	0	0	0	0	151	13	16	1	30	1	3	0	4	0
Lithuania	368	334	0	0	0	0	334	5	14	0	19	3	12	0	15	0
North Macedonia	423	418	0	0	0	0	418	0	2	0	2	3	0	0	3	0
Poland †	5655			5401			5401			254	254					0
Portugal	2521			2208			2208			281	281			32	32	0
Slovakia †	794	144	0	0	0	624	768	16	10	0	26					0
Spain	7287			5558			5558			1239	1239			490	490	0
Türkiye	13641	10070	38	156	3	9	10276	423	941	0	1364	1991	10	0	2001	0

Abbreviations used: HD: haemodialysis; Unkn: unknown; HF: haemofiltration; HDF: haemodiafiltration; APD: automated peritoneal dialysis; CAPD: continuous ambulatory peritoneal dialysis; PD: peritoneal dialysis; Tx: transplant
When cells are left empty, the data are unavailable
* Patients younger than 18 years of age are not reported
† Data include dialysis patients only
¤ Preemptive transplantations are not reported for Lazio

Table C.2.7

Incidence per million population by treatment modality, unadjusted

at day 1

	All	Haemodialysis						Peritoneal dialysis				Preemptive kidney transplant				Missing
		HD hospital/ centre	HD home	HD type Unkn	HF	HDF	Total HD/ HF/HDF	APD	CAPD	PD type Unkn	Total PD	Living donor	Deceased donor	Tx type Unkn	Total Tx	
	Pmp	Pmp	Pmp	Pmp	Pmp	Pmp	Pmp	Pmp	Pmp	Pmp	Pmp	Pmp	Pmp	Pmp	Pmp	Pmp
Albania	144.1	131.7	0	0	0	0	131.7	0	4.8	0	4.8	0.7	0	0	0.7	6.9
Belarus *	104.6	94.0	0	0	0	5.7	99.7	0.5	1.5	0	2.0	0	2.8	0	2.8	0
Croatia †	147.5	110.3	0	0	0	25.0	135.3	0	7.3	0.3	7.6					4.6
Cyprus	268.7	162.3	0	0	0	98.0	260.3	0	2.1	1.1	3.2	2.1	1.1	2.1	5.3	0
Czech Republic	161.1			145.3			145.3			7.1	7.1	0.6	8.0	0	8.7	0
Finland	83.4	50.4	1.6	0	0	4.2	56.3	6.2	13.5	0	19.7	1.5	6.0	0	7.5	0
Hungary	137.6	113.9	0	0	1.8	3.6	119.3			15.4	15.4	0.8	0.7	1.1	2.7	0.2
Ireland																
Israel	187.6	150.2	0	0	0	9.0	159.2	8.9	9.6	0.2	18.7	9.2	0.4	0	9.7	0
Italy (10 of 20 regions)	169.8	110.3	0.2	5.3	2.6	25.2	143.6	10.1	12.9	0.3	23.3	1.1	1.4	0.4	2.9	0
Italy (5 extra regions) ¤	174.1	103.6	0.3	7.1	0.5	34.3	145.8	9.7	13.3	2.2	25.1	0.5	0.9	1.9	3.2	0
Italy (15 of 20 regions) ¤	172.2	108.2	0.3	6.0	1.8	28.8	145.1	10.0	13.1	1.0	24.1	0.9	1.2	1.0	3.0	0
Kosovo *	133.3	132.1	0	0	0	0	132.1	0	0	0	0	1.2	0	0	1.2	0
Latvia	106.8	87.2	0	0	0	0	87.2	7.5	9.2	0.6	17.3	0.6	1.7	0	2.3	0
Lithuania	128.8	116.9	0	0	0	0	116.9	1.7	4.9	0	6.6	1.0	4.2	0	5.2	0
North Macedonia	231.6	228.9	0	0	0	0	228.9	0	1.1	0	1.1	1.6	0	0	1.6	0
Poland †	150.3			143.5			143.5			6.7	6.7					0
Portugal	236.9			207.5			207.5			26.4	26.4			3.0	3.0	0
Slovakia †	177.6	32.2	0	0	0	139.5	171.7	3.6	2.2	0	5.8					0
Spain	151.5			115.6			115.6			25.8	25.8			10.2	10.2	0
Türkiye	159.8	118.0	0.4	1.8	0	0.1	120.4	5.0	11.0	0	16.0	23.3	0.1	0	23.4	0

Abbreviations used: HD: haemodialysis; Unkn: unknown; HF: haemofiltration; HDF: haemodiafiltration; APD: automated peritoneal dialysis; CAPD: continuous ambulatory peritoneal dialysis; PD: peritoneal dialysis; Tx: transplant
Categories may not add up due to rounding
When cells are left empty, the data are unavailable
* Patients younger than 18 years of age are not reported
† Data include dialysis patients only
¤ Preemptive transplantations are not reported for Lazio

Table C.2.8

Treatment modality distribution, unadjusted

at day 1

	All	Haemodialysis						Peritoneal dialysis				Preemptive kidney transplant				Missing
		HD hospital/ centre	HD home	HD type Unkn	HF	HDF	Total HD/ HF/HDF	APD	CAPD	PD type Unkn	Total PD	Living donor	Deceased donor	Tx type Unkn	Total Tx	
	%	%	%	%	%	%	%	%	%	%	%	%	%	%	%	%
Albania	100	91.4	0	0	0	0	91.4	0	3.3	0	3.3	0.5	0	0	0.5	4.8
Belarus *	100	89.9	0	0	0	5.4	95.4	0.5	1.5	0	1.9	0	2.7	0	2.7	0
Croatia †	100	74.8	0	0	0	16.9	91.7	0	5.0	0.2	5.2					3.1
Cyprus	100	60.4	0	0	0	36.5	96.9	0	0.8	0.4	1.2	0.8	0.4	0.8	2.0	0
Czech Republic	100			90.2			90.2			4.4	4.4	0.4	5.0	0	5.4	0
Finland	100	60.5	2.0	0	0	5.0	67.5	7.4	16.2	0	23.6	1.7	7.2	0	9.0	0
Hungary	100	82.7	0	0	1.3	2.6	86.7			11.2	11.2	0.6	0.5	0.8	2.0	0.2
Ireland																
Israel	100	80.1	0	0	0	4.8	84.9	4.8	5.1	0.1	10.0	4.9	0.2	0	5.1	0
Italy (10 of 20 regions)	100	64.9	0.1	3.1	1.5	14.8	84.5	6.0	7.6	0.2	13.7	0.7	0.8	0.2	1.7	0
Italy (5 extra regions) ¤	100	59.5	0.2	4.1	0.3	19.7	83.8	5.5	7.6	1.2	14.4	0.3	0.5	1.1	1.8	0
Italy (15 of 20 regions) ¤	100	62.8	0.2	3.5	1.0	16.7	84.2	5.8	7.6	0.6	14.0	0.5	0.7	0.6	1.8	0
Kosovo *	100	99.1	0	0	0	0	99.1	0	0	0	0	0.9	0	0	0.9	0
Latvia	100	81.6	0	0	0	0	81.6	7.0	8.6	0.5	16.2	0.5	1.6	0	2.2	0
Lithuania	100	90.8	0	0	0	0	90.8	1.4	3.8	0	5.2	0.8	3.3	0	4.1	0
North Macedonia	100	98.8	0	0	0	0	98.8	0	0.5	0	0.5	0.7	0	0	0.7	0
Poland †	100			95.5			95.5			4.5	4.5					0
Portugal	100			87.6			87.6			11.1	11.1			1.3	1.3	0
Slovakia †	100	18.1	0	0	0	78.6	96.7	2.0	1.3	0	3.3					0
Spain	100			76.3			76.3			17.0	17.0			6.7	6.7	0
Türkiye	100	73.8	0.3	1.1	0	0.1	75.3	3.1	6.9	0	10.0	14.6	0.1	0	14.7	0

Abbreviations used: HD: haemodialysis; Unkn: unknown; HF: haemofiltration; HDF: haemodiafiltration; APD: automated peritoneal dialysis; CAPD: continuous ambulatory peritoneal dialysis; PD: peritoneal dialysis; Tx: transplant
Categories may not add up due to rounding
When cells are left empty, the data are unavailable
* Patients younger than 18 years of age are not reported
† Data include dialysis patients only
¤ Preemptive transplantations are not reported for Lazio

Table C.3.1

Incident counts and percentages by age and sex

at day 91

	All			0-19						20-44						45-64						65-74						75+					
	All	Male	Female	All		Male		Female		All		Male		Female		All		Male		Female		All		Male		Female		All		Male		Female	
	N (100%)	N (100%)	N (100%)	N	%	N	%	N	%	N	%	N	%	N	%	N	%	N	%	N	%	N	%	N	%	N	%	N	%	N	%	N	%
Albania	390	257	133	1	0	1	0	0	0	26	7	21	8	5	4	166	43	115	45	51	38	123	32	73	28	50	38	74	19	47	18	27	20
Belarus *	751	423	328	2	0	1	0	1	0	145	19	85	20	60	18	334	44	203	48	131	40	210	28	102	24	108	33	60	8	32	8	28	9
Croatia †	413	248	165	0	0	0	0	0	0	18	4	9	4	9	5	84	20	42	17	42	25	112	27	76	31	36	22	199	48	121	49	78	47
Cyprus	236	165	71	2	1	2	1	0	0	27	11	19	12	8	11	56	24	36	22	20	28	58	25	43	26	15	21	93	39	65	39	28	39
Czech Republic	1598	1051	547	5	0	4	0	1	0	145	9	91	9	54	10	451	28	331	31	120	22	511	32	331	31	180	33	486	30	294	28	192	35
Finland	451	308	143	9	2	5	2	4	3	67	15	48	16	19	13	160	35	104	34	56	39	113	25	77	25	36	25	102	23	74	24	28	20
Hungary	985	531	454	11	1	7	1	4	1	104	11	63	12	41	9	340	35	199	37	141	31	296	30	159	30	137	30	234	24	103	19	131	29
Ireland																																	
Israel	1723	1105	618	29	2	19	2	10	2	157	9	91	8	66	11	483	28	329	30	154	25	498	29	310	28	188	30	556	32	356	32	200	32
Italy (10 of 20 regions)	4458	2936	1522	23	1	15	1	8	1	306	7	195	7	111	7	1154	26	758	26	396	26	1183	27	784	27	399	26	1792	40	1184	40	608	40
Italy (5 extra regions)																																	
Italy (15 of 20 regions)																																	
Kosovo *	191	100	91	1	1	1	1	0	0	17	9	10	10	7	8	69	36	41	41	28	31	68	36	31	31	37	41	36	19	17	17	19	21
Latvia	180	98	82	1	1	0	0	1	1	24	13	12	12	12	15	72	40	43	44	29	35	52	29	29	30	23	28	31	17	14	14	17	21
Lithuania	352	211	141	5	1	3	1	2	1	44	13	26	12	18	13	132	38	86	41	46	33	78	22	46	22	32	23	93	26	50	24	43	30
North Macedonia	341	203	138	3	1	3	1	0	0	27	8	17	8	10	7	129	38	74	36	55	40	102	30	67	33	35	25	80	23	42	21	38	28
Poland																																	
Portugal	2426																																
Slovakia †	595	358	237	3	1	1	0	2	1	59	10	28	8	31	13	227	38	139	39	88	37	189	32	121	34	68	29	117	20	69	19	48	20
Spain																																	
Türkiye																																	

Categories may not add up due to missing values or rounding
When cells are left empty, the data are unavailable
* Patients younger than 18 years of age are not reported
† Data include dialysis patients only

Table C.3.2

Incidence per million (age-related) population by age and sex, unadjusted

at day 91

	All			0-19			20-44			45-64			65-74			75+		
	All	Male	Female	All	Male	Female	All	Male	Female	All	Male	Female	All	Male	Female	All	Male	Female
	Pmp	Pmp	Pmp	Pmarp	Pmarp	Pmarp	Pmarp	Pmarp	Pmarp	Pmarp	Pmarp	Pmarp	Pmarp	Pmarp	Pmarp	Pmarp	Pmarp	Pmarp
Albania	142.6	189.9	96.3	1.6	3.2	0	27.2	43.6	10.5	231.1	330.7	137.6	443.1	550.4	345.0	429.3	575.3	297.8
Belarus *	88.9	108.5	72.2	1.1	1.1	1.1	51.0	60.1	42.0	142.6	190.4	102.7	228.0	292.2	188.9	119.8	250.2	75.1
Croatia †	125.9	156.1	97.5	0	0	0	18.1	17.6	18.6	92.4	94.9	90.2	255.7	383.1	150.2	637.2	1052.5	395.2
Cyprus	248.7	354.7	146.7	10.3	20.1	0	76.6	108.0	45.3	234.4	320.3	158.1	632.8	966.6	318.1	1277.2	1988.0	697.9
Czech Republic	147.6	198.0	99.1	2.2	3.4	0.9	43.3	53.3	32.9	152.2	222.2	81.4	403.7	574.5	261.0	516.0	832.6	326.1
Finland	82.1	113.3	51.6	8.0	8.7	7.3	38.7	53.7	22.7	118.1	152.8	83.1	168.9	242.4	102.4	166.1	294.0	77.3
Hungary	102.6	114.8	91.3	5.8	7.2	4.4	34.2	40.2	27.7	125.8	149.2	103.0	253.6	322.8	203.0	290.9	385.8	243.8
Ireland																		
Israel	178.9	231.2	127.3	8.6	11.0	6.1	50.0	57.6	42.3	258.6	361.1	161.0	706.2	946.0	498.0	981.5	1480.9	613.3
Italy (10 of 20 regions)	155.8	210.1	104.0	4.6	5.9	3.3	38.7	48.3	28.7	130.2	174.0	87.9	352.8	494.8	225.6	508.4	822.3	291.6
Italy (5 extra regions)																		
Italy (15 of 20 regions)																		
Kosovo *	113.2	117.7	108.6	1.6	3.0	0	26.3	30.9	21.7	241.8	291.4	193.5	900.3	848.6	948.8	953.5	1035.1	890.8
Latvia	103.9	122.1	88.2	2.7	0	5.6	45.8	44.7	46.9	150.5	192.7	113.7	266.0	383.1	192.0	184.7	301.2	140.1
Lithuania	123.2	158.0	92.7	8.9	10.4	7.3	49.0	56.6	41.1	159.6	221.4	104.8	252.2	373.6	171.9	354.5	643.8	232.8
North Macedonia	186.7	224.4	149.8	7.4	14.3	0	45.7	56.7	34.5	258.6	301.7	216.9	485.2	677.6	314.3	661.6	817.0	546.7
Poland																		
Portugal	228.0																	
Slovakia †	133.1	163.7	103.7	3.2	2.1	4.4	39.5	36.6	42.7	184.2	226.5	142.2	371.8	541.0	238.8	388.4	665.2	243.0
Spain																		
Türkiye																		

When cells are left empty, the data are unavailable
* Patients younger than 18 years of age are not reported
† Data include dialysis patients only

Table C.3.3

Sex, mean age, and median age of incident patients

at day 91

	All						Male						Female					
	%	Mean (years)	SD	Median (years)	P25	P75	%	Mean (years)	SD	Median (years)	P25	P75	%	Mean (years)	SD	Median (years)	P25	P75
Albania	100	62.8	15.2	64.0	54.0	72.0	65.9	64.2	12.3	64.5	57.0	73.0	34.1	60.1	16.4	64.0	53.3	71.0
Belarus *	100						56.3						43.7					
Croatia †	100	66.3	13.5	69.0	58.0	77.0	60.0	67.0	12.4	69.0	57.0	76.0	40.0	65.4	15.0	69.0	61.0	78.0
Cyprus	100	67.0	16.1	70.0	59.0	78.0	69.9	66.8	16.1	70.0	60.0	78.0	30.1	67.3	15.9	71.0	58.0	78.5
Czech Republic	100	65.8	14.2	69.0	58.0	76.0	65.8	65.2	13.9	68.0	58.0	75.0	34.2	66.8	14.7	71.0	59.0	77.0
Finland	100	61.9	16.6	64.5	52.3	74.3	68.3	62.4	16.2	65.8	54.3	75.0	31.7	60.7	17.2	63.3	50.1	73.4
Hungary	100	62.9	15.2	66.0	53.0	74.0	53.9	61.4	15.0	64.0	51.0	72.0	46.1	64.6	15.3	68.0	55.0	75.0
Ireland																		
Israel	100	65.9	16.4	69.2	57.9	77.3	64.1	66.0	16.0	69.5	58.1	77.1	35.9	65.8	17.1	69.0	57.3	77.9
Italy (10 of 20 regions)	100	66.9	14.6	70.7	57.9	76.9	65.9	67.1	14.4	70.7	58.1	76.9	34.1	66.7	14.7	70.6	57.6	76.9
Italy (5 extra regions)																		
Italy (15 of 20 regions)																		
Kosovo *	100	64.0	12.7	67.0	54.0	71.0	52.4	62.3	13.2	64.0	54.0	71.0	47.6	65.9	11.5	69.0	61.0	77.0
Latvia	100	61.7	15.3	63.0	53.0	72.0	54.4	61.9	14.5	62.0	54.3	71.0	45.6	61.5	16.3	64.5	51.3	74.0
Lithuania	100	63.0	16.6	64.8	53.8	76.3	59.9	62.1	16.2	64.0	51.9	73.8	40.1	64.3	17.3	66.1	55.3	78.0
North Macedonia	100	64.0	13.3	66.0	56.0	74.0	59.5	63.6	13.1	65.0	57.5	73.0	40.5	64.6	13.4	66.5	56.0	75.0
Poland																		
Portugal																		
Slovakia †	100	62.6	14.2	65.0	54.0	73.0	60.2	62.9	13.6	66.0	54.0	72.3	39.8	62.2	15.1	64.0	54.0	73.0
Spain																		
Türkiye																		

Categories may not add up due to missing values or rounding
When cells are left empty, the data are unavailable
* Patients younger than 18 years of age are not reported
† Data include dialysis patients only

Table C.3.4

Incidence per million population, adjusted

at day 91, adjusted for age and sex

	All	Male	Female
	Pmp	Pmp	Pmp
Albania	155.8	204.8	108.9
Belarus			
Croatia †	125.3	158.2	93.8
Cyprus	281.5	389.6	178.2
Czech Republic	154.4	210.4	100.9
Finland	80.1	109.7	51.9
Hungary	105.8	122.3	90.0
Ireland			
Israel	271.1	352.3	193.4
Italy (10 of 20 regions)	138.4	184.3	94.5
Italy (5 extra regions)			
Italy (15 of 20 regions)			
Kosovo *	286.8	286.1	287.4
Latvia	108.9	133.8	85.2
Lithuania	130.0	173.0	88.9
North Macedonia	201.6	237.1	167.7
Poland			
Portugal			
Slovakia †	147.2	187.1	109.0
Spain			
Türkiye			

When cells are left empty, the data are unavailable
* Patients younger than 18 years of age are not reported
† Data include dialysis patients only

Table C.3.5

Incidence per million population and percentages by primary renal disease (1995 PRD codes), unadjusted

at day 91

	All		GN		PN		PKD		DM						HT		RVD		Misc		Unkn		Missing	
									Type 1		Type 2		Total											
	Pmp	%	Pmp	%	Pmp	%	Pmp	%	Pmp	%	Pmp	%	Pmp	%	Pmp	%	Pmp	%	Pmp	%	Pmp	%	Pmp	%
Albania	142.6	100	29.3	20.5	18.3	12.8	9.1	6.4	2.9	2.1	31.1	21.8	34.0	23.8	25.6	17.9	2.6	1.8	12.8	9.0	9.1	6.4	1.8	1.3
Belarus *	88.9	100	18.7	21.0	8.5	9.6	7.2	8.1	5.1	5.7	15.3	17.2	20.4	22.9	10.3	11.6	6.8	7.6	16.8	18.9	0.2	0.3	0	0
Croatia †	125.9	100	12.5	9.9	1.5	1.2	12.2	9.7	2.7	2.2	29.9	23.7	32.6	25.9	33.5	26.6	3.0	2.4	23.8	18.9	6.7	5.3	0	0
Cyprus	248.7	100	15.8	6.4	0	0	10.5	4.2					85.3	34.3	47.4	19.1	8.4	3.4	43.2	17.4	37.9	15.3	0	0
Czech Republic $ ||	147.6	100	14.3	9.7	3.2	2.2	6.6	4.4					30.7	20.8			22.3	15.1	20.5	13.9	46.4	31.4	3.6	2.4
Finland	82.1	100	8.0	9.8	1.3	1.6	8.9	10.9	13.1	16.0	15.3	18.6	28.4	34.6	6.2	7.5	0.4	0.4	10.4	12.6	15.1	18.4	3.5	4.2
Hungary	102.6	100	7.2	7.0	12.4	12.1	7.4	7.2	2.0	1.9	31.7	30.9	33.6	32.8	13.8	13.4	0.5	0.5	1.5	1.4	1.9	1.8	24.4	23.8
Ireland																								
Israel	178.9	100	7.3	4.1	2.5	1.4	4.5	2.5	4.2	2.3	64.8	36.2	68.9	38.5	17.2	9.6	1.2	0.7	16.0	8.9	50.1	28.0	11.1	6.2
Italy (10 of 20 regions)	155.8	100	14.5	9.3	5.2	3.3	7.0	4.5					18.6	12.0	15.9	10.2	3.9	2.5	0.6	0.4	76.4	49.0	13.7	8.8
Italy (5 extra regions)																								
Italy (15 of 20 regions)																								
Kosovo *	113.2	100	10.1	8.9	29.0	25.7	5.9	5.2	1.2	1.0	32.6	28.8	33.8	29.8	19.0	16.8	4.1	3.7	7.7	6.8	3.6	3.1	0	0
Latvia	103.9	100	19.0	18.3	8.7	8.3	11.0	10.6	5.2	5.0	14.4	13.9	19.6	18.9	13.9	13.3	1.2	1.1	29.4	28.3	1.2	1.1	0	0
Lithuania	123.2	100	16.8	13.6	13.3	10.8	10.8	8.8					20.6	16.8	26.6	21.6	4.2	3.4	21.3	17.3	9.4	7.7	0	0
North Macedonia	186.7	100	12.6	6.7	4.9	2.6	12.6	6.7	0	0	50.4	27.0	50.4	27.0	66.3	35.5	0	0	26.8	14.4	13.1	7.0	0	0
Poland																								
Portugal																								
Slovakia †	133.1	100	16.3	12.3	10.7	8.1	3.1	2.4					40.7	30.6	10.3	7.7	8.1	6.1	42.7	32.1	1.1	0.8	0	0
Spain																								
Türkiye																								

Abbreviations used: GN: glomerulonephritis/sclerosis; PN: pyelonephritis; PKD: polycystic kidneys, adult type; DM: diabetes mellitus; HT: hypertension; RVD: renal vascular disease; Misc: miscellaneous; Unkn: unknown
Categories may not add up due to rounding
When cells are left empty, the data are unavailable
* Patients younger than 18 years of age are not reported
† Data include dialysis patients only
$ Hypertension is not reported separately, but is included in renal vascular disease
|| Total DM includes: 29 patients with type 1 DM, 249 patients with type 2 DM and 54 patients with unspecified type of DM

Table C.3.6

Incident counts by treatment modality

at day 91

	All	Haemodialysis						Peritoneal dialysis				Kidney transplant				Missing
		HD hospital/ centre	HD home	HD type Unkn	HF	HDF	Total HD/ HF/HDF	APD	CAPD	PD type Unkn	Total PD	Living donor	Deceased donor	Tx type Unkn	Total Tx	
	N	N	N	N	N	N	N	N	N	N	N	N	N	N	N	N
Albania	390	373	0	0	0	0	373	0	13	0	13	3	0	0	3	1
Belarus *	751	543	0	0	5	130	678	4	46	0	50	0	23	0	23	0
Croatia †	413	307	0	0	0	81	388	0	24	1	25					0
Cyprus	236	147	0	0	0	81	228	0	2	1	3	2	1	2	5	0
Czech Republic	1598	472	2	0	0	953	1427	22	26	29	77	7	87	0	94	0
Finland	451	228	15	0	0	55	298	57	47	0	104	10	38	0	48	1
Hungary	985	771	0	0	2	24	797			147	147	9	11	0	20	21
Ireland																
Israel	1723	1352	0	0	0	83	1435	86	90	2	178	105	5	0	110	0
Italy (10 of 20 regions)	4458	2861	6	145	63	651	3726	287	362	1	650	32	38	12	82	0
Italy (5 extra regions)																
Italy (15 of 20 regions)																
Kosovo *	191	189	0	0	0	0	189	0	0	0	0	2	0	0	2	0
Latvia	180	146	0	0	0	0	146	13	17	0	30	1	3	0	4	0
Lithuania	352	318	0	0	0	0	318	5	14	0	19	3	12	0	15	0
North Macedonia	341	336	0	0	0	0	336	0	2	0	2	3	0	0	3	0
Poland																
Portugal #	2426			2116			2116			278	278			32	32	0
Slovakia †	595	91	0	0	0	480	571	15	9	0	24					0
Spain																
Türkiye																

Abbreviations used: HD: haemodialysis; Unkn: unknown; HF: haemofiltration; HDF: haemodiafiltration; APD: automated peritoneal dialysis; CAPD: continuous ambulatory peritoneal dialysis; PD: peritoneal dialysis; Tx: transplant
When cells are left empty, the data are unavailable
* Patients younger than 18 years of age are not reported
† Data include dialysis patients only
# Only preemptive transplantations (at day 1) are included

Table C.3.7

Incidence per million population by treatment modality, unadjusted

at day 91

	All	Haemodialysis						Peritoneal dialysis				Kidney transplant				Missing
		HD hospital/ centre	HD home	HD type Unkn	HF	HDF	Total HD/ HF/HDF	APD	CAPD	PD type Unkn	Total PD	Living donor	Deceased donor	Tx type Unkn	Total Tx	
	Pmp	Pmp	Pmp	Pmp	Pmp	Pmp	Pmp	Pmp	Pmp	Pmp	Pmp	Pmp	Pmp	Pmp	Pmp	Pmp
Albania	142.6	136.4	0	0	0	0	136.4	0	4.8	0	4.8	1.1	0	0	1.1	0.4
Belarus *	88.9	64.3	0	0	0.6	15.4	80.3	0.5	5.4	0	5.9	0	2.7	0	2.7	0
Croatia †	125.9	93.6	0	0	0	24.7	118.3	0	7.3	0.3	7.6					0
Cyprus	248.7	154.9	0	0	0	85.3	240.2	0	2.1	1.1	3.2	2.1	1.1	2.1	5.3	0
Czech Republic	147.6	43.6	0.2	0	0	88.0	131.8	2.0	2.4	2.7	7.1	0.6	8.0	0	8.7	0
Finland	82.1	41.5	2.7	0	0	10.0	54.3	10.4	8.6	0	18.9	1.8	6.9	0	8.7	0.2
Hungary	102.6	80.3	0	0	0.2	2.5	83.0			15.3	15.3	0.9	1.1	0	2.1	2.2
Ireland																
Israel	178.9	140.3	0	0	0	8.6	149.0	8.9	9.3	0.2	18.5	10.9	0.5	0	11.4	0
Italy (10 of 20 regions)	155.8	100.0	0.2	5.1	2.2	22.8	130.2	10.0	12.7	0	22.7	1.1	1.3	0.4	2.9	0
Italy (5 extra regions)																
Italy (15 of 20 regions)																
Kosovo *	113.2	112.0	0	0	0	0	112.0	0	0	0	0	1.2	0	0	1.2	0
Latvia	103.9	84.3	0	0	0	0	84.3	7.5	9.8	0	17.3	0.6	1.7	0	2.3	0
Lithuania	123.2	111.3	0	0	0	0	111.3	1.7	4.9	0	6.6	1.0	4.2	0	5.2	0
North Macedonia	186.7	184.0	0	0	0	0	184.0	0	1.1	0	1.1	1.6	0	0	1.6	0
Poland																
Portugal #	228.0			198.9			198.9			26.1	26.1			3.0	3.0	0
Slovakia †	133.1	20.4	0	0	0	107.3	127.7	3.4	2.0	0	5.4					0
Spain																
Türkiye																

Abbreviations used: HD: haemodialysis; Unkn: unknown; HF: haemofiltration; HDF: haemodiafiltration; APD: automated peritoneal dialysis; CAPD: continuous ambulatory peritoneal dialysis; PD: peritoneal dialysis; Tx: transplant
Categories may not add up due to rounding
When cells are left empty, the data are unavailable
* Patients younger than 18 years of age are not reported
† Data include dialysis patients only
# Only preemptive transplantations (at day 1) are included

Table C.3.8

Treatment modality distribution, unadjusted

at day 91

	All	Haemodialysis						Peritoneal dialysis				Kidney transplant				Missing
		HD hospital/ centre	HD home	HD type Unkn	HF	HDF	Total HD/ HF/HDF	APD	CAPD	PD type Unkn	Total PD	Living donor	Deceased donor	Tx type Unkn	Total Tx	
	%	%	%	%	%	%	%	%	%	%	%	%	%	%	%	%
Albania	100	95.6	0	0	0	0	95.6	0	3.3	0	3.3	0.8	0	0	0.8	0.3
Belarus *	100	72.3	0	0	0.7	17.3	90.3	0.5	6.1	0	6.7	0	3.1	0	3.1	0
Croatia †	100	74.3	0	0	0	19.6	93.9	0	5.8	0.2	6.1					0
Cyprus	100	62.3	0	0	0	34.3	96.6	0	0.8	0.4	1.3	0.8	0.4	0.8	2.1	0
Czech Republic	100	29.5	0.1	0	0	59.6	89.3	1.4	1.6	1.8	4.8	0.4	5.4	0	5.9	0
Finland	100	50.6	3.3	0	0	12.2	66.1	12.6	10.4	0	23.1	2.2	8.4	0	10.6	0.2
Hungary	100	78.3	0	0	0.2	2.4	80.9			14.9	14.9	0.9	1.1	0	2.0	2.1
Ireland																
Israel	100	78.5	0	0	0	4.8	83.3	5.0	5.2	0.1	10.3	6.1	0.3	0	6.4	0
Italy (10 of 20 regions)	100	64.2	0.1	3.3	1.4	14.6	83.6	6.4	8.1	0	14.6	0.7	0.9	0.3	1.8	0
Italy (5 extra regions)																
Italy (15 of 20 regions)																
Kosovo *	100	99.0	0	0	0	0	99.0	0	0	0	0	1.0	0	0	1.0	0
Latvia	100	81.1	0	0	0	0	81.1	7.2	9.4	0	16.7	0.6	1.7	0	2.2	0
Lithuania	100	90.3	0	0	0	0	90.3	1.4	4.0	0	5.4	0.9	3.4	0	4.3	0
North Macedonia	100	98.5	0	0	0	0	98.5	0	0.6	0	0.6	0.9	0	0	0.9	0
Poland																
Portugal #	100			87.2			87.2			11.5	11.5			1.3	1.3	0
Slovakia †	100	15.3	0	0	0	80.7	96.0	2.5	1.5	0	4.0					0
Spain																
Türkiye																

Abbreviations used: HD: haemodialysis; Unkn: unknown; HF: haemofiltration; HDF: haemodiafiltration; APD: automated peritoneal dialysis; CAPD: continuous ambulatory peritoneal dialysis; PD: peritoneal dialysis; Tx: transplant
Categories may not add up due to rounding
When cells are left empty, the data are unavailable
* Patients younger than 18 years of age are not reported
† Data include dialysis patients only
# Only preemptive transplantations (at day 1) are included

Table C.4.1

Prevalent counts and percentages by age and sex

prevalent patients on December 31

	All			0-19						20-44						45-64						65-74						75+					
	All	Male	Female	All		Male		Female		All		Male		Female		All		Male		Female		All		Male		Female		All		Male		Female	
	N (100%)	N (100%)	N (100%)	N	%	N	%	N	%	N	%	N	%	N	%	N	%	N	%	N	%	N	%	N	%	N	%	N	%	N	%	N	%
Albania	1724	1030	694	18	1	12	1	6	1	398	23	246	24	152	22	578	34	347	34	231	33	479	28	269	26	210	30	251	15	156	15	95	14
Belarus *	4374	2454	1920	16	0	9	0	7	0	1233	28	720	29	513	27	2025	46	1153	47	872	45	892	20	457	19	435	23	208	5	115	5	93	5
Croatia †	2159	1306	853	1	0	1	0	0	0	102	5	58	4	44	5	457	21	283	22	174	20	555	26	355	27	200	23	1044	48	609	47	435	51
Cyprus																																	
Czech Republic † &	5937	3749	2188	9	0	7	0	2	0	417	7	256	7	161	7	1638	28	1129	30	509	23	1865	31	1206	32	659	30	2008	34	1151	31	857	39
Finland	5292	3275	2017	124	2	76	2	48	2	819	15	502	15	317	16	1991	38	1227	37	764	38	1327	25	811	25	516	26	1031	19	659	20	372	18
Hungary	8862	5048	3814	118	1	72	1	46	1	1319	15	802	16	517	14	3710	42	2247	45	1463	38	2265	26	1260	25	1005	26	1450	16	667	13	783	21
Ireland	5257	3375	1882	105	2	59	2	46	2	1002	19	651	19	351	19	2130	41	1343	40	787	42	1148	22	751	22	397	21	872	17	571	17	301	16
Israel †	7087	4453	2634	38	1	23	1	15	1	479	7	282	6	197	7	1901	27	1226	28	675	26	2103	30	1329	30	774	29	2566	36	1593	36	973	37
Italy (10 of 20 regions)	34633	22199	12434	343	1	207	1	136	1	3904	11	2466	11	1438	12	12410	36	7970	36	4440	36	8341	24	5359	24	2982	24	9635	28	6197	28	3438	28
Italy (5 extra regions) ¤	19135	12348	6787	148	1					1600	8					6777	35					4730	25					5880	31				
Italy (15 of 20 regions) ¤	53768	34547	19221	491	1					5504	10					19187	36					13071	24					15515	29				
Kosovo *	1063	608	455	4	0	3	0	1	0	148	14	91	15	57	13	464	44	277	46	187	41	315	30	174	29	141	31	132	12	63	10	69	15
Latvia	1145	635	510	13	1	6	1	7	1	247	22	150	24	97	19	489	43	277	44	212	42	270	24	137	22	133	26	126	11	65	10	61	12
Lithuania	2466																																
North Macedonia	1779	1123	656	18	1	13	1	5	1	239	13	154	14	85	13	764	43	493	44	271	41	489	27	301	27	188	29	269	15	162	14	107	16
Poland†	20536	12534	8002	46	0					2274	11					6340	31					6520	32					5356	26				
Portugal	21511	13274	8237																														
Slovakia †	3183	1957	1226	12	0	4	0	8	1	309	10	208	11	101	8	1007	32	684	35	323	26	1076	34	652	33	424	35	779	24	409	21	370	30
Spain § #	67604	42278	23720	338	1	219	1	116	0	7492	11	4590	11	2895	12	25201	37	16141	38	9051	38	16510	24	10837	26	5671	24	16482	24	10491	25	5987	25
Türkiye	89527																																

Categories may not add up due to missing values or rounding
When cells are left empty, the data are unavailable
* Patients younger than 18 years of age are not reported
† Data include dialysis patients only
& Additionally there are 6021 prevalent kidney transplant recipients
¤ Excluding data from Trentino; transplant patients are not reported for Lazio
§ Data presented in column 0-19 refer to patients aged 0-14 years and data presented in column 20-44 refer to patients aged 15-44 years
# Including 1606 patients (2.4%) with unknown sex and 1581 patients (2.3%) with unknown age

Table C.4.2

Prevalence per million (age-related) population by age and sex, unadjusted

prevalent patients on December 31

	All			0-19			20-44			45-64			65-74			75+		
	All	Male	Female	All	Male	Female	All	Male	Female	All	Male	Female	All	Male	Female	All	Male	Female
	Pmp	Pmp	Pmp	Pmarp	Pmarp	Pmarp	Pmarp	Pmarp	Pmarp	Pmarp	Pmarp	Pmarp	Pmarp	Pmarp	Pmarp	Pmarp	Pmarp	Pmarp
Albania	630.5	761.1	502.6	29.5	38.8	20.0	416.0	510.8	319.9	804.7	997.8	623.4	1725.7	2028.2	1448.8	1456.1	1909.4	1047.7
Belarus *	518.0	629.3	422.5	8.7	9.5	7.8	433.9	509.4	359.2	864.7	1081.6	683.5	968.5	1309.0	760.7	415.3	899.2	249.4
Croatia †	658.1	821.9	504.2	1.6	3.1	0	102.7	113.7	91.0	503.0	639.2	373.5	1267.0	1789.3	834.5	3342.8	5297.3	2204.2
Cyprus																		
Czech Republic †	548.3	706.2	396.4	3.9	5.9	1.8	124.5	149.8	98.2	552.6	758.1	345.1	1473.2	2093.1	955.4	2131.8	3259.6	1455.5
Finland	963.6	1204.7	727.3	110.3	132.2	87.4	473.3	561.8	378.9	1470.2	1803.3	1133.9	1983.3	2553.1	1468.3	1678.9	2618.1	1026.5
Hungary	923.1	1091.4	766.7	62.7	74.5	50.3	433.1	512.1	349.5	1373.0	1684.8	1069.2	1940.5	2558.3	1489.5	1802.8	2498.5	1457.2
Ireland	1020.9	1326.4	722.6	77.8	85.5	69.7	579.3	768.5	397.8	1646.9	2093.5	1207.3	2603.0	3466.2	1769.4	2600.8	3834.2	1615.1
Israel †	735.7	931.7	542.6	11.3	13.4	9.2	152.5	178.6	126.2	1018.0	1345.8	705.8	2982.1	4055.5	2050.3	4529.6	6626.5	2983.7
Italy (10 of 20 regions)	1210.4	1588.5	849.4	69.1	80.9	56.5	493.6	611.0	371.3	1400.7	1829.9	985.6	2487.5	3381.9	1686.2	2733.5	4304.0	1648.9
Italy (5 extra regions) ¤	1121.7	1479.7	778.8	49.3			336.5			1270.2			2465.9			2870.7		
Italy (15 of 20 regions) ¤	1182.3	1554.8	862.5	62.4			439.7			1360.3			2457.1			2740.3		
Kosovo *	629.9	715.6	543.0	6.2	9.0	3.2	229.0	281.4	176.5	1625.9	1969.0	1292.4	4170.7	4763.2	3615.7	3496.3	3835.8	3234.8
Latvia	660.9	791.2	548.5	35.5	31.8	39.4	470.9	558.6	379.0	1022.4	1241.4	830.9	1381.0	1809.6	1110.1	750.8	1398.4	502.8
Lithuania	863.1																	
North Macedonia	974.1	1241.2	711.9	44.3	62.1	25.4	405.0	513.4	292.8	1531.5	2010.2	1068.6	2326.0	3044.3	1688.2	2224.6	3151.1	1539.3
Poland †	545.6	689.3	411.3	6.1			180.3			640.5			1408.7			1833.4		
Portugal	2021.8	2611.2	1482.5															
Slovakia †	711.8	894.8	536.6	12.8	8.3	17.5	207.1	271.6	139.1	817.0	1114.4	522.0	2116.6	2915.2	1489.3	2585.8	3942.9	1873.1
Spain § #	1405.9	1794.1	967.4	37.1	46.7	26.3	509.6	618.4	397.7	1726.5	2228.1	1231.0	3367.4	4708.5	2180.1	3444.6	5487.1	2083.9
Türkiye	1048.7																	

When cells are left empty, the data are unavailable
* Patients younger than 18 years of age are not reported
† Data include dialysis patients only
¤ Excluding data from Trentino; transplant patients are not reported for Lazio
§ Data presented in column 0-19 refer to patients aged 0-14 years and data presented in column 20-44 refer to patients aged 15-44 years
# Including 1606 patients (2.4%) with unknown sex and 1581 patients (2.3%) with unknown age

Table C.4.3

Sex, mean age, and median age

prevalent patients on December 31

	All						Male						Female					
	%	Mean (years)	SD	Median (years)	P25	P75	%	Mean (years)	SD	Median (years)	P25	P75	%	Mean (years)	SD	Median (years)	P25	P75
Albania	100	52.8	12.7	52.9	42.3	68.8	59.7	54.2	14.5	55.1	39.0	73.0	40.3	50.6	10.6	50.8	49.2	63.4
Belarus *	100						56.1						43.9					
Croatia †	100	66.7	15.2	69.0	58.0	78.0	60.5	66.0	15.0	68.0	58.0	77.0	39.5	67.7	15.5	70.0	58.0	79.0
Cyprus																		
Czech Republic †	100	67.0	13.6	70.0	59.0	77.0	63.1	66.4	13.2	69.0	59.0	76.0	36.9	68.1	14.1	71.5	60.0	78.0
Finland	100	60.2	16.6	62.9	50.6	72.7	61.9	60.4	16.5	62.8	50.3	73.1	38.1	60.0	16.6	62.9	50.8	72.2
Hungary	100	59.3	15.4	61.0	49.0	71.0	57.0	58.1	15.0	59.0	48.0	69.0	43.0	60.9	15.7	63.0	50.0	73.0
Ireland	100	57.9	16.6	59.0	47.0	70.0	64.2	58.2	16.5	59.0	47.0	71.0	35.8	57.3	16.8	58.5	46.0	70.0
Israel †	100	68.3	14.5	70.9	60.7	78.2	62.8	68.4	14.2	70.8	60.7	78.0	37.2	68.3	14.9	71.0	60.4	78.7
Italy (10 of 20 regions)	100	62.2	15.9	65.7	51.8	75.5	64.1	62.3	15.8	65.8	51.9	75.5	35.9	62.1	16.0	65.6	51.6	75.5
Italy (5 extra regions)																		
Italy (15 of 20 regions)																		
Kosovo *	100	61.1	13.5	63.0	30.0	70.0	57.2	59.9	13.9	62.0	31.0	69.0	42.8	62.7	12.9	64.0	30.0	70.0
Latvia	100	56.8	15.5	59.0	46.0	68.0	55.5	55.8	15.4	58.0	45.0	67.0	44.5	58.0	15.6	60.5	47.3	70.0
Lithuania																		
North Macedonia	100	60.2	14.3	62.0	51.0	71.0	63.1	59.8	14.2	62.0	51.0	70.0	36.9	60.7	14.4	63.0	52.0	72.0
Poland †	100						61.0						39.0					
Portugal †	100	67.7					61.7						38.3					
Slovakia †	100	64.1	14.2	67.0	56.0	74.0	61.5	62.9	14.2	66.0	54.5	73.0	38.5	66.0	14.1	69.0	59.0	76.0
Spain #	100	61.4	15.1	65.0	51.9	74.9	62.5	61.6	15.0	65.2	52.1	74.9	35.1	61.1	15.4	64.5	51.4	75.1
Türkiye																		

Categories may not add up due to missing values or rounding
When cells are left empty, the data are unavailable
* Patients younger than 18 years of age are not reported
† Data include dialysis patients only
# Sex was unknown for 2.4% and age was unknown for 2.3% of prevalent patients

Table C.4.4

Prevalence per million population, adjusted

prevalent patients on December 31, adjusted for age and sex

	All	Male	Female
	Pmp	Pmp	Pmp
Albania	643.8	791.5	502.5
Belarus			
Croatia †	665.1	843.8	494.3
Cyprus			
Czech Republic †	576.3	751.9	408.3
Finland	951.8	1186.0	727.9
Hungary	943.1	1136.9	757.8
Ireland	1193.3	1539.0	862.9
Israel †	1134.1	1437.2	844.3
Italy (10 of 20 regions)	1103.1	1434.3	786.5
Italy (5 extra regions)			
Italy (15 of 20 regions)			
Kosovo *	1392.5	1542.6	1248.9
Latvia	679.8	834.4	532.0
Lithuania			
North Macedonia	1024.7	1307.3	754.4
Poland			
Portugal			
Slovakia †	796.9	1031.0	573.1
Spain			
Türkiye			

When cells are left empty, the data are unavailable
† Data include dialysis patients only
* Patients younger than 18 years of age are not reported

Table C.4.5

Prevalence per million population and percentages by primary renal disease (1995 PRD codes), unadjusted

prevalent patients on December 31

	All		GN		PN		PKD		DM						HT		RVD		Misc		Unkn		Missing	
									Type 1		Type 2		Total											
	Pmp	%	Pmp	%	Pmp	%	Pmp	%	Pmp	%	Pmp	%	Pmp	%	Pmp	%	Pmp	%	Pmp	%	Pmp	%	Pmp	%
Albania	630.5	100	101.3	16.1	101.7	16.1	46.8	7.4	14.3	2.3	152.1	24.1	166.4	26.4	69.9	11.1	14.3	2.3	64.0	10.2	56.7	9.0	9.5	1.5
Belarus *	518.0	100	214.0	41.3	39.3	7.6	51.5	9.9	27.4	5.3	35.6	6.9	63.0	12.2	44.6	8.6	16.0	3.1	87.8	16.9	1.8	0.3	0	0
Croatia †	658.1	100	109.7	16.7	24.1	3.7	67.4	10.2	16.2	2.5	144.8	22.0	160.9	24.5	142.7	21.7	15.2	2.3	85.3	13.0	43.6	6.6	9.1	1.4
Cyprus																								
Czech Republic † $ | |	548.3	100	73.1	13.3	16.0	2.9	28.3	5.2					133.7	24.4			93.3	17.0	92.6	16.9	66.0	12.0	45.3	8.3
Finland	963.6	100	203.6	21.1	39.0	4.0	143.3	14.9	146.8	15.2	85.4	8.9	232.2	24.1	45.0	4.7	1.8	0.2	193.4	20.1	100.9	10.5	4.6	0.5
Hungary	923.1	100	64.7	7.0	100.5	10.9	72.0	7.8	28.8	3.1	190.4	20.6	219.2	23.7	120.8	13.1	4.1	0.4	13.9	1.5	18.4	2.0	309.6	33.5
Ireland																								
Israel †	735.7	100	51.3	7.0	14.4	2.0	23.3	3.2	25.5	3.5	304.6	41.4	330.1	44.9	83.6	11.4	8.8	1.2	77.2	10.5	144.1	19.6	2.9	0.4
Italy (10 of 20 regions)	1210.4	100	213.2	17.6	58.9	4.9	99.7	8.2					118.7	9.8	117.7	9.7	28.4	2.4	135.2	11.2	342.2	28.3	96.4	8.0
Italy (5 extra regions) ¤	1121.7	100	27.6	2.5	10.9	1.0	26.0	2.3					54.3	4.8			47.7	4.3	54.4	4.9	74.8	6.7	825.9	73.6
Italy (15 of 20 regions) ¤	1182.3	100	144.5	12.2	41.1	3.5	72.5	6.1					95.1	8.0	74.1	6.2	35.8	3.0	105.5	8.9	243.4	20.6	370.5	31.3
Kosovo *	629.9	100	51.6	8.2	200.9	31.9	36.1	5.7	11.3	1.8	164.1	26.1	175.4	27.8	100.7	16.0	20.7	3.3	30.8	4.9	13.6	2.2	0	0
Latvia	660.9	100	189.9	28.7	76.2	11.5	94.7	14.3	39.3	5.9	42.7	6.5	82.0	12.4	62.3	9.4	4.0	0.6	133.9	20.3	17.9	2.7	0	0
Lithuania	863.1	100	77.0	8.9	54.9	6.4	44.8	5.2					68.6	7.9	109.5	12.7	12.2	1.4	77.0	8.9	15.7	1.8	403.2	46.7
North Macedonia	974.1	100	134.7	13.8	66.8	6.9	94.7	9.7	15.9	1.6	167.0	17.1	182.9	18.8	278.7	28.6	1.6	0.2	127.6	13.1	87.1	8.9	0	0
Poland †	545.6	100	74.7	13.7	30.2	5.5	39.0	7.1	21.6	4.0	106.4	19.5	128.0	23.5	66.2	12.1	20.4	3.7	67.9	12.4	119.1	21.8	0.2	0
Portugal ‡ + &	2021.8	100	173.8	8.6			86.8	4.3					348.6	17.2	162.8	8.1			292.5	14.5	244.6	12.1	4.5	0.2
Slovakia †	711.8	100	100.0	14.0	87.0	12.2	35.3	5.0					195.9	27.5	41.1	5.8	19.7	2.8	167.5	23.5	65.3	9.2	0	0
Spain ¶ §	1405.9	100	287.8	20.5									228.4	16.2	147.2	10.5			683.4	48.6			59.0	4.2
Türkiye	1048.7	100	9.9	0.9	2.6	0.3	8.7	0.8	11.4	1.1	60.4	5.8	71.8	6.8	65.4	6.2	2.0	0.2	31.7	3.0	23.1	2.2	833.4	79.5

Abbreviations used: GN: glomerulonephritis/sclerosis; PN: pyelonephritis; PKD: polycystic kidneys, adult type; DM: diabetes mellitus; HT: hypertension; RVD: renal vascular disease; Misc: miscellaneous; Unkn: unknown
Categories may not add up due to rounding
When cells are left empty, the data are unavailable
* Patients younger than 18 years of age are not reported
† Data include dialysis patients only
$ Hypertension is not reported separately, but is included in renal vascular disease
| | Total DM includes: 212 patients with type 1 DM, 1100 patients with type 2 DM and 136 patients with unspecified type of DM
¤ Excluding data from Trentino; transplant patients are not reported for Lazio
‡ Data on primary renal disease are available for dialysis patients only (N=13976, 65.0% of total)
+ Renal vascular disease is not reported separately, but is included in miscellaneous
& Pyelonephritis is not reported separately, but is included in miscellaneous
¶ Renal vascular disease is not reported separately, but is included in hypertension
§ Miscellaneous renal disorders include: Tubulointerstitial disease (n=7414); Other systemic diseases affecting the kidney (n=1600), Familial/Hereditary nephropathies (n=9229), and Miscellaneous renal disorders (n=14620)

Table C.4.6

Prevalent counts by treatment modality

prevalent patients on December 31

	All	Haemodialysis						Peritoneal dialysis				Kidney transplant				Missing
		HD hospital/ centre	HD home	HD type Unkn	HF	HDF	Total HD/ HF/HDF	APD	CAPD	PD type Unkn	Total PD	Living donor	Deceased donor	Tx type Unkn	Total Tx	
	N	N	N	N	N	N	N	N	N	N	N	N	N	N	N	N
Albania	1724	1397	0	0	0	0	1397	0	27	0	27	264	30	6	300	0
Belarus *	4374	1395	0	0	1	488	1884	9	106	0	115	94	2281	0	2375	0
Croatia †	2159	1395	0	0	0	641	2036	0	100	3	103					20
Cyprus																
Czech Republic † &	5937	874	63	0	0	4810	5747	94	68	28	190					0
Finland	5292	729	101	0	0	697	1527	168	111	0	279			3485	3485	1
Hungary	8862	4488	0	13	3	742	5246			688	688	122	484	2314	2920	8
Ireland	5257		57	2197			2254			248	248			2755	2755	
Israel †	7087	5937	0	0	0	558	6495	320	269	3	592					0
Italy (10 of 20 regions)	34633	13287	59	1	1092	5358	19797	1232	1079	41	2352	1146	10390	948	12484	0
Italy (5 extra regions) ¤	19135	6237	54	609	68	5199	12167	680	601	30	1311	357	1555	3745	5657	0
Italy (15 of 20 regions) ¤	53768	19524	113	610	1160	10557	31964	1912	1680	71	3663	1503	11945	4693	18141	0
Kosovo *	1063	904	0	0	0	0	904	0	0	0	0	159	0	0	159	0
Latvia	1145	474	0	0	0	0	474	50	55	0	105	76	486	4	566	0
Lithuania	2466	1265	0	0	0	0	1265	27	22	0	49			1152	1152	0
North Macedonia	1779	1507	0	0	0	0	1507	7	7	0	14	214	44	0	258	0
Poland †	20536			19770			19770			766	766					0
Portugal	21511			13087			13087			889	889	853	6682	0	7535	0
Slovakia †	3183	291	0	0	0	2829	3120	37	23	0	60					3
Spain	67604	26207	435	0	0	0	26642	698	2539	0	3237			37725	37725	0
Türkiye	89527	62004	1331	39	3	77	63454	1396	2282	0	3678			22395	22395	0

Abbreviations used: HD: haemodialysis; Unkn: unknown; HF: haemofiltration; HDF: haemodiafiltration; APD: automated peritoneal dialysis; CAPD: continuous ambulatory peritoneal dialysis; PD: peritoneal dialysis; Tx: transplant
When cells are left empty, the data are unavailable
* Patients younger than 18 years of age are not reported
† Data include dialysis patients only
& Additionally there are 6021 prevalent kidney transplant recipients
¤ Excluding data from Trentino; transplant patients are not reported for Lazio

Table C.4.7

Prevalence per million population by treatment modality, unadjusted

prevalent patients on December 31

	All	Haemodialysis						Peritoneal dialysis				Kidney transplant				Missing
		HD hospital/ centre	HD home	HD type Unkn	HF	HDF	Total HD/ HF/HDF	APD	CAPD	PD type Unkn	Total PD	Living donor	Deceased donor	Tx type Unkn	Total Tx	
	Pmp	Pmp	Pmp	Pmp	Pmp	Pmp	Pmp	Pmp	Pmp	Pmp	Pmp	Pmp	Pmp	Pmp	Pmp	Pmp
Albania	630.5	510.9	0	0	0	0	510.9	0	9.9	0	9.9	96.6	11.0	2.2	109.7	0
Belarus *	518.0	165.2	0	0	0.1	57.8	223.1	1.1	12.6	0	13.6	11.1	270.1	0	281.3	0
Croatia †	658.1	425.2	0	0	0	195.4	620.6	0	30.5	0.9	31.4					6.1
Cyprus																
Czech Republic †	548.3	80.7	5.8	0	0	444.2	530.8	8.7	6.3	2.6	17.5					0
Finland	963.6	132.7	18.4	0	0	126.9	278.1	30.6	20.2	0	50.8			634.6	634.6	0.2
Hungary	923.1	467.5	0	1.4	0.3	77.3	546.5			71.7	71.7	12.7	50.4	241.0	304.2	0.8
Ireland	1020.9		11.1	426.7			437.7			48.2	48.2			535.0	535.0	
Israel †	735.7	616.3	0	0	0	57.9	674.2	33.2	27.9	0.3	61.5					0
Italy (10 of 20 regions)	1210.4	464.4	2.1	0	38.2	187.3	691.9	43.1	37.7	1.4	82.2	40.1	363.1	33.1	436.3	0
Italy (5 extra regions) ¤	1121.7	365.6	3.2	35.7	4.0	304.8	713.2	39.9	35.2	1.8	76.8	20.9	91.2	219.5	331.6	0
Italy (15 of 20 regions) ¤	1182.3	429.3	2.5	13.4	25.5	232.1	702.9	42.0	36.9	1.6	80.5	33.1	262.7	103.2	398.9	0
Kosovo *	629.9	535.7	0	0	0	0	535.7	0	0	0	0	94.2	0	0	94.2	0
Latvia	660.9	273.6	0	0	0	0	273.6	28.9	31.7	0	60.6	43.9	280.5	2.3	326.7	0
Lithuania	863.1	442.7	0	0	0	0	442.7	9.4	7.7	0	17.1			403.2	403.2	0
North Macedonia	974.1	825.2	0	0	0	0	825.2	3.8	3.8	0	7.7	117.2	24.1	0	141.3	0
Poland †	545.6			525.3			525.3			20.4	20.4					0
Portugal	2021.8			1230.0			1230.0			83.6	83.6	80.2	628.0	0	708.2	0
Slovakia †	711.8	65.1	0	0	0	632.7	697.7	8.3	5.1	0	13.4					0.7
Spain	1405.9	545.0	9.0	0	0	0	554.1	14.5	52.8	0	67.3			784.5	784.5	0
Türkiye	1048.7	726.3	15.6	0.5	0	0.9	743.3	16.4	26.7	0	43.1			262.3	262.3	0

Abbreviations used: HD: haemodialysis; Unkn: unknown; HF: haemofiltration; HDF: haemodiafiltration; APD: automated peritoneal dialysis; CAPD: continuous ambulatory peritoneal dialysis; PD: peritoneal dialysis; Tx: transplant
Categories may not add up due to rounding
When cells are left empty, the data are unavailable
* Patients younger than 18 years of age are not reported
† Data include dialysis patients only
¤ Excluding data from Trentino; transplant patients are not reported for Lazio

Table C.4.8

Treatment modality distribution, unadjusted

prevalent patients on December 31

	All	Haemodialysis						Peritoneal dialysis				Kidney transplant				Missing
		HD hospital/ centre	HD home	HD type Unkn	HF	HDF	Total HD/ HF/HDF	APD	CAPD	PD type Unkn	Total PD	Living donor	Deceased donor	Tx type Unkn	Total Tx	
	%	%	%	%	%	%	%	%	%	%	%	%	%	%	%	%
Albania	100	81.0	0	0	0	0	81.0	0	1.6	0	1.6	15.3	1.7	0.3	17.4	0
Belarus *	100	31.9	0	0	0	11.2	43.1	0.2	2.4	0	2.6	2.1	52.1	0	54.3	0
Croatia †	100	64.6	0	0	0	29.7	94.3	0	4.6	0.1	4.8					0.9
Cyprus																
Czech Republic †	100	14.7	1.1	0	0	81.0	96.8	1.6	1.1	0.5	3.2					0
Finland	100	13.8	1.9	0	0	13.2	28.9	3.2	2.1	0	5.3			65.9	65.9	0
Hungary	100	50.6	0	0.1	0	8.4	59.2			7.8	7.8	1.4	5.5	26.1	32.9	0.1
Ireland	100		1.1	41.8			42.9			4.7	4.7			52.4	52.4	
Israel†	100	83.8	0	0	0	7.9	91.6	4.5	3.8	0	8.4					0
Italy (10 of 20 regions)	100	38.4	0.2	0	3.2	15.5	57.2	3.6	3.1	0.1	6.8	3.3	30.0	2.7	36.0	0
Italy (5 extra regions) ¤	100	32.6	0.3	3.2	0.4	27.2	63.6	3.6	3.1	0.2	6.9	1.9	8.1	19.6	29.6	0
Italy (15 of 20 regions) ¤	100	36.3	0.2	1.1	2.2	19.6	59.4	3.6	3.1	0.1	6.8	2.8	22.2	8.7	33.7	0
Kosovo *	100	85.0	0	0	0	0	85.0	0	0	0	0	15.0	0	0	15.0	0
Latvia	100	41.4	0	0	0	0	41.4	4.4	4.8	0	9.2	6.6	42.4	0.3	49.4	0
Lithuania	100	51.3	0	0	0	0	51.3	1.1	0.9	0	2.0			46.7	46.7	0
North Macedonia	100	84.7	0	0	0	0	84.7	0.4	0.4	0	0.8	12.0	2.5	0	14.5	0
Poland †	100			96.3			96.3			3.7	3.7					0
Portugal	100			60.8			60.8			4.1	4.1	4.0	31.1	0	35.0	0
Slovakia †	100	9.1	0	0	0	88.9	98.0	1.2	0.7	0	1.9					0.1
Spain	100	38.8	0.6	0	0	0	39.4	1.0	3.8	0	4.8			55.8	55.8	0
Türkiye	100	69.3	1.5	0	0	0.1	70.9	1.6	2.5	0	4.1			25.0	25.0	0

Abbreviations used: HD: haemodialysis; Unkn: unknown; HF: haemofiltration; HDF: haemodiafiltration; APD: automated peritoneal dialysis; CAPD: continuous ambulatory peritoneal dialysis; PD: peritoneal dialysis; Tx: transplant
Categories may not add up due to rounding
When cells are left empty, the data are unavailable
* Patients younger than 18 years of age are not reported
† Data include dialysis patients only
¤ Excluding data from Trentino; transplant patients are not reported for Lazio

Table C.5.1

Kidney transplant counts and percentages by donor type

	All		Living donor								Deceased donor		Donor type Unkn	
			Related		Unrelated		Type Unkn		All					
	N	%	N	%	N	%	N	%	N	%	N	%	N	%
Albania	26	100	26	100	0	0	0	0	26	100	0	0	0	0
Belarus *	322	100	0	0	0	0	2	0.6	2	0.6	320	99.4	0	0
Croatia	141	100					11	7.8	11	7.8	130	92.2	0	0
Cyprus	35	100	17	48.6	0	0	0	0	17	48.6	18	51.4	0	0
Czech Republic	488	100					36	7.4	36	7.4	452	92.6	0	0
Finland	319	100	22	6.9	21	6.6	0	0	43	13.5	276	86.5	0	0
Hungary	265	100					45	17.0	45	17.0	220	83.0	0	0
Ireland	189	100					30	15.9	30	15.9	159	84.1	0	0
Israel	492	100					330	67.1	330	67.1	155	31.5	7	1.4
Italy (10 of 20 regions)	823	100	25	3.0	10	1.2	56	6.8	91	11.1	726	88.2	6	0.7
Italy (5 extra regions) ¤	393	100	18	4.6	8	2.0	0	0	26	6.6	174	44.3	193	49.1
Italy (15 of 20 regions) ¤	1216	100	43	3.5	18	1.5	56	4.6	117	9.6	900	74.0	199	16.4
Kosovo	11	100	5	45.5	6	54.5	0	0	11	100	0	0	0	0
Latvia	46	100	2	4.3	1	2.2	0	0	3	6.5	43	93.5	0	0
Lithuania	102	100					6	5.9	6	5.9	96	94.1	0	0
North Macedonia	22	100	16	72.7	0	0	0	0	16	72.7	6	27.3	0	0
Poland	1075	100					78	7.3	78	7.3	997	92.7	0	0
Portugal	547	100					71	13.0	71	13.0	476	87.0	0	0
Slovakia +	128	100	13	10.2	3	2.3	0	0	16	12.5	112	87.5	0	0
Spain ‡	3690	100	217	5.9	195	5.3	23	0.6	435	11.8	3255	88.2	0	0
Türkiye	3452	100	2905	84.2	246	7.1	0	0	3151	91.3	301	8.7	0	0

Categories may not add up due to rounding
When cells are left empty, the data are unavailable
* Patients younger than 18 years of age are not reported
¤ Excluding data from Lazio
+ Data from: National Transplant Organization (NTO)
‡ Data from: Organización Nacional de Trasplantes (ONT)

Table C.5.2

Kidney transplants per million population by donor type, unadjusted

	All	Living donor				Deceased donor	Donor type Unkn
		Related	Unrelated	Type Unkn	All		
	Pmp	Pmp	Pmp	Pmp	Pmp	Pmp	Pmp
Albania	9.5	9.5	0	0	9.5	0	0
Belarus *	38.1	0	0	0.2	0.2	37.9	0
Croatia	43.0			3.4	3.4	39.6	0
Cyprus	36.9	17.9	0	0	17.9	19.0	0
Czech Republic	45.1			3.3	3.3	41.7	0
Finland	58.1	4.0	3.8	0	7.8	50.3	0
Hungary	27.6			4.7	4.7	22.9	0
Ireland	36.7			5.8	5.8	30.9	0
Israel	51.1			34.3	34.3	16.1	0.7
Italy (10 of 20 regions)	28.8	0.9	0.3	2.0	3.2	25.4	0.2
Italy (5 extra regions) ¤	33.1	1.5	0.7	0	2.2	14.6	16.2
Italy (15 of 20 regions) ¤	30.2	1.1	0.4	1.4	2.9	22.3	4.9
Kosovo	6.5	3.0	3.6	0	6.5	0	0
Latvia	26.6	1.2	0.6	0	1.7	24.8	0
Lithuania	35.7			2.1	2.1	33.6	0
North Macedonia	12.0	8.8	0	0	8.8	3.3	0
Poland	28.6			2.1	2.1	26.5	0
Portugal	51.4			6.7	6.7	44.7	0
Slovakia +	28.6	2.9	0.7	0	3.6	25.0	0
Spain ‡	76.7	4.5	4.1	0.5	9.0	67.7	0
Türkiye	40.4	34.0	2.9	0	36.9	3.5	0

Categories may not add up due to rounding
When cells are left empty, the data are unavailable
* Patients younger than 18 years of age are not reported
¤ Excluding data from Lazio
+ Data from: National Transplant Organization (NTO)
‡ Data from: Organización Nacional de Trasplantes (ONT)

Section D

Paediatric data reference tables

The incidence and prevalence of KRT in paediatric patients are based on data from 26 registries from 15 countries that provided individual patient data on children to the ERA Registry, including Austria, Bosnia and Herzegovina, Denmark, Estonia, France, Greece, Iceland, Netherlands, Norway, Romania, Serbia, Spain (Andalusia), Spain (Aragon), Spain (Basque country), Spain (Canary Islands), Spain (Catalonia), Spain (Community of Madrid), Spain (Extremadura), Spain (Galicia), Spain (La Rioja), Spain (Murcia), Spain (Valencian Region), Sweden, Switzerland, and United Kingdom (England, Northern Ireland and Wales, and Scotland). In the analyses the data collected between 2018 and 2023 were used. Since not all countries provided data or had full coverage over the entire period and to allow for comparison across years, data are shown both including and excluding the data from Austria, Estonia, Netherlands, Romania, Serbia, Spain (Community of Madrid), Spain (La Rioja), and Switzerland. The methods applied to the paediatric patient data were similar to those applied to the adult patient data and are described in the Methods section. However, as not all regions in Spain had specialized paediatric centres, non-residents were also included in the incidence and prevalence.

D1 GENERAL POPULATION AGE DISTRIBUTION

Table D.1.1

Population covered (in thousands)

by age and cohort

Cohort	All	0-4	5-9	10-14	15-19
	N	N	N	N	N
2018	46064	10982	11929	11741	11411
2019	46138	10841	11867	11942	11488
2020	46102	10640	11775	12105	11582
2021	45582	10287	11449	12110	11736
2022	45565	10154	11338	12171	11902
2023	45581	10023	11254	12180	12124
2018 *	59858	14314	15339	15228	14977
2019 *	59996	14193	15268	15466	15069
2020 *	59819	13953	15128	15606	15131
2021 *	59446	13600	14855	15672	15319
2022 *	59431	13413	14773	15743	15502
2023 *	59530	13245	14726	15769	15789
2018-2023 *	358080	82718	90090	93484	91788

* Additionally including data from Austria (coverage 99% in 2020-2021, 98% in 2022-2023, and 100% for all other years), Romania (coverage 98% in 2018, 99% in 2019, 95% in 2020, and 100% in 2021-2023), Serbia (90% in 2018-2020, 95% in 2021-2022, and 97% in 2023), Spain (Community of Madrid) (coverage 95% in 2022), Spain (la Rioja) since 2019, Switzerland (coverage 97% in 2021, 99% in 2022, 95% in 2023, and 100% in all other years), Netherlands (coverage 93% for incidence in 2018-2019, 96% for prevalence in 2018-2023 and 94% for incidence in 2020, 92% for incidence in 2021-2022, and 93% for incidence in 2023), and Estonia (coverage 95% in 2023)

Table D.2.1

Incident counts by age and cohort

at day 1

Cohort	All	0-4	5-9	10-14	15-19
	N	N	N	N	N
2018-2019	822	174	112	196	340
2020-2021	848	153	122	204	369
2022-2023	809	129	113	218	349
2018-2019 *	1028	201	141	240	446
2020-2021 *	1038	183	159	245	451
2022-2023 *	1040	170	153	278	439
2018-2023 *	3106	554	453	763	1336

* Additionally including data from Austria (coverage 99% in 2020-2021, 98% in 2022-2023, and 100% for all other years), Romania (coverage 98% in 2018, 99% in 2019, 95% in 2020, and 100% in 2021-2022), Serbia (90% in 2018-2020, 95% in 2021-2022, and 97% in 2023), Spain (Community of Madrid) (coverage 95% in 2022), Spain (la Rioja) since 2019, Switzerland (coverage 97% in 2021, 99% in 2022, 95% in 2023, and 100% in all other years), Netherlands (coverage 93% in 2018-2019, 94% in 2020, 92% in 2021-2022, and 93% in 2023), and Estonia (coverage 95% in 2023)

Table D.2.2

Incident counts by age, treatment modality, and cohort

at day 1

Cohort	All			0-4			5-9			10-14			15-19		
	HD	PD	Tx	HD	PD	Tx	HD	PD	Tx	HD	PD	Tx	HD	PD	Tx
	N	N	N	N	N	N	N	N	N	N	N	N	N	N	N
2018-2019	368	260	183	51	96	23	41	32	36	95	60	39	181	72	85
2020-2021	402	267	175	46	90	17	53	34	34	77	69	57	226	74	67
2022-2023	371	242	196	44	66	19	34	42	37	100	49	69	193	85	71
2018-2019 *	500	292	225	59	111	27	60	36	42	128	65	45	253	80	111
2020-2021 *	504	321	208	55	107	21	69	51	38	98	79	67	282	84	82
2022-2023 *	490	291	257	55	83	32	52	52	49	136	58	84	247	98	92
2018-2023 *	1494	904	690	169	301	80	181	139	129	362	202	196	782	262	285

For 18 patients information on treatment modality at start was unavailable
* Additionally including data from Austria (coverage 99% in 2020-2021, 98% in 2022-2023, and 100% for all other years), Romania (coverage 98% in 2018, 99% in 2019, 95% in 2020, and 100% in 2021-2022), Serbia (90% in 2018-2020, 95% in 2021-2022, and 97% in 2023), Spain (Community of Madrid) (coverage 95% in 2022), Spain (la Rioja) since 2019, Switzerland (coverage 97% in 2021, 99% in 2022, 95% in 2023, and 100% in all other years), Netherlands (coverage 93% in 2018-2019, 94% in 2020, 92% in 2021-2022, and 93% in 2023), and Estonia (coverage 95% in 2023)

Table D.2.3

Incident counts by age and primary renal disease

between 2018 and 2023, at day 1

	All	0-4	5-9	10-14	15-19
	N	N	N	N	N
CAKUT	900	189	161	243	307
Glomerulonephritis	518	87	54	103	274
Cystic kidney disease	333	73	63	104	93
Hereditary Nephropathy	247	47	40	48	112
Ischaemic Renal Failure	57	16	5	14	22
HUS	90	20	25	18	27
Metabolic disorders	72	13	11	21	27
Vasculitis	100	1	10	16	73
Miscellaneous	230	45	17	46	122
Missing, unknown	559	63	67	150	279

Abbreviations used: CAKUT: congenital anomalies of the kidney and urinary tract; HUS: haemolytic uraemic syndrome
Additionally including data from Austria (coverage 99% in 2020-2021, 98% in 2022-2023, and 100% for all other years), Romania (coverage 98% in 2018, 99% in 2019, 95% in 2020, and 100% in 2021-2022), Serbia (90% in 2018-2020, 95% in 2021-2022, and 97% in 2023), Spain (Community of Madrid) (coverage 95% in 2022), Spain (la Rioja) since 2019, Switzerland (coverage 97% in 2021, 99% in 2022, 95% in 2023, and 100% in all other years), Netherlands (coverage 93% in 2018-2019, 94% in 2020, 92% in 2021-2022, and 93% in 2023), and Estonia (coverage 95% in 2023)

Table D.2.4

Incidence per million age-related population by age and cohort
at day 1

Cohort	All	0-4	5-9	10-14	15-19
	Pmarp	Pmarp	Pmarp	Pmarp	Pmarp
2018-2019	8.9	8.0	4.7	8.3	14.8
2020-2021	9.2	7.3	5.3	8.4	15.8
2022-2023	8.9	6.4	5.0	9.0	14.5
2018-2019 *	8.6	7.1	4.6	7.8	14.8
2020-2021 *	8.7	6.6	5.3	7.8	14.8
2022-2023 *	8.7	6.4	5.2	8.8	14.0
2018-2023 *	8.7	6.7	5.0	8.2	14.6

* Additionally including data from Austria (coverage 99% in 2020-2021, 98% in 2022-2023, and 100% for all other years), Romania (coverage 98% in 2018, 99% in 2019, 95% in 2020, and 100% in 2021-2022), Serbia (90% in 2018-2020, 95% in 2021-2022, and 97% in 2023), Spain (Community of Madrid) (coverage 95% in 2022), Spain (la Rioja) since 2019, Switzerland (coverage 97% in 2021, 99% in 2022, 95% in 2023, and 100% in all other years), Netherlands (coverage 93% in 2018-2019, 94% in 2020, 92% in 2021-2022, and 93% in 2023), and Estonia (coverage 95% in 2023)

Table D.2.5

Incidence per million age-related population by age, treatment modality, and cohort
at day 1

Cohort	All			0-4			5-9			10-14			15-19		
	HD	PD	Tx	HD	PD	Tx	HD	PD	Tx	HD	PD	Tx	HD	PD	Tx
	Pmarp	Pmarp	Pmarp	Pmarp	Pmarp	Pmarp	Pmarp	Pmarp	Pmarp	Pmarp	Pmarp	Pmarp	Pmarp	Pmarp	Pmarp
2018-2019	4.0	2.8	2.0	2.3	4.4	1.1	1.7	1.3	1.5	4.0	2.5	1.6	7.9	3.1	3.7
2020-2021	4.4	2.9	1.9	2.2	4.3	0.8	2.3	1.5	1.5	3.2	2.8	2.4	9.7	3.2	2.9
2022-2023	4.1	2.7	2.2	2.2	3.3	0.9	1.5	1.9	1.6	4.1	2.0	2.8	8.0	3.5	3.0
2018-2019 *	4.2	2.4	1.9	2.1	3.9	0.9	2.0	1.2	1.4	4.2	2.1	1.5	8.4	2.7	3.7
2020-2021 *	4.2	2.7	1.7	2.0	3.9	0.8	2.3	1.7	1.3	3.1	2.5	2.1	9.3	2.8	2.7
2022-2023 *	4.1	2.4	2.2	2.1	3.1	1.2	1.8	1.8	1.7	4.3	1.8	2.7	7.9	3.1	2.9
2018-2023 *	4.2	2.5	1.9	2.0	3.6	1.0	2.0	1.5	1.4	3.9	2.2	2.1	8.5	2.9	3.1

For 18 patients information on treatment modality at start was unavailable
* Additionally including data from Austria (coverage 99% in 2020-2021, 98% in 2022-2023, and 100% for all other years), Romania (coverage 98% in 2018, 99% in 2019, 95% in 2020, and 100% in 2021-2022), Serbia (90% in 2018-2020, 95% in 2021-2022, and 97% in 2023), Spain (Community of Madrid) (coverage 95% in 2022), Spain (la Rioja) since 2019, Switzerland (coverage 97% in 2021, 99% in 2022, 95% in 2023, and 100% in all other years), Netherlands (coverage 93% in 2018-2019, 94% in 2020, 92% in 2021-2022, and 93% in 2023), and Estonia (coverage 95% in 2023)

Table D.2.6

Incidence per million age-related population by age and primary renal disease
between 2018 and 2023, at day 1

	All	0-4	5-9	10-14	15-19
	Pmarp	Pmarp	Pmarp	Pmarp	Pmarp
CAKUT	2.5	2.3	1.8	2.6	3.3
Glomerulonephritis	1.4	1.1	0.6	1.1	3.0
Cystic kidney disease	0.9	0.9	0.7	1.1	1.0
Hereditary Nephropathy	0.7	0.6	0.4	0.5	1.2
Ischaemic Renal Failure	0.2	0.2	0.1	0.1	0.2
HUS	0.3	0.2	0.3	0.2	0.3
Metabolic disorders	0.2	0.2	0.1	0.2	0.3
Vasculitis	0.3	0.0	0.1	0.2	0.8
Miscellaneous	0.6	0.5	0.2	0.5	1.3
Missing, unknown	1.6	0.8	0.7	1.6	3.0

Abbreviations used: CAKUT: congenital anomalies of the kidney and urinary tract; HUS: haemolytic uraemic syndrome
Additionally including data from Austria (coverage 99% in 2020-2021, 98% in 2022-2023, and 100% for all other years), Romania (coverage 98% in 2018, 99% in 2019, 95% in 2020, and 100% in 2021-2022), Serbia (90% in 2018-2020, 95% in 2021-2022, and 97% in 2023), Spain (Community of Madrid) (coverage 95% in 2022), Spain (la Rioja) since 2019, Switzerland (coverage 97% in 2021, 99% in 2022, 95% in 2023, and 100% in all other years), Netherlands (coverage 93% in 2018-2019, 94% in 2020, 92% in 2021-2022, and 93% in 2023), and Estonia (coverage 95% in 2023)

Table D.3.1

Prevalent counts by age and cohort

prevalent patients on December 31

Cohort	All	0-4	5-9	10-14	15-19
	N	N	N	N	N
2018	3191	266	558	903	1464
2019	3205	243	565	911	1486
2020	3241	221	556	941	1523
2021	3245	221	525	968	1531
2022	3245	198	527	984	1536
2023	3101	174	499	932	1496
2018 *	3793	310	657	1064	1762
2019 *	3797	278	656	1096	1767
2020 *	3844	254	651	1120	1819
2021 *	3852	262	615	1164	1811
2022 *	3849	238	613	1182	1816
2023 *	3823	222	623	1140	1838

* Additionally including data from Austria (coverage 99% in 2020-2021, 98% in 2022-2023, and 100% for all other years), Romania (coverage 98% in 2018, 99% in 2019, 95% in 2020, and 100% in 2021-2023), Serbia (90% in 2018-2020, 95% in 2021-2022, and 97% in 2023), Spain (Community of Madrid) (coverage 95% in 2022), Spain (la Rioja) since 2019, Switzerland (coverage 97% in 2021, 99% in 2022, 95% in 2023, and 100% in all other years), Netherlands (coverage 96% in 2018-2023), and Estonia (coverage 95% in 2023)

Table D.3.2

Prevalent counts by age, treatment modality, and cohort

prevalent patients on December 31

Cohort	All			0-4			5-9			10-14			15-19		
	HD	PD	Tx	HD	PD	Tx	HD	PD	Tx	HD	PD	Tx	HD	PD	Tx
	N	N	N	N	N	N	N	N	N	N	N	N	N	N	N
2018	412	270	2457	55	103	104	58	41	456	96	58	738	203	68	1159
2019	425	262	2477	52	87	102	65	48	448	103	59	740	205	68	1187
2020	452	264	2484	55	76	88	62	44	446	98	62	766	237	82	1184
2021	444	263	2498	47	81	91	68	37	413	93	63	803	236	82	1191
2022	451	241	2528	51	64	82	59	36	429	97	61	818	244	80	1199
2023	432	215	2438	42	59	73	49	41	406	92	43	793	249	72	1166
2018 *	547	317	2876	62	115	129	74	51	529	132	68	853	279	83	1365
2019 *	563	304	2888	61	99	116	81	52	519	145	73	869	276	80	1384
2020 *	590	313	2899	61	88	103	79	55	513	134	73	898	316	97	1385
2021 *	575	327	2908	59	96	105	85	54	469	124	75	956	307	102	1378
2022 *	583	301	2939	63	76	98	75	47	488	125	83	966	320	95	1387
2023 *	574	278	2955	53	77	92	71	55	494	125	58	953	325	88	1416

* Additionally including data from Austria (coverage 99% in 2020-2021, 98% in 2022-2023, and 100% for all other years), Romania (coverage 98% in 2018, 99% in 2019, 95% in 2020, and 100% in 2021-2023), Serbia (90% in 2018-2020, 95% in 2021-2022, and 97% in 2023), Spain (Community of Madrid) (coverage 95% in 2022), Spain (la Rioja) since 2019, Switzerland (coverage 97% in 2021, 99% in 2022, 95% in 2023, and 100% in all other years), Netherlands (coverage 96% in 2018-2023), and Estonia (coverage 95% in 2023)

Table D.3.3

Prevalent counts by age and primary renal disease

prevalent patients on December 31, 2023

	All	0-4	5-9	10-14	15-19
	N	N	N	N	N
CAKUT	1426	77	246	452	651
Glomerulonephritis	517	23	95	135	264
Cystic kidney disease	449	26	75	136	212
Hereditary Nephropathy	318	17	64	106	131
Ischaemic Renal Failure	80	8	12	20	40
HUS	130	3	28	36	63
Metabolic disorders	101	12	9	27	53
Vasculitis	46	0	2	11	33
Miscellaneous	263	23	44	64	132
Missing, unknown	493	33	48	153	259

Abbreviations used: CAKUT: congenital anomalies of the kidney and urinary tract; HUS: haemolytic uraemic syndrome
Including data from all countries, also those with incomplete coverage

Table D.3.4

Prevalence per million age-related population by age and cohort

prevalent patients on December 31

Cohort	All	0-4	5-9	10-14	15-19
	Pmarp	Pmarp	Pmarp	Pmarp	Pmarp
2018	69.3	24.2	46.8	76.9	128.3
2019	69.5	22.4	47.6	76.3	129.3
2020	70.3	20.8	47.2	77.7	131.5
2021	71.2	21.5	45.9	79.9	130.5
2022	71.2	19.5	46.5	80.8	129.1
2023	68.0	17.4	44.3	76.5	123.4
2018 *	63.2	21.6	42.8	69.7	117.4
2019 *	63.2	19.6	42.9	70.7	117.0
2020 *	64.2	18.2	43.0	71.7	120.0
2021 *	64.6	19.2	41.3	74.1	117.9
2022 *	64.6	17.7	41.4	74.9	116.8
2023 *	64.1	16.7	42.2	72.2	116.2

* Additionally including data from Austria (coverage 99% in 2020-2021, 98% in 2022-2023, and 100% for all other years), Romania (coverage 98% in 2018, 99% in 2019, 95% in 2020, and 100% in 2021-2023), Serbia (90% in 2018-2020, 95% in 2021-2022, and 97% in 2023), Spain (Community of Madrid) (coverage 95% in 2022), Spain (la Rioja) since 2019, Switzerland (coverage 97% in 2021, 99% in 2022, 95% in 2023, and 100% in all other years), Netherlands (coverage 96% in 2018-2023), and Estonia (coverage 95% in 2023)

Table D.3.5

Prevalence per million age-related population by age, treatment modality, and cohort

prevalent patients on December 31

Cohort	All			0-4			5-9			10-14			15-19		
	HD	PD	Tx	HD	PD	Tx	HD	PD	Tx	HD	PD	Tx	HD	PD	Tx
	Pmarp	Pmarp	Pmarp	Pmarp	Pmarp	Pmarp	Pmarp	Pmarp	Pmarp	Pmarp	Pmarp	Pmarp	Pmarp	Pmarp	Pmarp
2018	8.9	5.9	53.3	5.0	9.4	9.5	4.9	3.4	38.2	8.2	4.9	62.9	17.8	6.0	101.6
2019	9.2	5.7	53.7	4.8	8.0	9.4	5.5	4.0	37.8	8.6	4.9	62.0	17.8	5.9	103.3
2020	9.8	5.7	53.9	5.2	7.1	8.3	5.3	3.7	37.9	8.1	5.1	63.3	20.5	7.1	102.2
2021	9.7	5.8	54.8	4.6	7.9	8.8	5.9	3.2	36.1	7.7	5.2	66.3	20.1	7.0	101.5
2022	9.9	5.3	55.5	5.0	6.3	8.1	5.2	3.2	37.8	8.0	5.0	67.2	20.5	6.7	100.7
2023	9.5	4.7	53.5	4.2	5.9	7.3	4.4	3.6	36.1	7.6	3.5	65.1	20.5	5.9	96.2
2018 *	9.1	5.3	48.0	4.3	8.0	9.0	4.8	3.3	34.4	8.7	4.5	55.9	18.6	5.5	90.9
2019 *	9.4	5.1	48.0	4.3	7.0	8.2	5.3	3.4	33.9	9.4	4.7	56.1	18.3	5.3	91.7
2020 *	9.9	5.2	48.4	4.4	6.3	7.4	5.2	3.6	33.9	8.6	4.7	57.5	20.9	6.4	91.4
2021 *	9.6	5.5	48.8	4.3	7.0	7.7	5.7	3.6	31.5	7.9	4.8	60.9	20.0	6.6	89.7
2022 *	9.8	5.1	49.3	4.7	5.7	7.3	5.1	3.2	33.0	7.9	5.3	61.2	20.6	6.1	89.2
2023 *	9.6	4.7	49.5	4.0	5.8	6.9	4.8	3.7	33.5	7.9	3.7	60.3	20.5	5.6	89.5

* Additionally including data from Austria (coverage 99% in 2020-2021, 98% in 2022-2023, and 100% for all other years), Romania (coverage 98% in 2018, 99% in 2019, 95% in 2020, and 100% in 2021-2023), Serbia (90% in 2018-2020, 95% in 2021-2022, and 97% in 2023), Spain (Community of Madrid) (coverage 95% in 2022), Spain (la Rioja) since 2019, Switzerland (coverage 97% in 2021, 99% in 2022, 95% in 2023, and 100% in all other years), Netherlands (coverage 96% in 2018-2023), and Estonia (coverage 95% in 2023)

Table D.3.6

Prevalence per million age-related population by age and primary renal disease

prevalent patients on December 31, 2023

	All	0-4	5-9	10-14	15-19
	Pmarp	Pmarp	Pmarp	Pmarp	Pmarp
CAKUT	23.9	5.8	16.7	28.6	41.2
Glomerulonephritis	8.7	1.7	6.4	8.5	16.7
Cystic kidney disease	7.5	2.0	5.1	8.6	13.4
Hereditary Nephropathy	5.3	1.3	4.3	6.7	8.3
Ischaemic Renal Failure	1.3	0.6	0.8	1.3	2.5
HUS	2.2	0.2	1.9	2.3	4.0
Metabolic disorders	1.7	0.9	0.6	1.7	3.4
Vasculitis	0.8	0.0	0.1	0.7	2.1
Miscellaneous	4.4	1.7	3.0	4.1	8.3
Missing, unknown	8.3	2.5	3.3	9.7	16.4

Abbreviations used: CAKUT: congenital anomalies of the kidney and urinary tract; HUS: haemolytic uraemic syndrome
Including data from all countries, also those with incomplete coverage

Methods

The ERA Registry is located at the Department of Medical Informatics in the Amsterdam University Medical Centres, location Academic Medical Centre in Amsterdam, Netherlands. This department hosts several clinical registries which share an ISO 9001:2015 certified quality management system with standard operating procedures for data collection, data storage, data analysis, and the production of the annual report. In addition, the clinical registries share an ISO 27001 certified information security management system, including procedures to ensure a lasting level of information security and data protection.

Data collection and preparation

Individual patient data

General Data Protection Regulation (GDPR)

All registries participating with individual patient data in this annual report have signed a Data Transfer Agreement with the AMC Medical Research B.V., where the ERA Registry is located.

Data collection

On an annual basis data sets containing individual patient data are uploaded to the ERA Registry office in Amsterdam. Supported file formats for data delivery include Microsoft Access, Microsoft Excel, SPSS, SAS, and delimited text files. Collaborating registries upload their data via a secure data upload portal.

Variables

The ERA Registry asks national and regional registries for the data shown in Table 1.

Table 1
Variables collected for individual patients

Patient data	Transaction data
Patient identifier	Patient identifier
Country of registry	Date of event
Month and year of birth	Type of event / treatment
Sex	Cause of death
Primary Renal Disease	Treatment centre
Date of first KRT	Souce registry
	Destination registry

Collaborating registries and participating countries

Individual patient data from 32 national and regional registries in 16 countries were used to create the tables in Section B of this annual report.

Registry data are subject to continuous alteration and improvement. Small differences between ERA Registry data and the data presented in the national and regional registry's own annual reports may result from different times of data extraction.

Differences between collaborating registries

Definitions and collection of data

In the comparison of data between registries it should be recognized that there may be small differences between registries in definitions and in the collection of their data. For example, the different registries do not collect data at the same level of detail, especially with regard to the various subtypes of treatment modalities.

Patient population

The data from Belgium (Dutch-speaking), Belgium (French-speaking), Spain (Cantabria), Spain (Castile and León), Spain (Castile-La Mancha) and Spain (Navarre) is comprised of patients older than 20 years of age. It has been estimated that the inclusion of data on children would add approximately 2 per million population (Pmp) to the incidence rate and about 13 Pmp to the prevalence.

For the calculation of the incidence of those alive and on KRT on day 91, registries were asked to provide data with follow-up until 31st March 2024. For the following registries data were only available until 31st December 2023: Estonia, France, Spain (Castile and León), Spain (Catalonia), Switzerland, United Kingdom (England/Northern Ireland/Wales) and United Kingdom (Scotland). For these registries the incidence on day 91 was estimated as explained in the paragraph on statistical analyses.

Data loading and cleaning

The data from the registries were imported using an in-house developed import utility. This program first uniforms the data and then translates any non-standard codes. Consistency checks were performed, and thereafter the data were converted to the desired format in order to make the storage of the data in the ERA Registry database possible. The new data, provided by the national and regional registries, replaced all existing data in the database. In this way changes in the data sets made by the national and regional registries were also adopted by the ERA Registry.

After data analysis, the results were checked by the national and regional registries. In the case of discrepancies potential problems regarding (the interpretation of) the data set were solved. Thereafter, more detailed reports were produced which needed approval from the registry representative before publication in this ERA Registry Annual Report.

ERA Registry database

Microsoft SQL Server (a database management system) was used to manage the ERA Registry database. This relational and event-driven database consists of a patient and a transaction table which can be extended according to future needs.

General population data

Midyear population data of the contributing countries were provided by Eurostat [1] or the national bureau of statistics. The sources of the population data used for the analyses in Section B are shown in Table 2. For countries and regions where the coverage of the general population was lower than 100%, population data were adjusted for this lower coverage where applicable.

Reference population

In this year's Annual Report, the age and sex distribution of the EU27 of 2022 as provided by Eurostat [1] was used for the adjustment of the incidence and prevalence for age and sex. The age and sex distribution of EU27 in 2022 is shown in Table 3.

Table 2

Sources of the general population data for the national and regional registries participating with individual patient data in Section B

Registry	Sources of general population data of 2023
Austria	www.statistik.at
Belgium, Dutch-speaking	www.ec.europa.eu/eurostat
Belgium, French-speaking	www.ec.europa.eu/eurostat
Bosnia and Herzegovina	www.popis2013.ba
Denmark	www.ec.europa.eu/eurostat
Estonia	www.ec.europa.eu/eurostat
France	www.ec.europa.eu/eurostat
Greece	www.ec.europa.eu/eurostat
Iceland	www.ec.europa.eu/eurostat
Netherlands	www.ec.europa.eu/eurostat
Norway	www.ec.europa.eu/eurostat
Romania	www.ec.europa.eu/eurostat
Serbia	www.ec.europa.eu/eurostat
Spain, Andalusia	www.ine.es
Spain, Aragon	www.ine.es
Spain, Basque country	www.ine.es
Spain, Canary Islands	www.ine.es
Spain, Cantabria	www.ine.es
Spain, Castile and León	www.ine.es
Spain, Castile-La Mancha	www.ine.es
Spain, Catalonia	www.ine.es
Spain, Community of Madrid	www.ine.es
Spain, Extremadura	www.ine.es
Spain, Galicia	www.ine.es
Spain, La Rioja	www.ine.es
Spain, Murcia	www.ine.es
Spain, Navarre	www.ine.es
Spain, Valencian Region	www.ine.es
Sweden	www.ec.europa.eu/eurostat
Switzerland	www.ec.europa.eu/eurostat
United Kingdom, England/Northern Ireland/Wales	www.ons.gov.uk
United Kingdom, Scotland	www.ons.gov.uk

Table 3

The age and sex distribution of the reference population EU27 (2022)

Age groups	All	Male	Female
	N	N	N
0-4	20.834.819	10.687.875	10.146.944
5-9	22.411.109	11.503.817	10.907.292
10-14	23.673.861	12.159.723	11.514.138
15-19	23.220.320	11.953.413	11.266.907
20-24	23.900.313	12.337.593	11.562.720
25-29	25.335.811	12.953.454	12.382.357
30-34	28.134.334	14.271.548	13.862.786
35-39	29.110.270	14.644.455	14.465.815
40-44	30.646.596	15.366.935	15.279.661
45-49	31.391.394	15.711.728	15.679.666
50-54	32.409.024	16.137.114	16.271.910
55-59	31.794.915	15.675.743	16.119.172
60-64	29.530.602	14.253.247	15.277.355
65-69	26.569.096	12.463.233	14.105.863
70-74	23.458.925	10.695.632	12.763.293
75-79	17.210.908	7.518.978	9.691.930
80-84	13.874.309	5.619.574	8.254.735
+85	13.228.685	4.390.932	8.837.753
Total	446.735.291	218.344.994	228.390.297

Aggregated data

Data collection

In the comparison of data between registries it should be recognized that there may be small differences between registries in definitions and in the collection of their data. For example, the different registries do not collect data at the same level of detail, especially with regard to the various subtypes of treatment modalities.

Collaborating registries

Aggregated data from 19 national and regional registries were used for the preparation of the tables in Section C.

In Italy the availability of variables differed by region. Therefore more extensive results were presented based on the following 10 regions: Calabria, Emilia-Romagna, Friuli Venezia Giulia, Liguria, Marche, Piedmont, Puglia, Sicilia, Valle d'Aosta and Veneto, and additionally more limited results were presented for the following 5 regions: Basilicata, Lazio, Lombardy, Molise and Trentino-Alto Adige.

Data loading and cleaning

The Microsoft Excel template used for data collection includes automated consistency checks. Additional checks were performed manually and data were compared with previous reports. In case of inconsistencies potential problems regarding (the interpretation of) the data set were solved together with the collaborating registry.

General population data

The population data needed for the calculation of incidence and prevalence Pmp were reported by the collaborating registries. If the coverage of the general population in a country by the registry was lower than 100%, population counts were adjusted for this where applicable.

Reference population

As in Section B, we used the age and sex distribution of the EU27 of 2022 (see Table 3) as provided by Eurostat [1] for the adjustment of incidence rates and prevalence.

Coding system

Primary renal disease

Primary renal diseases (PRD) were defined according to both the old (Appendix 1, from 1995) and new (Appendix 2, from 2018) ERA PRD codes and subsequently categorized into groups. Tables showing the old ERA PRD categorization were based on countries that provided either the old or new ERA PRD codes (note that the new ERA PRD codes were assigned to the old ERA PRD groups resulting in a slightly different distribution of PRD groups). Tables showing the new ERA PRD categorization were based only on countries that provided the new ERA PRD codes. Because the new ERA PRD codes were not available for prevalent patients who started KRT before the introduction of the new ERA PRD coding, tables showing the distribution of PRDs according to the new ERA PRD categorization were presented for incident patients only.

Causes of death

Causes of death were defined according to the ERA coding systems and subsequently classified into groups (Appendix 3).

Event type codes

Appendix 4 provides an overview of the coding currently used for event type codes in the ERA Registry database.

Statistical analyses

An overview of the renal registries contributing data for the different types of analyses is given in Appendix 5. For the data analyses SAS 9.4 [2] was used. This statistical software package is able to maintain large datasets, is syntax driven which increases reproducibility of results, and has extensive capabilities with regard to statistics and data management.

Summary section

The tables and figures in the summary section are based on data both from registries providing individual patient data and registries providing aggregated data. Summary statistics were calculated for all registries providing individual patient data, for all registries providing aggregated data and for all registries combined. Where possible these summary statistics were calculated as the sum of the individual values. In all other cases summary statistics were calculated as weighted averages. The weights were dependent on the denominator (i.e. the population size for Pmp values and the total incident count or prevalent count for percentages) and were calculated as the contribution of the registry to the total of the registries.

Individual patient data

Incidence and prevalence

Methods for calculating incidence and prevalence

The incidence and the prevalence of those alive and on KRT were calculated for the total population and for subgroups based on sex, age group, treatment modality, and primary renal disease. The incidence or prevalence Pmp is the observed incident or prevalent count divided by the general population in that year and multiplied by one million. For the incidence or prevalence per million age- related population (Pmarp) the observed incident or prevalent count per age category was divided by the general population in that age category and multiplied by one million.

For those registries that had no data available with a follow-up until March 31st, 2024, the incidence of KRT on day 91 was estimated. We assumed that the relative difference between the incidence on day 1 and the incidence on day 91 was similar for patients who started KRT in the first nine months of 2023, when compared to patients who started KRT in the last three months of 2023.

For the calculation of the mean and median age for incident patients on day 91, we used the actual age on day 91.

Adjustment of incidence and prevalence

Differences in the unadjusted incidence and prevalence across countries might be due to the differences in the age and sex distribution of the general population. The EU27 age and sex distribution of the European population (Table 3) was therefore used to adjust incidence and prevalence for age and sex [1]. Adjusted rates were derived by applying the weights of the reference population to the observed variable-specific rates (e.g. incidence rate per age group) in a country. This weighted average provides a single summary rate for each country that would be expected if that country had the age and sex distribution of the reference population. The following example shows how to calculate the age-adjusted incidence rate. In this example the incidence rate of country A is 269 Pmp, the age-specific rates of country A and the reference population distribution with respect to age are shown in Table 4.

Table 4

Calculation of the adjusted incidence rate

Age groups	Incidence rate country A	Reference population
	Pmp	%
0-19	332	31.7
20-44	261	39.3
45-64	64	29.0
Total	269	100

The adjusted incident rate of country A is: (0.317 * 332) + (0.393 * 261) + (0.29 * 64) = 226 Pmp

Performed kidney transplants

For all participating countries and regions the number of kidney transplants performed during the year was presented by donor type. In Iceland the number of transplants was somewhat lower, because several patients received transplants in other countries. Rates are expressed as numbers, percentages and Pmp.

Survival probability

In this annual report, patient survival on KRT, patient survival on dialysis, patient survival after the first transplant, and graft survival after the first transplant are presented in tables by age, sex and primary renal disease. In addition, patient survival is presented in figures by primary renal disease and by treatment modality. Survival probabilities are presented as percentages from 0 to 100.

For this report the survival analyses were based on data from registries that provided individual patients data for a sufficiently long follow-up period, including Austria, Belgium (Dutch-speaking), Belgium (French-speaking), Bosnia and Herzegovina, Denmark, Estonia, France, Greece, Iceland, Netherlands, Norway, Spain (Andalusia), Spain (Aragon), Spain (Basque country), Spain (Canary Islands), Spain (Cantabria), Spain (Castile and León), Spain (Castile-La Mancha), Spain (Catalonia), Spain (Community of Madrid), Spain (Extremadura), Spain (Galicia), Spain (Murcia), Spain (Navarre), Spain (Valencian region), Sweden, United Kingdom (England/Northern Ireland/Wales) and United Kingdom (Scotland). The differences between the survival probabilities in this annual report and survival probabilities published in earlier annual reports of the ERA Registry may be partly due to differences in participating countries.

Methods

Survival was analysed from day 1 and from day 91 onwards for those patients alive and on KRT on those days. For the analysis of survival data two five-year cohorts were used:

- 2014-2018 - for the presentation of 90-day, one-, two- and five-year survival probabilities.
- 2017-2021 - for the presentation of 90-day, one- and two-year survival probabilities.

31st December 2023 was applied as censoring date for all survival analyses. For the different types of survival analysis the events and reasons for censoring were defined as shown in Table 5.

Table 5

Overview of the events and reasons for censoring as defined for the survival analyses in Section B

Survival type	Event	Censoring
Patients on kidney replacement therapy	Death of patient	Recovery of renal function *<br>Loss to follow-up<br>End of follow-up time
Patients on dialysis	Death of patient	Transplantation<br>Recovery of renal function *<br>Loss to follow-up<br>End of follow-up time
First transplant recipients	Death of patient	Loss to follow-up<br>End of follow-up time
First graft	Death of patient<br>Graft failure<br>Re-transplantation	Loss to follow-up<br>End of follow-up time

* This was only considered as a censored observation when a patient's renal function had recovered for a period of more than 30 days.

Unadjusted survival probabilities

Unadjusted survival probabilities were calculated using the Kaplan-Meier method.

Adjusted survival probabilities

The Cox regression model was used to calculate survival probabilities while accounting for confounders [3]. Survival probabilities were adjusted for age, sex and primary renal disease. As the KRT population changes over time, the survival models are adjusted for fixed values for age, sex and PRD to make the results comparable across time periods. The fixed values of variables used for the survival tables are shown in Table 6.

Patients for whom age, sex, or primary renal disease was missing were excluded.

Table 6

Overview of the variables used to adjust the survival probabilities in Section B

Survival type	Age	Sex	Renal disease
Patients on kidney replacement therapy	67 years	63% male	24% Diabetes<br>19% Hypertension / renal vascular disease<br>11% Glomerulonephritis<br>46% Other causes
Patients on dialysis	67 years	63% male	24% Diabetes<br>19% Hypertension / renal vascular disease<br>11% Glomerulonephritis<br>46% Other causes
First transplant recipients (deceased donor)	50 years	63% male	14% Diabetes<br>10% Hypertension / renal vascular disease<br>23% Glomerulonephritis<br>53% Other causes
First transplant recipients (living donor)	50 years	63% male	14% Diabetes<br>10% Hypertension / renal vascular disease<br>23% Glomerulonephritis<br>53% Other causes

Confidence intervals

When survival probabilities were calculated using the Kaplan-Meier method or Cox regression method, the corresponding confidence intervals were calculated according to Bie et al. [4], using the standard error.

Expected remaining lifetimes

Expected remaining lifetimes represent the average number of years of life remaining for those who have reached a given age. In this annual report, expected remaining lifetimes were calculated for patients receiving KRT and for the general population. For the calculation of expected remaining lifetimes of both groups, we adopted the methodology used by the United States Renal Data System (USRDS)[5].

In order to calculate expected remaining lifetimes we used data from registries providing complete follow-up data for the period from 2019 to 2023. The data of the following national or regional registries from 13 countries were included for this analysis: Austria, Belgium (Dutch- speaking), Belgium (French- speaking), Bosnia and Herzegovina, Denmark, Estonia, France, Greece, Iceland, Netherlands, Norway, Spain (Andalusia), Spain (Aragon), Spain (Basque Country), Spain (Canary Islands), Spain (Cantabria), Spain (Castile and León), Spain (Castile-La Mancha), Spain (Catalonia), Spain (Community of Madrid), Spain (Extremadura), Spain (Galicia), Spain (Murcia), Spain (Navarre), Spain (Valencian region), Sweden, United Kingdom (England, Northern Ireland and Wales), and United Kingdom (Scotland). With regard to the inclusion of the data, it should be noted that the differences between the expected remaining lifetimes of this annual report and expected remaining lifetimes published in earlier annual reports of the ERA Registry may be partly due to differences in participating countries.

KRT population

The expected remaining lifetimes for KRT patients are presented by age, sex and treatment modality. The expected remaining lifetime for a certain patient group is the average of the remaining life expectancies for the patients in that group. Although this cannot be known until all the patients in the group have died, the expected remaining lifetime can be projected by assuming that patients in the patient group will die at the same rates as those observed among older groups of recent prevalent KRT patients.

General population

The number of deaths Pmp by sex and age of the general population of the contributing countries were provided by Eurostat [1]. The size of the contribution of each country to the general population that was used to calculate expected remaining lifetimes was in proportion to the size of the general population covered by its renal registry.

Aggregated data

Incidence and prevalence counts were provided by the contributing registries. To increase the consis- tency of the data, general explanations of how to complete the Microsoft Excel template and how the adjusted incidence and prevalence were calculated were provided to support the participating registries.

Paediatric Section

The incidence and prevalence tables in Section D of this annual report were based on data from 26 national or regional registries from 15 countries that provided complete individual patient data in their paediatric population for 2018 to 2023, including Austria, Bosnia and Herzegovina, Denmark, Estonia, France, Greece, Iceland, Netherlands, Norway, Romania, Serbia, Spain (Andalusia), Spain (Aragon), Spain (Basque country), Spain (Canary Islands), Spain (Catalonia), Spain (Community of Madrid), Spain (Extremadura), Spain (Galicia), Spain (La Rioja), Spain (Murcia), Spain (Valencian Region), Sweden, Switzerland, United Kingdom (England, Northern Ireland and Wales) and United Kingdom (Scotland). Since not all countries provided data or had full coverage over the entire period, and to allow for comparison across years, data are shown both including and excluding the data from these countries. The years for which data was available and/or the coverage are indicated below the tables.

Furthermore, as not every region in Spain had a specialised paediatric centre, paediatric patients could be treated in other regions than their resident region. This would result in an underestimation of the incidence and prevalence in these regions. To allow for this, non-resident paediatric patients were not excluded from the paediatric tables.

Incidence (on day 1) and prevalence on KRT were calculated for subgroups based on age, treatment modality, and primary renal disease. Incidence on KRT by 2-year cohorts was presented for paediatric patients who started KRT between 2018 and 2023. The incidence rate on KRT (Pmarp) was the observed incident count in that particular time period divided by the age-related general population in that time period (for example the total population in 2020 plus the total population in 2021) multiplied by one million. The grouping of primary renal disease codes for the paediatric population is described in Table 7 as it differs from the grouping in the adult population

Table 7

Grouping of primary renal diseases for paediatric patients in Section D

Groups and codes included

**Primary renal disease group**	**Primary renal disease code** (see appendix 1)
Congenital anomalies of the kidney and urinary tract (CAKUT)	20, 21, 22, 23, 24, 25, 29, 60, 61, 63, and 66
Glomerulonephritis	10, 11, 12, 13, 14, 15, 16, 17, 19, and 86
Cystic kidney disease	40, 41, 42, 43, and 49
Hereditary nephropathy	50, 51, and 59
Ischaemic renal failure	90
Haemolytic uraemic syndrome (HUS)	88
Metabolic disorders	52, 53, 54, 92, and 93
Vasculitis	73, 74, 84, 85, and 87
Miscellaneous	30, 31, 32, 33, 34, 39, 70, 71, 72, 75, 76, 78, 79, 80, 81, 82, 83, 89, 91, 94, 95, 96, and 99
Missing, unknown	missing and 00

The prevalence on KRT was presented on 31st December of each year from 2018 to 2023. The prevalence on KRT by primary renal disease was calculated for patients prevalent on 31st December, 2023.

Bibliography

1. Eurostat: www.ec.europa.eu/eurostat/data/database
2. SAS Institute Inc., Cary, NC, USA.
3. Altman, D. Practical statistics for medical research. second ed. 1999, London: Chapman & Hall.
4. Bie O, Borgan O and Liestol K. Confidence intervals and confidence bands for the cumulative hazard rate function and their small sample properties. Scandinavian Journal of Statistics 1987; 14: 221-233.
5. U.S. Renal Data System, USRDS 2012 Annual Data Report: Atlas of End-Stage Renal Disease in the United States, National Institutes of Health, National Institute of Diabetes and Digestive and Kidney Diseases, Bethesda, MD, 2012.

Appendices

Appendix 1 - Grouping of primary renal disease codes 1995

1994 code	1995 code	PRD group	Primary renal disease
10	10	I	Glomerulonephritis; histologically NOT examined
11	11	I	Focal segmental glomerulosclerosis with nephrotic syndrome in children
12	12	I	IgA nephropathy (proven by immunofluorescence, not code 76 or 85)
13	13	I	IDense deposit disease; membrano-proliferative GN; type II (proven by immunofluore / electron microscopy)
14	14	I	Membranous nephropathy
15	15	I	Membrano-proliferative GN; type I proven by immunofluorescence / electron microscopy, not code 84 or 89)
16	16	I	Crescentic (extracapillary) glomerulonephritis (type I, II, III)
17	17	I	Focal segmental glomerulosclerosis with nephrotic syndrome in adults
19	19	I	Glomerulonephritis; histologically examined, not given above
20	20	II	Pyelonephritis; cause not specified
21	21	II	Pyelonephritis associated with neurogenic bladder
22	22	II	Pyelonephritis due to congenital obstructive uropathy with/without vesico-ureteric reflux
23	23	II	Pyelonephritis due to acquired obstructive uropathy
24	24	II	Pyelonephritis due to vesico-ureteric reflux without obstruction
25	25	II	Pyelonephritis due to urolithiasis
29	29	II	Pyelonephritis due to other cause
30	30	VII	Interstitial nephritis (not pyelonephritis) due to other cause, or unspecified (not mentioned above)
31	31	VII	Nephropathy (interstitial) due to analgesic drugs
32	32	VII	Nephropathy (interstitial) due to cis-platinum
33	33	VII	Nephropathy (interstitial) due to cyclosporin A
	34	VII	Lead induced nephropathy (interstitial)
39	39	VII	Drug induced nephropathy (interstitial) not mentioned above
40	40	VII	Cystic kidney disease - type unspecified
41	41	III	Polycystic kidneys; adult type (dominant)
42	42	VII	Polycystic kidneys; infantile (recessive)
43	43	VII	Medullary cystic disease; including nephronophthisis
49	49	VII	Cystic kidney disease - other specified type
50	50	VII	Hereditary / Familial nephropathy - type unspecified
51	51	VII	Hereditary nephritis with nerve deafness (Alport's Syndrome)
52	52	VII	Cystinosis
53	53	VII	Primary oxalosis
54	54	VII	Fabry's disease
59	59	VII	Hereditary nephropathy - other specified type
60	60	VII	Renal hypoplasia (congenital) - type unspecified
61	61	VII	Oligomeganephronic hypoplasia
63	63	VII	Congenital renal dysplasia with or without urinary tract malformation
66	66	VII	Syndrome of agenesis of abdominal muscles (Prune Belly)
70	70	V	Renal vascular disease - type unspecified
71	71	IV	Renal vascular disease due to malignant hypertension
72	72	IV	Renal vascular disease due to hypertension
73	73	VII	Renal vascular disease due to polyarteritis
74	74	VII	Granulomatosis with polyangiitis
	75	VII	Ischaemic renal disease/cholesterol embolism (1998 prd code)
76	76	VII	Glomerulonephritis related to liver cirrhosis
	78	VII	Cryoglobulinaemic glomerulonephritis
79	79	V	Renal vascular disease - due to other cause (not given above and not code 84-88)
80	80	VI	Diabetes glomerulosclerosis or diabetic nephropathy - Type I
81	80	VI	Diabetes glomerulosclerosis or diabetic nephropathy - Type II
82	82	VII	Myelomatosis / light chain deposit disease
83	83	VII	Amyloid
84	84	VII	Lupus erythematosus
85	85	VII	Henoch-Schoenlein purpura
86	86	VII	Goodpasture's Syndrome
87	87	VII	Systemic sclerosis (scleroderma)
88	88	VII	Haemolytic Uraemic Syndrome (including Moschcowitz Syndrome)
89	89	VII	Multi-system disease - other (not mentioned above)
90	90	VII	Tubular necrosis (irreversible) or cortical necrosis (different from 88)
91	91	VII	Tuberculosis
92	92	VII	Gout
93	93	VII	Nephrocalcinosis and hypercalcaemic nephropathy
94	94	VII	Balkan nephropathy
95	95	VII	Kidney tumour
96	96	VII	Traumatic or surgical loss of kidney
99	99	VII	Other identified renal disorders
00	00	VIII	Chronic renal failure; aetiology uncertain

PRD group: I: glomerulonephritis / sclerosis; II: pyelonephritis; III: polycystic kidneys, adult type; IV: hypertension; V: renal vascular disease; VI: diabetes; VII: miscellaneous; VIII: unknown

Appendix 2 - Grouping of primary renal disease codes 2018

I: GLOMERULAR DISEASE

Code	Primary renal disease
1003	Adult nephrotic syndrome - no histology
1472	Anti-Glomerular basement membrane (GBM) disease / Goodpasture's syndrome - histologically proven
1464	Anti-Glomerular basement membrane (GBM) disease / Goodpasture's syndrome - no histology
1440	Churg-Strauss syndrome - histologically proven
1438	Churg-Strauss syndrome - no histology
1088	Congenital nephrotic syndrome (CNS) - congenital infection
1057	Congenital nephrotic syndrome (CNS) - diffuse mesangial sclerosis
1042	Congenital nephrotic syndrome (CNS) - Finnish type - histologically proven
1035	Congenital nephrotic syndrome (CNS) - Finnish type - no histology
1061	Congenital nephrotic syndrome (CNS) - focal segmental glomerulosclerosis (FSGS)
1026	Congenital nephrotic syndrome (CNS) - no histology
1570	Cryoglobulinaemia secondary to hepatitis C - histologically proven
1562	Cryoglobulinaemia secondary to hepatitis C - no histology
1591	Cryoglobulinaemia secondary to systemic disease - histologically proven
1589	Cryoglobulinaemia secondary to systemic disease - no histology
1074	Denys-Drash syndrome
1331	Diffuse endocapillary glomerulonephritis
1558	Essential mixed cryoglobulinaemia - histologically proven
1543	Essential mixed cryoglobulinaemia - no histology
1308	Familial focal segmental glomerulosclerosis (FSGS) - autosomal dominant - histologically proven
1298	Familial focal segmental glomerulosclerosis (FSGS) - autosomal dominant - no histology
1280	Familial focal segmental glomerulosclerosis (FSGS) - autosomal recessive - histologically proven
1279	Familial focal segmental glomerulosclerosis (FSGS) - autosomal recessive - no histology
1144	Familial IgA nephropathy - histologically proven
1137	Familial IgA nephropathy - no histology
1354	Focal and segmental proliferative glomerulonephritis
3754	Focal segmental glomerulosclerosis (FSGS) secondary to HIV
3765	Focal segmental glomerulosclerosis (FSGS) secondary to lithium
1320	Focal segmental glomerulosclerosis (FSGS) secondary to obesity - histologically proven
1312	Focal segmental glomerulosclerosis (FSGS) secondary to obesity - no histology
3777	Focal segmental glomerulosclerosis (FSGS) secondary to sickle cell
1377	Glomerulonephritis - histologically indeterminate
3749	Glomerulonephritis - no histology
1365	Glomerulonephritis - secondary to systemic disease
1417	Granulomatosis with polyangiitis - histologically proven
1401	Granulomatosis with polyangiitis - no histology
1515	Henoch-Schönlein purpura / nephritis - histologically proven
1504	Henoch-Schönlein purpura / nephritis - no histology
1251	Idiopathic rapidly progressive (crescentic) glomerulonephritis
1128	IgA nephropathy - histologically proven
1116	IgA nephropathy - no histology
1163	IgA nephropathy secondary to liver cirrhosis - histologically proven
1159	IgA nephropathy secondary to liver cirrhosis - no histology
1171	IgM - associated nephropathy
1205	Membranous nephropathy - drug induced
1185	Membranous nephropathy - idiopathic
1214	Membranous nephropathy - infection associated
1192	Membranous nephropathy - malignancy associated
1349	Mesangial proliferative glomerulonephritis
1222	Mesangiocapillary glomerulonephritis type 1
1233	Mesangiocapillary glomerulonephritis type 2 (dense deposit disease)
1246	Mesangiocapillary glomerulonephritis type 3
1429	Microscopic polyangiitis - histologically proven
1100	Minimal change nephropathy - histologically proven
1090	Minimal change nephropathy - no histology
3615	Nephrotic syndrome of childhood - no trial of steroids - no histology
3604	Nephrotic syndrome of childhood - steroid resistant - no histology
1019	Nephrotic syndrome of childhood - steroid sensitive - no histology
1455	Polyarteritis nodosa
1267	Primary focal segmental glomerulosclerosis (FSGS)
1536	Renal scleroderma / systemic sclerosis - histologically proven
1527	Renal scleroderma / systemic sclerosis - no histology
1493	Systemic lupus erythematosus / nephritis - histologically proven
1486	Systemic lupus erythematosus / nephritis - no histology
1383	Systemic vasculitis - ANCA negative - histologically proven
3847	Systemic vasculitis - ANCA negative - no histology
3852	Systemic vasculitis - ANCA positive - histologically proven
1396	Systemic vasculitis - ANCA positive - no histology

Appendix 2 - Grouping of primary renal disease codes 2018

II: TUBULOINTERSTITIAL DISEASE

Code	Primary renal disease
1768	Acquired obstructive nephropathy due to neurogenic bladder
1752	Acquired obstructive uropathy / nephropathy
2196	Acute urate nephropathy - histologically proven
2183	Acute urate nephropathy - no histology
1995	Aristolochic acid nephropathy (Balkan / Chinese herb / endemic nephropathy) - histologically proven
1982	Aristolochic acid nephropathy (Balkan / Chinese herb / endemic nephropathy) - no histology
1710	Bladder exstrophy
1845	Calcium oxalate urolithiasis
1832	Calculus nephropathy / urolithiasis
2203	Chronic urate nephropathy - histologically proven
3636	Chronic urate nephropathy - no histology
1625	Congenital dysplasia / hypoplasia
1706	Congenital neurogenic bladder
1660	Congenital pelvi-ureteric junction obstruction
1673	Congenital vesico-ureteric junction obstruction
2005	Drug-induced tubulointerstitial nephritis - no histology
2014	Drug-induced tubulointerstitial nephritis - histologically proven
1850	Enteric hyperoxaluria
1911	Familial interstitial nephropathy - histologically proven
1907	Familial interstitial nephropathy - no histology
1618	Familial reflux nephropathy
1656	Glomerulocystic disease
2257	Hantavirus nephropathy
3662	Hypercalcaemic nephropathy
1813	Idiopathic retroperitoneal fibrosis
2177	Lead induced nephropathy - histologically proven
2165	Lead induced nephropathy - no histology
2242	Leptospirosis
1866	Magnesium ammonium phosphate (struvite) urolithiasis
1723	Megacystis-megaureter
1639	Multicystic dysplastic kidneys
2098	Nephropathy due to aminoglycosides - histologically proven
2080	Nephropathy due to aminoglycosides - no histology
2112	Nephropathy due to amphotericin - histologically proven
2108	Nephropathy due to amphotericin - no histology
2033	Nephropathy due to analgesic drugs - histologically proven
2022	Nephropathy due to analgesic drugs - no histology
2051	Nephropathy due to ciclosporin - histologically proven
2046	Nephropathy due to ciclosporin - no histology
2131	Nephropathy due to cisplatin - histologically proven
2120	Nephropathy due to cisplatin - no histology
2154	Nephropathy due to lithium - histologically proven
2149	Nephropathy due to lithium - no histology
2079	Nephropathy due to tacrolimus - histologically proven
2067	Nephropathy due to tacrolimus - no histology
2288	Nephropathy related to HIV - histologically proven
2274	Nephropathy related to HIV - no histology
1799	Obstructive nephropathy due to bladder cancer
1809	Obstructive nephropathy due to other malignancies
1781	Obstructive nephropathy due to prostate cancer
1775	Obstructive nephropathy due to prostatic hypertrophy
1734	Oligomeganephronia
2300	Other specific infection
1687	Posterior urethral valves
1602	Primary reflux nephropathy - sporadic
2219	Radiation nephritis
2226	Renal / perinephric abscess
3627	Renal cysts and diabetes syndrome
1641	Renal dysplasia due to fetal ACE-inhibitor exposure
1747	Renal papillary necrosis
3796	Renal papillary necrosis caused by analgesics
3783	Renal papillary necrosis caused by diabetes
3806	Renal papillary necrosis caused by sickle cell
1976	Renal sarcoidosis - histologically proven
1969	Renal sarcoidosis - no histology

Appendix 2 - Grouping of primary renal disease codes 2018

II: TUBULOINTERSTITIAL DISEASE (continued)

Code	Primary renal disease
2235	Renal tuberculosis
3689	Retroperitoneal fibrosis secondary to drugs
1821	Retroperitoneal fibrosis secondary to malignancies
3670	Retroperitoneal fibrosis secondary to peri-aortitis
2290	Schistosomiasis
1694	Syndrome of agenesis of abdominal muscles - prune belly syndrome
1897	Tubulointerstitial nephritis - histologically proven
1884	Tubulointerstitial nephritis - no histology
1930	Tubulointerstitial nephritis associated with autoimmune disease - histologically proven
1924	Tubulointerstitial nephritis associated with autoimmune disease - no histology
1953	Tubulointerstitial nephritis with uveitis (TINU) - histologically proven
1948	Tubulointerstitial nephritis with uveitis (TINU) - no histology
1878	Uric acid urolithiasis
2261	Xanthogranulomatous pyelonephritis

III: DIABETES MELLITUS

Code	Primary renal disease
2328	Diabetic nephropathy in type I diabetes - histologically proven
2316	Diabetic nephropathy in type I diabetes - no histology
2344	Diabetic nephropathy in type II diabetes - histologically proven
2337	Diabetic nephropathy in type II diabetes - no histology

IV: HYPERTENSION / RENAL VASCULAR DISEASE

Code	Primary renal disease
2392	Ageing kidney - no histology
2448	Atheroembolic renal disease - histologically proven
2430	Atheroembolic renal disease - no histology
2363	Chronic hypertensive nephropathy - histologically proven
2359	Chronic hypertensive nephropathy - no histology
2453	Fibromuscular dysplasia of renal artery
2407	Ischaemic nephropathy - no histology
2411	Ischaemic nephropathy / microvascular disease - histologically proven
2385	Malignant hypertensive nephropathy / accelerated hypertensive nephropathy - histologically proven
2371	Malignant hypertensive nephropathy / accelerated hypertensive nephropathy - no histology
2469	Renal arterial thrombosis / occlusion
2424	Renal artery stenosis

V: OTHER SYSTEMIC DISEASES AFFECTING THE KIDNEY

Code	Primary renal disease
2513	AA amyloid secondary to chronic inflammation
2521	AL amyloid secondary to plasma cell dyscrasia
2623	Atypical haemolytic uraemic syndrome (HUS) - diarrhoea negative
2482	Cardiorenal syndrome
2652	Congenital haemolytic uraemic syndrome (HUS)
2566	Familial AA amyloid secondary to familial Mediterranean fever / TRAPS (Hibernian fever) - histologically proven
2550	Familial AA amyloid secondary to familial Mediterranean fever / TRAPS (Hibernian fever) - no histology
2545	Familial amyloid secondary to protein mutations - histologically proven
2532	Familial amyloid secondary to protein mutations - no histology
2668	Familial haemolytic uraemic syndrome (HUS)
2675	Familial thrombotic thrombocytopenic purpura (TTP)
2610	Haemolytic uraemic syndrome (HUS) - diarrhoea associated
2647	Haemolytic uraemic syndrome (HUS) secondary to systemic disease
2495	Hepatorenal syndrome
2606	Immunotactoid / fibrillary nephropathy
2597	Light chain deposition disease
2584	Myeloma cast nephropathy - histologically proven
2578	Myeloma kidney - no histology
2681	Nephropathy due to eclampsia
3834	Nephropathy due to pre-eclampsia
2509	Renal amyloidosis
2476	Renal vein thrombosis
2702	Sickle cell nephropathy - histologically proven
2699	Sickle cell nephropathy - no histology
2634	Thrombotic thrombocytopenic purpura (TTP)

Appendix 2 - Grouping of primary renal disease codes 2018

VI: FAMILIAL / HEREDITARY NEPHROPATHIES

Code	Primary renal disease
3071	Alagille syndrome
2760	Alport syndrome - histologically proven
2756	Alport syndrome - no histology
3118	Apparent mineralocorticoid excess
2718	Autosomal dominant (AD) polycystic kidney disease
2725	Autosomal dominant (AD) polycystic kidney disease type I
2739	Autosomal dominant (AD) polycystic kidney disease type II
2741	Autosomal recessive (AR) polycystic kidney disease
3085	Bartter syndrome
2773	Benign familial haematuria
3322	Branchio-oto-renal syndrome
2794	Cystic kidney disease
2964	Cystinosis
2955	Cystinuria
2929	Dent disease
3028	Distal renal tubular acidosis (RTA) - type I
3037	Distal renal tubular acidosis with sensorineural deafness - gene mutations
3230	Fabry disease - histologically proven
3224	Fabry disease - no histology
3173	Familial hypercalciuric hypocalcaemia
3160	Familial hypocalciuric hypercalcaemia
3187	Familial hypomagnesaemia
3379	Familial nephropathy
3314	Frasier syndrome
3092	Gitelman syndrome
3125	Glucocorticoid suppressible hyperaldosteronism
3305	Horse-shoe kidney
2993	Hypophosphataemic rickets autosomal recessive (AR)
2986	Hypophosphataemic rickets X-linked (XL)
3139	Inherited / genetic diabetes mellitus type II
2940	Inherited aminoaciduria
2972	Inherited renal glycosuria
3810	Kidney stones due to ARPT deficiency
3351	Lawrence-Moon-Biedl / Bardet-Biedl syndrome
3059	Lesch Nyhan syndrome - hypoxanthine guanine phosphoribosyl transferase deficiency
3102	Liddle syndrome
2938	Lowe syndrome (oculocerebrorenal syndrome)
2804	Medullary cystic kidney disease type I
2815	Medullary cystic kidney disease type II
3295	Medullary sponge kidneys
3367	Mitochondrial cytopathy
3253	Nail-patella syndrome
3044	Nephrogenic diabetes insipidus
2836	Nephronophthisis
2843	Nephronophthisis - type 1 (juvenile type)
2858	Nephronophthisis - type 2 (infantile type)
2862	Nephronophthisis - type 3 (adolescent type)
2870	Nephronophthisis - type 4 (juvenile type)
2889	Nephronophthisis - type 5
2891	Nephronophthisis - type 6
3063	Phosphoribosyl pyrophosphate synthetase (PRPPS) superactivity
2901	Primary Fanconi syndrome
3194	Primary hyperoxaluria
3207	Primary hyperoxaluria type I
3211	Primary hyperoxaluria type II
3731	Primary hyperoxaluria type III
3000	Primary renal tubular acidosis (RTA)
3016	Proximal renal tubular acidosis (RTA) - type II
3141	Pseudohypoaldosteronism type 1
3156	Pseudohypoaldosteronism type 2 (Gordon syndrome)
3658	Renal coloboma syndrome
3269	Rubinstein-Taybi syndrome
2787	Thin basement membrane disease
3346	Townes-Brocks syndrome
3276	Tuberous sclerosis
2917	Tubular disorder as part of inherited metabolic diseases
2827	Uromodulin-associated nephropathy (familial juvenile hyperuricaemic nephropathy)
3282	Von Hippel-Lindau disease
3333	Williams syndrome
3248	Xanthinuria

Appendix 2 - Grouping of primary renal disease codes 2018

VII: MISCELLANEOUS RENAL DISORDERS

Code	Primary renal disease
3442	Acute cortical necrosis
3380	Acute kidney injury
3403	Acute kidney injury due to circulatory failure
3398	Acute kidney injury due to hypovolaemia
3435	Acute kidney injury due to nephrotoxicity
3426	Acute kidney injury due to rhabdomyolysis
3419	Acute kidney injury due to sepsis
3457	Acute pyelonephritis
3564	Chronic kidney disease (CKD) / chronic renal failure (CRF) - aetiology uncertain / unknown - histologically proven
3555	Chronic kidney disease (CKD) / chronic renal failure (CRF) - aetiology uncertain / unknown - no histology
3529	Chronic kidney disease (CKD) / chronic renal failure (CRF) caused by tumour nephrectomy
3540	Chronic kidney disease (CKD) / chronic renal failure (CRF) due to donor nephrectomy
3538	Chronic kidney disease (CKD) / chronic renal failure (CRF) due to traumatic loss of kidney
3708	Chronic renal failure
3643	Chronic renal failure due to systemic infection
3572	Haematuria and proteinuria - no histology
3823	Infiltration by lymphoma - histologically proven
3712	Isolated haematuria - no histology
3720	Isolated proteinuria - no histology
3461	Kidney tumour
3501	Mesoblastic nephroma - histologically proven
3474	Renal cell carcinoma - histologically proven
3691	Renal failure
3517	Single kidney identified in adulthood
3488	Transitional cell carcinoma - histologically proven
3490	Wilms tumour - histologically proven

Appendix 3 - Grouping of causes of death

1994 code	1995 code	COD group	Cause of death
0	0	XI	Cause of death uncertain / not determined
11	11	I	Myocardial ischaemia and infarction
12	12	X	Hyperkalaemia
13	13	X	Haemorrhagic pericarditis
14	14	II	Other causes of cardiac failure
15	15	III	Cardiac arrest / sudden death; other cause or unknown
16	16	II	Hypertensive cardiac failure
17	17	X	Hypokalaemia
18	18	II	Fluid overload / pulmonary oedema
21	21	X	Pulmonary embolus
22	22	IV	Cerebro-vascular accident, other cause or unspecified
23	23	X	Gastro-intestinal haemorrhage
24	24	X	Haemorrhage from graft site
25	25	X	Haemorrhage from vascular access or dialysis circuit
26	26	X	Haemorrhage from ruptured vascular aneurysm (not code 22 or 23)
27	27	X	Haemorrhage from surgery (not code 23, 24 or 26)
28	28	X	Other haemorrhage (not codes 23-27)
29	29	X	Mesenteric infarction
31	31	V	Pulmonary infection (bacterial - not code 73)
32	32	V	Pulmonary infection (viral)
33	33	V	Pulmonary infection (fungal or protozoal; parasitic)
34		V	Infections elsewhere except virus hepatitis
35	35	V	Septicaemia
36	36	V	Tuberculosis (lung)
37	37	V	Tuberculosis (elsewhere)
38	38	V	Generalized viral infection
39	39	V	Peritonitis (all causes except for Peritoneal Dialysis)
41	41	X	Liver disease due to hepatitis B virus
42	42	X	Liver disease due to other viral hepatitis
43	43	X	Liver disease due to drug toxicity
44	44	X	Cirrhosis - not viral
45	45	X	Cystic liver disease
46	46	X	Liver failure - cause unknown
51	51	VI	Patient refused further treatment for ESRD
52	52	VI	Suicide
53	53	VII	ESRD treatment ceased for any other reason
	54	VII	ESRD treatment withdrawn for medical reasons
61		X	Uremia caused by graft failure
62	62	X	Pancreatitis
63	63	X	Bone marrow depression
64	64	VIII	Cachexia
66	66	IX	Malignant disease, possibly induced by immunosuppressive therapy
67	67	IX	Malignant disease: solid tumors except those of 66
	68	IX	Malignant disease: lymphoproliferative disorders except those of 66
69	69	X	Dementia
70	70	V	Peritonitis (sclerosing, with peritoneal dialysis)
71	71	X	Perforation of peptic ulcer
72	72	X	Perforation of colon
73	73	X	Chronic obstructive airways disease
81	81	X	Accident related to ESRD treatment (not code 25)
82	82	X	Accident unrelated to ESRD treatment
	100	V	Peritonitis (bacterial, with peritoneal dialysis)
	101	V	Peritonitis (fungal, with peritoneal dialysis)
	102	V	Peritonitis (due to other cause, with peritoneal dialysis)
99	99	X	Other identified cause of death

COD group: I: myocardial ischaemia and infarction; II: heart failure; III: cardiac arrest, other cause / unknown; IV: cerebrovascular accident; V: infection; VI: suicide / refusal treatment; VII: withdrawal; VIII: cachexia; IX: malignancies; X: miscellaneous; XI: unknown / unavailable

Appendix 4 - Event type codes

Event code	Event
001	Home haemodialysis
002	Centre haemodialysis
003	Haemofiltration
004	Haemodiafiltration
009	Haemodialysis, type unknown
011	Continuous Ambulatory Peritoneal Dialysis (CAPD)
012	Intermittent Ambulatory Peritoneal Dialysis (CAPD)
013	Continuous Automated Peritoneal Dialysis (APD)
014	Intermittent Automated Peritoneal Dialysis (APD)
019	Peritoneal Dialysis, type unknown
090	Unknown dialysis
020	Transplantation, living donor
021	Transplantation, living related donor
022	Transplantation, living unrelated donor
023	Transplantation, deceased donor
029	Transplantation, donor type unknown
060	Transplantation follow-up (still functioning)
061	Graft failure
031	Treatment type unknown / unavailable
051	Recovery Renal Function
052	Death
053	Transfer out to other registry
054	Transfer in from other registry
055	Lost to follow-up
071	Limited care / stopped treatment (without recovery of renal function)

Appendix 5 - Renal registries contributing data for the different types of analyses

	Registries providing individual patient data			Registries providing aggregated data	Registries providing paedriatic data
	Incidence and prevalence	Survival	Expected remaining lifetimes	Incidence and prevalence	Incidence and prevalence
Albania	-	-	-	+	-
Austria	+	+	+	-	+
Belarus	-	-	-	+	-
Belgium, Dutch-speaking	+	+	+	-	-
Belgium, French-speaking	+	+	+	-	-
Bosnia and Herzegovina	+	+	+	-	+
Croatia	-	-	-	+	-
Cyprus	-	-	-	+	-
Czech Republic	-	-	-	+	-
Denmark	+	+	+	-	+
Estonia	+	+	+	-	+
Finland	-	-	-	+	-
France	+	+	+	-	+
Greece	+	+	+	-	+
Hungary	-	-	-	+	-
Iceland	+	+	+	-	+
Ireland	-	-	-	+	-
Israel	-	-	-	+	-
Italy (10 of 20 regions)	-	-	-	+	-
Italy (5 extra regions)	-	-	-	+	-
Kosovo	-	-	-	+	-
Latvia	-	-	-	+	-
Lithuania	-	-	-	+	-
Netherlands	+	+	+	-	+
North Macedonia	-	-	-	+	-
Norway	+	+	+	-	+
Poland	-	-	-	+	-
Portugal	-	-	-	+	-
Romania	+	-	-	-	+
Serbia	+	-	-	-	+
Slovakia	-	-	-	+	-
Spain, Andalusia	+	+	+	+	+
Spain, Aragon	+	+	+	+	+
Spain, Asturias	-	-	-	+	-
Spain, Balearic Islands	-	-	-	+	-
Spain, Basque country	+	+	+	+	+
Spain, Canary Islands	+	+	+	+	+
Spain, Cantabria	+	+	+	+	-
Spain, Castile and León	+	+	+	+	-
Spain, Castile-La Mancha	+	+	+	+	-
Spain, Catalonia	+	+	+	+	+
Spain, Ceuta	-	-	-	+	-
Spain, Community of Madrid	+	+	+	+	+
Spain, Extremadura	+	+	+	+	+
Spain, Galicia	+	+	+	+	+
Spain, La Rioja	+	-	-	+	+
Spain, Melilla	-	-	-	+	-
Spain, Murcia	+	+	+	+	+
Spain, Navarre	+	+	+	+	-
Spain, Valencian region	+	+	+	+	+
Sweden	+	+	+	-	+
Switzerland	+	-	-	-	+
Türkiye	-	-	-	+	-
United Kingdom, England	+	+	+	-	+
United Kingdom, Northern Ireland	+	+	+	-	+
United Kingdom, Scotland	+	+	+	-	+
United Kingdom, Wales	+	+	+	-	+

FIND

ERA Registry
Amsterdam UMC, location AMC
Department of Medical Informatics
PO Box 22700
1100 DE Amsterdam
Netherlands

November 2025

Amsterdam UMC
University Medical Centers

FIND
